# NanoLuc Luciferase as a Fluorogen-Activating Protein for GFP Chromophore Based Fluorogens

**DOI:** 10.3390/ijms24097958

**Published:** 2023-04-27

**Authors:** Yulia A. Bogdanova, Elvira R. Zaitseva, Alexander Yu. Smirnov, Nadezhda S. Baleeva, Alexey S. Gavrikov, Ivan N. Myasnyanko, Sergey A. Goncharuk, Erik F. Kot, Konstantin S. Mineev, Alexander S. Mishin, Mikhail S. Baranov

**Affiliations:** 1 Institute of Bioorganic Chemistry, Russian Academy of Sciences, Miklukho-Maklaya 16/10, 117997 Moscow, Russia; bogdanova.biochem@gmail.com (Y.A.B.);; 2 Laboratory of Medicinal Substances Chemistry, Institute of Translational Medicine, Pirogov Russian National Research Medical University, Ostrovitianov 1, 117997 Moscow, Russia; 3 Moscow Institute of Physics and Technology, 141701 Dolgoprudny, Russia

**Keywords:** GFP, Kaede, fluorogen, fluorescence, bioluminescence, nanoLuc

## Abstract

In this work, we showed that the well-known NanoLuc luciferase can act as a fluorogen activating protein for various arylidene-imidazolones structurally similar to the Kaede protein chromophore. We showed that such compounds can be used as fluorescent sensors for this protein and can also be used in pairs with it in fluorescent microscopy as a genetically encoded tag.

## 1. Introduction

Fluorescent labeling is a common technique in modern science. Various tags are widely used for the visualization of the processes occurring in living cells and organisms. Among these tags, a special place is occupied by genetically encoded labels [1]. Fluorescent proteins (FPs) are the most commonly used in this role due to their variety of colors, lifetimes and many other properties [2,3]. However, such proteins have many intrinsic insolvable drawbacks. Their chromophores are formed from their own amino acid residues, and therefore, their maturation takes time and requires oxygen. Moreover, when bleached, these chromophores cannot recover, and FP molecules become non-fluorescent. The latter drawback also applies to many other tags, including those based on low-molecular-weight fluorescent dyes. Thereby, in recent decades, fluorescent tags based on fluorogens have become more and more popular [4]. This group of compounds does not have prominent fluorescence in the free state in solutions but can obtain it upon binding to a specific target. This feature has made them very efficient in fluorescence microscopy of living systems since their background signal is extremely low. Aryliden-azolones and especially aryliden-imidazolones are the most important examples of this dye class [5]. These substances represent the structural core of the above-mentioned FP chromophores and are characterized by a noticeable variety of colors, good solubility in water and simple chemical syntheses [6]. Recently, aryliden-imidazolones have been successfully used for fluorescent labeling of nucleic acids [7,8,9,10] and other cell components [11,12,13]. However, they have found even greater use as ligands for fluorogen-activating proteins (FAPs), an alternative type of genetically encoded tag [14,15,16,17]. These proteins do not have their own internal chromophore but have a pocket capable of fluorogen binding, thereby activating their fluorescence [18]. Such tags do not require oxygen, and their formation time is short since it corresponds to the protein-folding period. Moreover, the photostability of FAPs is usually higher than the stability of FPs since in the case of photobleaching, the destroyed fluorogen molecule located in the pocket can be replaced by a molecule from solution [19].

NanoLuc is one of the best-known luciferases–proteins that catalyze bioluminescence, an oxidation reaction of small molecules accompanied by the emission of light [20]. This protein has been constructed from sea shrimp *Oplophorus gracilirostris* luciferase by several rounds of mutagenesis, resulting in luminescence output and protein size optimization. Bioluminescent systems based on this protein are used for various biomedical and biological applications, including studies of protein–protein interactions, genetic regulation and cell signaling, protein stability, and molecular imaging [21]. Many such experiments also require fluorescent labeling of the luciferase, which is needed for orthogonal labeling, resulting in more precise localization. For this purpose, fusion with fluorescent proteins, as well as staining with antibodies, are used. The first approach can affect the behavior of luciferase due to the large size of the introduced label (FP), while the second one is very time-consuming and laborious.

**Scheme 1:**
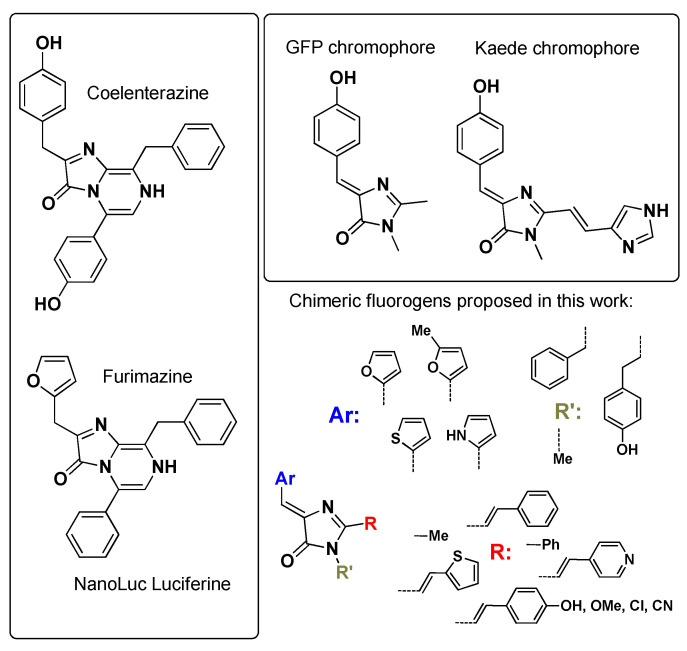
Chromophores of fluorescent proteins, luciferines and chimeric fluorogens based on their core.

Original marine luciferases utilize Coelenterazine as a substrate (Scheme 1), while NanoLuc gives the best reaction with furimazine, a furan-containing analogue (Scheme 1). Both compounds show extremely high structural similarity to arylidene-imidazolones, such as the chromophores of the green fluorescent protein (GFP) or the Kaede protein (Scheme 1).

In this work, based on this similarity, we proposed that NanoLuc can bind similar aryliden-imidazolones and act as a fluorogen-activating protein.

We synthesized a wide library of such compounds (Scheme 1) and screened them with the protein to reveal several of them that demonstrated the prominent fluorescent increase upon NanoLuc binding. We showed that these compounds in pairs with Nanoluc can be used as a genetically encoded tag capable of live-cell component fluorescent labeling.

## 2. Results

**Scheme 2:**
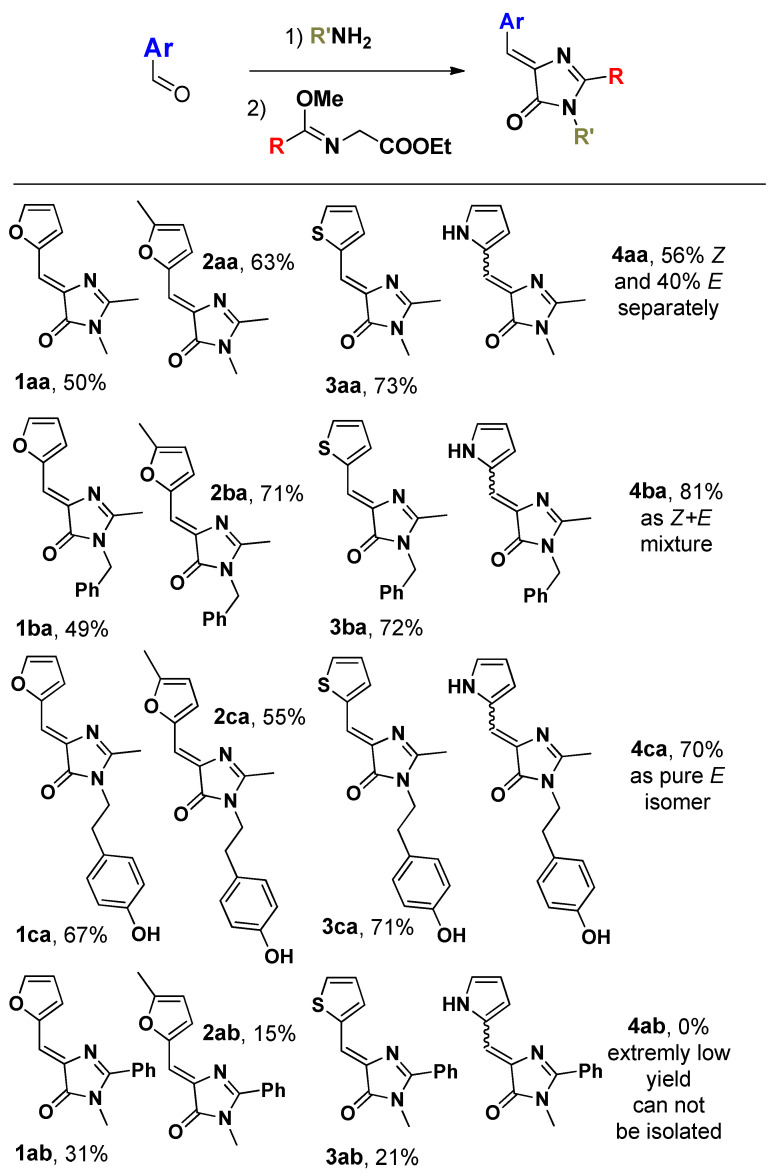
Synthesis of fluorogens based on GFP chromophore core.

**Scheme 3:**
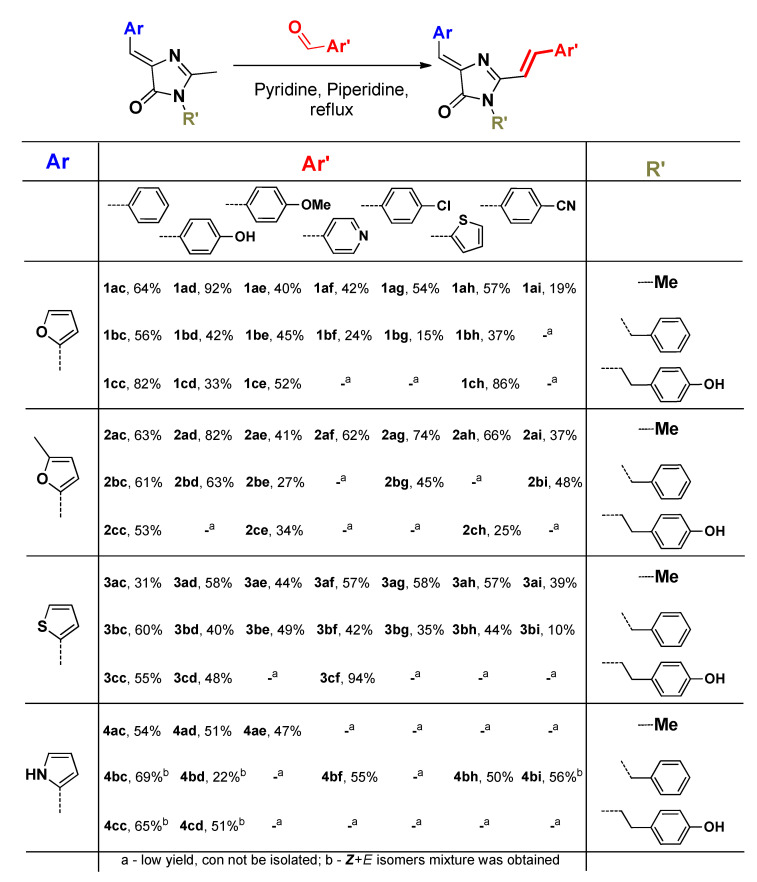
Synthesis of fluorogens based on Kaede chromophore core.

Based on the structure of GFP and Kaede chromophores, as well as the structure of NanoLuc luciferine–furimazine, we proposed a series of chimeric aryliden-imidazolone derivatives with pronounced structural similarity to the luciferine structure. The synthesis of these substances was performed using the known approaches presented in Scheme 2 and Scheme 3.

GFP derivatives were created using the reaction of imidoesters with Shiff bases [5,6]. For this reaction, we used furan, thiophene and pyrrole carbaldehydes in pairs with methylamine, benzylamine and tyramine. The introduction of such five-membered rings into the arylidene part and an additional substituent at the nitrogen atom increased the extent of the similarity of substances to furimazine. All substances were obtained with fairly good yields, with the exception of phenyl derivatives, for which the yields decreased. In particular, we failed to obtain the pyrrole derivative **4ab**. Moreover, all pyrrole derivatives were obtained as mixtures of *cis* and *trans* isomers in the arylidene part of the molecule. 

Using compounds with a methyl group, we synthesized the Kaede chromophore derivatives with an extended pi-conjugation system (Scheme 3). These fluorogens were even more similar to furimazine due to the introduction of an additional aromatic ring. Their synthesis was carried out according to previously described technology [5,6] using the condensation of the methyl group and various aromatic aldehydes. Unfortunately, we were not able to obtain a full set of derivatives since this reaction was complicated by the formation of inseparable side products and a low yield of the target compounds. The yields were especially low for pyrrole derivatives **4**. Nevertheless, we prepared almost six dozen substances, with yields ranging from 10 to 90% (Scheme 3). For such fluorogens, pyrrole derivatives were also often obtained as a mixture of isomers, which were easily transformed into each other in solution. Nevertheless, in all cases, the main isomer had a *Z* configuration in the arylidene part and an *E* configuration in the styrene part, which was confirmed by two-dimensional NMR spectroscopy (SI, Part 7, Figure S7.1).

**Scheme 4:**
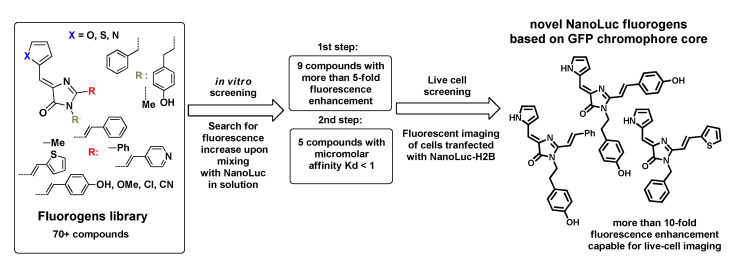
Fluorogen screening pipeline and a novel effective fluorogen for NanoLuc.

With this library of fluorogens in hand, we performed a wide screening against the purified NanoLuc protein in vitro (Scheme 4).

**Figure 1:**
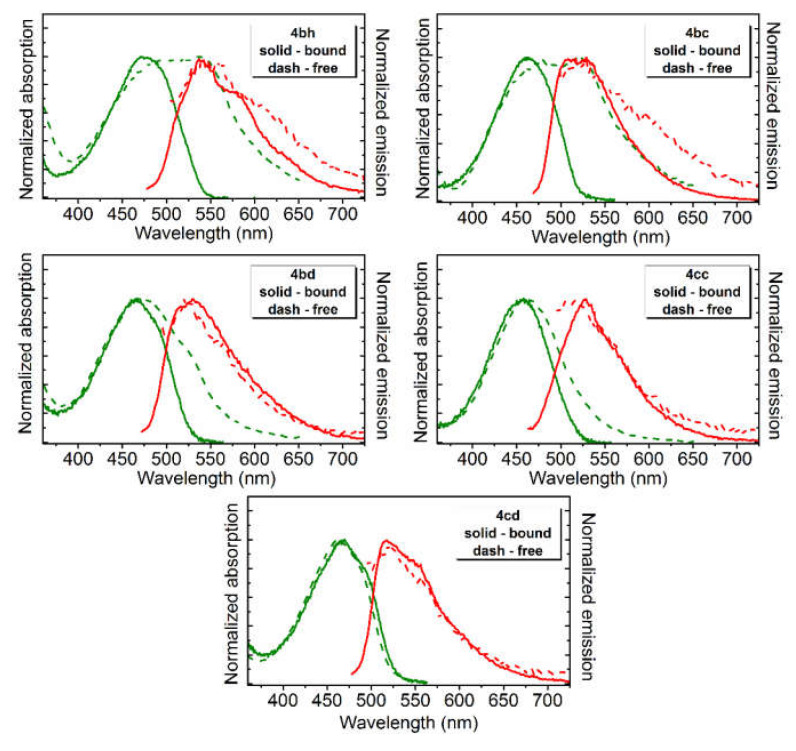
Fluorescence (red) and absorption (green) spectra of fluorogens bound with NanoLuc (solid) and free (dash) in water (PBS buffer).

First, we measured the fluorescence intensity for free fluorogen solutions and their mixture with NanoLuc (SI, Part 3, Table S3.1). As a result, we revealed a series of compounds with pronounced fluorescence increases upon formation of a complex with NanoLuc. Next, we measured the dissociation constants of formed complexes and revealed five compounds with both remarkable fluorescence increase and high affinity (K_d_ < 1 uM). For these compounds, we investigated the optical characteristics in more detail (Table 1, Figure 1, Figure S4.1, Table S4.1).

We found that fluorogen binding to the NanoLuc protein led to a noticeable constriction of the absorption spectra and an increase in the extinction coefficient (Table 1, Figure 1). This phenomenon can be explained by the mobility decrease of fluorogens in complexes and, as a consequence, a decrease in vibrational modes.

**Figure 2:**
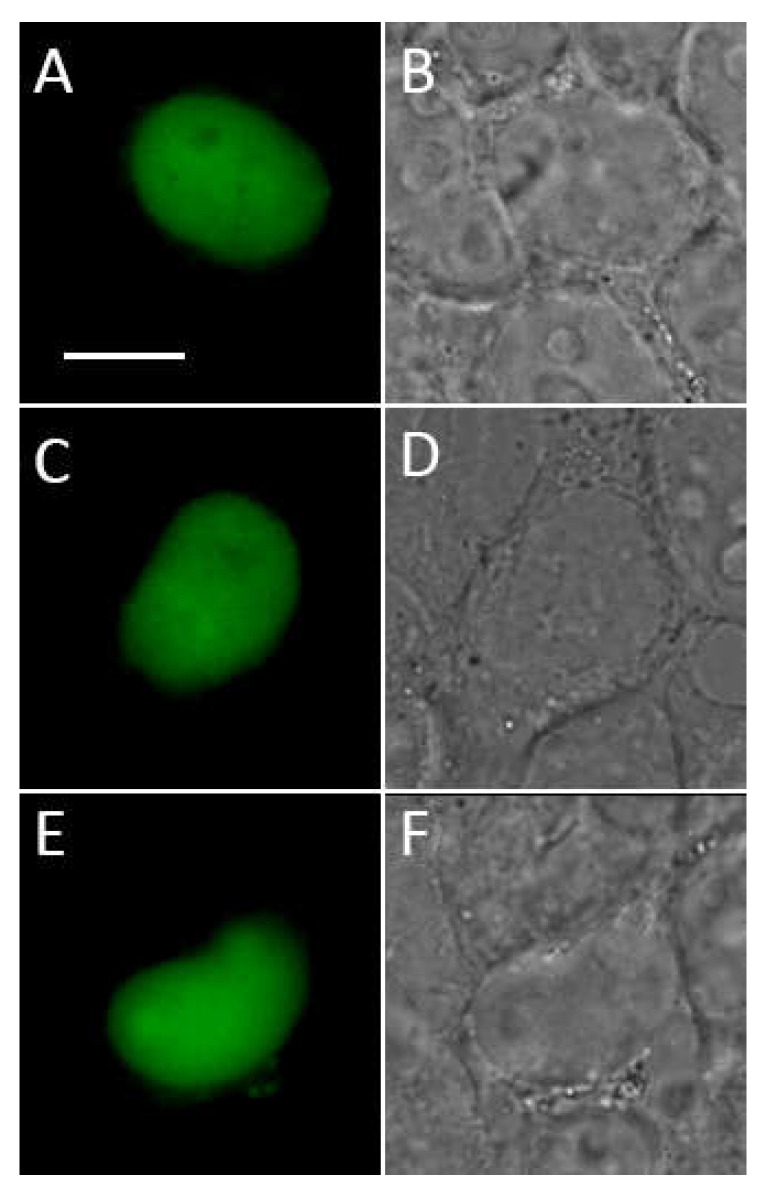
Live-cell imaging with a set of fluorogens. HEK293 cells transiently transfected with NanoLuc-H2B. Cells were imaged using GFP filter in the presence of compounds **4cc** (**A**), **4cd** (**C**) and **4bh** (**E**) at 1 μM concentration and the same cells were observed using bright field microscopy ((**B**,**D**,**F**) respectively). The scale bar is 10 μm. Background low-intensity pixels were filtered using the “background subtraction from ROI” plugin.

Finally, we performed live-cell imaging experiments using HEK293 cells transiently transfected with NanoLuc-H2B (Figure 2). H2B is one of the histone proteins that binds to DNA and participates in chromatin structuration. This protein localizes strictly in the cellular nucleus in high concentrations, which makes H2B fuses a good model for labeling experiments that have often been used in similar experiments [14,15,16,17,18,19]. Moreover, the use of such a protein fuse allows estimation of the membrane permeability of fluorogens since too lipophilic and non-permeable compounds would not stain the FAP localized in the cell nucleus.

We revealed that two of the created compounds (**4bc** and **4bd**) are indeed too lipophilic; they did not penetrate the nucleus, and their presence led to excessive off-target staining of various cellular membranes. Good and pronounced target labeling (although low-level off-target membrane labeling was still present) was achieved for three derivatives—**4cc**, **4cd** and **4bh**. These fluorogens did not accumulate in the nucleus region of NanoLuc non-expressing cells (SI, Part 5, Figure S5.1). We also found that the presence of these dyes at a concentration of 1–5 μM in the cell medium for a long time (several hours) did not lead to their death, which probably indicates their low cytotoxicity.

**Figure 3:**
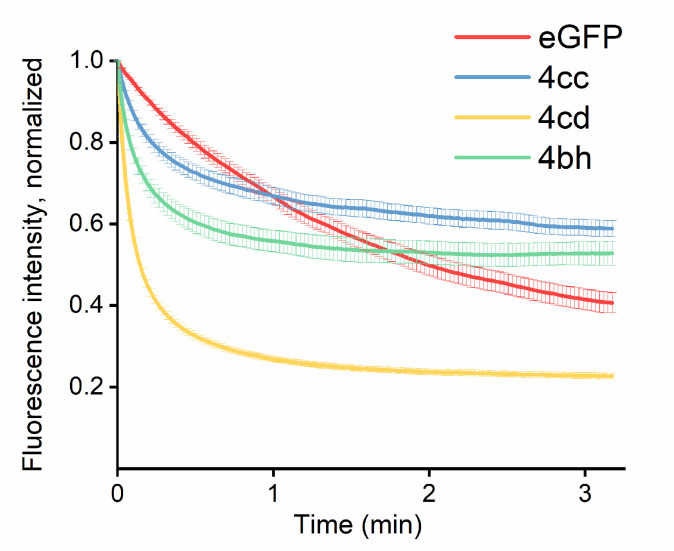
Fluorescent signal photobleaching curves of H2B-eGFP or H2B-NanoLuc (in the presence of 1 uM of **4cc**, **4cd**, and **4bh**), expressed in live HEK293 cells (±SEM, *n* = 9).

Because labeling photostability is an important characteristic in live cell imaging, we also examined the photobleaching rate of the proposed complexes compared to eGFP in the same cellular localization (Figure 3). As a result, we found that the photostability of nanoLuc staining with **4cc**, **4cd** and **4bh** was somewhat worse. However, despite the fact that in all three cases, a noticeable part of the signal was lost very quickly, the residual 20–50% of the signal persisted for a long time, during which the eGFP bleached to close values. The presence of such a residual signal indicates an exchange between the dye in the complex and in the solution, due to which equilibrium occurs and the signal reaches a plateau. Similar behavior was previously observed for other fluorogenic [11,12,13] dyes, and it is their advantage over classical fluorescent labels.

Finally, we showed that the proposed labeling technique works not only for live cells but also with fixed ones (SI, Part 5, Figure S5.2). This result showed that the NanoLuc protein does not lose the fluorogen-activating properties upon fixation and that proposed fluorogens can be possibly used for localization of this luciferase in fixed animal tissues.

## 3. Discussion

Synthesis and step-by-step screening allowed us to identify compounds not only binding to NanoLuc but also showing a noticeable increase of fluorescence upon such binding. Success was achieved by introducing various five-membered heterocyclic fragments into the arylidene moiety of the molecule. It is interesting to note that a noticeable fluorescence increase was revealed not for derivatives of furan, which is the part of furimazine but for pyrrole derivatives. This probably indicates the need for additional polar contacts for the appearance of fluorogenic properties. An increase in fluorescence was also observed for bulkier ligands containing large substituents at the nitrogen atom of the imidazolone ring. Fluorogens with these substituents are more similar to the original NanoLuc luciferins. However, both phenolic derivatives and molecules without a hydroxyl group showed activity, which indicates that no additional hydrogen bonds with proteins occur in this region, and the additional binding probably appears based on pi-stacking or hydrophobic interactions. Such a structure makes revealed fluorogens related not only to furimazine but also to the original luciferin of marine organisms–coelenterazine (Scheme 1). NanoLuc works with both of these luciferins, as well as many other substrates [20,21]. Thus, the search for other related compounds as potential fluorogens seems promising.

## 4. Materials and Methods

### 4.1. Synthesis

Commercially available reagents were used without additional purification. E. Merck Kieselgel 60 was used for column chromatography. Thin layer chromatography (TLC) was performed on silica gel 60 F254 glass-backed plates (MERCK, Rahway, NJ, USA). Visualization was performed by UV light irradiation (254 or 312 nm) and staining with KMnO4. NMR spectra were recorded on a 700 MHz Bruker Avance III NMR at 303 K, 800 MHz Bruker Avance III NMR at 333 K and Bruker Fourier 300. Chemical shifts are reported relative to residue peaks of DMSO-d6 (2.51 ppm for ^1^H and 39.5 ppm for ^13^C). Melting points were measured on an SMP 30 apparatus. High-resolution mass spectra (HRMS) were recorded on an LTQ Orbitrap Elite (ThermoScientific, Waltham, MA, USA) using electrospray ionization (ESI). The measurements were done in a positive ion mode (interface capillary voltage −5000 V) or in a negative ion mode (3500 V); the interface temperature was set at 275 °C. 

All spectral data and other characteristics of the obtained compounds are presented in Supplementary Materials Parts 6 and 8. All solid chromophores were dissolved in DMSO (Sigma Aldrich, St. Louis, MO, USA. “for molecular biology” grade. #cat D8418) in 5 mM concentration and stored in a dark place at −20 °C for no more than 3 months.

#### 4.1.1. General Procedure for the Synthesis of 5-(Z)-Arylidene-2-Methyl/Phenyl-3-R-3,5-Dihydro-4H-Imidazol-4-Ones

The corresponding aromatic aldehyde (10 mmol) was dissolved in CHCl_3_ (50 mL) and mixed with corresponding amine (10.5 mmol), pyrrolidine (7 mg, 0.1 mmol) and anhydrous Na_2_SO_4_ (10 g). The mixture was stirred for 72 h at room temperature, filtered and dried over the additional Na_2_SO_4_. The solvent was evaporated and corresponding imidate (12 mmol) was added to the residue (5–10 mL of methanol was also added if it would not mix). The mixture was stirred for 3–40 days (the progress of the reaction was monitored with TLC CHCl_3_/EtOH, *v*/*v* 100/5) at room temperature. The solvents were evaporated, and the product was purified by column chromatography (CHCl_3_-EtOH or Hexane-EtOAc).

#### 4.1.2. General Procedure for the Synthesis of 5-(Z)-Arylidene-2-(E)-Arylvinyl-3-Methyl-3,5-Dihydro-4H-Imidazol-4-Ones

A solution of 5-(Z)-arylidene-2-methyl-3-R-3,5-dihydro-4H-imidazol-4-one (1 mmol) in pyridine (5 mL) piperidine (0.02 mL) and corresponding aldehyde (5 mmol) was added. The mixture was refluxed for 5–100 h (the progress of the reaction was monitored with TLC CHCl_3_/EtOH, *v*/*v* 100/5), and the solvent was evaporated. The mixture was dissolved in EtOAc (35 mL) and washed with phosphate buffer (pH = 7.0, 3 × 15 mL), brine (3 × 25 mL) and dried over Na_2_SO_4_. The solvent was evaporated, and the product was purified by column chromatography (CHCl_3_-EtOH or Hexane-EtOAc).

#### 4.1.3. Structural Analysis of Synthesized Compounds

The structure of the obtained compounds was confirmed by two-dimensional NMR spectroscopy with ^1^H-^13^C-HSQC and ^1^H-^13^C and ^1^H–^15^N-HMBC experiments, and the configurations of the double bonds were determined on the basis of the heteronuclear J-couplings obtained from the analysis of the shape of the cross-peak lines in ^1^H–^13^C/^1^H–^15^N HSQCMBC spectra [22].

Such an analysis was performed for four relevant compounds with various arylidene moieties—**1ac**, **2ac**, **3ac** and **4ac** (see Supplementary Materials Part 7, Figure S7.1 for chemical shifts and coupling constants).

For all compounds, the vicinal J-coupling between the proton of arylidene moiety (and the carbonyl carbon was 4.0–4.4 Hz, which indicates the (Z)-configuration of the exo-double bond [23]. This is also confirmed by the large value of the J-coupling between the proton in the double bond and the nitrogen atom in the imidazolone ring (4.6–4.9 Hz), which reveals their mutual trans-configuration. In the 4th compound, the coupling constant between the same hydrogen and the nitrogen in the pyrrole ring is also large (3.7 Hz) and shows their mutual trans-orientation as well, which is also supported by the low coupling constant to the carbon (3.5 Hz) atom in the pyrrole ring. The corresponding ^1^H-^13^C constant in the other three compounds had a close value (4.1–4.6 Hz), which indicates the same trans-orientation of the heteroatoms.

The conformation of the styrene moiety was also determined based on the coupling constant values. The J-coupling between the double bond proton and the imino nitrogen of imidazolone was 3.0–3.2 Hz, which shows their mutual trans-orientation, which is supported by the absence of a cross-peak in HSQCMBC between the amino nitrogen and the same proton, suggesting zero J-coupling. Additionally, the large coupling constant between the ethylene protons (16 Hz) was indicative of the trans-configuration of the double bond [24].

### 4.2. Expression and Purification of NanoLuc

NanoLuc was expressed in *E. coli* XL-1 cells using pQE/NanoLuc plasmid (see SI, Part 1 for sequences). The transformed cells were cultured overnight at 37 °C in LB medium containing 100 μg/mL ampicillin. Overnight culture was diluted 1:200 in TB medium containing 100 μg/mL ampicillin and trace metals (1:20,000 *v*:*v*) [25], and cells were grown at 37 °C. Protein expression was induced by 0.1 mM isopropyl β-d-1-thiogalactopyranoside (IPTG) at OD600 = 0.6–0.8, and the cells were cultured for an additional 14–16 h at 25 °C. Cells were harvested by centrifugation at 5000× *g*.

The cell pellet was resuspended in IMAC buffer (20 mM Tris, pH 8.0, 2 M Urea, 10 mM Imidazole) containing 200 μM PMSF, and disrupted on ice by 15 cycles of ultrasonication (BANDELIN SONOPULS). The supernatant was clarified by centrifugation at 14,000× *g* for 60 min at 4 °C and filtration using a 0.22 μm membrane (Millipore) and loaded to a Ni Sepharose HP resin (GE) column, which was pre-equilibrated with IMAC buffer (20 mM Tris, pH 8.0, 250 mM NaCl, 10 mM bME). The impurities were removed by washing the column with an IMAC buffer. The NanoLuc was eluted by IMAC buffer with 50 mM imidazole. The fractions with target protein were analyzed by SDS-PAGE (see Supplementary Materials Part 2, Figure S2.1), pooled, dialyzed at 4 °C against the buffer (20 mM Tris, pH 7.65, 2 mM EDTA) and stored at 4 °C. The concentration of NanoLuc was determined spectroscopically at 280 nm using an extinction coefficient of 25,440 M^−1^ cm^−1^.

### 4.3. Screening In Vitro

The optical properties of chromophores were investigated using 20 µM solutions in acetonitrile. Chromophore–protein binding was tested using solutions containing 10 µM and 1 µM of NanoLuc and chromophore, respectively, in the PBS buffer (pH 7.4. #cat E404-200TABS, Amresco).

Fluorescence intensity enhancement was defined as the ratio of fluorescence intensity of the chromophore with protein solution to the fluorescence intensity of free chromophore solution recorded on a Tecan Infinite 200 Pro M Nano dual mode plate reader. The final concentrations were 1 µM for the chromophore (together in free form and in complex) and 10 for µM protein. Both solutions were excited at the wavelengths listed in Table S3.1. Due to the concentrations used, there can be non-saturated conditions. Correction to the absorbance intensity was not made.

### 4.4. Determination of Affinity Constants

The affinity constants were determined for chromophores with fluorescence intensity enhancement exceeding 5 (see Table S3.1). The affinity constants for complexes (Nanoluc–chromophore) were determined by spectrofluorometric titration of protein by chromophore solutions with various concentrations on the Tecan Infinite 200 Pro M Nano dual-mode plate reader. The protein concentration was 0.10 µM. The least squares fit (Figure S4.1 line) gave the dissociation constants K_D_ presented in Table S3.1. The titration experiments were performed at 25 °C in pH 7.4 PBS (pH 7.4. #cat E404-200TABS. Amresco, Fountain Parkway Solon, OH, USA). Fitting was performed using Origin 8.6 software.

### 4.5. Spectra of Chromophores and Their Complexes with NanoLuc

UV-VIS spectra were recorded on a Varian Cary 100 spectrophotometer. Fluorescence excitation and emission spectra were recorded on an Agilent Cary Eclipse fluorescence spectrophotometer. Spectra of free chromophores and their complexes with FAST were recorded for chromophores with K_D_ < 1 (see Table S3.1). 

The optical properties of chromophores (together in free form and in complex) were investigated using 5 µM solutions for absorption spectra registration and 0.5 µM for emission spectra registration in PBS buffer (pH 7.4, #cat E404-200TABS, Amresco). Protein was added in such an amount that, in all cases, lead α ≥ 95%. The final protein concentration for each complex was calculated using the following equation:

(1) $$\left[Pr\right]=\frac{K_{D}\times \left(\alpha \times \left[Chr\right]\right)}{\left[Chr\right]-\left(\alpha \times \left[Chr\right]\right)}+\left(\alpha \times \left[Chr\right]\right)\;$$

where *K_D_*—dissociation constant, [*Chr*]—final chromophore concentration.

### 4.6. Determination of Extinction Coefficients

The chromophores solutions were mixed with a protein solution in PBS buffer (pH 7.4, #cat E404-200TABS, Amresco). The final concentration of chromophores (together in free form and in complex) for all experiments was 5 µM. The NanoLuc protein was added in such an amount that led to almost complete transfer of the chromophore into the complex (α ≥ 95%, see Equation (1)). 

The molar extinction coefficient was calculated using the formula:

(2) $$\varepsilon =\frac{A}{cl}\;$$

where *A* is the absorbance intensity at maxima, *c* is the molar concentration of complexes, and *l* is the pathlength.

### 4.7. Fluorescence Quantum Yield Determination

Fluorescence quantum yields for chromophores and their complexes with Nanoluc were calculated according to the procedure described in the literature [26], with the use of Coumarine 153 as a standard. The chromophores solutions were mixed with a protein solution in PBS buffer (pH 7.4, #cat E404-200TABS, Amresco). The final concentration of chromophores (together in free form and in complex) was 5 µM for absorption spectra registration and 0.5 µM for emission spectra registration. Protein was added in such an amount that in all cases lead α ≥ 95% (see Equation (1)).

The quantum yield was calculated using the formula:

(3) $$\Phi_{x}=\Phi_{\mathrm{st}}\times \frac{F_{x}}{F_{\mathrm{st}}}\times \frac{f_{\mathrm{st}}}{f_{x}}\times \frac{n_{x}^{2}}{n_{\mathrm{st}}^{2}}\;$$

where F is the area under the emission peak, f is the absorption factor (see below), n is the refractive index of the solvent, Φ is the quantum yield, the subscript x corresponds to the novel compounds, and the subscript st is for the standard.

(4) $$f=1-10^{-A}$$

where A is absorbance at the excitation wavelength.

### 4.8. Fluorescent Imaging in Living Cells

Targeted to the cell nucleus, NanoLuc fused with H2B histone protein was cloned by Golden Gate assembly, following the MoClo syntax. The coding sequence of NanoLuc in Level 0 plasmid was ordered from Cloning Facility (Moscow, Russia). The construct was put under a CMV promoter and possessed an SV40 poly(A) sequence. BpiI (BbsI), Eco31I (BsaI) restriction endonucleases (Thermo Scientific, Waltham, MA, USA) and T4 DNA ligase (Evrogen LK001) were used for the cloning procedure. Plasmid coding of the H2B-eGFP construct under the CMV-promoter was available in-house [16]. HEK293 was obtained from the Institute of Bioorganic Chemistry collection of cell lines. Cells were plated onto a 35 mm glass-bottomed culture dish (SPL Life Sciences, Gyeonggi-do, Republic of Korea) and grown in the DMEM medium (PanEco, Moscow, Russia) with 10% (*v*/*v*) FBS (fetal bovine serum; Sigma, St. Louis, MO, USA) containing 50 U/mL penicillin and 50 μg/mL streptomycin (PanEco) at 37 °C and 5% CO_2_ for 24 h before transfection.

Transfection was performed using polyethylenimine, PEI (#23966-1, Polysciences, Warrington, PA, USA). The cell culture media was changed for Opti-MEM one hour before the transfection procedure. Six microliters of PEI were mixed with 250 μL of Opti-MEM per dish. In a separate tube, 2 μg of plasmid DNA was mixed with 250 μL of Oti-MEM. The PEI-containing mixture was incubated for 5 min, following which PEI- and DNA-containing media were mixed and incubated for 20 min. The PEI–DNA mixture was added to cells dropwise, cells were incubated for 3 h, and transfection media was replaced with DMEM complete.

The imaging experiment was performed 48 h after transfection using a BZ-9000 inverted fluorescence microscope (Keyence, Osaka, Japan) in 2 mL of Hanks’ Balanced Salt Solution (PanEco) with 10 mM HEPES (Sigma, Darmstadt, Germany), pH 7.3, and 1 μM chromophore (from 10 mM DMSO stock solution) at room temperature. Imaging of fixed cells was carried out after a 10 min fixation–permeabilization procedure with 1 mL ice-cold methanol. The imaging was carried out with a 60 × PlanApo 1.40 NA oil objective (Nikon, Melville, NY, USA), GFP-B filter (Keyence, Ex. 470/40 nm, DM 495 nm, BA 535/50 nm) were used. The resulting images were processed using Fiji software.

The photobleaching experiment was performed 48 h after transfection using a Leica confocal microscope DMIRE2 TCS SP2 (Leica, Wetzlar, Germany) with HCX PL APO lbd.BL 63.0 × 1.40 oil immersion objective. Live HEK293 cells transiently transfected with H2B-NanoLuc (in the presence of 1 µM **4cc**, **4cd**, and **4bh**) or H2B-eGFP were illuminated by a 488 nm laser. The resulting images were processed using Fiji software.

## 5. Conclusions

In this work, we showed that the well-known NanoLuc luciferase can act as a fluorogen-activating protein for various arylidene-imidazolones structurally similar to the Kaede protein chromophore. For this purpose, we synthesized a library containing several dozen novel fluorogens similar in structure to furimazine, the bioluminescent substrate of this protein and showed that some of them can be used for genetically encoded fluorescent labeling together with NanoLuc.

The creation of new pairs of fluorogens and proteins that activate them is, in itself, an important task in the rapidly developing field of living systems fluorescent labeling. However, no less importantly, our work allows us to carry out orthogonal labeling and analysis of the NanoLuc protein not with the help of bioluminescent methods but with the help of fluorescence microscopy. We have shown that this protein can be simultaneously used not only for bioluminescent labeling but also for fluorescent labeling. It avoids the need to introduce additional fusions with fluorescent proteins into NanoLuc when such labeling is necessary. Additionally, it may be used as a fast and cheap alternative to immunocyto- or histochemistry for the determination of precise cellular localization of luciferase (e.g., on fixed animal tissues after in vivo bioluminescence imaging). Even more effective systems for such double labeling can be created in the future with further improvement of the created substances, as well as directed protein mutagenesis.

Moreover, the present study demonstrates the overall success of the proposed approach for searching for fluorogens suitable for labeling of any target protein. The simplicity of the syntheses of various arylidene-imidazoline libraries, as well as the simplicity of the proposed screening, make it possible to claim that this approach may be effective in the search for new fluorogens suitable for target protein labeling.

## Figures and Tables

**Table 1:** Optical properties of chromophores and their complexes with NanoLuc.

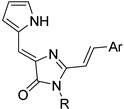	R	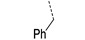			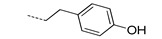	
	Ar	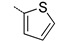	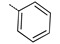	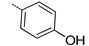	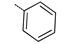	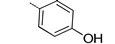
		4bh	4bc	4bd	4cc	4cd
FQY ^a^	Free ^b^	0.40	0.38	0.24	0.16	0.13
	Bound ^c^	3.39	3.75	3.46	2.51	4.29
Enhancement (=FQY_bound_/FQY_free_)		8.4	9.8	14.5	16.6	33.7
Absorption ^d^	Free ^b^	480–535 ^g^	475–525 ^g^	470	459	462
	Bound ^c^	474	460	467	458	465
Emisson ^d^	Free ^b^	540	515	520	520	510
	Bound ^c^	540	513	525	522	520
EC ^e^	Free ^b^	13,000	11,000	16,000	19,000	27,000
	Bound ^c^	34,000	31,000	23,000	24,000	24,000
*K*_d_^f^		0.32 ±0.02	0.59 ±0.04	0.50 ±0.03	0.55 ±0.04	0.48 ±0.02

^a^—fluorescence quantum yield in %; ^b^—characteristic of fluorogen in solution without NanoLuc in water (PBS buffer); ^c^—characteristic of fluorogen in complex with NanoLuc; ^d^—maxima position in nm; ^e^—extinction coefficient in M^−1^ × cm^−1^ × 10^−3^; ^f^—dissocation constant in μM; ^g^—broad spectra, see Figure 1.

## Data Availability

Data is contained within the article or Supplementary Materials.

## Supplementary Materials

The following supporting information can be downloaded at: https://www.mdpi.com/article/10.3390/ijms24097958/s1.
