# Supplementary material for: NanoLuc Luciferase as a Fluorogen-Activating Protein for GFP Chromophore Based Fluorogens
Source: Int J Mol Sci. 2023 Apr 27;24(9):7958. doi: 10.3390/ijms24097958 (PMC10178166; doi:10.3390/ijms24097958)
Supplement: Supplementary file 1 [file ijms-24-07958-s001.zip › ijms-2337815-supplementary.pdf]

Contents

**1. 1. Sequences**	**S2**
**2. Expression and purification of NanoLuc**	**S3**
**3. Screening in vitro**	**S4**
**4. Determination of affinity constants**	**S16**
**5. Fluorescent microscopy**	**S19**
**6. Spectral characteristics**	**S21**
**7. Structure confirmation**	**S61**
**8. Copies of 1H and 13C NMR spectra**	**S62**

1. Sequences

Coding sequences for NanoLuc

ATGAGAGGATCGCATCACCATCACCATCACGGATCCATGGTCTTCACACTCGAAGATTTCGTTGGGGACTGGCGACAGACAGCCGGCTACAACCTGGACCAAGTCCTTGAACAGGGAGGTGTGTCCAGTTTGTTTCAGAATCTCGGGGTGTCCGTAACTCCGATCCAAAGGATTGTCCTGAGCGGTGAAAATGGGCTGAAGATCGACATCCATGTCATCATCCCGTATGAAGGTCTGAGCGGCGACCAAATGGGCCAGATCGAAAAAATTTTTAAGGTGGTGTACCCTGTGGATGATCATCACTTTAAGGTGATCCTGCACTATGGCACACTGGTAATCGACGGGGTTACGCCGAACATGATCGACTATTTCGGACGGCCGTATGAAGGCATCGCCGTGTTCGACGGCAAAAAGATCACTGTAACAGGGACCCTGTGGAACGGCAACAAAATTATCGACGAGCGCCTGATCAACCCCGACGGCTCCCTGCTGTTCCGAGTAACCATCAACGGAGTGACCGGCTGGCGGCTGTGCGAACGCATTCTGGCGTAA

NanoLuc protein sequence

MRGSHHHHHHGSMVFTLEDFVGDWRQTAGYNLDQVLEQGGVSSLFQNLGVSVTPIQRIVLSGENGLKIDIHVIIPYEGLSGDQMGQIEKIFKVVYPVDDHHFKVILHYGTLVIDGVTPNMIDYFGRPYEGIAVFDGKKITVTGTLWNGNKIIDERLINPDGSLLFRVTINGVTGWRLCERILA

2. Expression and purification of NanoLuc

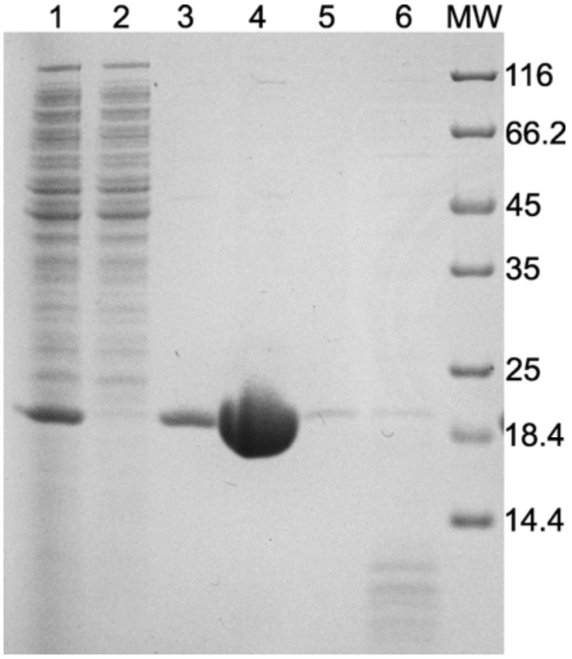

Figure S2.1. Purification of NanoLuc by immobilized metal affinity chromatography. (1) cell lysate, (2) flow-through, (3-5) elution fractions at 50 mM Imidazole, (6) elution at 500 mM Imidazole, MW - protein marker.

3. Screening *in vitro*

Table S3.1. Optical properties of chromophores and the results of interaction with Nanoluc

Cmpd	Stucture	Abs[a]	Enhancement					
			380	430	480	530	580	630
**1aa**	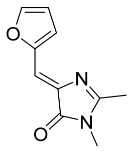	372	1.6	1.2	1.1	1.1	1.3	1.0
**1ab**	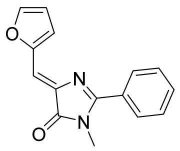	394	1.3	1.2	1.1	1.1	1.2	1.1
**1ac**	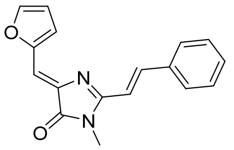	429	1.5	1.6	1.6	1.1	1.1	0.9
**1ad**	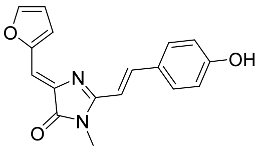	432	1.5	1.7	1.9	1.4	1.1	0.9
**1ae**	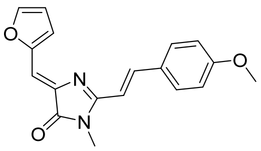	433	1.4	1.5	1.5	1.1	1.0	1.7
**1af**	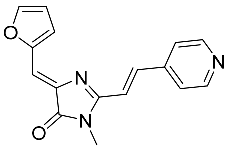	432	1.3	1.3	1.3	1.1	0.9	1.0
**1ag**	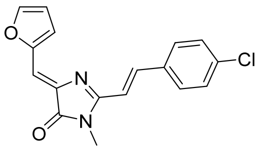	433	1.4	1.4	1.4	1.1	0.9	0.9
**1ah**	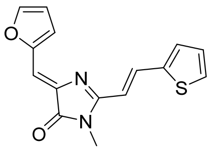	436	1.4	1.3	1.3	1.2	1.0	1.8
**1ai**	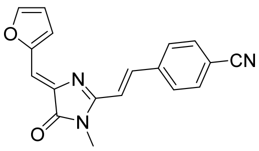	438	1.4	1.2	1.3	1.2	0.9	1.2

**2aa**	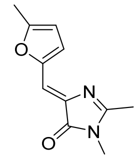	381	1.3	1.2	1.1	1.1	1.0	0.6
**2ab**	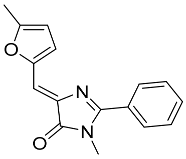	407	1.4	1.2	1.1	1.0	1.0	0.9
**2ac**	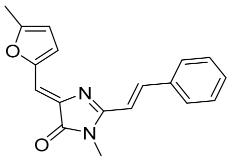	442	1.5	1.8	1.9	1.2	1.0	1.0
**2ad**	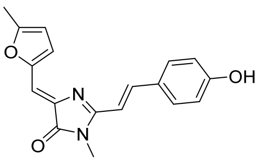	445	1.4	1.3	1.3	1.2	1.0	1.2
**2ae**	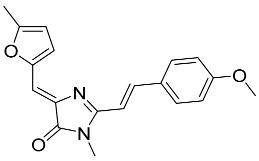	445	1.4	1.6	1.6	1.2	0.9	1.3
**2af**	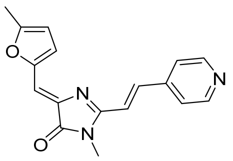	446	1.4	1.3	1.3	1.1	1.1	1.3
**2ag**	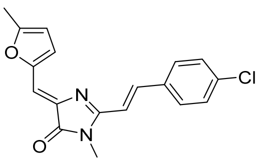	445	1.5	1.6	1.6	1.1	0.9	1.1
**2ah**	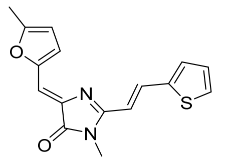	452	1.4	1.3	1.5	1.3	1.1	0.9
**2ai**	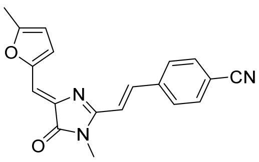	451	1.4	1.4	1.4	1.2	1.1	1.1

**3aa**	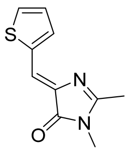	381	1.3	1.2	1.1	1.0	1.1	1.0
**3ab**	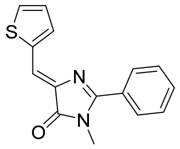	395	1.4	1.2	1.1	1.0	1.0	0.9
**3ac**	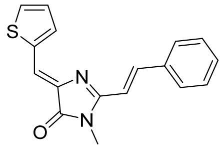	429	1.9	2.7	2.5	1.1	1.0	1.0
**3ad**	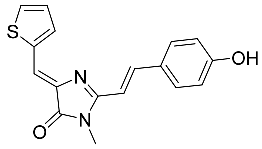	435	1.5	2.1	2.8	2.1	1.0	1.1
**3ae**	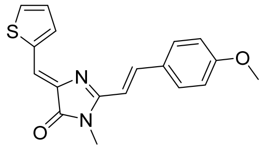	434	1.5	1.8	1.7	1.1	1.0	2.2
**3af**	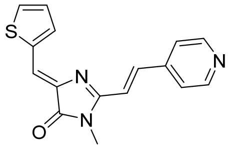	432	1.4	1.7	1.7	1.1	1.2	1.7
**3ag**	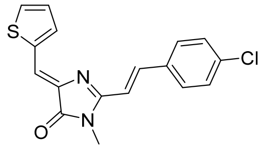	432	1.5	1.6	1.6	1.1	1.0	0.9
**3ah**	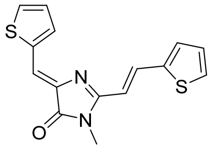	437	1.4	1.5	1.8	1.3	1.1	1.1
**3ai**	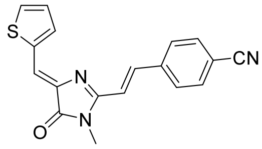	437	1.4	1.7	1.7	1.1	1.1	1.2

**4aa**	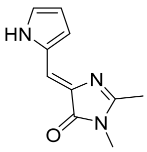	386	1.3	1.1	1.1	1.0	1.0	1.9
**4ac**	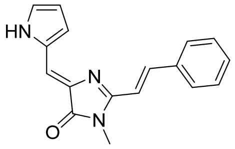	452	1.6	3.4	3.6	1.3	1.1	1.0
**4ad**	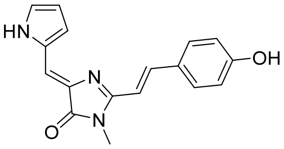	452	2.1	8.1	10.2	2.8	1.3	0.9
**4ae**	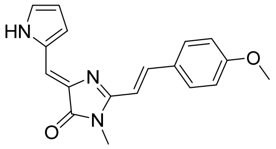	454	2.3	9.3	9.1	1.5	1.1	1.4

**1ba**	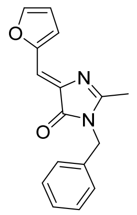	372	1.4	1.2	1.1	1.1	0.9	1.0
**1bc**	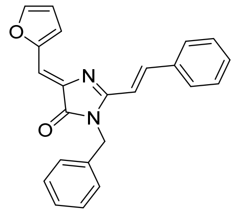	430	1.7	2.8	3.0	1.1	1.2	1.0
**1bd**	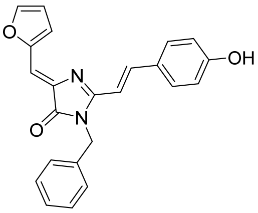	438	1.9	3.1	4.9	4.1	1.2	1.3
**1be**	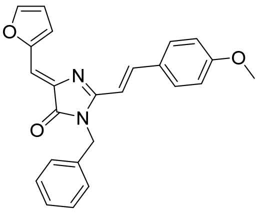	434	1.4	1.5	1.3	1.1	1.0	1.0
**1bf**	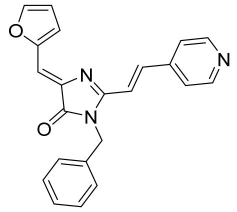	433	1.7	2.7	2.6	1.1	1.0	0.7
**1bg**	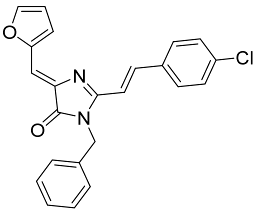	433	1.4	1.5	1.3	1.0	1.0	1.0
**1bh**	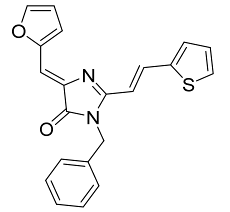	439	1. 2	1.8	2.8	1.8	1.2	0.9

**2ba**	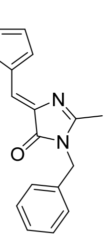	382	1.4	1.2	1.1	1.1	0.9	0.9
**2bc**	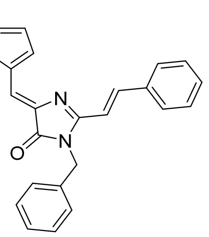	443	1.5	2.6	2.8	1.1	1.0	0.9
**2bd**	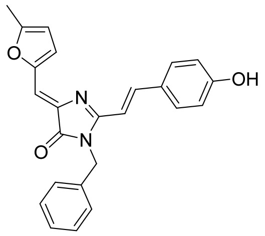	448	1.9	3.0	4.7	3.4	1.0	1.3
**2be**	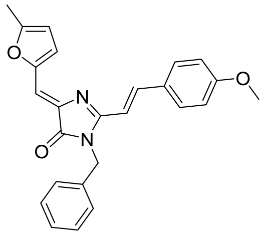	446	1.5	1.5	1.4	1.0	1.0	1.0
**2bg**	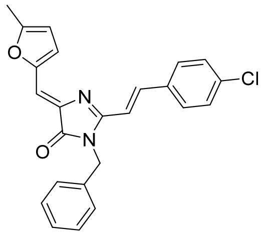	446	1.3	1.4	1.2	1.0	1.0	1.1
**2bi**	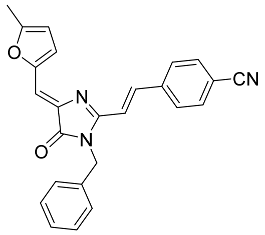	453	1.7	2.8	2.5	1.2	0.9	1.2

**3ba**	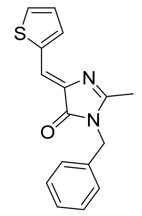	372	1.4	1.2	1.1	1.2	0.9	1.2
**3bc**	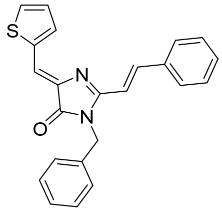	430	1.4	1.6	1.5	1.0	1.0	1.8
**3bd**	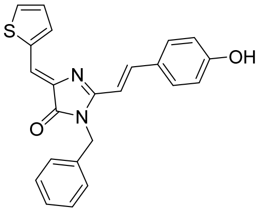	437	1.6	2.4	3.5	2.5	1.2	2.8
**3be**	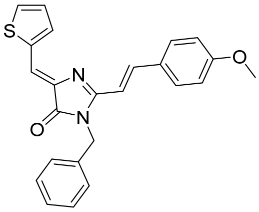	437	1.3	1.4	1.3	1.1	1.0	1.2
**3bf**	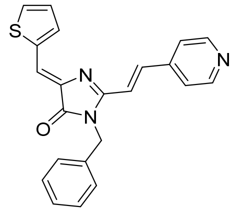	433	2.3	4.3	3.0	1.2	1.0	1.0
**3bg**	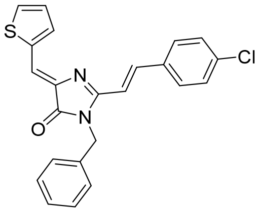	433	1.4	1.7	1.4	1.1	1.3	1.8
**3bh**	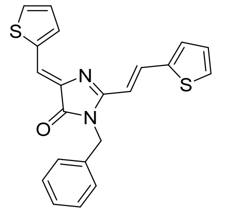	441	1.4	1.9	3.0	1.4	1.4	1.1
**3bi**	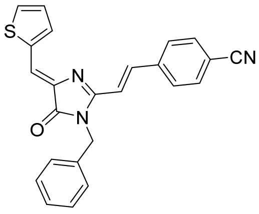	438	1.6	2.8	2.4	1.2	1.8	1.3

**4ba**	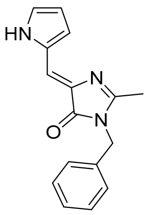	390	1.4	1.2	1.1	1.2	1.2	1.0
**4bc**	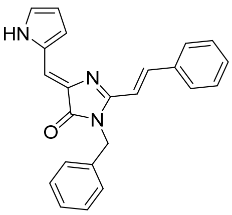	453	1.8	4.6	5.8	1.7	1.5	1.2
**4bd**	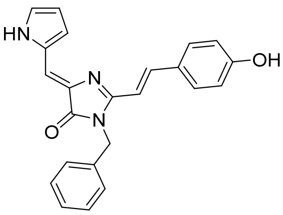	456	1.9	3.7	5.7	5.0	2.0	1.3
**4bf**	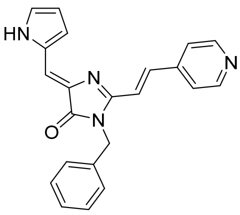	461	1.8	4.6	5.8	2.7	1.7	1.0
**4bh**	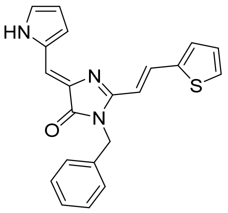	461	2.0	4.5	7.9	6.7	1.0	1.2
**4bi**	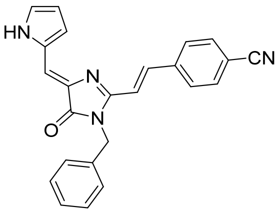	464	2.0	3.5	4.8	3.3	2.5	0.9

**1ca**	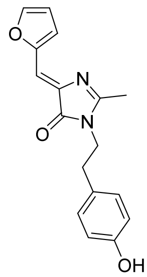	373	1.4	1.2	1.1	1.1	1.1	1.1
**1cc**	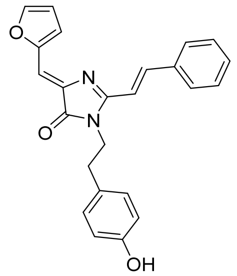	430	2.0	3.1	2.6	1.1	1.3	1.5
**1cd**	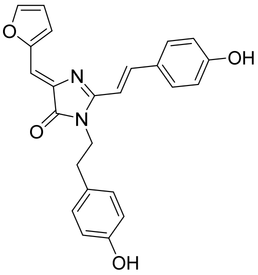	437	1.5	1.8	2.3	1.2	0.9	1.0
**1ce**	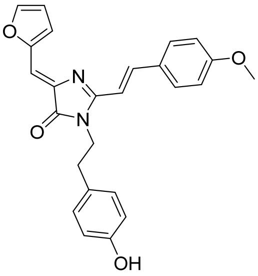	436	1.6	2.1	2.6	1.2	1.3	1.1
**1ch**	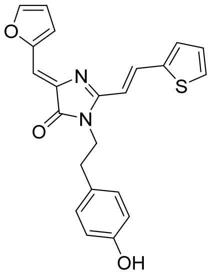	439	1.4	1.6	1.7	1.2	1.0	0.9

**2ca**	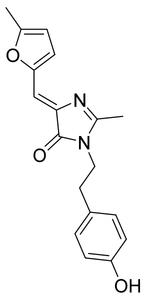	383	1.4	1.2	1.1	1.1	1.4	1.1
**2cc**	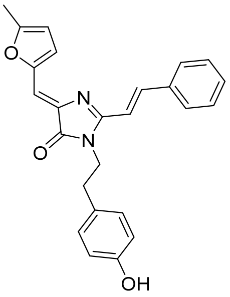	443	2.1	4.1	2.9	1.2	1.1	1.0
**2ce**	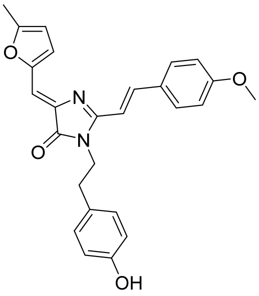	446	1.7	2.5	3.0	1.2	1.0	1.1
**2ch**	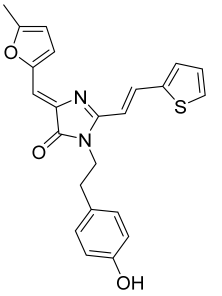	452	1.4	1.5	1.7	1.2	1.0	0.9

**3ca**	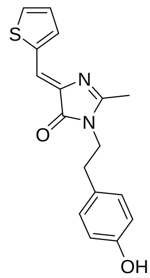	374	1.4	1.2	1.2	1.1	1.0	1.1
**3cc**	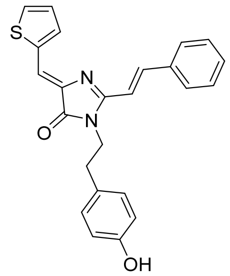	430	1.9	2.6	1.6	1.2	1.4	0.9
**3cd**	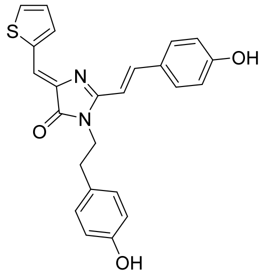	436	1.4	1.7	1.9	1.4	1.4	1.3
**3cf**	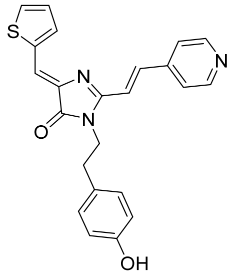	433	1.5	2.1	2.0	1.3	1.5	0.9

**4ca**	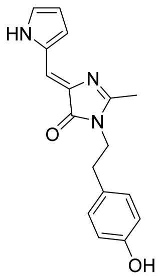	397	1.4	1.2	1.1	1.1	1.2	1.3
**4cc**	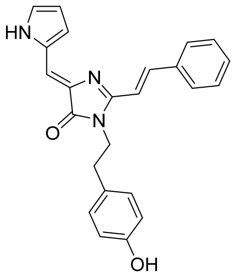	452	1.7	3.7	5.7	2.0	1.1	1.4
**4cd**	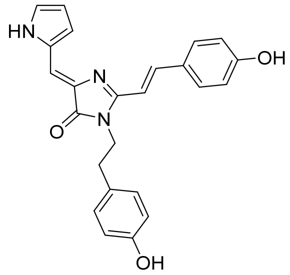	454	1.6	3.4	6.8	3.7	1.4	0.9

a – maxima position in nm;

4. Determination of affinity constants

Table S4.1. Dissociation constants values of complexes [Nanoluc-chromophore]

**Chromophore**	**Structure**	**$K_{D.}$ µM**
**4ad**	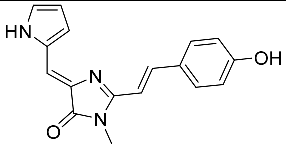	>5
**4ae**	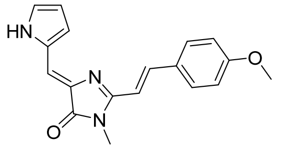	1.81±0.07
**4bc**	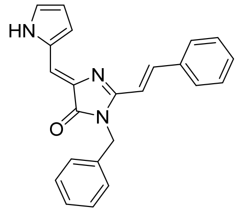	0.59±0.04
**4bd**	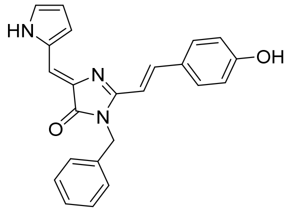	0.50±0.03
**4bf**	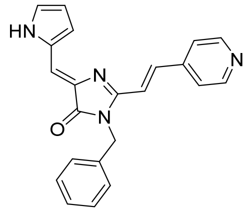	>5
**4bh**	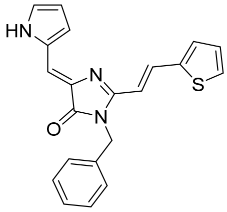	0.32±0.02
**4cc**	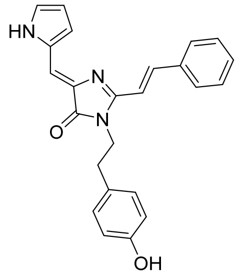	0.55±0.04

**4cd**	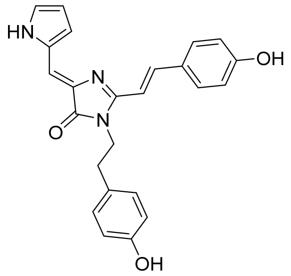	0.48±0.02

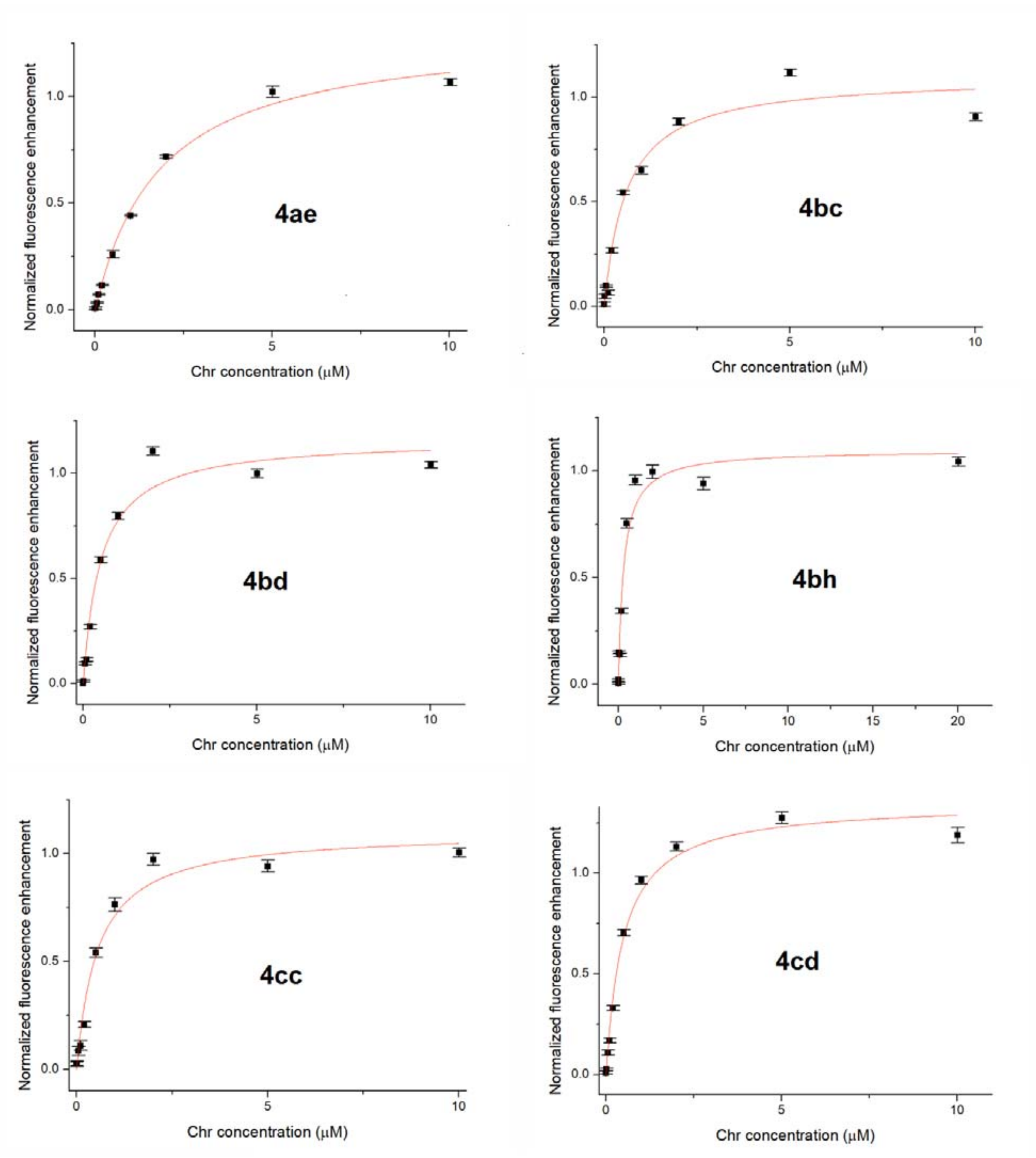

Fig S4.1. Titration curves.

5. Fluorescent microscopy

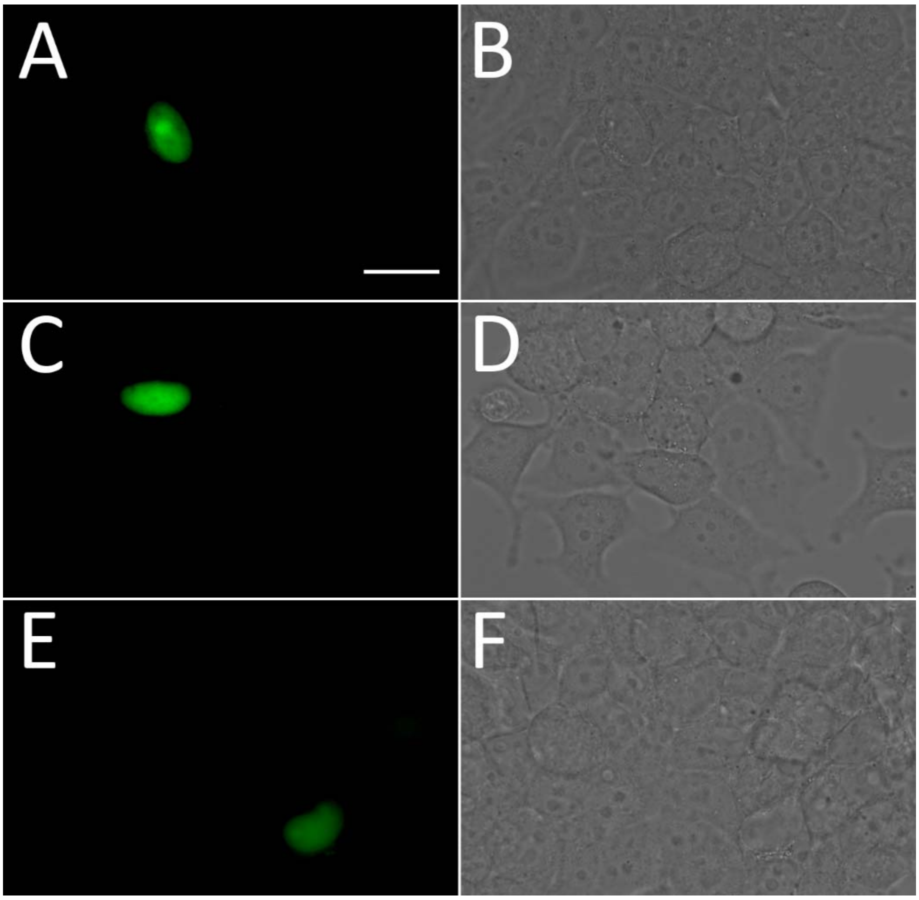

Fig. S 5.1. Live-cell imaging of sparsely expressing NanoLuc HEK293 cells with **4cc**, **4cd**, and **4bh** fluorogens at 1 μM concentration. HEK293 cells were transiently transfected with NanoLuc-H2B and were imaged using GFP filter and bright field microscopy (B, D, F respectively) in the presence of fluorogens (A, C, E). Only NanoLuc expressing cells show fluorescent signal in the nuclei. Scale bar is 20 μm.

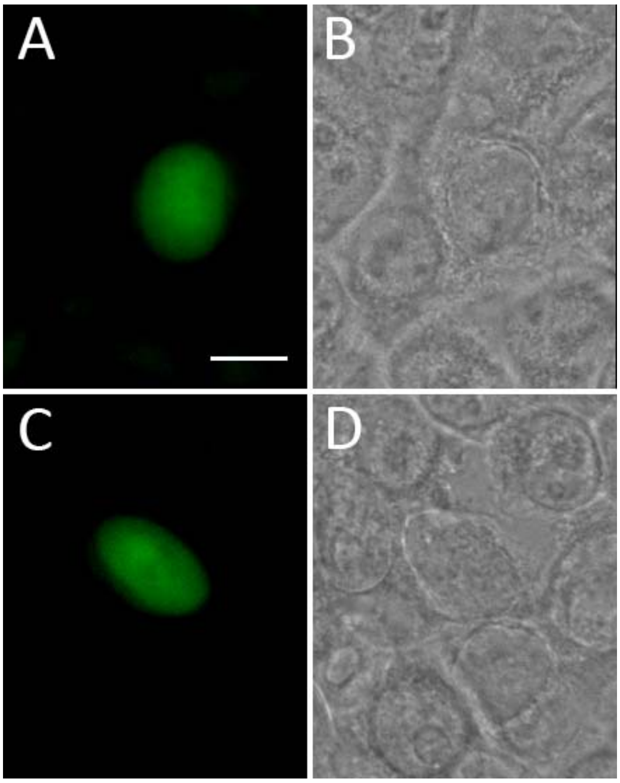

Fig. S 5.2. Imaging of fixated with methanol NanoLuc-H2B expressing HEK293 cells with **4cc** (A, B), **4cd** (C, D) fluorogens at 1 μM concentration. Widefield fluorescent microscopy with GFP filter (A, C) and bright field microscopy (B, D) were used. Scale bar is 10 μm.

6. Spectral characteristics

5.1 5-(Z)-arylidene-2-methyl/phenyl-3-R-3,5-dihydro-4H-imidazol-4-ones

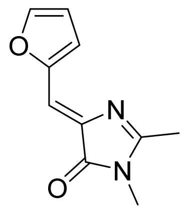

(*Z*)-5-(furan-2-ylmethylene)-2,3-dimethyl-3,5-dihydro-4*H*-imidazol-4-one (1aa)

Dark-yellow solid (0.95 g, 50%); mp 119-121 ºC; ^{1}H NMR (300 MHz, DMSO-d_6) δ ppm 7.92 (d, *J*=1.1 Hz, 1 H), 7.32 (d, *J*=3.4 Hz, 1 H), 6.81 (s, 1 H), 6.70 (dd, *J*=3.0, 1.6 Hz, 1 H), 3.07 (s, 3 H), 2.33 (s, 3 H); ^{13}C NMR (75 MHz, DMSO-d_6) δ ppm 169.3, 163.8, 150.3, 146.2, 136.4, 117.8, 113.5, 111.9, 26.3, 15.4; HRMS (ESI) m/z: 191.0815 found (calcd for $C_{10}H_{11}N_2O_2^+$, $[M+H]^+$ 191.0815).

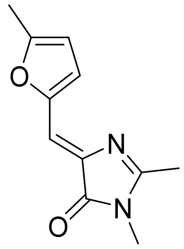

(*Z*)-5-((5-methylfuran-2-yl)methylene)-2,3-dimethyl-3,5-dihydro-4*H*-imidazol-4-one (2aa)

Orange solid (1.29 g, 63%); mp 132-134 ºC; ^{1}H NMR (300 MHz, DMSO-d_6) δ ppm 7.26 (d, *J*=3.3 Hz, 1 H), 6.73 (s, 1 H), 6.36 (d, *J*=3.3 Hz, 1 H), 3.07 (s, 3 H), 2.35 (s, 3 H), 2.31 (s, 3 H); ^{13}C NMR (75 MHz, DMSO-d_6) δ ppm 169.3, 162.9, 155.9, 149.1, 135.2, 119.6, 112.1, 110.4, 26.3, 15.3, 13.7; HRMS (ESI) m/z: 205.0972 found (calcd for $C_{11}H_{13}N_2O_2^+$, $[M+H]^+$ 205.0972).

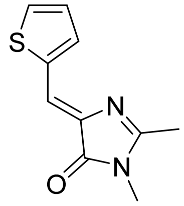

(*Z*)-5-(thiophen-2-ylmethylene)-2,3-dimethyl-3,5-dihydro-4*H*-imidazol-4-one (3aa)

Yellow solid (1.50 g, 73%); ^{1}H NMR (300 MHz, DMSO-d_6) δ ppm 7.85 (d, *J*=5.1 Hz, 1 H), 7.66 (d, *J*=3.4 Hz, 1 H), 7.32 (s, 1 H), 7.14 (dd, *J*=5.0, 3.6 Hz, 1 H), 3.08 (s, 3 H), 2.33 (s, 3 H).[1]

[1] Zaitseva S.O., Golodukhina S.V., Baleeva N.S., Levina E.A., Smirnov A.Yu., Zagudaylova M.B., Baranov M.S. Chem. Select, **2018**, 3 (30), 8593-8596.

5-((1*H*-pyrrol-2-yl)methylene)-2,3-dimethyl-3,5-dihydro-4*H*-imidazol-4-one (4aa)

It was obtained as a mixture of two isomers. The mixture was successfully separated by column chromatography:

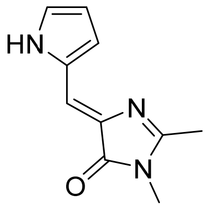

(*Z*)-5-((1*H*-pyrrol-2-yl)methylene)-2,3-dimethyl-3,5-dihydro-4*H*-imidazol-4-one (4aa-Z)

Yellow solid (1.05 g, 56%); mp 150-152 ºC; ^{1}H NMR (700 MHz, DMSO-d_6) δ ppm 11.23 (br. s., 1 H), 7.15 (d, *J*=1.2 Hz, 1 H), 6.88 (s, 1 H), 6.81 (br. s., 1 H), 6.22 (ddd, *J*=3.5, 2.3, 2.2 Hz, 1 H), 3.08 (s, 3 H), 2.33 (s, 3 H); ^{13}C NMR (75 MHz, DMSO-d_6) δ ppm 168.9, 160.0, 133.0, 128.1, 125.4, 117.9, 115.9, 110.5, 26.1, 15.1; HRMS (ESI) m/z: 190.0975 found (calcd for $C_{10}H_{12}N_3O^+$, $[M+H]^+$ 190.0975).

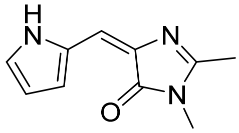

(*E*)-5-((1*H*-pyrrol-2-yl)methylene)-2,3-dimethyl-3,5-dihydro-4*H*-imidazol-4-one (4aa-E)

Yellow solid (0.76 g, 40%); mp 141-143 ºC; ^{1}H NMR (700 MHz, DMSO-d_6) δ ppm 12.89 (br. s., 1 H), 7.30 (d, *J*=1.1 Hz, 1 H), 7.17 (s, 1 H), 6.82 (br. s., 1 H), 6.32 (ddd, *J*=3.6, 2.3, 2.1 Hz, 1 H), 3.16 (s, 3 H), 2.26 (s, 3 H); ^{13}C NMR (176 MHz, DMSO-d_6) δ ppm 168.2, 155.9, 132.7, 128.3, 125.2, 124.7, 119.9, 111.4, 26.5, 14.6; HRMS (ESI) m/z: 190.0975 found (calcd for $C_{10}H_{12}N_3O^+$, $[M+H]^+$ 190.0975).

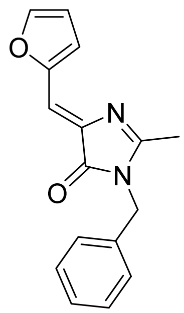

(*Z*)-5-(furan-2-ylmethylene)-3-benzyl-2-methyl-3,5-dihydro-4*H*-imidazol-4-one (1ba)

Yellow solid (1.31 g, 49%); mp 96-98 ºC; ^{1}H NMR (700 MHz, DMSO-d_6) δ ppm 7.94 (d, *J*=1.3 Hz, 1 H), 7.34 - 7.38 (m, 3 H), 7.29 (t, J=7.4 Hz, 1 H), 7.24 (d, *J*=7.2 Hz, 2 H), 6.90 (s, 1 H), 6.72 (dd, *J*=3.2, 1.5 Hz, 1 H), 4.82 (s, 2 H), 2.25 (s, 3 H); ^{13}C NMR (75 MHz, DMSO-d_6) δ ppm 169.3, 162.9, 150.3, 146.5, 136.7, 135.7, 128.8, 127.5, 126.9, 118.3, 113.5, 112.8, 43.0, 15.6; HRMS (ESI) m/z: 267.1126 found (calcd for $C_{16}H_{15}N_2O_2^+$, $[M+H]^+$ 267.1128).

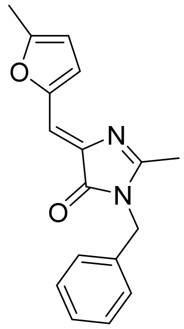

(*Z*)-5-((5-methylfuran-2-yl)methylene)-3-benzyl-2-methyl-3,5-dihydro-4*H*-imidazol-4-one (2ba)

Dark-yellow solid (1.99 g, 71%); mp 98-100 ºC; ^{1}H NMR (700 MHz, DMSO-d_6) δ ppm 7.36 (t, J=7.6 Hz, 2 H), 7.27 - 7.31 (m, 2 H), 7.23 (d, *J*=7.2 Hz, 2 H), 6.82 (s, 1 H), 6.37 (d, *J*=3.4 Hz, 1 H), 4.81 (s, 2 H), 2.37 (s, 3 H), 2.23 (s, 3 H); ^{13}C NMR (75 MHz, DMSO-d_6) δ ppm 169.3, 161.9, 156.3, 149.1, 136.8, 134.5, 128.8, 127.5, 126.9, 120.1, 112.9, 110.5, 43.0, 15.6, 13.7; HRMS (ESI) m/z: 281.1282 found (calcd for $C_{17}H_{17}N_2O_2^+$, $[M+H]^+$ 281.1285).

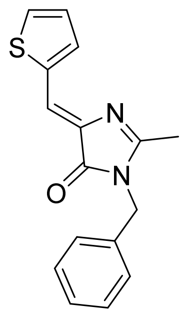

(*Z*)-5-(thiophen-2-ylmethylene)-3-benzyl-2-methyl-3,5-dihydro-4*H*-imidazol-4-one (3ba)

Yellow solid (2.03 g, 72%); mp 101-103 ºC; ^{1}H NMR (700 MHz, DMSO-d_6) δ ppm 7.87 (d, *J*=5.1 Hz, 1 H), 7.70 (d, *J*=3.4 Hz, 1 H), 7.41 (s, 1 H), 7.36 (t, *J*=7.5 Hz, 2 H), 7.29 (t, *J*=7.5 Hz, 1 H), 7.24 (d, *J*=7.2 Hz, 2 H), 7.16 (dd, *J*=5.1, 3.7 Hz, 1 H), 4.82 (s, 2 H), 2.25 (s, 3 H); ^{13}C NMR (75 MHz, DMSO-d_6) δ ppm 169.1, 162.0, 137.2, 136.8, 135.8, 135.1, 134.5, 128.8, 127.6, 127.5, 126.9, 119.9, 43.0, 15.7; HRMS (ESI) m/z: 283.0897 found (calcd for $C_{16}H_{15}N_2OS^+$, $[M+H]^+$ 283.0900).

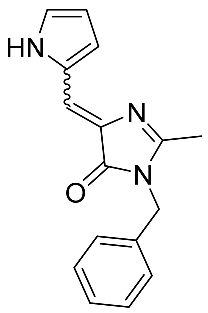

5-((1*H*-pyrrol-2-yl)methylene)-3-benzyl-2-methyl-3,5-dihydro-4*H*-imidazol-4-one (4ba)

Yellow solid (2.15 g, 81%); mp 127-129 ºC.

It was obtained as a mixture of two inseparable isomers. ^{1}H NMR signals were assigned to individual isomers:

(*E*)-5-((1*H*-pyrrol-2-yl)methylene)-3-benzyl-2-methyl-3,5-dihydro-4*H*-imidazol-4-one

^{1}H NMR (700 MHz, DMSO-d_6) δ ppm 12.84 (br. s., 1 H), 7.35 - 7.38 (m, 2 H), 7.32 (d, *J*=1.1 Hz, 1 H), 7.27 - 7.31 (m, 1 H), 7.24 - 7.27 (m, 3 H), 6.88 (br. s., 1 H), 6.35 (ddd, *J*=3.6, 2.5, 2.3 Hz, 1 H), 4.89 (s, 2 H), 2.19 (s, 3 H).

(*Z*)-5-((1*H*-pyrrol-2-yl)methylene)-3-benzyl-2-methyl-3,5-dihydro-4*H*-imidazol-4-one

^{1}H NMR (700 MHz, DMSO-d_6) δ ppm 11.28 (br. s., 1 H), 7.34 - 7.37 (m, 2 H), 7.27 - 7.31 (m, 1 H), 7.23 (d, *J*=7.1 Hz, 2 H), 7.17 (d, *J*=1.1 Hz, 1 H), 6.97 (s, 1 H), 6.86 (br. s., 1 H), 6.24 (ddd, *J*=3.5, 2.4, 2.2 Hz, 1 H), 4.81 (s, 2 H), 2.24 (s, 3 H).

^{13}C and HRMS data presented as is for a mixture:

^{13}C NMR (75 MHz, DMSO-d_6) δ ppm 168.9, 168.2, 159.1, 155.1, 137.0, 136.8, 132.4, 132.1, 128.8 (2 C), 128.3, 128.0, 127.5 (2 C), 126.9, 126.8, 125.8 (2 C), 125.6, 120.6, 118.4, 116.8, 111.7, 110.7, 43.3, 42.8, 15.4, 15.0; HRMS (ESI) m/z: 266.1286 found (calcd for $C_{16}H_{16}N_3O^+$, $[M+H]^+$ 266.1288).

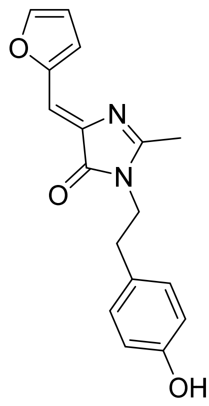

(*Z*)-5-(furan-2-ylmethylene)-3-(4-hydroxyphenethyl)-2-methyl-3,5-dihydro-4*H*-imidazol-4-one (1ca)

Brown solid (1.99 g, 67%); mp 149-151 ºC; ^{1}H NMR (700 MHz, DMSO-d_6) δ ppm 9.22 (s, 1 H), 7.91 (d, *J*=1.0 Hz, 1 H), 7.31 (d, *J*=3.2 Hz, 1 H), 6.96 (d, *J*=8.4 Hz, 2 H), 6.80 (s, 1 H), 6.70 (dd, *J*=3.0, 1.6 Hz, 1 H), 6.67 (d, *J*=8.4 Hz, 2 H), 3.70 (t, *J*=7.0 Hz, 2 H), 2.73 (t, *J*=7.1 Hz, 2 H), 2.03 (s, 3 H); ^{13}C NMR (75 MHz, DMSO-d_6) δ ppm 169.2, 163.3, 156.0, 150.3, 146.3, 136.0, 129.8, 128.2, 117.9, 115.3, 113.4, 112.0, 42.1, 33.4, 15.0; HRMS (ESI) m/z: 297.1231 found (calcd for $C_{17}H_{17}N_2O_3^+$, $[M+H]^+$ 297.1234).

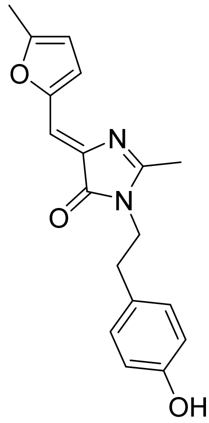

(*Z*)-5-((5-methylfuran-2-yl)methylene)-3-(4-hydroxyphenethyl)-2-methyl-3,5-dihydro-4*H*-imidazol-4-one (2ca)

Orange solid (1.71 g, 55%); mp 192-194 ºC; ^{1}H NMR (700 MHz, DMSO-d_6) δ ppm 9.22 (s, 1 H), 7.24 (d, *J*=2.9 Hz, 1 H), 6.95 (d, *J*=8.4 Hz, 2 H), 6.72 (s, 1 H), 6.67 (d, *J*=8.2 Hz, 2 H), 6.35 (d, *J*=2.5 Hz, 1 H), 3.69 (t, *J*=7.0 Hz, 2 H), 2.72 (t, *J*=7.1 Hz, 2 H), 2.36 (s, 3 H), 2.01 (s, 3 H); ^{13}C NMR (75 MHz, DMSO-d_6) δ ppm 169.2, 162.3, 156.0, 156.0, 149.1, 134.8, 129.8, 128.3, 119.7, 115.3, 112.1, 110.4, 42.1, 33.5, 15.0, 13.7; HRMS (ESI) m/z: 311.1388 found (calcd for $C_{18}H_{19}N_2O_3^+$, $[M+H]^+$ 311.1390).

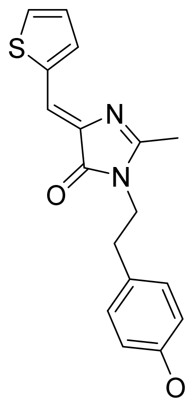

(*Z*)-5-(thiophen-2-ylmethylene)-3-(4-hydroxyphenethyl)-2-methyl-3,5-dihydro-4*H*-imidazol-4-one (3ca)

Yellow solid (2.22 g, 71%); mp 135-137 ºC; ^{1}H NMR (700 MHz, DMSO-d_6) δ ppm 9.22 (s, 1 H), 7.84 (d, *J*=5.0 Hz, 1 H), 7.66 (d, *J*=3.4 Hz, 1 H), 7.29 (s, 1 H), 7.15 (dd, *J*=5.1, 3.7 Hz, 1 H), 6.97 (d, *J*=8.4 Hz, 2 H), 6.67 (d, *J*=8.4 Hz, 2 H), 3.70 (t, *J*=7.1 Hz, 2 H), 2.74 (t, *J*=7.1 Hz, 2 H), 2.05 (s, 3 H); ^{13}C NMR (75 MHz, DMSO-d_6) δ ppm 169.0, 162.3, 156.0, 137.3, 136.1, 134.7, 134.2, 129.8, 128.2, 127.6, 119.0, 115.3, 42.1, 33.5, 15.1; HRMS (ESI) m/z: 313.1003 found (calcd for $C_{17}H_{17}N_2O_2S^+$, $[M+H]^+$ 313.1005).

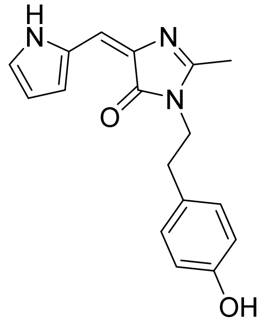

(*E*)-5-((1*H*-pyrrol-2-yl)methylene)-3-(4-hydroxyphenethyl)-2-methyl-3,5-dihydro-4*H*-imidazol-4-one (4ca)

Dark-yellow solid (2.07 g, 70%); mp ~230 ºC with decomposition; ^{1}H NMR (700 MHz, DMSO-d_6) δ ppm 12.88 (br. s., 1 H), 9.24 (br. s., 1 H), 7.31 (br. s., 1 H), 7.16 (s, 1 H), 6.96 (d, *J*=8.0 Hz, 2 H), 6.83 (br. s., 1 H), 6.68 (d, *J*=8.2 Hz, 2 H), 6.33 (br. s., 1 H), 3.76 (t, *J*=7.1 Hz, 2 H), 2.78 (t, *J*=7.1 Hz, 2 H), 1.97 (s, 3 H); ^{13}C NMR (75 MHz, DMSO-d_6) δ ppm 168.1, 156.0, 155.5, 132.4, 129.8, 128.4, 128.2, 125.4, 124.9, 120.1, 115.3, 111.5, 42.4, 33.4, 14.4; HRMS (ESI) m/z: 296.1391 found (calcd for $C_{17}H_{18}N_3O_2^+$, $[M+H]^+$ 296.1394).

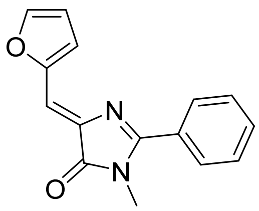

(*Z*)-5-(furan-2-ylmethylene)-3-methyl-2-phenyl-3,5-dihydro-4*H*-imidazol-4-one (1ab)

Yellow solid (0.60 g, 31%); mp 153-155 ºC; ^{1}H NMR (300 MHz, DMSO-d_6) δ ppm 7.99 (d, *J*=1.6 Hz, 1 H), 7.94 (dd, *J*=7.8, 1.6 Hz, 2 H), 7.56 - 7.67 (m, *3* H), 7.48 (d, *J*=3.5 Hz, 1 H), 7.02 (s, 1 H), 6.76 (dd, *J*=3.4, 1.8 Hz, 1 H), 3.27 (s, 3 H); ^{13}C NMR (75 MHz, DMSO-d_6) δ ppm 170.1, 162.1, 150.5, 146.8, 136.2, 131.6, 128.9, 128.8, 128.7, 119.0, 113.8 (2 C), 28.8; HRMS (ESI) m/z: 253.0971 found (calcd for $C_{15}H_{13}N_2O_2^+$, $[M+H]^+$ 253.0972).

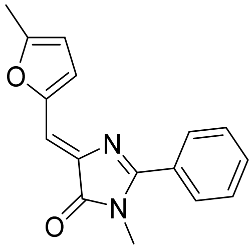

(*Z*)-5-((5-methylfuran-2-yl)methylene)-3-methyl-2-phenyl-3,5-dihydro-4*H*-imidazol-4-one (2ab)

Orange solid (0.40 g, 15%); mp 164-166 ºC; ^{1}H NMR (300 MHz, DMSO-d_6) δ ppm 7.92 (dd, *J*=7.9, 1.6 Hz, 2 H), 7.54 - 7.66 (m, 3 H), 7.42 (d, *J*=3.4 Hz, 1 H), 6.93 (s, 1 H), 6.42 (d, *J*=3.3 Hz, 1 H), 3.25 (s, 3 H), 2.38 (s, 3 H); ^{13}C NMR (75 MHz, DMSO-d_6) δ ppm 170.0, 161.1, 156.6, 149.4, 135.1, 131.4, 129.0, 128.8, 128.6, 120.8, 113.9, 110.8, 28.7, 13.8; HRMS (ESI) m/z: 267.1126 found (calcd for $C_{16}H_{15}N_2O_2^+$, $[M+H]^+$ 267.1128).

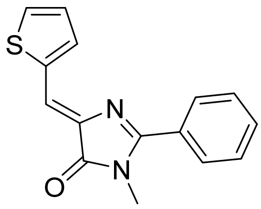

(*Z*)-5-(thiophen-2-ylmethylene)-3-methyl-2-phenyl-3,5-dihydro-4*H*-imidazol-4-one (3ab)

Yellow solid (0.56 g, 21%); mp 142-144 ºC; ^{1}H NMR (300 MHz, DMSO-d_6) δ ppm 7.88 - 8.03 (m, 3 H), 7.75 (d, *J*=3.4 Hz, 1 H), 7.52 - 7.69 (m, 4 H), 7.19 (dd, *J*=5.1, 3.7 Hz, 1 H), 3.28 (s, 3 H); ^{13}C NMR (75 MHz, DMSO-d_6) δ ppm 169.8, 161.1, 137.6, 136.4, 135.4, 135.1, 131.5, 129.0, 128.8, 128.7, 127.7, 121.2, 28.8; HRMS (ESI) m/z: 269.0740 found (calcd for $C_{15}H_{13}N_2OS^+$, $[M+H]^+$ 269.0743).

5.2 5-(Z)-arylidene-2-(E)-arylvinyl-3-methyl-3,5-dihydro-4H-imidazol-4-ones

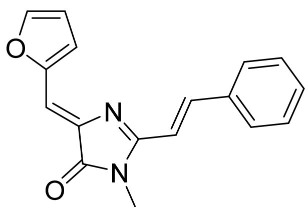

(*Z*)-5-(furan-2-ylmethylene)-3-methyl-2-((E)-styryl)-3,5-dihydro-4*H*-imidazol-4-one (1ac)

Brown solid (178 mg, 64%); mp 143-145 ºC; ^{1}H NMR (700 MHz, DMSO-d_6) δ ppm 8.04 (d, *J*=15.8 Hz, 1 H), 7.96 (d, *J*=1.3 Hz, 1 H), 7.87 (d, *J*=7.1 Hz, 2 H), 7.57 (d, *J*=3.4 Hz, 1 H), 7.43 - 7.49 (m, 3 H), 7.24 (d, *J*=15.8 Hz, 1 H), 6.88 (s, 1 H), 6.78 (dd, *J*=3.2, 1.5 Hz, 1 H), 3.28 (s, 3 H); ^{13}C NMR (75 MHz, DMSO-d_6) δ ppm 169.5, 160.0, 150.9, 146.4, 140.4, 137.1, 135.1, 130.2, 129.0, 128.4, 118.5, 113.9 (2 C), 111.9, 26.5; HRMS (ESI) m/z: 279.1125 found (calcd for $C_{17}H_{15}N_2O_2^+$, $[M+H]^+$ 279.1128).

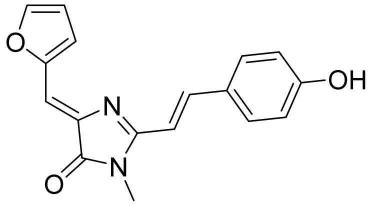

(*Z*)-5-(furan-2-ylmethylene)-3-methyl-2-((*E*)-4-hydroxystyryl)-3,5-dihydro-4*H*-imidazol-4-one (1ad)

Brown solid (270 mg, 92%); mp ~250 ºC with decomposition; ^{1}H NMR (300 MHz, DMSO-d_6) δ ppm 10.07 (s, 1 H), 7.90 - 8.02 (m, 2 H), 7.72 (d, *J*=8.3 Hz, 2 H), 7.52 (d, *J*=3.3 Hz, 1 H), 6.99 (d, *J*=15.6 Hz, 1 H), 6.85 (d, *J*=8.5 Hz, 2 H), 6.80 (s, 1 H), 6.76 (br. s., 1 H), 3.25 (s, 3 H); ^{13}C NMR (75 MHz, DMSO-d_6) δ ppm 169.5, 160.4, 159.9, 151.0, 146.1, 141.0, 137.3, 130.5, 126.4, 117.9, 115.9, 113.8, 110.7, 109.9, 26.4; HRMS (ESI) m/z: 295.1074 found (calcd for $C_{17}H_{15}N_2O_3^+$, $[M+H]^+$ 295.1077).

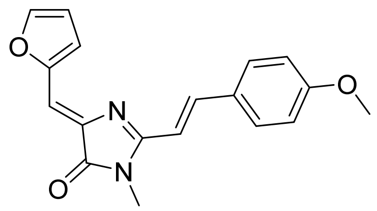

(*Z*)-5-(furan-2-ylmethylene)-3-methyl-2-((*E*)-4-methoxystyryl)-3,5-dihydro-4*H*-imidazol-4-one (1ae)

Orange solid (123 mg, 40%); mp 142-144 ºC; ^{1}H NMR (300 MHz, DMSO-d_6) δ ppm 8.01 (d, *J*=15.7 Hz, 1 H), 7.95 (d, *J*=1.3 Hz, 1 H), 7.84 (d, *J*=8.7 Hz, 2 H), 7.55 (d, *J*=3.4 Hz, 1 H), 7.00 - 7.12 (m, 3 H), 6.82 (s, 1 H), 6.77 (dd, *J*=4.3, 1.5 Hz, 1 H), 3.83 (s, 3 H), 3.26 (s, 3 H); ^{13}C NMR (75 MHz, DMSO-d_6) δ ppm 169.5, 161.1, 160.3, 151.0, 146.2, 140.5, 137.3, 130.3, 127.9, 118.1, 114.5, 113.8, 111.1 (2 C), 55.4, 26.5; HRMS (ESI) m/z: 309.1231 found (calcd for $C_{18}H_{17}N_2O_3^+$, $[M+H]^+$ 309.1234).

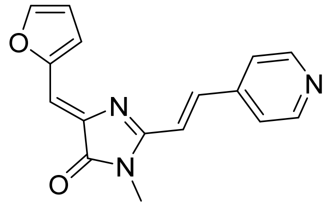

(*Z*)-5-(furan-2-ylmethylene)-3-methyl-2-((*E*)-2-(pyridine-4-yl)vinyl)-3,5-dihydro-4*H*-imidazol-4-one (1af)

Brown solid (117 mg, 42%); mp 192-194 ºC; ^{1}H NMR (300 MHz, DMSO-d_6) δ ppm 8.67 (d, *J*=5.6 Hz, 2 H), 7.93 - 8.04 (m, 2 H), 7.82 (d, *J*=5.9 Hz, 2 H), 7.60 (d, *J*=3.4 Hz, 1 H), 7.49 (d, *J*=15.9 Hz, 1 H), 6.95 (s, 1 H), 6.80 (dd, *J*=3.1, 1.4 Hz, 1 H), 3.28 (s, 3 H); ^{13}C NMR (75 MHz, DMSO-d_6) δ ppm 169.4, 159.3, 150.8, 150.4, 146.9, 142.1, 137.5, 136.8, 122.1, 119.2, 118.6, 114.0, 113.1, 26.6; HRMS (ESI) m/z: 280.1077 found (calcd for $C_{16}H_{14}N_3O_2^+$, $[M+H]^+$ 280.1081).

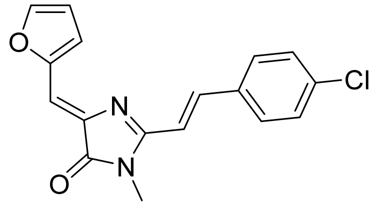

(*Z*)-5-(furan-2-ylmethylene)-3-methyl-2-((*E*)-4-chlorostyryl)-3,5-dihydro-4*H*-imidazol-4-one (1ag)

Orange solid (168 mg, 54%); mp 189-191 ºC; ^{1}H NMR (700 MHz, DMSO-d_6) δ ppm 8.03 (d, *J*=15.8 Hz, 1 H), 7.97 (d, *J*=1.1 Hz, 1 H), 7.91 (d, *J*=8.4 Hz, 2 H), 7.57 (d, *J*=3.4 Hz, 1 H), 7.54 (d, *J*=8.6 Hz, 2 H), 7.27 (d, *J*=15.8 Hz, 1 H), 6.89 (s, 1 H), 6.78 (dd, *J*=2.9, 1.5 Hz, 1 H), 3.27 (s, 3 H); ^{13}C NMR (176 MHz, DMSO-d_6) δ ppm 169.3, 159.7, 150.8, 146.4, 138.9, 137.0, 134.6, 134.1, 130.0, 128.9, 118.5, 114.6, 113.8, 112.0, 26.4; HRMS (ESI) m/z: 313.0734 found (calcd for $C_{17}H_{14}ClN_2O_2^+$, $[M+H]^+$ 313.0738).

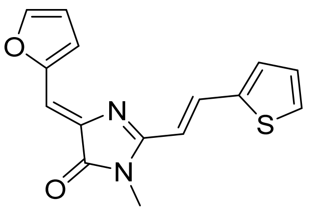

(*Z*)-5-(furan-2-ylmethylene)-3-methyl-2-((*E*)-2-(thiophen-2-yl)vinyl)-3,5-dihydro-4*H*-imidazol-4-one (1ah)

Red solid (162 mg, 57%); mp 154-156 ºC; ^{1}H NMR (300 MHz, DMSO-d_6) δ ppm 8.21 (d, *J*=15.5 Hz, 1 H), 7.96 (d, *J*=1.3 Hz, 1 H), 7.78 (d, *J*=4.9 Hz, 1 H), 7.67 (d, *J*=3.4 Hz, 1 H), 7.55 (d, *J*=3.4 Hz, 1 H), 7.20 (dd, *J*=5.0, 3.7 Hz, 1 H), 6.81 - 6.90 (m, 2 H), 6.77 (dd, *J*=3.3, 1.7 Hz, 1 H), 3.24 (s, 3 H); ^{13}C NMR (176 MHz, DMSO-d_6) δ ppm 169.3, 159.6, 150.9, 146.2, 140.3, 137.1, 133.1, 131.6, 129.9, 128.7, 118.2, 113.7, 112.1, 111.4, 26.3; HRMS (ESI) m/z: 285.0694 found (calcd for $C_{15}H_{13}N_2O_2S^+$, $[M+H]^+$ 285.0692).

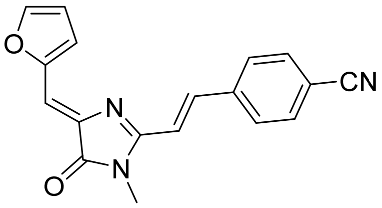

(*Z*)-5-(furan-2-ylmethylene)-3-methyl-2-((*E*)-4-cyanostyryl)-3,5-dihydro-4*H*-imidazol-4-one (1ai)

Orange solid (58 mg, 19%); mp 240-242 ºC; ^{1}H NMR (300 MHz, DMSO-d_6) δ ppm 8.03 - 8.13 (m, 3 H), 7.99 (d, *J*=1.1 Hz, 1 H), 7.94 (d, *J*=8.3 Hz, 2 H), 7.60 (d, *J*=3.4 Hz, 1 H), 7.43 (d, *J*=16.0 Hz, 1 H), 6.93 (s, 1 H), 6.79 (dd, *J*=3.0, 1.5 Hz, 1 H), 3.28 (s, 3 H); ^{13}C NMR (75 MHz, DMSO-d_6) δ ppm 169.3, 159.4, 150.8, 146.8, 139.6, 138.1, 136.9, 132.7, 129.0, 119.0, 118.7, 117.5, 113.9, 112.8, 111.8, 26.5; HRMS (ESI) m/z: 304.1079 found (calcd for $C_{18}H_{14}N_3O_2^+$, $[M+H]^+$ 304.1081).

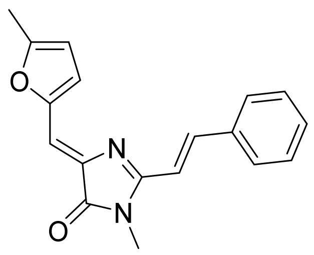

(*Z*)-5-((5-methylfuran-2-yl)methylene)-3-methyl-2-((*E*)-styryl)-3,5-dihydro-4*H*-imidazol-4-one (2ac)

Orange solid (184 mg, 63%); mp 145-147 ºC; ^{1}H NMR (300 MHz, DMSO-d_6) δ ppm 8.01 (d, *J*=15.8 Hz, 1 H), 7.86 (d, *J*=6.1 Hz, 2 H), 7.39 - 7.56 (m, 4 H), 7.22 (d, *J*=15.9 Hz, 1 H), 6.80 (s, 1 H), 6.45 (br. s., 1 H), 3.27 (s, 3 H), 2.40 (s, 3 H); ^{13}C NMR (75 MHz, DMSO-d_6) δ ppm 169.4, 159.0, 156.2, 149.7, 140.0, 136.0, 135.2, 130.1, 128.9, 128.4, 120.3, 113.9, 112.0, 110.9, 26.4, 13.8; HRMS (ESI) m/z: 293.1282 found (calcd for $C_{18}H_{17}N_2O_2^+$, $[M+H]^+$ 293.1285).

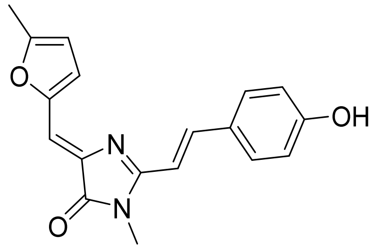

(*Z*)-5-((5-methylfuran-2-yl)methylene)-3-methyl-2-((*E*)-4-hydroxystyryl)-3,5-dihydro-4*H*-imidazol-4-one (2ad)

Red solid (253 mg, 82%); mp ~250 ºC with decomposition; ^{1}H NMR (300 MHz, DMSO-d_6) δ ppm 10.05 (br. s, 1 H), 7.94 (d, *J*=15.7 Hz, 1 H), 7.70 (d, *J*=8.5 Hz, 2 H), 7.47 (d, *J*=3.4 Hz, 1 H), 6.97 (d, *J*=15.7 Hz, 1 H), 6.84 (d, *J*=8.5 Hz, 2 H), 6.73 (s, 1 H), 6.42 (d, *J*=2.9 Hz, 1 H), 3.24 (s, 3 H), 2.38 (s, 3 H); ^{13}C NMR (75 MHz, DMSO-d_6) δ ppm 169.4, 159.8, 159.5, 155.8, 149.8, 140.5, 136.2, 130.4, 126.5, 119.7, 115.9, 110.9, 110.7, 110.0, 26.4, 13.8; HRMS (ESI) m/z: 309.1231 found (calcd for $C_{18}H_{17}N_2O_3^+$, $[M+H]^+$ 309.1234).

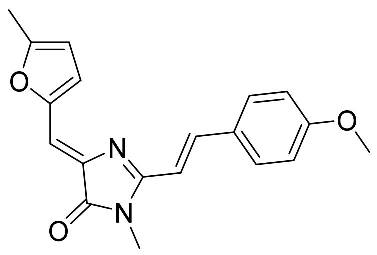

(*Z*)-5-((5-methylfuran-2-yl)methylene)-3-methyl-2-((*E*)-4-methoxystyryl)-3,5-dihydro-4*H*-imidazol-4-one (2ae)

Red solid (132 mg, 41%); mp 133-135 ºC; ^{1}H NMR (700 MHz, DMSO-d_6) δ ppm 7.98 (d, *J*=15.6 Hz, 1 H), 7.82 (d, *J*=8.8 Hz, 2 H), 7.49 (d, *J*=3.2 Hz, 1 H), 7.01 - 7.07 (m, 3 H), 6.75 (s, 1 H), 6.43 (d, *J*=3.2 Hz, 1 H), 3.83 (s, 3 H), 3.25 (s, 3 H), 2.39 (s, 3 H); ^{13}C NMR (75 MHz, DMSO-d_6) δ ppm 169.4, 161.0, 159.4, 155.9, 149.8, 140.0, 136.2, 130.2, 128.0, 119.8, 114.5, 111.2, 111.1, 110.8, 55.4, 26.4, 13.8; HRMS (ESI) m/z: 323.1387 found (calcd for $C_{19}H_{19}N_2O_3^+$, [M+H]$^+$ 323.1390).

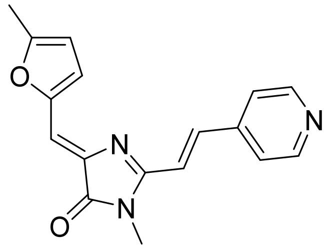

(*Z*)-5-((5-methylfuran-2-yl)methylene)-3-methyl-2-((*E*)-2-(pyridin-4-yl)vinyl)-3,5-dihydro-4*H*-imidazol-4-one (2af)

Red solid (182 mg, 62%); mp 174-176 ºC; ^{1}H NMR (300 MHz, DMSO-d_6) δ ppm 8.66 (br. s., 2 H), 7.95 (d, *J*=15.9 Hz, 1 H), 7.81 (d, *J*=4.8 Hz, 2 H), 7.55 (d, *J*=3.0 Hz, 1 H), 7.47 (d, *J*=15.7 Hz, 1 H), 6.87 (s, 1 H), 6.46 (d, *J*=2.2 Hz, 1 H), 3.27 (s, 3 H), 2.40 (s, 3 H); ^{13}C NMR (75 MHz, DMSO-d_6) δ ppm 169.2, 158.3, 156.8, 150.3, 149.6, 142.2, 137.0, 135.7, 122.1, 121.0, 118.6, 113.2, 111.1, 26.5, 13.8; HRMS (ESI) m/z: 294.1237 found (calcd for $C_{17}H_{16}N_3O_2^+$, [M+H]$^+$ 294.1237).

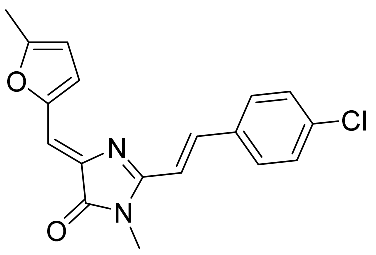

(*Z*)-5-((5-methylfuran-2-yl)methylene)-3-methyl-2-((*E*)-4-chlorostyryl)-3,5-dihydro-4*H*-imidazol-4-one (2ag)

Orange solid (241 mg, 74%); mp 177-179 ºC; ^{1}H NMR (300 MHz, DMSO-d_6) δ ppm 8.00 (d, *J*=15.7 Hz, 1 H), 7.90 (d, *J*=8.4 Hz, 2 H), 7.48 - 7.58 (m, 3 H), 7.25 (d, *J*=15.9 Hz, 1 H), 6.81 (s, 1 H), 6.45 (d, *J*=3.3 Hz, 1 H), 3.26 (s, 3 H), 2.40 (s, 3 H); ^{13}C NMR (176 MHz, DMSO-d_6) δ ppm 169.2, 158.8, 156.2, 149.6, 138.4, 135.9, 134.4, 134.1, 129.9, 128.9, 120.3, 114.6, 112.2, 110.8, 26.4, 13.7; HRMS (ESI) m/z: 327.0891 found (calcd for $C_{18}H_{16}ClN_2O_2^+$, $[M+H]^+$ 327.0895).

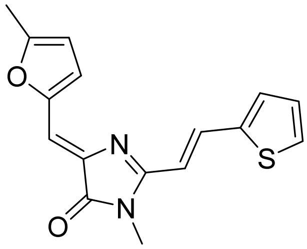

(*Z*)-5-((5-methylfuran-2-yl)methylene)-3-methyl-2-((*E*)-4-chlorostyryl)-3,5-dihydro-4*H*-imidazol-4-one (2ah)

Red solid (197 mg, 66%); mp 171-173 ºC; ^{1}H NMR (700 MHz, DMSO-d_6) δ ppm 8.17 (d, *J*=15.4 Hz, 1 H), 7.76 (d, *J*=5.1 Hz, 1 H), 7.65 (d, *J*=3.6 Hz, 1 H), 7.50 (d, *J*=3.4 Hz, 1 H), 7.19 (dd, *J*=5.0, 3.6 Hz, 1 H), 6.83 (d, *J*=15.6 Hz, 1 H), 6.77 (s, 1 H), 6.43 (d, *J*=3.4 Hz, 1 H), 3.23 (s, 3 H), 2.39 (s, 3 H); ^{13}C NMR (75 MHz, DMSO-d_6) δ ppm 169.2, 158.8, 156.1, 149.7, 140.4, 136.0, 132.8, 131.5, 129.8, 128.7, 120.1, 112.2, 111.6, 110.8, 26.3, 13.8; HRMS (ESI) m/z: 299.0845 found (calcd for $C_{16}H_{15}N_2O_2S^+$, $[M+H]^+$ 299.0849).

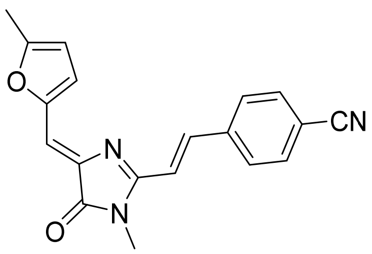

(*Z*)-5-((5-methylfuran-2-yl)methylene)-3-methyl-2-((*E*)-4-cyanostyryl)-3,5-dihydro-4*H*-imidazol-4-one (2ai)

Orange solid (117 mg, 37%); mp 222-224 ºC; ^{1}H NMR (300 MHz, DMSO-d_6) δ ppm 8.01 - 8.09 (m, 3 H), 7.93 (d, *J*=8.4 Hz, 2 H), 7.55 (d, *J*=3.2 Hz, 1 H), 7.41 (d, *J*=15.9 Hz, 1 H), 6.85 (s, 1 H), 6.46 (d, *J*=3.1 Hz, 1 H), 3.27 (s, 3 H), 2.40 (s, 3 H); ^{13}C NMR (75 MHz, DMSO-d_6) δ ppm 169.2, 158.5, 156.7, 149.7, 139.7, 137.7, 135.8, 132.7, 128.9, 120.9, 118.7, 117.5, 112.9, 111.7, 111.1, 26.5, 13.8; HRMS (ESI) m/z: 318.1228 found (calcd for $C_{19}H_{16}N_3O_2^+$, $[M+H]^+$ 318.1237).

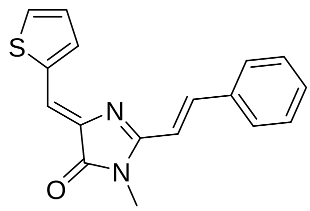

(*Z*)-5-(thiophen-2-ylmethylene)-3-methyl-2-((*E*)-styryl)-3,5-dihydro-4*H*-imidazol-4-one (3ac)

Orange solid (91 mg, 31%); mp 172-174 ºC; ^{1}H NMR (700 MHz, DMSO-d_6) δ ppm 7.97 (d, *J*=15.8 Hz, 1 H), 7.92 (d, *J*=5.0 Hz, 1 H), 7.85 (d, *J*=7.2 Hz, 2 H), 7.73 (d, *J*=3.6 Hz, 1 H), 7.44 - 7.49 (m, 3 H), 7.40 (s, 1 H), 7.24 (d, *J*=15.8 Hz, 1 H), 7.19 (dd, *J*=5.0, 3.6 Hz, 1 H), 3.28 (s, 3 H); ^{13}C NMR (75 MHz, DMSO-d_6) δ ppm 169.2, 159.2, 140.1, 138.1, 137.2, 135.1, 134.9, 134.7, 130.2, 129.0, 128.4, 127.8, 119.3, 114.1, 26.5; HRMS (ESI) m/z: 295.0897 found (calcd for $C_{17}H_{15}N_2OS^+$, $[M+H]^+$ 295.0900).

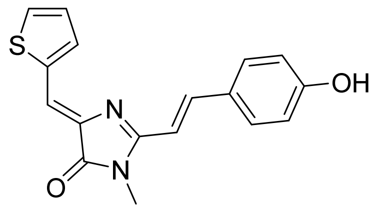

(*Z*)-5-(thiophen-2-ylmethylene)-3-methyl-2-((*E*)-4-hydroxystyryl)-3,5-dihydro-4*H*-imidazol-4-one (3ad)

Orange solid (180 mg, 58%); mp ~250 ºC with decomposition; ^{1}H NMR (300 MHz, DMSO-d_6) δ ppm 10.07 (br. s., 1 H), 7.85 - 7.96 (m, 2 H), 7.64 - 7.74 (m, 3 H), 7.32 (s, 1 H), 7.17 (dd, *J*=4.7, 3.7 Hz, 1 H), 6.99 (d, *J*=15.6 Hz, 1 H), 6.85 (d, *J*=8.6 Hz, 2 H), 3.25 (s, 3 H); ^{13}C NMR (75 MHz, DMSO-d_6) δ ppm 169.3, 159.8, 159.6, 140.6, 138.2, 137.4, 134.3, 134.1, 130.5, 127.7, 126.3, 118.0, 115.9, 110.1, 26.4; HRMS (ESI) m/z: 311.0846 found (calcd for $C_{17}H_{15}N_2O_2S^+$, $[M+H]^+$ 311.0849).

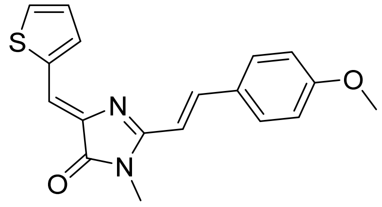

(*Z*)-5-(thiophen-2-ylmethylene)-3-methyl-2-((*E*)-4-methoxystyryl)-3,5-dihydro-4*H*-imidazol-4-one (3ae)

Orange solid (143 mg, 44%); mp 169-171 ºC; ^{1}H NMR (300 MHz, DMSO-d_6) δ ppm 7.94 (d, *J*=15.7 Hz, 1 H), 7.89 (d, *J*=5.1 Hz, 1 H), 7.82 (d, *J*=8.8 Hz, 2 H), 7.71 (d, *J*=3.4 Hz, 1 H), 7.34 (s, 1 H), 7.18 (dd, *J*=4.8, 3.9 Hz, 1 H), 7.00 - 7.12 (m, 3 H), 3.83 (s, 3 H), 3.26 (s, 3 H); ^{13}C NMR (75 MHz, DMSO-d_6) δ ppm 169.3, 161.1, 159.5, 140.1, 138.2, 137.4, 134.5, 134.3, 130.2, 127.8, 127.7, 118.4, 114.5, 111.3, 55.4, 26.4; HRMS (ESI) m/z: 325.1002 found (calcd for $C_{18}H_{17}N_2O_2S^+$, $[M+H]^+$ 325.1005).

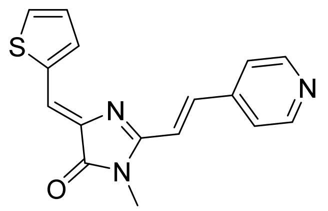

(*Z*)-5-(thiophen-2-ylmethylene)-3-methyl-2-((*E*)-2-(pyridine-4-yl)vinyl)-3,5-dihydro-4*H*-imidazol-4-one (3af)

Red-orange solid (150 mg, 51%); mp 203-205 ºC; ^{1}H NMR (300 MHz, DMSO-d_6) δ ppm 8.67 (d, *J*=5.1 Hz, 2 H), 7.96 (d, *J*=4.9 Hz, 1 H), 7.89 (d, *J*=15.8 Hz, 1 H), 7.81 (d, *J*=5.7 Hz, 2 H), 7.77 (d, *J*=3.4 Hz, 1 H), 7.44 - 7.55 (m, 2 H), 7.20 (dd, *J*=4.6, 4.1 Hz, 1 H), 3.29 (s, 3 H); ^{13}C NMR (75 MHz, DMSO-d_6) δ ppm 169.1, 158.5, 150.4, 142.1, 137.9, 137.1, 137.0, 135.5, 135.3, 127.9, 122.1, 120.6, 118.8, 26.6; HRMS (ESI) m/z: 296.0851 found (calcd for $C_{16}H_{14}N_3OS^+$, $[M+H]^+$ 296.0852).

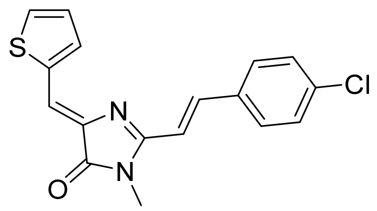

(*Z*)-5-(thiophen-2-ylmethylene)-3-methyl-2-((*E*)-4-chlorostyryl)-3,5-dihydro-4*H*-imidazol-4-one (3ag)

Orange solid (190 mg, 58%); mp 204-206 ºC; ^{1}H NMR (700 MHz, DMSO-d_6) δ ppm 7.94 (d, *J*=15.8 Hz, 1 H), 7.92 (d, *J*=5.1 Hz, 1 H), 7.89 (d, *J*=8.6 Hz, 2 H), 7.74 (d, *J*=3.6 Hz, 1 H), 7.54 (d, *J*=8.4 Hz, 2 H), 7.40 (s, 1 H), 7.27 (d, *J*=15.6 Hz, 1 H), 7.19 (dd, *J*=5.0, 3.8 Hz, 1 H), 3.27 (s, 3 H); ^{13}C NMR (176 MHz, DMSO-d_6) δ ppm 169.1, 158.9, 138.5, 138.0, 137.1, 134.8, 134.6 (2 C), 134.0, 129.9, 128.9, 127.7, 119.4, 114.8, 26.4; HRMS (ESI) m/z: 329.0511 found (calcd for $C_{17}H_{14}ClN_2OS^+$, $[M+H]^+$ 329.0510).

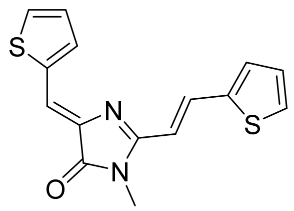

(*Z*)-5-(thiophen-2-ylmethylene)-3-methyl-2-((*E*)-2-(thuophen-2-yl)vinyl)-3,5-dihydro-4*H*-imidazol-4-one (3ah)

Orange solid (171 mg, 57%); mp 131-133 ºC; ^{1}H NMR (300 MHz, DMSO-d_6) δ ppm 8.11 (d, *J*=15.6 Hz, 1 H), 7.91 (d, *J*=5.1 Hz, 1 H), 7.78 (d, *J*=5.0 Hz, 1 H), 7.72 (d, *J*=3.6 Hz, 1 H), 7.67 (d, *J*=3.4 Hz, 1 H), 7.37 (s, 1 H), 7.17 – 7.19 (m, 2 H), 6.87 (d, *J*=15.5 Hz, 1 H), 3.24 (s, 3 H); ^{13}C NMR (176 MHz, DMSO-d_6) δ ppm 169.0, 158.8, 140.2, 138.0, 137.2, 134.6, 134.4, 132.7, 131.4, 129.8, 128.7, 127.7, 118.7, 112.3, 26.3; HRMS (ESI) m/z: 301.0463 found (calcd for $C_{15}H_{13}N_2OS_2^+$, $[M+H]^+$ 301.0464).

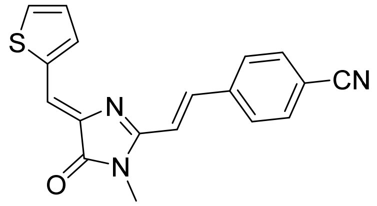

(*Z*)-5-(thiophen-2-ylmethylene)-3-methyl-2-((*E*)-4-cyanostyryl)-3,5-dihydro-4*H*-imidazol-4-one (3ai)

Red solid (124 mg, 39%); mp 231-233 ºC; ^{1}H NMR (300 MHz, DMSO-d_6) δ ppm 8.06 (d, *J*=8.4 Hz, 2 H), 7.91 - 8.02 (m, 4 H), 7.76 (d, *J*=3.4 Hz, 1 H), 7.38 - 7.49 (m, 2 H), 7.17 - 7.23 (m, 1 H), 3.28 (s, 3 H); ^{13}C NMR (75 MHz, DMSO-d_6) δ ppm 169.1, 158.6, 139.6, 138.0, 137.8, 137.1, 135.3, 135.1, 132.8, 128.9, 127.9, 120.3, 118.7, 117.7, 111.8, 26.5; HRMS (ESI) m/z: 320.0853 found (calcd for $C_{18}H_{14}N_3OS^+$, $[M+H]^+$ 320.0852).

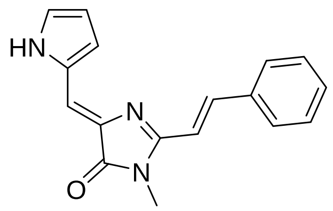

(*Z*)-5-((1*H*-pyrrol-2-yl)methylene)-3-methyl-2-((*E*)-styryl)-3,5-dihydro-4*H*-imidazol-4-one (4ac)

Orange solid (150 mg, 54%); mp 191-193 ºC; ^{1}H NMR (300 MHz, DMSO-d_6) δ ppm 11.42 (br. s., 1 H), 8.18 (d, *J*=15.9 Hz, 1 H), 7.83 (d, *J*=6.9 Hz, 2 H), 7.42 - 7.51 (m, 3 H), 7.25 (br. s., 1 H), 7.19 (d, *J*=15.8 Hz, 1 H), 6.96 (s, 1 H), 6.89 (br. s., 1 H), 6.24 - 6.34 (m, 1 H), 3.27 (s, 3 H); ^{13}C NMR (176 MHz, DMSO-d_6) δ ppm 168.9, 156.5, 139.2, 135.5, 133.8, 129.7, 128.9, 128.8, 128.0, 126.3, 118.8, 116.0, 113.8, 110.9, 26.2; HRMS (ESI) m/z: 278.1285 found (calcd for $C_{17}H_{16}N_3O^+$, $[M+H]^+$ 278.1288).

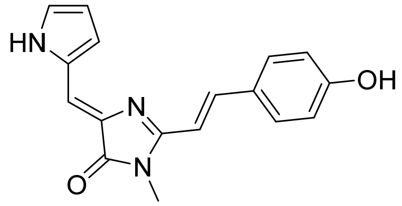

(*Z*)-5-((1*H*-pyrrol-2-yl)methylene)-3-methyl-2-((*E*)-4-hydroxystyryl)-3,5-dihydro-4*H*-imidazol-4-one (4ad)

Dark-red solid (149 mg, 51%); mp ~190 ºC with decomposition; ^{1}H NMR (300 MHz, DMSO-d_6) δ ppm 11.39 (br. s., 1 H), 10.00 (br. s., 1 H), 8.09 (d, *J*=15.8 Hz, 1 H), 7.68 (d, *J*=8.7 Hz, 2 H), 7.22 (br. s., 1 H), 6.94 (d, *J*=15.7 Hz, 1 H), 6.82 - 6.90 (m, 4 H), 6.22 - 6.31 (m, 1 H), 3.24 (s, 3 H); ^{13}C NMR (176 MHz, DMSO-d_6) δ ppm 169.0, 159.4, 157.0, 139.7, 134.0, 130.0, 128.9, 126.7, 125.8, 118.2, 115.8, 114.9, 110.7, 109.9, 26.2; HRMS (ESI) m/z: 294.1235 found (calcd for $C_{17}H_{16}N_3O_2^+$, $[M+H]^+$ 294.1237).

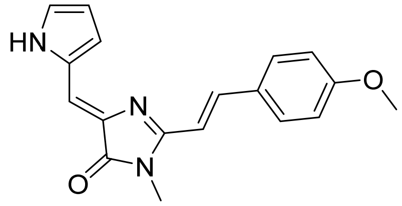

(*Z*)-5-((1*H*-pyrrol-2-yl)methylene)-3-methyl-2-((*E*)-4-methoxystyryl)-3,5-dihydro-4*H*-imidazol-4-one (4ae)

Orange solid (144 mg, 47%); mp 182-184 ºC; ^{1}H NMR (300 MHz, DMSO-d_6) δ ppm 11.40 (br. s., 1 H), 8.14 (d, *J*=15.8 Hz, 1 H), 7.79 (d, *J*=8.7 Hz, 2 H), 7.23 (br. s., 1 H), 6.99 - 7.07 (m, 3 H), 6.91 (s, 1 H), 6.86 (br. s., 1 H), 6.23 - 6.30 (m, 1 H), 3.83 (s, 3 H), 3.25 (s, 3 H); ^{13}C NMR (176 MHz, DMSO-d_6) δ ppm 168.9, 160.7, 156.9, 139.1, 133.9, 129.8, 128.9, 128.2, 125.9, 118.4, 115.2, 114.4, 111.1, 110.7, 55.3, 26.2; HRMS (ESI) m/z: 308.1391 found (calcd for $C_{18}H_{18}N_3O_2^+$, $[M+H]^+$ 308.1394).

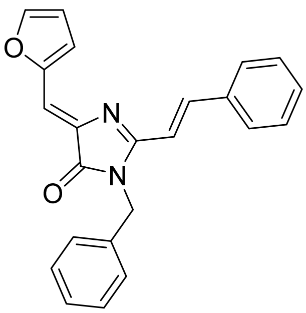

(*Z*)-5-((furan-2-ylmethylene)-3-benzyl-2-((*E*)-styryl)-3,5-dihydro-4*H*-imidazol-4-one (1bc)

Dark-red solid (198 mg, 56%); mp 157-159 ºC; ^{1}H NMR (700 MHz, DMSO-d_6) δ ppm 8.02 (d, *J*=15.6 Hz, 1 H), 7.99 (d, *J*=1.5 Hz, 1 H), 7.77 (d, *J*=7.1 Hz, 2 H), 7.61 (d, *J*=3.4 Hz, 1 H), 7.40 - 7.47 (m, 3 H), 7.35 (t, *J*=7.5 Hz, 2 H), 7.30 (d, *J*=7.4 Hz, 2 H), 7.26 (t, *J*=7.2 Hz, 1 H), 7.20 (d, *J*=15.8 Hz, 1 H), 6.96 (s, 1 H), 6.79 (dd, *J*=3.1, 1.4 Hz, 1 H), 5.07 (s, 2 H); ^{13}C NMR (75 MHz, DMSO-d_6) δ ppm 169.4, 159.3, 150.8, 146.7, 140.6, 137.3, 136.6, 135.0, 130.3, 129.0, 128.8, 128.4, 127.5, 126.9, 118.9, 114.0, 113.6, 112.6, 42.6; HRMS (ESI) m/z: 355.1436 found (calcd for $C_{23}H_{19}N_2O_2^+$, $[M+H]^+$ 355.1441).

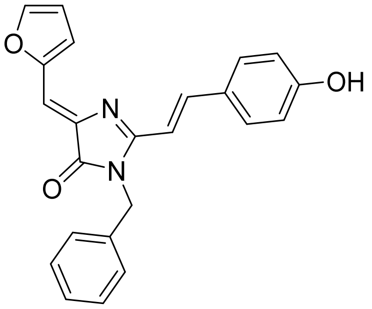

(*Z*)-5-((furan-2-ylmethylene)-3-benzyl-2-((*E*)-4-hydroxystyryl)-3,5-dihydro-4*H*-imidazol-4-one (1bd)

Dark-red solid (155 mg, 42%); mp 174-176 ºC; ^{1}H NMR (700 MHz, DMSO-d_6) δ ppm 10.06 (s, 1 H), 7.91 - 7.98 (m, 2 H), 7.62 (d, *J*=8.4 Hz, 2 H), 7.56 (d, *J*=3.4 Hz, 1 H), 7.34 (t, *J*=7.5 Hz, 2 H), 7.25 - 7.30 (m, 3 H), 6.94 (d, *J*=15.6 Hz, 1 H), 6.88 (s, 1 H), 6.82 (d, *J*=8.4 Hz, 2 H), 6.77 (d, *J*=1.7 Hz, 1 H), 5.03 (s, 2 H); ^{13}C NMR (75 MHz, DMSO-d_6) δ ppm 169.5, 159.9, 159.7, 151.0, 146.3, 141.1, 137.3, 136.8, 130.5, 128.8, 127.5, 126.9, 126.2, 118.3, 115.9, 113.8, 111.3, 109.7, 42.5; HRMS (ESI) m/z: 371.1384 found (calcd for $C_{23}H_{19}N_2O_3^+$, $[M+H]^+$ 371.1390).

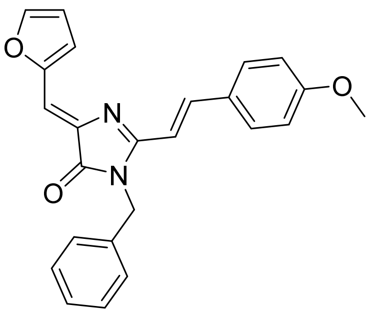

(*Z*)-5-((furan-2-ylmethylene)-3-benzyl-2-((*E*)-4-methoxystyryl)-3,5-dihydro-4*H*-imidazol-4-one (1be)

Dark-red solid (173 mg, 45%); mp 168-170 ºC; ^{1}H NMR (300 MHz, DMSO-d_6) δ ppm 7.94 - 8.04 (m, 2 H), 7.74 (d, *J*=8.7 Hz, 2 H), 7.59 (d, *J*=3.4 Hz, 1 H), 7.23 - 7.39 (m, 5 H), 6.97 - 7.09 (m, 3 H), 6.90 (s, 1 H), 6.78 (br. s., 1 H), 5.05 (s, 2 H), 3.81 (s, 3 H); ^{13}C NMR (75 MHz, DMSO-d_6) δ ppm 169.4, 161.2, 159.6, 150.9, 146.4, 140.6, 137.3, 136.8, 130.3, 128.8, 127.7, 127.5, 126.9, 118.4, 114.5, 113.9, 111.7, 110.9, 42.5, 29.0; HRMS (ESI) m/z: 385.1542 found (calcd for $C_{24}H_{21}N_2O_3^+$, $[M+H]^+$ 385.1547).

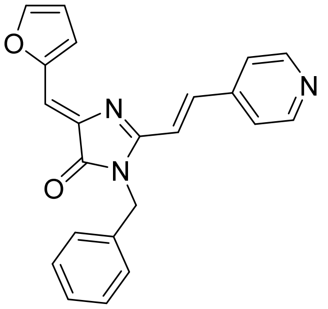

(*Z*)-5-((furan-2-ylmethylene)-3-benzyl-2-((*E*)-(pyridin-4-yl)vinyl)-3,5-dihydro-4*H*-imidazol-4-one (1bf)

Brown solid (85 mg, 24%); mp 184-186 ºC; ^{1}H NMR (700 MHz, DMSO-d_6) δ ppm 8.64 (d, *J*=5.9 Hz, 2 H), 8.02 (d, *J*=1.1 Hz, 1 H), 7.97 (d, *J*=15.8 Hz, 1 H), 7.72 (d, *J*=6.1 Hz, 2 H), 7.64 (d, *J*=3.6 Hz, 1 H), 7.47 (d, *J*=15.8 Hz, 1 H), 7.35 (t, *J*=7.5 Hz, 2 H), 7.28 - 7.32 (m, 2 H), 7.26 (t, *J*=7.2 Hz, 1 H), 7.03 (s, 1 H), 6.81 (dd, *J*=2.9, 1.7 Hz, 1 H), 5.08 (s, 2 H); ^{13}C NMR (75 MHz, DMSO-d_6) δ ppm 169.2, 158.6, 150.7, 150.4, 147.2, 141.9, 137.7, 137.2, 136.3, 128.8, 127.6, 126.9, 122.0, 119.6, 118.2, 114.1, 113.8, 42.6; HRMS (ESI) m/z: 356.1389 found (calcd for $C_{22}H_{18}N_3O_2^+$, $[M+H]^+$ 356.1394).

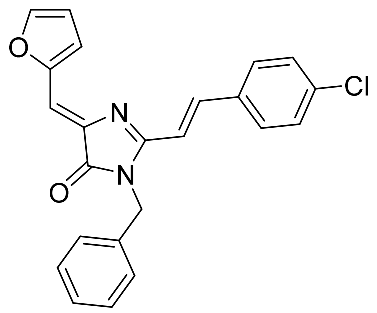

(*Z*)-5-((furan-2-ylmethylene)-3-benzyl-2-((*E*)-4-chlorostyryl)-3,5-dihydro-4*H*-imidazol-4-one (1bg)

Orange solid (58 mg, 15%); mp 189-191 ºC; ^{1}H NMR (700 MHz, DMSO-d_6) δ ppm 8.01 (d, *J*=15.8 Hz, 1 H), 7.99 (d, *J*=1.1 Hz, 1 H), 7.82 (d, *J*=8.4 Hz, 2 H), 7.61 (d, *J*=3.4 Hz, 1 H), 7.51 (d, *J*=8.6 Hz, 2 H), 7.34 (t, *J*=7.6 Hz, 2 H), 7.28 (d, *J*=7.4 Hz, 2 H), 7.22 - 7.27 (m, 2 H), 6.96 (s, 1 H), 6.79 (d, *J*=1.3 Hz, 1 H), 5.06 (s, 2 H); ^{13}C NMR (75 MHz, DMSO-d_6) δ ppm 169.3, 159.1, 150.8, 146.8, 139.2, 137.2, 136.5, 134.8, 134.0, 130.1, 129.0, 128.8, 127.5, 126.9, 119.1, 114.4, 114.0, 112.8, 42.6; HRMS (ESI) m/z: 389.1047 found (calcd for $C_{23}H_{18}ClN_2O_2^+$, $[M+H]^+$ 389.1051).

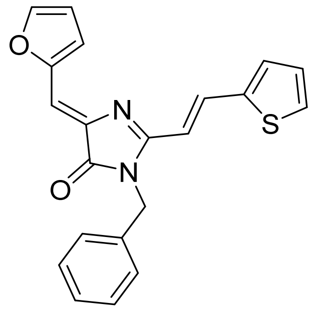

(*Z*)-5-((furan-2-ylmethylene)-3-benzyl-2-((*E*)-2-(thiophen-2-yl)vinyl)-3,5-dihydro-4*H*-imidazol-4-one (1bh)

Red solid (133 mg, 37%); mp 145-147 ºC; ^{1}H NMR (300 MHz, DMSO-d_6) δ ppm 8.16 (d, *J*=15.6 Hz, 1 H), 7.98 (br. s, 1 H), 7.73 (d, *J*=5.3 Hz, 1 H), 7.56 - 7.62 (m, 2 H), 7.32 - 7.39 (m, 2 H), 7.24 - 7.30 (m, 3 H), 7.17 (dd, *J*=4.5, 3.8 Hz, 1 H), 6.93 (s, 1 H), 6.73 - 6.84 (m, 2 H), 5.02 (s, 2 H); ^{13}C NMR (75 MHz, DMSO-d_6) δ ppm 169.3, 159.0, 150.9, 146.6, 140.3, 137.2, 136.7, 133.3 (2 C), 131.8, 130.2, 128.8, 127.6, 126.9, 118.8, 113.9, 112.1 (2 C), 42.5; HRMS (ESI) m/z: 361.1001 found (calcd for $C_{21}H_{17}N_2O_2S^+$, $[M+H]^+$ 361.1005).

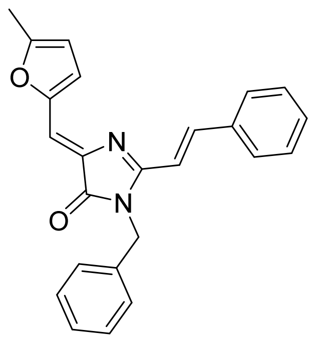

(*Z*)-5-((5-methylfuran-2-yl)methylene)-3-benzyl-2-((*E*)-styryl)-3,5-dihydro-4*H*-imidazol-4-one (2bc)

Dark-red solid (224 mg, 61%); mp 105-107 ºC; ^{1}H NMR (700 MHz, DMSO-d_6) δ ppm 7.99 (d, *J*=15.8 Hz, 1 H), 7.76 (d, *J*=7.1 Hz, 2 H), 7.56 (d, *J*=3.2 Hz, 1 H), 7.40 – 7.46 (m, 3 H), 7.34 (t, *J*=7.6 Hz, 2 H), 7.29 (d, *J*=7.2 Hz, 2 H), 7.26 (t, *J*=7.2 Hz, 1 H), 7.18 (d, *J*=15.8 Hz, 1 H), 6.88 (s, 1 H), 6.46 (d, *J*=3.2 Hz, 1 H), 5.05 (s, 2 H), 2.41 (s, 3 H); ^{13}C NMR (75 MHz, DMSO-d_6) δ ppm 169.3, 158.3, 156.5, 149.7, 140.1, 137.3, 135.5, 135.1, 130.2, 129.0, 128.8, 128.3, 127.5, 126.9, 120.7, 113.7, 112.7, 111.0, 42.5, 13.8; HRMS (ESI) m/z: 369.1592 found (calcd for $C_{24}H_{21}N_2O_2^+$, $[M+H]^+$ 369.1598).

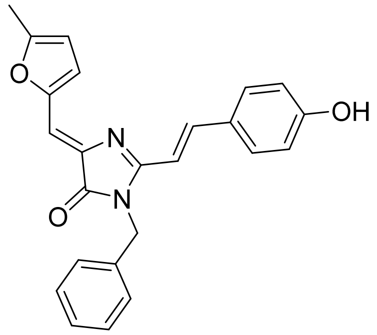

(*Z*)-5-((5-methylfuran-2-yl)methylene)-3-benzyl-2-((*E*)-4-hydroxystyryl)-3,5-dihydro-4*H*-imidazol-4-one (2bd)

Dark-red solid (242 mg, 63%); mp ~220 ºC with decomposition; ^{1}H NMR (300 MHz, DMSO-d_6) δ ppm 10.05 (s, 1 H), 7.91 (d, *J*=15.7 Hz, 1 H), 7.61 (d, *J*=8.4 Hz, 2 H), 7.51 (d, *J*=3.4 Hz, 1 H), 7.24 - 7.37 (m, 5 H), 6.93 (d, *J*=15.5 Hz, 1 H), 6.76 - 6.85 (m, 3 H), 6.44 (d, *J*=3.0 Hz, 1 H), 5.02 (s, 2 H), 2.40 (s, 3 H); ^{13}C NMR (75 MHz, DMSO-d_6) δ ppm 169.4, 159.8, 158.8, 156.1, 149.8, 140.6, 137.4, 135.8, 130.4, 128.7, 127.5, 126.9, 126.3, 120.1, 115.9, 111.5, 110.9, 109.8, 42.5, 13.8; HRMS (ESI) m/z: 385.1541 found (calcd for $C_{24}H_{21}N_2O_3^+$, $[M+H]^+$ 385.1547).

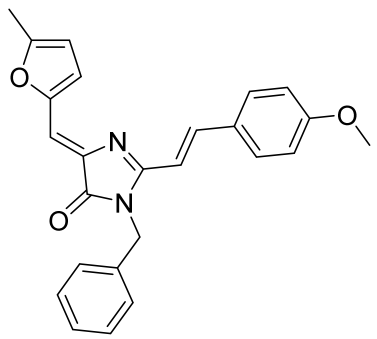

(*Z*)-5-((5-methylfuran-2-yl)methylene)-3-benzyl-2-((*E*)-4-methoxystyryl)-3,5-dihydro-4*H*-imidazol-4-one (2be)

Dark-red solid (107 mg, 27%); mp 146-148 ºC; ^{1}H NMR (700 MHz, DMSO-d_6) δ ppm 7.96 (d, *J*=15.6 Hz, 1 H), 7.73 (d, *J*=8.7 Hz, 2 H), 7.54 (d, *J*=3.3 Hz, 1 H), 7.24 - 7.37 (m, 5 H), 6.97 - 7.06 (m, 3 H), 6.83 (s, 1 H), 6.45 (d, *J*=3.1 Hz, 1 H), 5.03 (s, 2 H), 3.81 (s, 3 H), 2.40 (s, 3 H); ^{13}C NMR (75 MHz, DMSO-d_6) δ ppm 169.3, 161.1, 158.7, 156.2, 149.8, 140.1, 137.4, 135.7, 130.1, 128.7, 127.8, 127.5, 126.9, 120.3, 114.5, 113.4, 111.8, 110.9, 55.4, 42.5, 13.8; HRMS (ESI) m/z: 399.1699 found (calcd for $C_{25}H_{23}N_2O_3^+$, $[M+H]^+$ 399.1703).

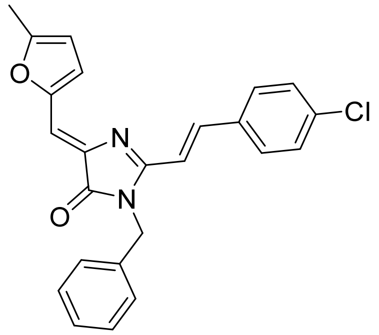

(*Z*)-5-((5-methylfuran-2-yl)methylene)-3-benzyl-2-((*E*)-4-chlorostyryl)-3,5-dihydro-4*H*-imidazol-4-one (2bg)

Red solid (180 mg, 45%); mp 168-170 ºC; ^{1}H NMR (300 MHz, DMSO-d_6) δ ppm 7.98 (d, *J*=15.8 Hz, 1 H), 7.81 (d, *J*=8.5 Hz, 2 H), 7.57 (d, *J*=3.2 Hz, 1 H), 7.51 (d, *J*=8.4 Hz, 2 H), 7.18 - 7.38 (m, 6 H), 6.88 (s, 1 H), 6.46 (d, *J*=3.1 Hz, 1 H), 5.05 (s, 2 H), 2.40 (s, 3 H); ^{13}C NMR (75 MHz, DMSO-d_6) δ ppm 169.2, 158.2, 156.7, 149.7, 138.7, 137.3, 135.4, 134.6, 134.0, 130.0, 129.0, 128.8, 127.5, 126.9, 120.9, 114.4, 112.9, 111.1, 42.5, 13.8; HRMS (ESI) m/z: 403.1204 found (calcd for $C_{24}H_{20}ClN_2O_2^+$, $[M+H]^+$ 403.1208).

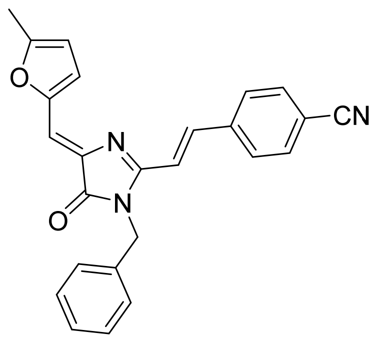

(*Z*)-5-((5-methylfuran-2-yl)methylene)-3-benzyl-2-((*E*)-4-cyanostyryl)-3,5-dihydro-4*H*-imidazol-4-one (2bi)

Red solid (189 mg, 48%); mp 167-169 ºC; ^{1}H NMR (300 MHz, DMSO-d_6) δ ppm 8.03 (d, *J*=15.8 Hz, 1 H), 7.97 (d, *J*=8.4 Hz, 2 H), 7.90 (d, *J*=8.4 Hz, 2 H), 7.60 (d, *J*=3.4 Hz, 1 H), 7.39 (d, *J*=15.6 Hz, 1 H), 7.23 - 7.23 (m, 5 H), 6.93 (s, 1 H), 6.48 (d, *J*=3.2 Hz, 1 H), 5.06 (s, 2 H), 2.41 (s, 3 H); ^{13}C NMR (176 MHz, DMSO-d_6) δ ppm 169.1, 157.7, 156.9, 149.6, 139.5, 137.8, 137.2, 135.3, 132.6, 128.7 (2 C), 127.4, 126.8, 121.3, 118.6, 117.1, 113.5, 111.7, 111.1, 42.5, 13.7; HRMS (ESI) m/z: 394.1557 found (calcd for $C_{25}H_{20}N_3O_2^+$, $[M+H]^+$ 394.1550).

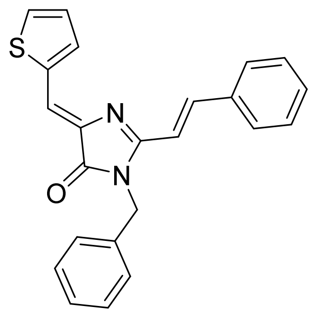

(*Z*)-5-(thiophen-2-ylmethylene)-3-benzyl-2-((*E*)-styryl)-3,5-dihydro-4*H*-imidazol-4-one (3bc)

Orange solid (222 mg, 60%); mp 185-187 ºC; ^{1}H NMR (700 MHz, DMSO-d_6) δ ppm 7.92 – 7.96 (m, 2 H), 7.77 (d, *J*=3.6 Hz, 1 H), 7.75 (d, *J*=6.8 Hz, 2 H), 7.48 (s, 1 H), 7.42 - 7.47 (m, 3 H), 7.35 (t, *J*=7.6 Hz, 2 H), 7.30 (d, *J*=7.3 Hz, 2 H), 7.26 (t, *J*=7.3 Hz, 1 H), 7.18 - 7.23 (m, 2 H), 5.07 (s, 2 H); ^{13}C NMR (176 MHz, DMSO-d_6) δ ppm 169.1, 158.5, 140.2, 138.0, 137.3, 136.7, 135.3, 135.1, 134.9, 130.3, 129.0, 128.8, 128.3, 127.9, 127.5, 126.9, 120.0, 113.8, 42.5; HRMS (ESI) m/z: 371.1207 found (calcd for $C_{23}H_{19}N_2OS^+$, $[M+H]^+$ 371.1213).

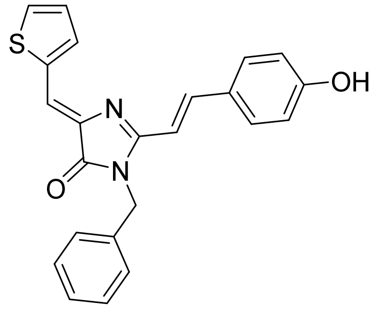

(*Z*)-5-(thiophen-2-ylmethylene)-3-benzyl-2-((*E*)-4-hydroxystyryl)-3,5-dihydro-4*H*-imidazol-4-one (3bd)

Orange solid (154 mg, 40%); mp ~250 ºC with decomposition; ^{1}H NMR (300 MHz, DMSO-d_6) δ ppm 10.08 (br. s., 1 H), 7.83 - 7.93 (m, 2 H), 7.73 (d, *J*=3.5 Hz, 1 H), 7.60 (d, *J*=8.6 Hz, 2 H), 7.40 (s, 1 H), 7.23 - 7.38 (m, 5 H), 7.19 (dd, *J*=5.1, 3.8 Hz, 1 H), 6.96 (d, *J*=15.7 Hz, 1 H), 6.82 (d, *J*=8.6 Hz, 2 H), 5.04 (s, 2 H); ^{13}C NMR (75 MHz, DMSO-d_6) δ ppm 169.2, 159.9, 158.9, 140.7, 138.1, 137.4, 136.9, 134.7, 134.5, 130.4, 128.8, 127.8, 127.5, 126.9, 126.1, 118.7, 116.0, 109.9, 42.5; HRMS (ESI) m/z: 387.1156 found (calcd for $C_{23}H_{19}N_2O_2S^+$, $[M+H]^+$ 387.1162).

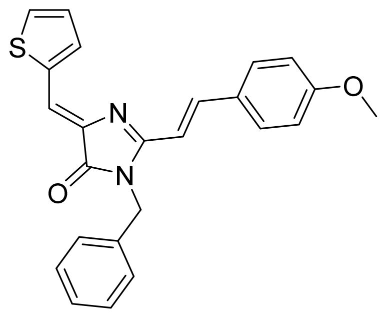

(*Z*)-5-(thiophen-2-ylmethylene)-3-benzyl-2-((*E*)-4-methoxystyryl)-3,5-dihydro-4*H*-imidazol-4-one (3be)

Red solid (200 mg, 49%); mp 204-206 ºC; ^{1}H NMR (300 MHz, DMSO-*d*$_6$) δ ppm 7.86 - 7.97 (m, 2 H), 7.67 - 7.77 (m, 3 H), 7.42 (s, 1 H), 7.24 - 7.38 (m, 5 H), 7.20 (dd, *J*=5.0, 3.8 Hz, 1 H), 6.96 - 7.09 (m, 3 H), 5.05 (s, 2 H), 3.82 (s, 3 H); ^{13}C NMR (75 MHz, DMSO-*d*$_6$) δ ppm 169.2, 161.1, 158.8, 140.2, 138.1, 137.4, 136.9, 134.8, 134.7, 130.2, 128.8, 127.8, 127.6, 127.5, 126.9, 119.1, 114.6, 111.1, 55.4, 42.5; HRMS (ESI) m/z: 401.1314 found (calcd for $C_{24}H_{21}N_2O_2S^+$, $[M+H]^+$ 401.1318).

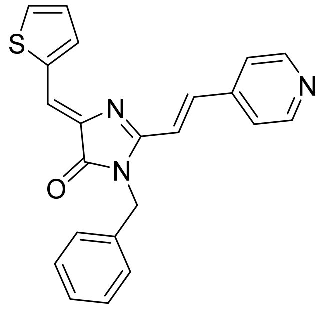

(*Z*)-5-(thiophen-2-ylmethylene)-3-benzyl-2-((*E*)-2-(pyridine-4-yl)vinyl)-3,5-dihydro-4*H*-imidazol-4-one (3bf)

Orange solid (156 mg, 42%); mp 173-175 ºC; ^{1}H NMR (300 MHz, DMSO-*d*$_6$) δ ppm 8.65 (d, *J*=5.3 Hz, 2 H), 7.99 (d, *J*=5.0 Hz, 1 H), 7.88 (d, *J*=15.7 Hz, 1 H), 7.81 (d, *J*=3.6 Hz, 1 H), 7.72 (d, *J*=5.9 Hz, 2 H), 7.56 (s, 1 H), 7.48 (d, *J*=15.7 Hz, 1 H), 7.26 - 7.38 (m, 5 H), 7.22 (dd, *J*=4.9, 3.9 Hz, 1 H), 5.08 (s, 2 H); ^{13}C NMR (75 MHz, DMSO-*d*$_6$) δ ppm 168.9, 157.8, 150.4, 141.8, 137.8, 137.3, 137.2, 136.5, 135.9, 135.7, 128.8, 128.0, 127.6, 126.9, 122.0, 121.4, 118.4, 42.6; HRMS (ESI) m/z: 372.1160 found (calcd for $C_{22}H_{18}N_3OS^+$, $[M+H]^+$ 372.1165).

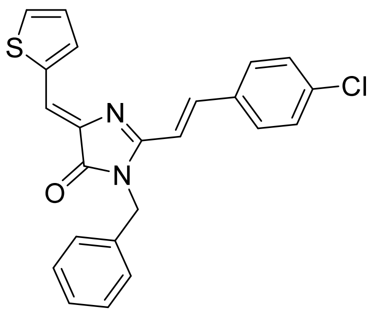

(Z)-5-(thiophen-2-ylmethylene)-3-benzyl-2-((E)-4-chlorostyryl)-3,5-dihydro-4H-imidazol-4-one (3bg)

Orange solid (141 mg, 35%); mp 210-212 ºC; ^{1}H NMR (700 MHz, DMSO-d_6) δ ppm 7.95 (d, J=5.0 Hz, 1 H), 7.92 (d, J=15.8 Hz, 1 H), 7.80 (d, J=8.4 Hz, 2 H), 7.78 (d, J=3.6 Hz, 1 H), 7.51 (d, J=8.6 Hz, 2 H), 7.48 (s, 1 H), 7.34 (t, J=7.5 Hz, 2 H), 7.29 (d, J=7.4 Hz, 2 H), 7.23 - 7.28 (m, 2 H), 7.21 (dd, J=4.9, 3.9 Hz, 1 H), 5.06 (s, 2 H); ^{13}C NMR (75 MHz, DMSO-d_6) δ ppm 169.1, 158.3, 138.8, 138.0, 137.3, 136.7, 135.4, 135.2, 134.7, 133.9, 130.0, 129.0, 128.8, 127.9, 127.5, 126.9, 120.3, 114.6, 42.5; HRMS (ESI) m/z: 405.0819 found (calcd for $C_{23}H_{18}ClN_2OS^+$, $[M+H]^+$ 405.0823).

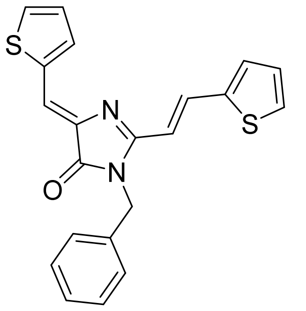

(Z)-5-(thiophen-2-ylmethylene)-3-benzyl-2-((E)-2-(thiophen-2-yl)vinyl)-3,5-dihydro-4H-imidazol-4-one (3bh)

Orange solid (165 mg, 44%); mp 182-184 ºC; ^{1}H NMR (300 MHz, DMSO-d_6) δ ppm 8.06 (d, J=15.5 Hz, 1 H), 7.93 (d, J=5.1 Hz, 1 H), 7.70 - 7.79 (m, 2 H), 7.59 (d, J=3.4 Hz, 1 H), 7.46 (s, 1 H), 7.24 - 7.40 (m, 5 H), 7.13 - 7.23 (m, 2 H), 6.80 (d, J=15.4 Hz, 1 H), 5.02 (s, 2 H); ^{13}C NMR (75 MHz, DMSO-d_6) δ ppm 169.0, 158.2, 140.1, 138.0, 137.2, 136.8, 135.1, 134.9, 132.8, 131.7, 130.1, 128.8 (3 C), 127.9, 127.6, 126.9, 119.6, 112.2, 42.5; HRMS (ESI) m/z: 377.0772 found (calcd for $C_{21}H_{17}N_2OS_2^+$, $[M+H]^+$ 377.0777).

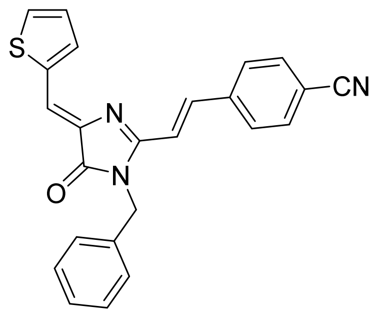

(*Z*)-5-(thiophen-2-ylmethylene)-3-benzyl-2-((*E*)-4-cyanostyryl)-3,5-dihydro-4*H*-imidazol-4-one (3bi)

Orange solid (40 mg, 10%); mp 193-195 ºC; ^{1}H NMR (300 MHz, DMSO-d_6) δ ppm 7.88 - 8.01 (m, 6 H), 7.80 (d, *J*=3.8 Hz, 1 H), 7.54 (s, 1 H), 7.42 (d, *J*=16.0 Hz, 1 H), 7.25 - 7.37 (m, 5 H), 7.22 (dd, *J*=4.5, 4.1 Hz, 1 H), 5.08 (s, 2 H); ^{13}C NMR (75 MHz, DMSO-d_6) δ ppm 169.0, 157.9, 139.4, 138.0, 137.9, 137.2, 136.6, 135.8, 135.6, 132.8, 128.9, 128.8, 128.0, 127.5, 126.9, 121.1, 118.7, 117.4, 111.9, 42.6; HRMS (ESI) m/z: 396.1154 found (calcd for $C_{24}H_{18}N_3OS^+$, $[M+H]^+$ 396.1165).

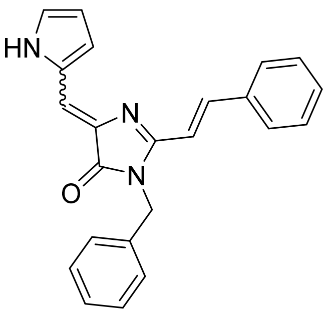

5-((1*H*-pyrrol-2-yl)methylene)-3-benzyl-2-((*E*)-styryl)-3,5-dihydro-4*H*-imidazol-4-one (4bc)

Dark-red solid (243 mg, 69%); mp 120-122 ºC.

It was obtained as a mixture of two inseparable isomers. ^{1}H NMR signals were assigned to individual isomers:

(*E*)-5-((1*H*-pyrrol-2-yl)methylene)-3-benzyl-2-((*E*)-styryl)-3,5-dihydro-4*H*-imidazol-4-one ^{1}H NMR (700 MHz, DMSO-d_6) δ ppm 12.97 (br. s., 1 H), 7.71 - 7.77 (m, 1 H), 7.70 (d, *J*=7.3 Hz, 2 H), 7.23 - 7.46 (m, 9 H), 7.14 (d, *J*=15.8 Hz, 1 H), 6.94 (br. s., 1 H), 6.39 (d, *J*=3.5 Hz, 1 H), 5.15 (s, 2 H).

(*Z*)-5-((1*H*-pyrrol-2-yl)methylene)-3-benzyl-2-((*E*)-styryl)-3,5-dihydro-4*H*-imidazol-4-one ^{1}H NMR (700 MHz, DMSO-d_6) δ ppm 11.45 (br. s., 1 H), 8.15 (d, *J*=15.8 Hz, 1 H), 7.71 - 7.77 (m, 2 H), 7.23 - 7.46 (m, 8 H), 7.14 (d, J=15.8 Hz, 1 H), 7.03 (s, 1 H), 6.94 (br. s., 1 H), 6.31 (d, *J*=3.3 Hz, 1 H), 5.05 (s, 2 H).

^{13}C and HRMS data presented as is for a mixture:

^{13}C NMR (75 MHz, DMSO-d_6) δ ppm 168.8, 168.0, 155.8, 152.6, 139.2, 137.5, 137.3, 137.2, 135.4, 135.3, 133.3, 133.1, 129.8, 129.6, 129.2, 128.9, 128.8 (2 C), 128.7 (2 C), 128.0, 127.8, 127.5, 127.4, 126.9, 126.8, 126.7, 126.6, 125.8, 121.3, 119.3, 116.7, 113.8, 113.6, 112.1, 111.1, 42.8, 42.4; HRMS (ESI) m/z: 354.1597 found (calcd for $C_{23}H_{20}N_3O^+$, $[M+H]^+$ 354.1601).

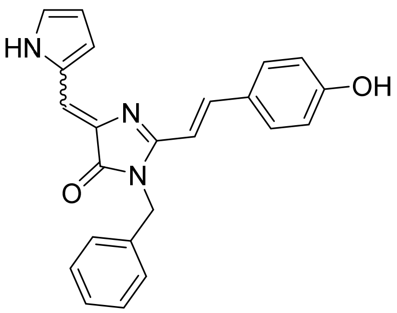

5-((1*H*-pyrrol-2-yl)methylene)-3-benzyl-2-((*E*)-4-hydroxystyryl)-3,5-dihydro-4*H*-imidazol-4-one (4bd)

Red solid (79 mg, 22%); mp ~240 ºC with decomposition.

It was obtained as a mixture of two inseparable isomers. ^{1}H NMR signals were assigned to individual isomers:

(*E*)-5-((1*H*-pyrrol-2-yl)methylene)-3-benzyl-2-((*E*)-4-hydroxystyryl)-3,5-dihydro-4*H*-imidazol-4-one

^{1}H NMR (700 MHz, DMSO-d_6) δ ppm 12.93 (br. s., 1 H), 9.91 (br. s., 1 H), 7.66 (d, *J*=15.8 Hz, 1 H), 7.54 (d, *J*=8.6 Hz, 2 H), 7.23 - 7.36 (m, 7 H), 6.85 - 6.92 (m, 2 H), 6.79 (d, *J*=8.6 Hz, 2 H), 6.35 - 6.39 (m, 1 H), 5.11 (s, 2 H).

(*Z*)-5-((1*H*-pyrrol-2-yl)methylene)-3-benzyl-2-((*E*)-4-hydroxystyryl)-3,5-dihydro-4*H*-imidazol-4-one

^{1}H NMR (700 MHz, DMSO-d_6) δ ppm 11.41 (br. s., 1 H), 9.98 (br. s., 1 H), 8.07 (d, *J*=15.6 Hz, 1 H), 7.58 (d, *J*=8.6 Hz, 4 H), 7.23 - 7.36 (m, 6 H), 6.97 (s, 1 H), 6.85 - 6.92 (m, 2 H), 6.82 (d, *J*=8.6 Hz, 2 H), 6.27 - 6.30 (m, 1 H), 5.02 (s, 2 H).

^{13}C and HRMS data presented as is for a mixture:

^{13}C NMR (75 MHz, DMSO-d_6) δ ppm 168.9, 168.1, 159.5, 159.2, 156.4, 153.2, 139.7, 137.6 (2 C), 137.3, 133.5, 133.3, 130.0, 129.7, 129.2, 128.9, 128.7 (2 C), 127.4 (2 C), 126.9, 126.8, 126.6, 126.5, 126.2, 126.1, 124.8, 120.7, 118.7, 115.9, 115.8, 115.5, 111.9, 110.9, 110.1, 109.8, 42.8, 42.3; HRMS (ESI) m/z: 370.1544 found (calcd for $C_{23}H_{20}N_3O_2^+$, $[M+H]^+$ 370.1550).

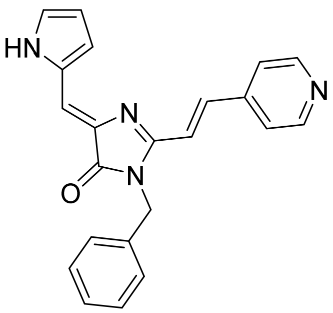

(*Z*)-5-((1*H*-pyrrol-2-yl)methylene)-3-benzyl-2-((*E*)-2-(pyridine-4-yl)vinyl)-3,5-dihydro-4*H*-imidazol-4-one (4bf)

Brown solid (193 mg, 55%); mp 196-198 ºC; ^{1}H NMR (700 MHz, DMSO-d_6) δ ppm 11.48 (br. s., 1 H), 8.64 (dd, *J*=4.5, 1.4 Hz, 2 H), 8.09 (d, *J*=15.8 Hz, 1 H), 7.66 (dd, *J*=4.6, 1.3 Hz, 2 H), 7.41 (d, *J*=15.8 Hz, 1 H), 7.34 (t, *J*=7.5 Hz, 2 H), 7.28 - 7.31 (m, 3 H), 7.26 (t, *J*=7.2 Hz, 1 H), 7.10 (s, 1 H), 6.99 (br. s., 1 H), 6.33 (dt, *J*=3.7, 2.3 Hz, 1 H), 5.07 (s, 2 H); ^{13}C NMR (176 MHz, DMSO-d_6) δ ppm 168.6, 154.9, 150.3, 142.3, 137.4, 136.2, 133.1, 128.8, 128.7 (2 C), 127.4, 126.8, 121.6 , 120.0, 118.1, 117.8, 111.3, 42.4; HRMS (ESI) m/z: 355.1548 found (calcd for $C_{22}H_{19}N_4O^+$, $[M+H]^+$ 355.1553).

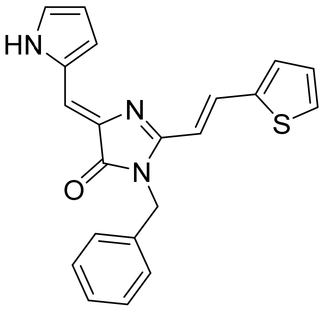

(*Z*)-5-((1*H*-pyrrol-2-yl)methylene)-3-benzyl-2-((*E*)-2-(thiophen-2-yl)vinyl)-3,5-dihydro-4*H*-imidazol-4-one (4bh)

Red solid (180 mg, 50%); mp 152-154 ºC; ^{1}H NMR (700 MHz, DMSO-d_6) δ ppm 11.44 (br. s., 1 H), 8.28 (d, *J*=15.4 Hz, 1 H), 7.69 (d, *J*=5.0 Hz, 1 H), 7.50 (d, *J*=3.4 Hz, 1 H), 7.34 - 7.36 (m, 2 H), 7.26 - 7.28 (m, 4 H), 7.16 (dd, *J*=5.0, 3.8 Hz, 1 H), 7.01 (s, 1 H), 6.77 (d, *J*=15.4 Hz, 1 H), 6.30 (d, *J*=3.4 Hz, 1 H), 5.01 (s, 2 H); ^{13}C NMR (176 MHz, DMSO-d_6) δ ppm 168.7, 155.4, 140.6, 137.4, 133.3, 131.9, 130.2, 129.2, 128.9, 128.7, 128.6, 127.4, 126.8, (2 C), 119.2, 116.3, 112.3, 111.0, 42.3; HRMS (ESI) m/z: 360.1155 found (calcd for $C_{21}H_{18}N_3OS^+$, $[M+H]^+$ 360.1165).

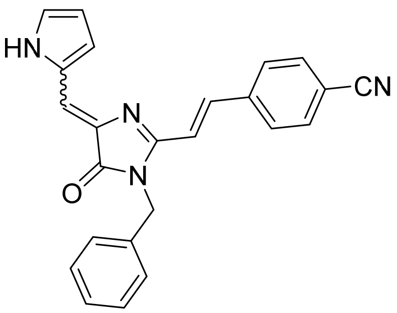

5-((1*H*-pyrrol-2-yl)methylene)-3-benzyl-2-((*E*)-4-cyanostyryl)-3,5-dihydro-4*H*-imidazol-4-one (4bi)

Red solid (210 mg, 56%); mp 201-203 ºC.

It was obtained as a mixture of two inseparable isomers. ^{1}H NMR signals were assigned to individual isomers:

(*E*)-5-((1*H*-pyrrol-2-yl)methylene)-3-benzyl-2-((*E*)-4-cyanostyryl)-3,5-dihydro-4*H*-imidazol-4-one

^{1}H NMR (700 MHz, DMSO-d_6) δ ppm 13.00 (br. s., 1 H), 7.90 - 7.94 (m, 2 H), 7.86 - 7.88 (m, 2 H), 7.79 (d, *J*=15.8 Hz, 1 H), 7.41 - 7.43 (m, 2 H), 7.25 - 7.37 (m, 6 H), 6.99 (br. s., 1 H), 6.41 (ddd, J=3.8, 2.4, 2.2 Hz, 1 H), 5.16 (s, 2 H).

(*Z*)-5-((1*H*-pyrrol-2-yl)methylene)-3-benzyl-2-((*E*)-4-cyanostyryl)-3,5-dihydro-4*H*-imidazol-4-one

^{1}H NMR (700 MHz, DMSO-d_6) δ ppm 11.47 (br. s., 1 H), 8.18 (d, *J*=15.8 Hz, 1 H), 7.90 - 7.94 (m, 4 H), 7.25 - 7.37 (m, 7 H), 7.08 (s, 1 H), 6.99 (br. s., 1 H), 6.33 (ddd, *J*=3.7, 2.3, 2.1 Hz, 1 H), 5.06 (s, 2 H).

^{13}C and HRMS data presented as is for a mixture:

^{13}C NMR (176 MHz, DMSO-d_6) δ ppm 168.6, 167.8, 155.1, 151.9, 139.8, 137.4, 137.1, 137.0, 135.0, 133.2, 132.9, 132.7 (2 C), 132.6, 129.2, 128.9, 128.7, 128.6, 128.4 (2 C), 128.3, 127.4 (2 C), 127.1, 126.9, 126.8, 126.5, 121.8, 119.9, 118.6, 117.5, 117.3, 117.1, 112.4, 111.4, 111.3, 111.2, 42.9, 42.4; HRMS (ESI) m/z: 379.1545 found (calcd for $C_{24}H_{19}N_4O^+$, $[M+H]^+$ 379.1553).

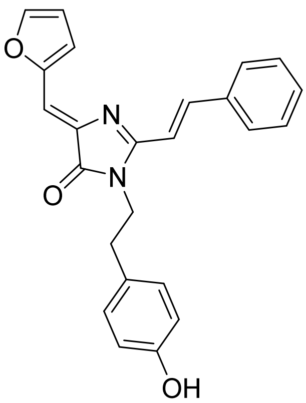

(*Z*)-5-(furan-2-ylmethylene)-3-(4-hydroxyphenethyl)-2-((*E*)-styryl)-3,5-dihydro-4*H*-imidazol-4-one (1cc)

Brown solid (316 mg, 82%); mp 193-195 ºC; ^{1}H NMR (700 MHz, DMSO-d_6) δ ppm 9.13 (s, 1 H), 7.96 (d, *J*=1.1 Hz, 1 H), 7.92 (d, *J*=15.6 Hz, 1 H), 7.75 (d, *J*=7.2 Hz, 2 H), 7.55 (d, *J*=3.4 Hz, 1 H), 7.46 (t, *J*=7.2 Hz, 2 H), 7.41 - 7.44 (m, 1 H), 7.00 (d, *J*=8.4 Hz, 2 H), 6.89 (d, *J*=15.6 Hz, 1 H), 6.85 (s, 1 H), 6.77 (dd, *J*=3.1, 1.5 Hz, 1 H), 6.63 (d, *J*=8.4 Hz, 2 H), 3.97 (t, *J*=7.0 Hz, 2 H), 2.76 (t, *J*=7.0 Hz, 2 H); ^{13}C NMR (75 MHz, DMSO-d_6) δ ppm 169.3, 159.6, 156.0, 150.9, 146.5, 140.1, 136.8, 135.1, 130.1, 129.9, 128.9, 128.4, 128.0, 118.5, 115.2, 113.9, 113.6, 111.8, 41.3, 34.1; HRMS (ESI) m/z: 385.1542 found (calcd for $C_{24}H_{21}N_2O_3^+$, $[M+H]^+$ 385.1547).

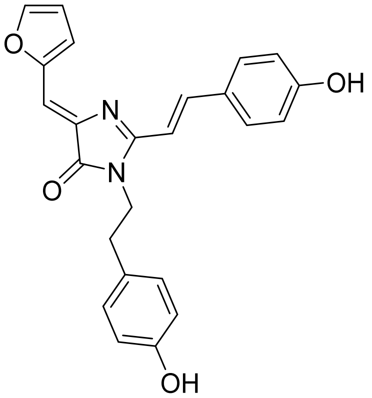

(*Z*)-5-(furan-2-ylmethylene)-3-(4-hydroxyphenethyl)-2-((*E*)-4-hydroxystyryl)-3,5-dihydro-4*H*-imidazol-4-one (1cd)

Red solid (133 mg, 33%); mp ~220 ºC with decomposition; ^{1}H NMR (700 MHz, DMSO-d_6) δ ppm 10.03 (s, 1 H), 9.13 (s, 1 H), 7.93 (d, *J*=1.1 Hz, 1 H), 7.87 (d, *J*=15.4 Hz, 1 H), 7.61 (d, *J*=8.6 Hz, 2 H), 7.50 (d, *J*=3.4 Hz, 1 H), 7.00 (d, *J*=8.4 Hz, 2 H), 6.84 (d, *J*=8.6 Hz, 2 H), 6.76 (s, 1 H), 6.74 - 6.76 (m, 1 H), 6.68 (d, *J*=15.6 Hz, 1 H), 6.63 (d, *J*=8.4 Hz, 2 H), 3.93 (t, *J*=7.0 Hz, 2 H), 2.75 (t, *J*=7.0 Hz, 2 H); ^{13}C NMR (75 MHz, DMSO-d_6) δ ppm 169.3, 160.0, 159.7, 155.9, 151.0, 146.0, 140.6, 137.0, 130.4, 129.8, 128.1, 126.3, 117.8, 115.8, 115.2, 113.7, 110.6, 109.7, 41.2, 34.1; HRMS (ESI) m/z: 401.1491 found (calcd for $C_{24}H_{21}N_2O_4^+$, $[M+H]^+$ 401.1496).

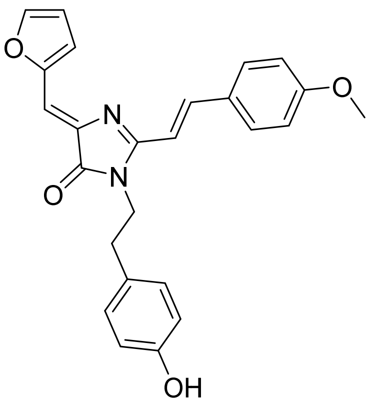

(*Z*)-5-(furan-2-ylmethylene)-3-(4-hydroxyphenethyl)-2-((*E*)-4-methoxystyryl)-3,5-dihydro-4*H*-imidazol-4-one (1ce)

Brown solid (213 mg, 51%); mp 201-203 ºC; ^{1}H NMR (700 MHz, DMSO-d_6) δ ppm 9.12 (s, 1 H), 7.93 (br. s, 1 H), 7.90 (d, *J*=15.6 Hz, 1 H), 7.72 (d, *J*=8.6 Hz, 2 H), 7.52 (d, *J*=3.2 Hz, 1 H), 7.02 (d, *J*=8.8 Hz, 2 H), 7.00 (d, *J*=8.2 Hz, 2 H), 6.79 (s, 1 H), 6.72 - 6.78 (m, 2 H), 6.63 (d, *J*=8.4 Hz, 2 H), 3.95 (t, *J*=6.9 Hz, 2 H), 3.83 (s, 3 H), 2.75 (t, *J*=6.8 Hz, 2 H); ^{13}C NMR (75 MHz, DMSO-d_6) δ ppm 169.3, 161.0, 155.9, 151.0, 146.2, 133.0, 130.2, 129.9, 129.8, 128.1, 127.9, 118.1, 115.2 (2 C), 114.4, 113.8, 113.4, 110.9, 55.4, 41.2, 34.1; HRMS (ESI) m/z: 415.1648 found (calcd for $C_{25}H_{23}N_2O_4^+$, $[M+H]^+$ 415.1652).

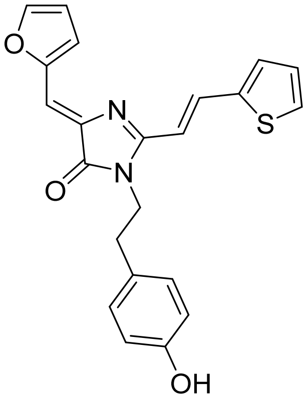

(*Z*)-5-(furan-2-ylmethylene)-3-(4-hydroxyphenethyl)-2-((*E*)-2-(thiophen-2-yl)vinyl)-3,5-dihydro-4*H*-imidazol-4-one (1ch)

Brown solid (334 mg, 86%); mp 169-171 ºC; ^{1}H NMR (700 MHz, DMSO-d_6) δ ppm 9.12 (s, 1 H), 8.06 (d, *J*=15.4 Hz, 1 H), 7.95 (br. s., 1 H), 7.75 (d, *J*=4.6 Hz, 1 H), 7.58 (br. s., 1 H), 7.53 (d, *J*=2.9 Hz, 1 H), 7.16 - 7.19 (m, 1 H), 6.97 (d, *J*=8.2 Hz, 2 H), 6.82 (s, 1 H), 6.76 (br. s., 1 H), 6.63 (d, *J*=8.0 Hz, 2 H), 6.49 (d, *J*=15.3 Hz, 1 H), 3.91 (t, *J*=6.6 Hz, 2 H), 2.74 (t, *J*=6.6 Hz, 3 H); ^{13}C NMR (75 MHz, DMSO-d_6) δ ppm 169.2, 159.3, 156.0, 150.9, 146.4, 140.4, 136.9, 132.9, 131.6, 129.9, 129.8, 128.6, 128.0, 118.4, 115.3, 113.8, 111.9, 111.4, 41.4, 34.0; HRMS (ESI) m/z: 391.1106 found (calcd for $C_{22}H_{19}N_2O_3S^+$, $[M+H]^+$ 391.1111).

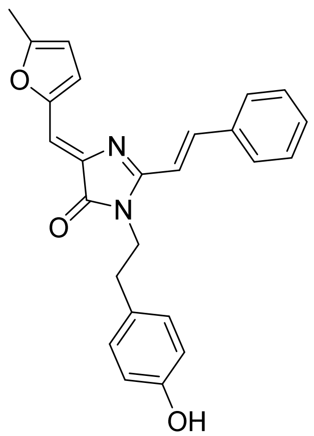

(*Z*)-5-((5-methylfuran-2-yl)methylene)-3-(4-hydroxyphenethyl)-2-((*E*)-styryl)-3,5-dihydro-4*H*-imidazol-4-one (2cc)

Red solid (212 mg, 53%); mp ~210 ºC with decomposition; ^{1}H NMR (300 MHz, DMSO-d_6) δ ppm 9.14 (s, 1 H), 7.89 (d, *J*=15.7 Hz, 1 H), 7.74 (d, *J*=6.6 Hz, 2 H), 7.39 - 7.52 (m, 4 H), 7.00 (d, *J*=8.2 Hz, 2 H), 6.88 (d, *J*=15.7 Hz, 1 H), 6.77 (s, 1 H), 6.63 (d, *J*=8.3 Hz, 2 H), 6.43 (d, *J*=2.8 Hz, 1 H), 3.95 (t, *J*=6.5 Hz, 2 H), 2.75 (t, *J*=6.5 Hz, 2 H), 2.39 (s, 3 H); ^{13}C NMR (75 MHz, DMSO-d_6) δ ppm 169.2, 158.7, 156.3, 155.9, 149.7, 139.7, 135.7, 135.2, 130.0, 129.9, 128.9, 128.3, 128.1, 120.3, 115.2, 113.6, 111.9, 110.9, 41.2, 34.1, 13.8; HRMS (ESI) m/z: 399.1699 found (calcd for $C_{25}H_{23}N_2O_3^+$, $[M+H]^+$ 399.1703).

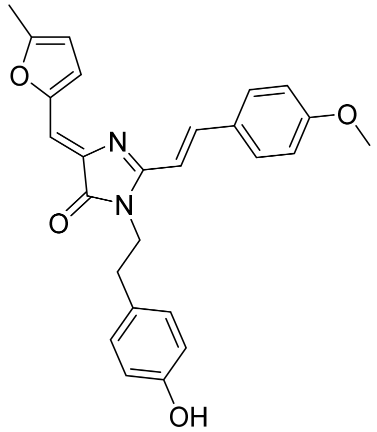

(*Z*)-5-((5-methylfuran-2-yl)methylene)-3-(4-hydroxyphenethyl)-2-((*E*)-4-methoxystyryl)-3,5-dihydro-4*H*-imidazol-4-one (2ce)

Brown solid (146 mg, 34%); mp 192-194 ºC; ^{1}H NMR (700 MHz, DMSO-d_6) δ ppm 9.12 (s, 1 H), 7.86 (d, *J*=15.4 Hz, 1 H), 7.71 (d, *J*=8.8 Hz, 2 H), 7.47 (d, *J*=3.1 Hz, 1 H), 6.99 - 7.03 (m, 4 H), 6.71 - 6.75 (m, 2 H), 6.63 (d, *J*=8.2 Hz, 2 H), 6.42 (br. s., 1 H), 3.94 (t, *J*=7.0 Hz, 2 H), 3.83 (s, 3 H), 2.75 (t, *J*=7.0 Hz, 2 H), 2.39 (s, 3 H); ^{13}C NMR (75 MHz, DMSO-d_6) δ ppm 169.2, 160.9, 159.0, 155.9 (2 C), 149.8, 139.7, 135.9, 130.1, 129.9, 128.1, 127.9, 119.9, 115.2, 114.4, 111.1, 110.9, 110.8, 55.4, 41.2, 34.1, 13.8; HRMS (ESI) m/z: 429.1806 found (calcd for $C_{26}H_{25}N_2O_4^+$, $[M+H]^+$ 429.1809).

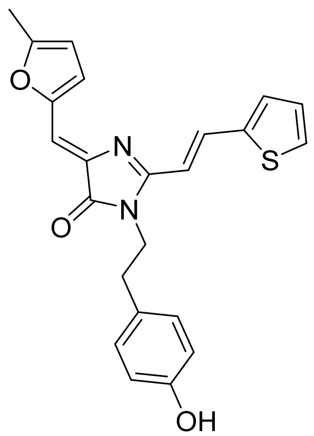

(*Z*)-5-((5-methylfuran-2-yl)methylene)-3-(4-hydroxyphenethyl)-2-((*E*)-2-(thiophen-2-yl)vinyl)-3,5-dihydro-4*H*-imidazol-4-one (2ch)

Dark-red solid (102 mg, 25%); mp 244-246 ºC; ^{1}H NMR (700 MHz, DMSO-d_6) δ ppm 9.13 (s, 1 H), 8.02 (d, *J*=15.4 Hz, 1 H), 7.73 (d, *J*=5.0 Hz, 1 H), 7.56 (d, *J*=3.2 Hz, 1 H), 7.48 (d, *J*=3.2 Hz, 1 H), 7.17 (dd, *J*=5.0, 3.6 Hz, 1 H), 6.97 (d, *J*=8.2 Hz, 2 H), 6.74 (s, 1 H), 6.63 (d, *J*=8.4 Hz, 2 H), 6.48 (d, *J*=15.4 Hz, 1 H), 6.43 (d, *J*=3.1 Hz, 1 H), 3.90 (t, *J*=6.9 Hz, 2 H), 2.73 (t, *J*=6.9 Hz, 2 H), 2.39 (s, 3 H); ^{13}C NMR (176 MHz, DMSO-d_6) δ ppm 169.0, 158.3, 156.1, 155.9, 149.7, 140.4, 135.7, 132.3, 131.2, 129.7, 129.6, 128.5, 128.0, 120.1, 115.2, 112.0, 111.4, 110.8, 41.3, 34.0, 13.7; HRMS (ESI) m/z: 405.1263 found (calcd for $C_{23}H_{21}N_2O_3S^+$, $[M+H]^+$ 405.1267).

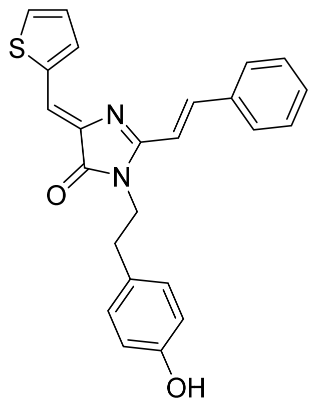

(*Z*)-5-(thiophen-2-ylmethylene)-3-(4-hydroxyphenethyl)-2-((*E*)-styryl)-3,5-dihydro-4*H*-imidazol-4-one (3cc)

Orange solid (219 mg, 55%); mp 101-103 ºC; ^{1}H NMR (700 MHz, DMSO-d_6) δ ppm 9.13 (s, 1 H), 7.91 (d, *J*=5.0 Hz, 1 H), 7.86 (d, *J*=15.6 Hz, 1 H), 7.70 - 7.77 (m, 3 H), 7.47 (t, *J*=7.2 Hz, 2 H), 7.44 (t, *J*=6.7 Hz, 1 H), 7.36 (s, 1 H), 7.19 (dd, *J*=5.1, 3.7 Hz, 1 H), 7.01 (m, *J*=8.4 Hz, 2 H), 6.91 (d, *J*=15.6 Hz, 1 H), 6.63 (m, *J*=8.4 Hz, 2 H), 3.97 (t, *J*=7.0 Hz, 2 H), 2.77 (t, *J*=7.0 Hz, 2 H); ^{13}C NMR (75 MHz, DMSO-d_6) δ ppm 169.0, 158.8, 156.0, 139.8, 138.0, 136.9, 135.0, 134.9, 134.7, 130.1, 129.9, 129.0, 128.3, 128.1, 127.8, 119.2, 115.2, 113.8, 41.2, 34.1; HRMS (ESI) m/z: 401.1314 found (calcd for $C_{24}H_{21}N_2O_2S^+$, $[M+H]^+$ 401.1318).

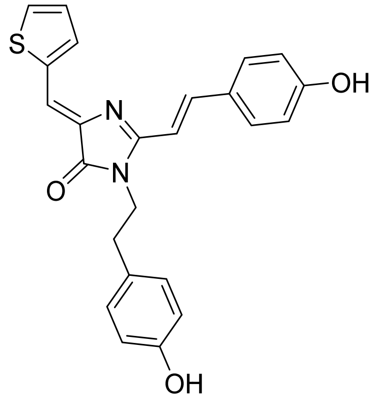

(*Z*)-5-(thiophen-2-ylmethylene)-3-(4-hydroxyphenethyl)-2-((*E*)-4-hydroxystyryl)-3,5-dihydro-4*H*-imidazol-4-one (3cd)

Orange solid (200 mg, 48%); mp ~240 ºC with decomposition; ^{1}H NMR (700 MHz, DMSO-d_6) δ ppm 10.04 (br. s., 1 H), 9.13 (s, 1 H), 7.87 (d, *J*=5.0 Hz, 1 H), 7.81 (d, *J*=15.6 Hz, 1 H), 7.69 (d, *J*=2.9 Hz, 1 H), 7.59 (d, *J*=8.2 Hz, 2 H), 7.28 (s, 1 H), 7.16 - 7.18 (m, 1 H), 7.01 (d, *J*=7.8 Hz, 2 H), 6.85 (d, *J*=7.6 Hz, 2 H), 6.69 (d, *J*=15.4 Hz, 1 H), 6.64 (d, *J*=8.2 Hz, 2 H), 3.93 (t, *J*=6.9 Hz, 2 H), 2.76 (t, *J*=6.8 Hz, 2 H); ^{13}C NMR (75 MHz, DMSO-d_6) δ ppm 169.1, 159.7, 159.2, 155.9, 140.3, 138.2, 137.2, 134.3, 134.2, 130.4, 129.9, 128.1, 127.7, 126.3, 118.0, 115.9, 115.2, 109.9, 41.2, 34.2; HRMS (ESI) m/z: 417.1263 found (calcd for $C_{24}H_{21}N_2O_3S^+$, $[M+H]^+$ 417.1267).

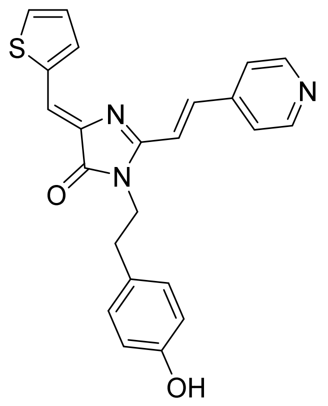

(*Z*)-5-(thiophen-2-ylmethylene)-3-(4-hydroxyphenethyl)-2-((*E*)-2-(pyridine-4-yl)vinyl)-3,5-dihydro-4*H*-imidazol-4-one (3cf)

Brown solid (378 mg, 94%); mp ~230 ºC with decomposition; ^{1}H NMR (300 MHz, DMSO-d_6) δ ppm 9.12 (s, 1 H), 8.65 (d, *J*=5.5 Hz, 2 H), 7.95 (d, *J*=4.9 Hz, 1 H), 7.69 - 7.78 (m, 2 H), 7.66 (d, *J*=5.5 Hz, 2 H), 7.44 (s, 1 H), 7.20 (dd, J=4.6, 4.1 Hz, 1 H), 7.10 (d, *J*=15.7 Hz, 1 H), 6.99 (d, *J*=8.1 Hz, 2 H), 6.60 (d, *J*=8.2 Hz, 2 H), 3.99 (t, *J*=6.2 Hz, 2 H), 2.76 (t, *J*=6.7 Hz, 2 H); ^{13}C NMR (201 MHz, DMSO-d_6) δ ppm 168.7, 158.0, 155.9, 150.1, 141.9, 137.8, 136.7, 136.4, 135.2, 135.0, 129.8, 127.9, 127.8, 121.8, 120.3, 118.2, 115.1, 41.2, 33.9; HRMS (ESI) m/z: 402.1267 found (calcd for $C_{23}H_{20}N_3O_2S^+$, $[M+H]^+$ 402.1271).

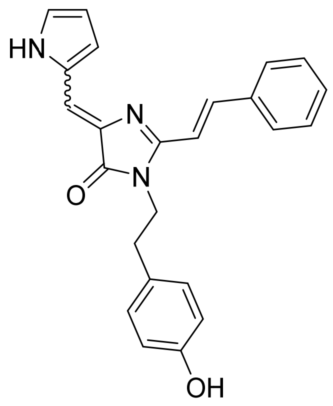

5-(1*H*-pyrrol-2-ylmethylene)-3-(4-hydroxyphenethyl)-2-((*E*)-styryl)-3,5-dihydro-4*H*-imidazol-4-one (4cc)

Dark-red solid (249 mg, 65%); mp ~180 ºC with decomposition.

It was obtained as a mixture of two inseparable isomers. ^{1}H NMR signals were assigned to individual isomers:

(*E*)-5-(1*H*-pyrrol-2-ylmethylene)-3-(4-hydroxyphenethyl)-2-((*E*)-styryl)-3,5-dihydro-4*H*-imidazol-4-one

^{1}H NMR (700 MHz, DMSO-d_6) δ ppm 12.99 (br. s., 1 H), 9.13 (s, 1 H), 7.69 (d, *J*=7.4 Hz, 2 H), 7.66 (d, *J*=15.6 Hz, 1 H), 7.42 - 7.47 (m, 3 H), 7.37 (br. s., 1 H), 7.29 (s, 1 H), 7.02 (d, *J*=8.4 Hz, 2 H), 6.89 (br. s., 1 H), 6.86 (d, *J*=15.8 Hz, 1 H), 6.60 - 6.66 (m, 2 H), 6.38 (ddd, *J*=3.6, 2.4, 2.2 Hz, 1 H), 4.04 (t, *J*=7.2 Hz, 2 H), 2.81 (t, *J*=7.1 Hz, 2 H).

(*Z*)-5-(1*H*-pyrrol-2-ylmethylene)-3-(4-hydroxyphenethyl)-2-((*E*)-styryl)-3,5-dihydro-4*H*-imidazol-4-one

^{1}H NMR (700 MHz, DMSO-d_6) δ ppm 11.39 (br. s., 1 H), 9.11 (s, 1 H), 8.05 (d, *J*=16.0 Hz, 1 H), 7.72 (d, *J*=7.2 Hz, 2 H), 7.42 - 7.47 (m, 1 H), 7.39 (d, *J*=7.2 Hz, 2 H), 7.24 (br. s., 1 H), 7.00 (d, *J*=8.6 Hz, 2 H) 6.93 (s, 1 H), 6.89 (br. s., 1 H), 6.85 (d, J=15.8 Hz, 1 H), 6.60 - 6.66 (m, 2 H), 6.26 - 6.30 (m, 1 H), 3.95 (t, *J*=7.1 Hz, 2 H), 2.76 (t, *J*=6.9 Hz, 2 H).

^{13}C and HRMS data presented as is for a mixture:

^{13}C NMR (201 MHz, DMSO-d_6) δ ppm 168.6, 167.9, 156.1, 155.9, 155.8, 152.9, 138.8, 136.8, 135.4 (2 C), 133.5, 133.3, 129.7 (2 C), 129.5, 129.3, 129.1, 128.8, 128.7 (2 C), 128.2, 128.1, 127.9, 127.7, 126.4, 126.0, 125.1, 120.7, 118.7, 115.7, 115.1 (2 C), 113.7, 113.5, 111.8, 110.8, 41.5, 41.0, 34.1, 33.9; HRMS (ESI) m/z: 384.1701 found (calcd for $C_{24}H_{22}N_3O_2^+$, $[M+H]^+$ 384.1707).

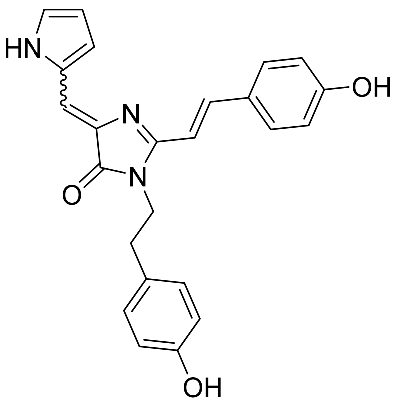

5-(1*H*-pyrrol-2-ylmethylene)-3-(4-hydroxyphenethyl)-2-((*E*)-4-hydroxystyryl)-3,5-dihydro-4*H*-imidazol-4-one (4cd)

Dark-red solid (205 mg, 51%); mp ~250 ºC with decomposition.

It was obtained as a mixture of two inseparable isomers. ^{1}H NMR signals were assigned to individual isomers:

(*E*)-5-(1*H*-pyrrol-2-ylmethylene)-3-(4-hydroxyphenethyl)-2-((*E*)-4-hydroxystyryl)-3,5-dihydro-4*H*-imidazol-4-one

^{1}H NMR (700 MHz, DMSO-d_6) δ ppm 12.95 (br. s., 1 H), 9.89 (s, 1 H), 9.14 (s, 1 H), 7.56 - 7.61 (m, 1 H), 7.53 (d, *J*=8.6 Hz, 2 H), 7.33 (br. s., 1 H), 7.22 (s, 1 H), 7.02 (d, J=8.4 Hz, 2 H), 6.80 - 6.85 (m, 3 H), 6.62 - 6.66 (m, 3 H), 6.35 (ddd, *J*=3.6, 2.3, 2.1 Hz, 1 H), 4.00 (t, *J*=7.2 Hz, 2 H), 2.79 (t, *J*=7.2 Hz, 2 H).

(*Z*)-5-(1*H*-pyrrol-2-ylmethylene)-3-(4-hydroxyphenethyl)-2-((*E*)-4-hydroxystyryl)-3,5-dihydro-4*H*-imidazol-4-one

^{1}H NMR (700 MHz, DMSO-d_6) δ ppm 11.35 (br. s., 1 H), 9.95 (s, 1 H), 9.12 (s, 1 H), 7.99 (d, *J*=15.6 Hz, 1 H), 7.56 - 7.61 (m, 2 H), 7.21 (br. s., 1 H), 7.00 (d, J=8.6 Hz, 2 H), 6.86 (s, 1 H), 6.80 - 6.85 (m, 3 H), 6.62 - 6.66 (m, 3 H), 6.26 (ddd, *J*=3.5, 2.3, 2.1 Hz, 1 H), 3.92 (t, *J*=7.1 Hz, 2 H), 2.75 (t, *J*=7.0 Hz, 2 H).

^{13}C and HRMS data presented as is for a mixture:

^{13}C NMR (201 MHz, DMSO-d_6) δ ppm 168.7, 168.0, 159.2, 159.0, 156.6, 155.8 (2 C), 153.4, 139.3, 137.2, 133.7, 133.5, 129.8, 129.7, 129.5, 129.1, 128.8, 128.7, 128.1, 128.0, 126.6, 125.7, 125.5, 124.1, 120.1, 118.1, 115.7, 115.6, 115.5, 115.1 (2 C), 114.7, 111.6, 110.6, 110.0, 109.7, 41.4, 40.9, 34.1, 33.9; HRMS (ESI) m/z: 400.1651 found (calcd for $C_{24}H_{22}N_3O_3^+$, $[M+H]^+$ 400.1656).

7. Structure confirmation

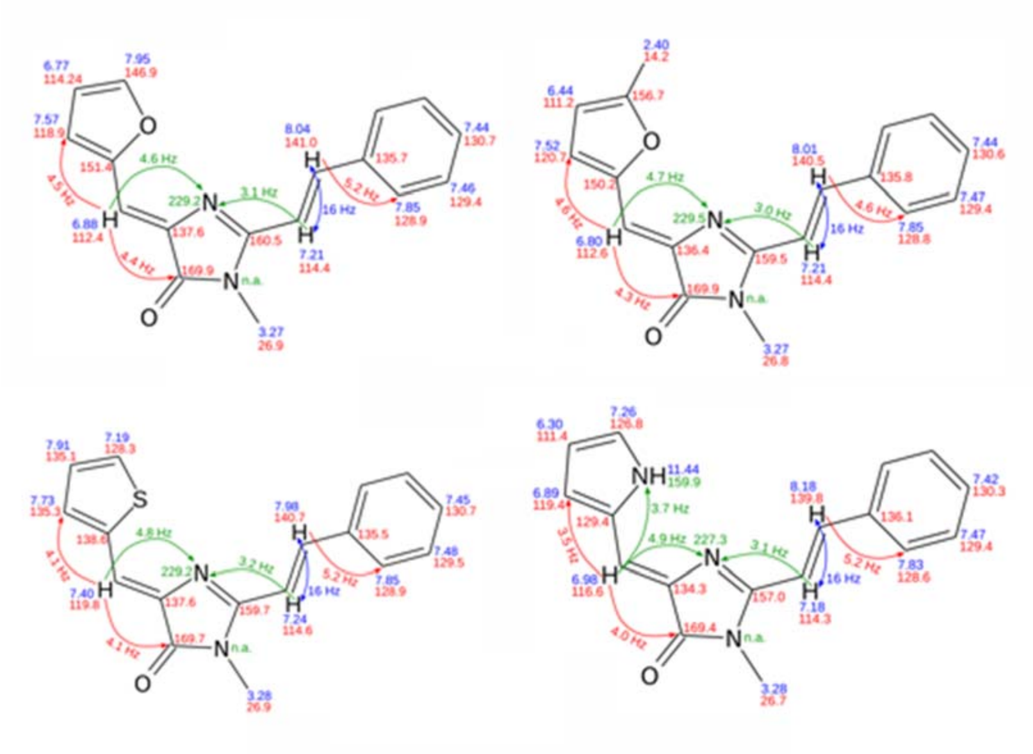

Fig S7.1. Results of the investigation of the structure of compounds **1ac**, **2ac**, **3ac**, and **4ac** by heteronuclear NMR spectroscopy. Chemical shifts of ^{1}H (in blue), ^{13}C (in red), and ^{15}N (in green) are shown. Key spin-spin coupling interactions are denoted with arrows.

8. Copies of ^1H and ^{13}C NMR spectra

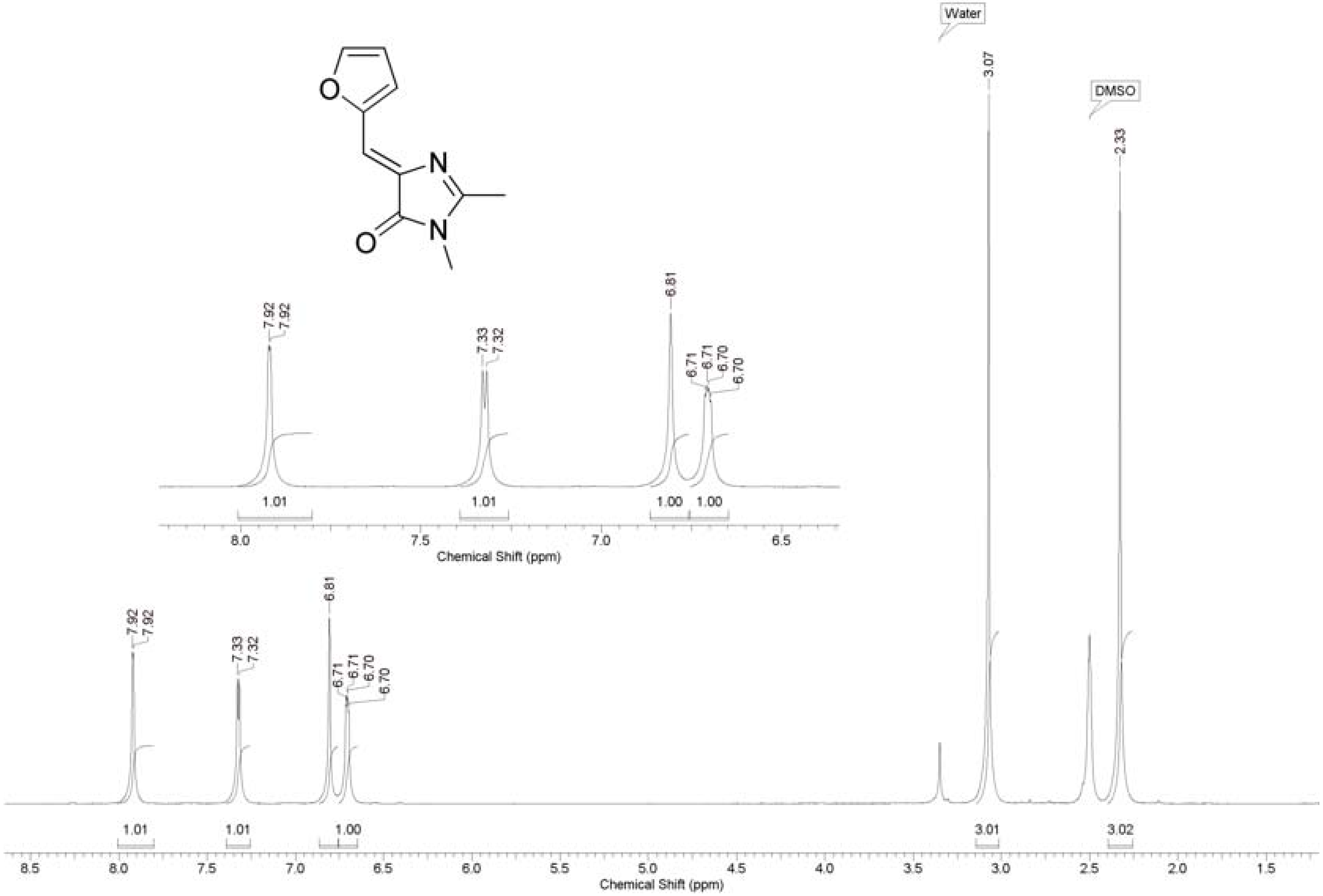
O
N
N
O
Water
3.07
DMSO
2.33
7.92
7.92
7.33
7.32
6.81
6.71
6.71
6.70
6.70
1.01
1.01
1.00
1.00
8.0
7.5
7.0
6.5
Chemical Shift (ppm)
3.01
3.02
8.5
6.0
5.5
5.0
4.5
4.0
3.5
3.0
2.5
2.0
1.5

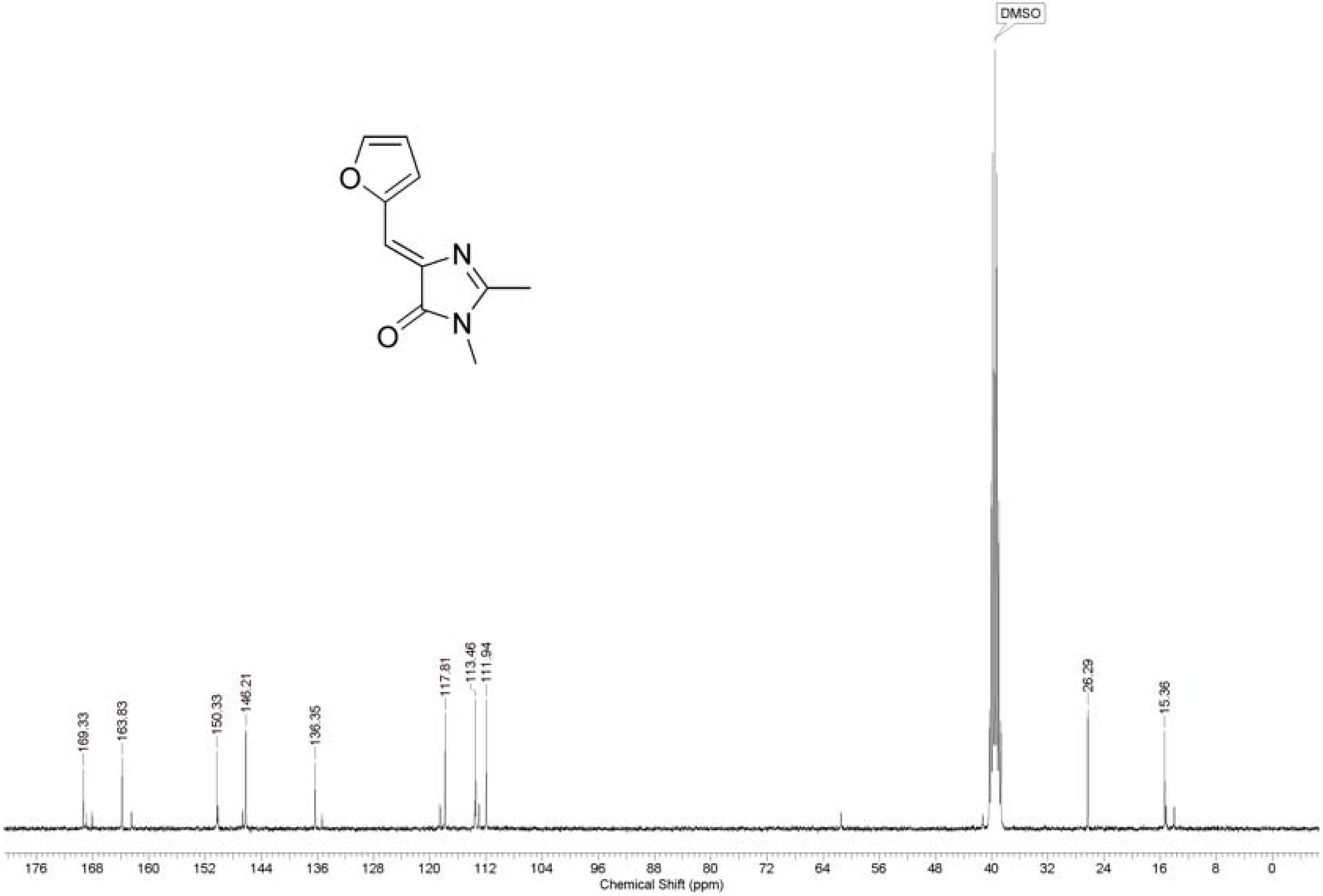
DMSO
169.33
163.83
150.33
146.21
136.35
117.81
113.46
111.94
26.29
15.36
176 168 160 152 144 136 128 120 112 104 96 88 80 72 64 56 48 40 32 24 16 8 0
Chemical Shift (ppm)

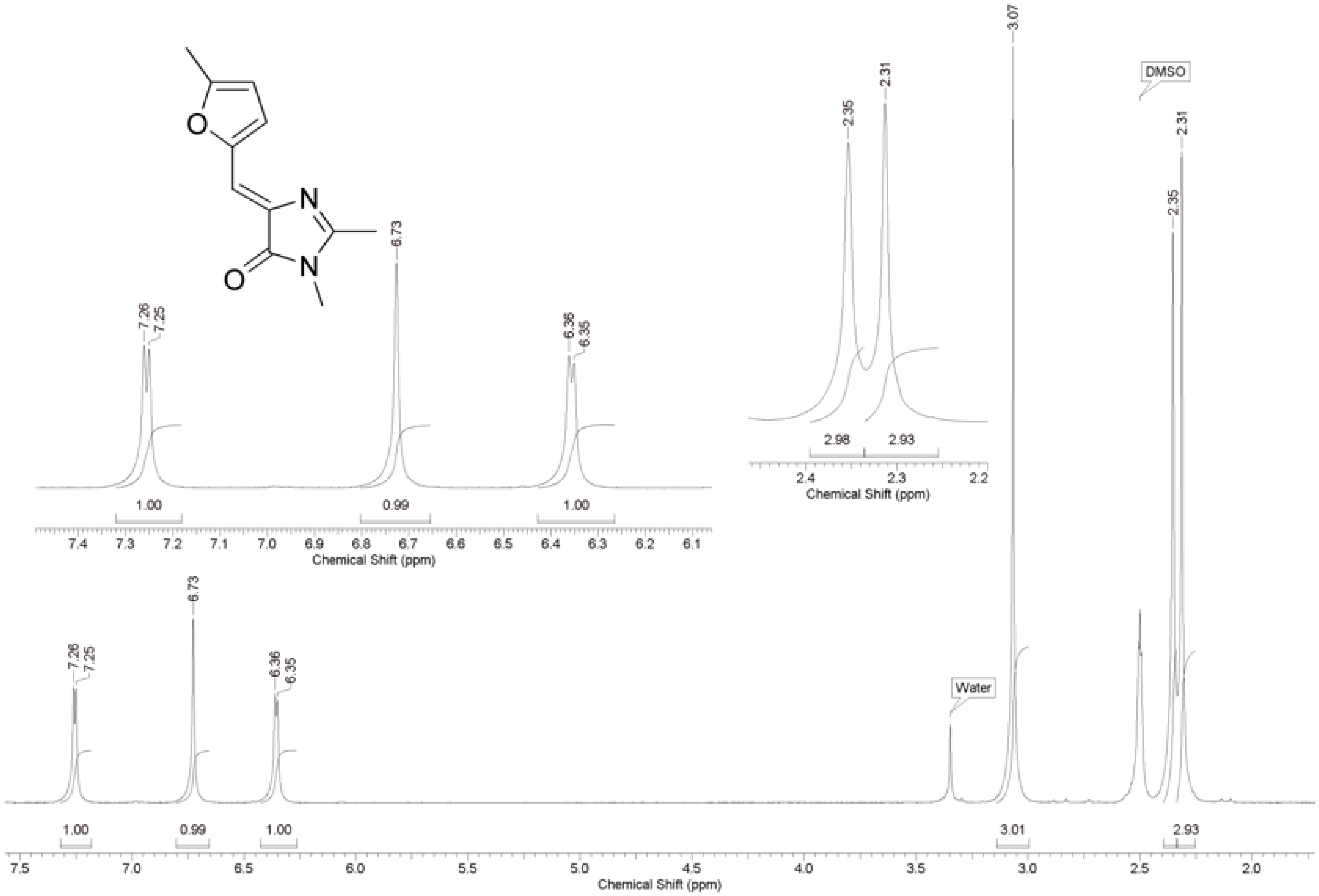

O
N
N
O
7.26
7.25
6.73
6.36
6.35
1.00
0.99
1.00
7.4 7.3 7.2 7.1 7.0 6.9 6.8 6.7 6.6 6.5 6.4 6.3 6.2 6.1
Chemical Shift (ppm)
2.35
2.31
2.98
2.93
2.4 2.3 2.2
Chemical Shift (ppm)
3.07
DMSO
2.31
2.35
Water
3.01
2.93
7.5 7.0 6.5 6.0 5.5 5.0 4.5 4.0 3.5 3.0 2.5 2.0
Chemical Shift (ppm)

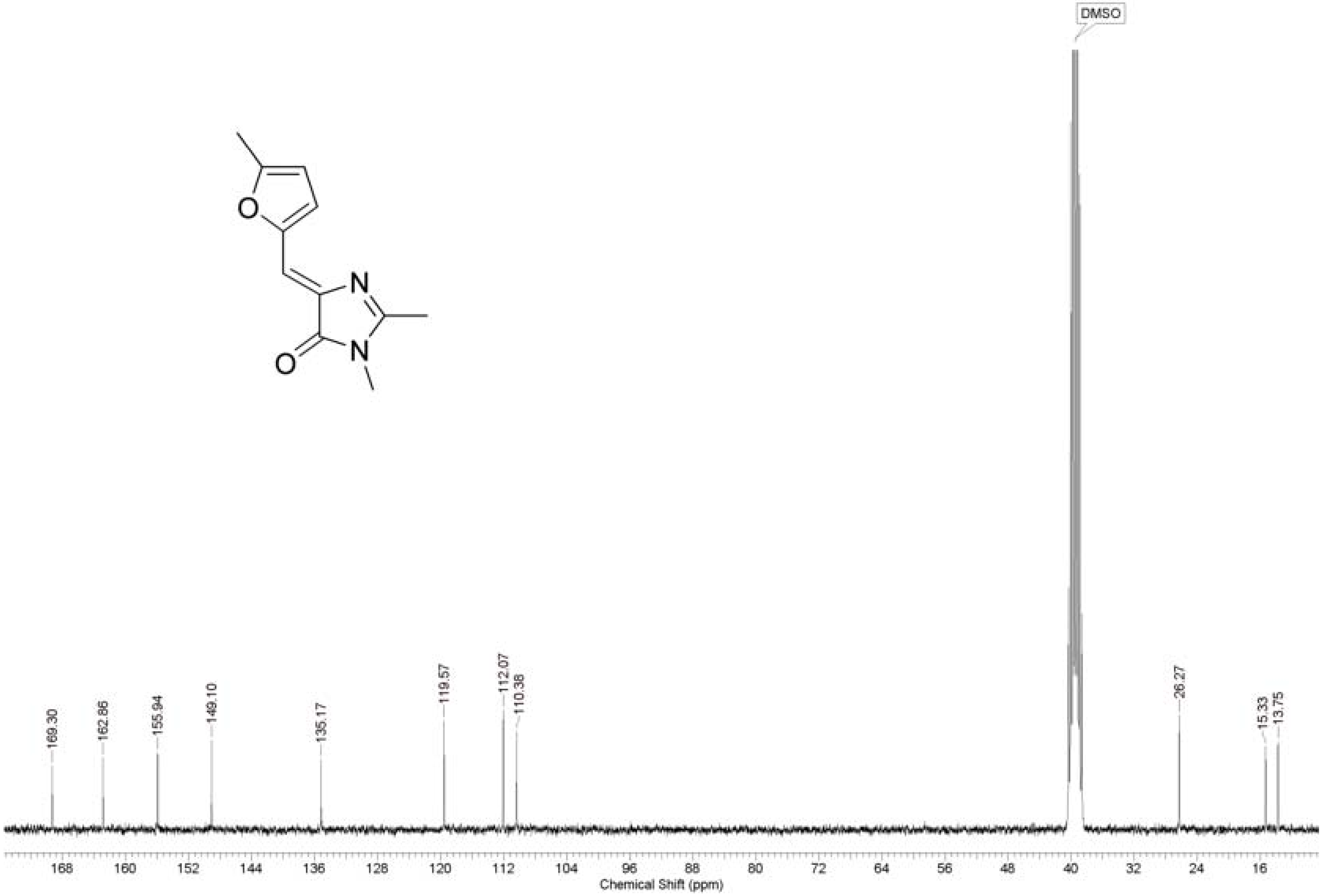
DMSO
169.30
162.86
155.94
149.10
135.17
119.57
112.07
110.38
26.27
15.33
13.75
168
160
152
144
136
128
120
112
104
96
88
80
72
64
56
48
40
32
24
16
Chemical Shift (ppm)

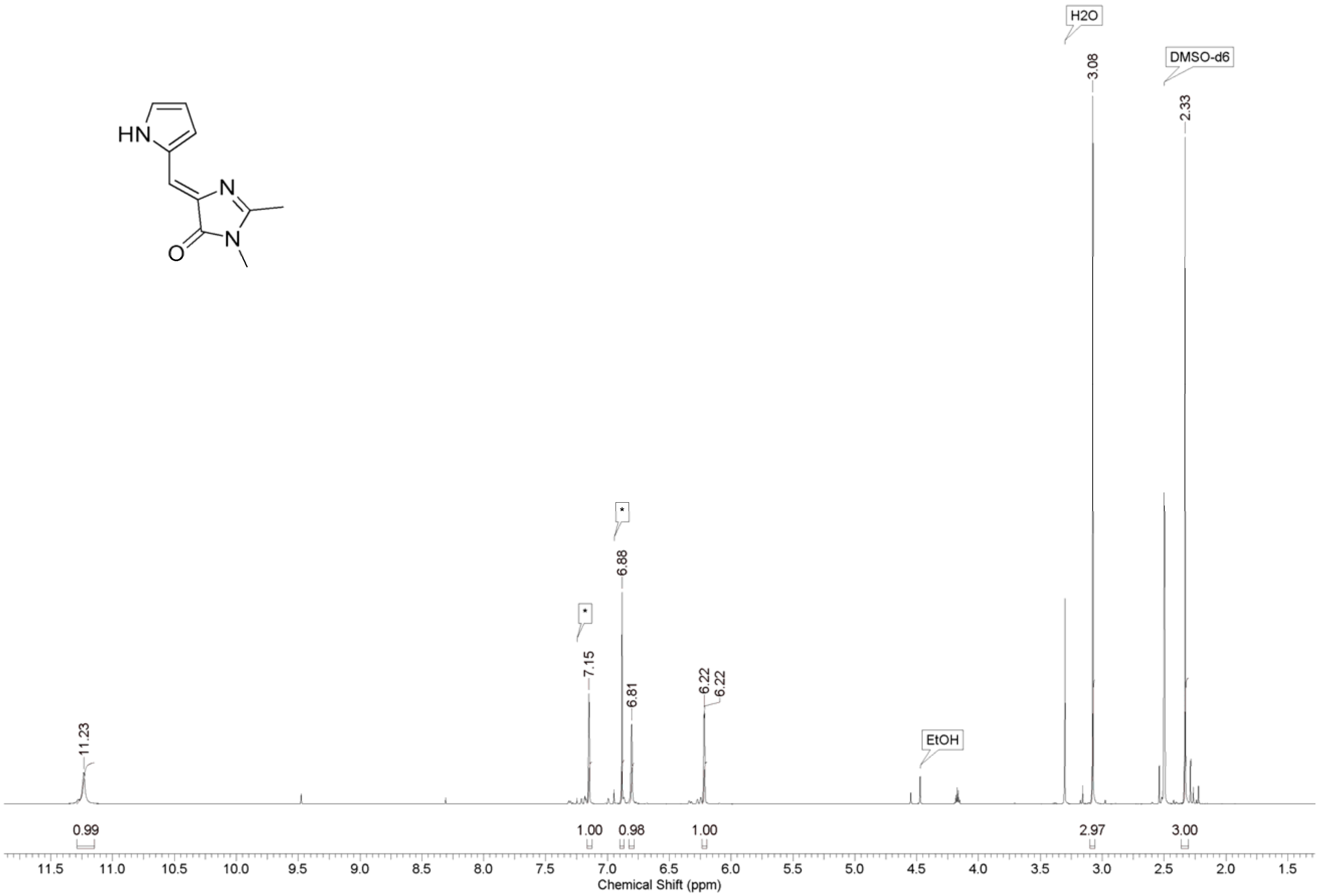
HN
N
N
O
H2O
DMSO-d6
EtOH
*
*
11.23
7.15
6.88
6.81
6.22
6.22
3.08
2.33
0.99
1.00
0.98
1.00
2.97
3.00
Chemical Shift (ppm)

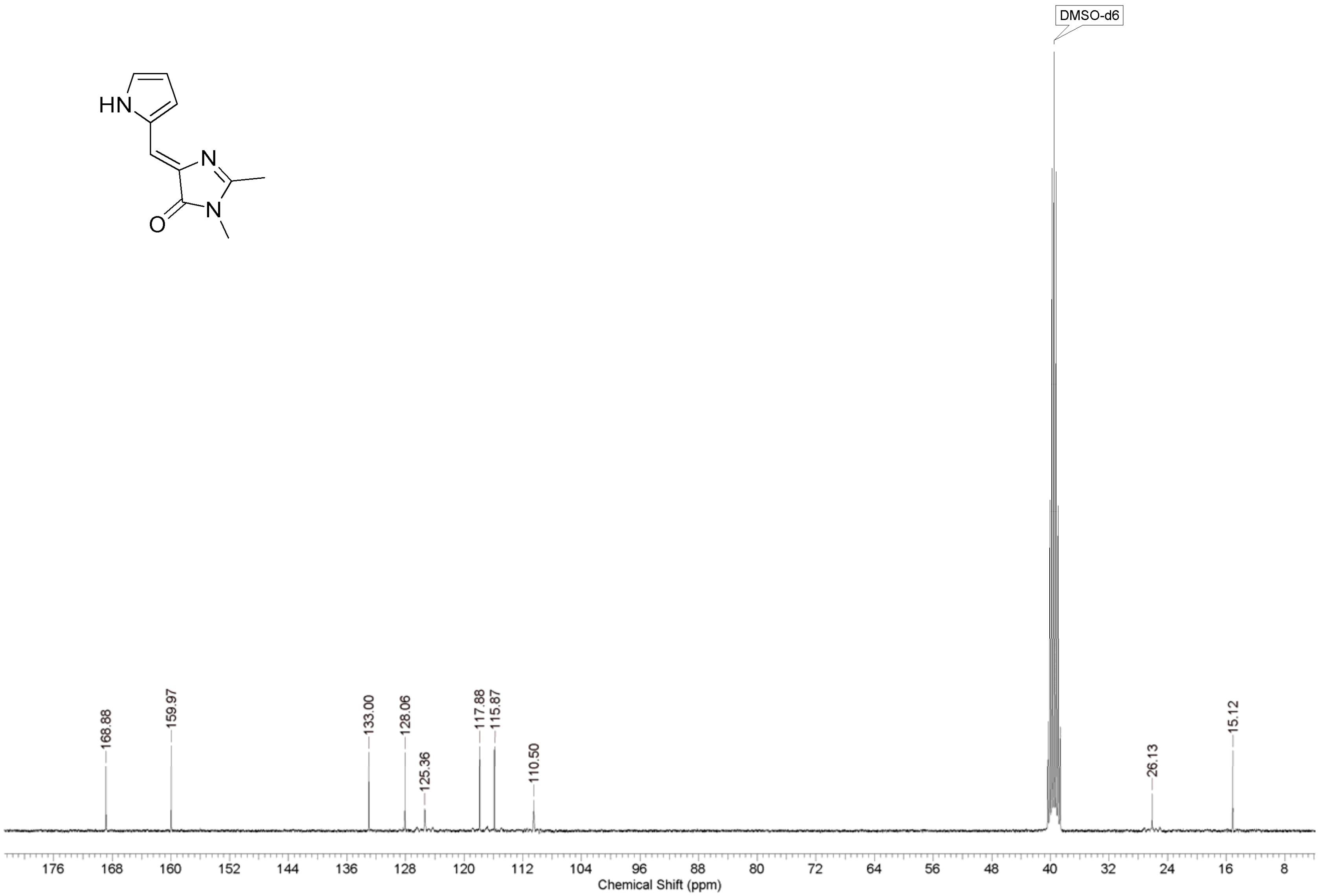
HN
N
N
O
DMSO-d6
168.88
159.97
133.00
128.06
125.36
117.88
115.87
110.50
26.13
15.12
176
168
160
152
144
136
128
120
112
104
96
88
80
72
64
56
48
40
32
24
16
8
Chemical Shift (ppm)

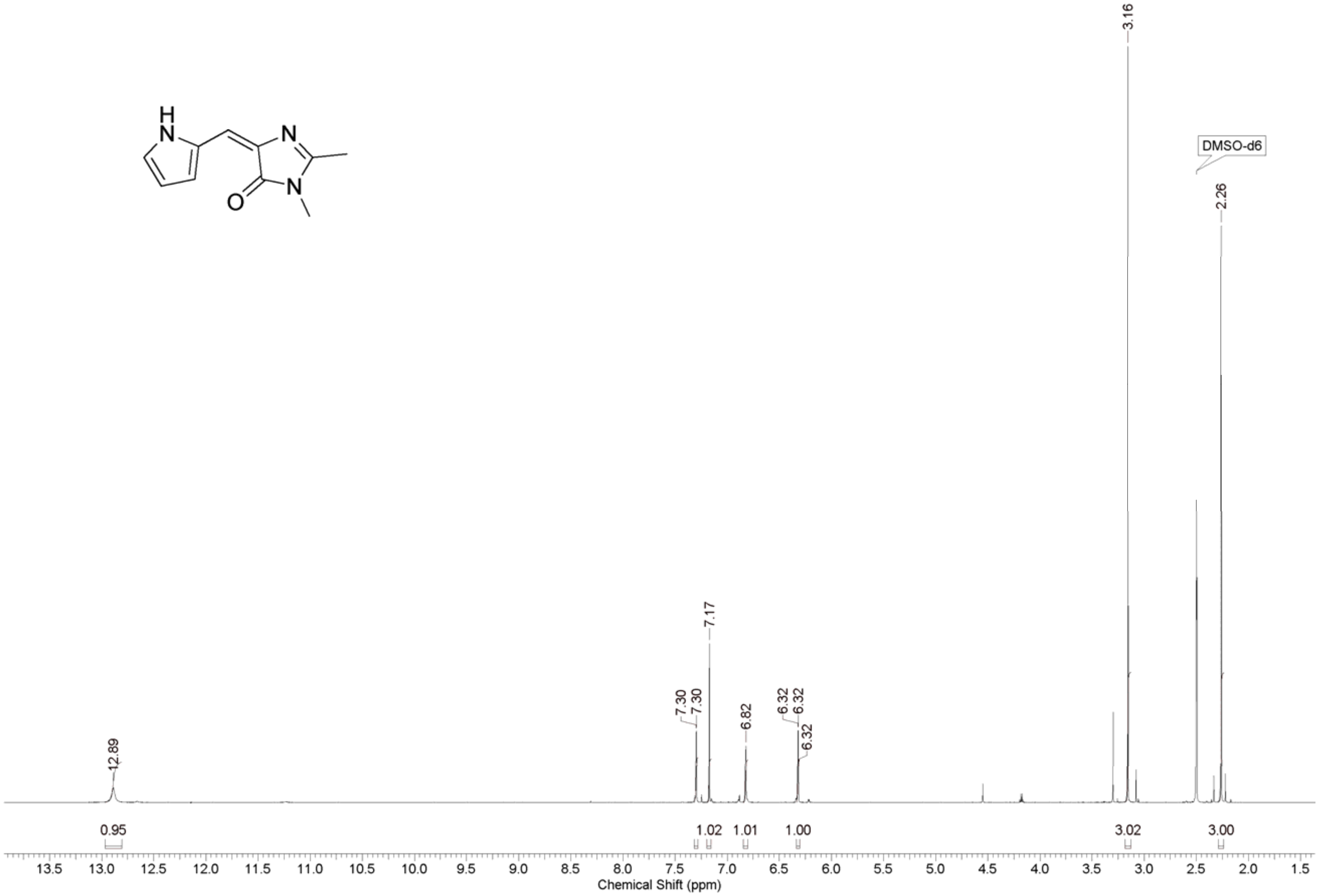

H
N
N
O
N
DMSO-d6
3.16
2.26
7.17
7.30
7.30
6.82
6.32
6.32
6.32
12.89
0.95
1.02
1.01
1.00
3.02
3.00
13.5
13.0
12.5
12.0
11.5
11.0
10.5
10.0
9.5
9.0
8.5
8.0
7.5
7.0
6.5
6.0
5.5
5.0
4.5
4.0
3.5
3.0
2.5
2.0
1.5
Chemical Shift (ppm)

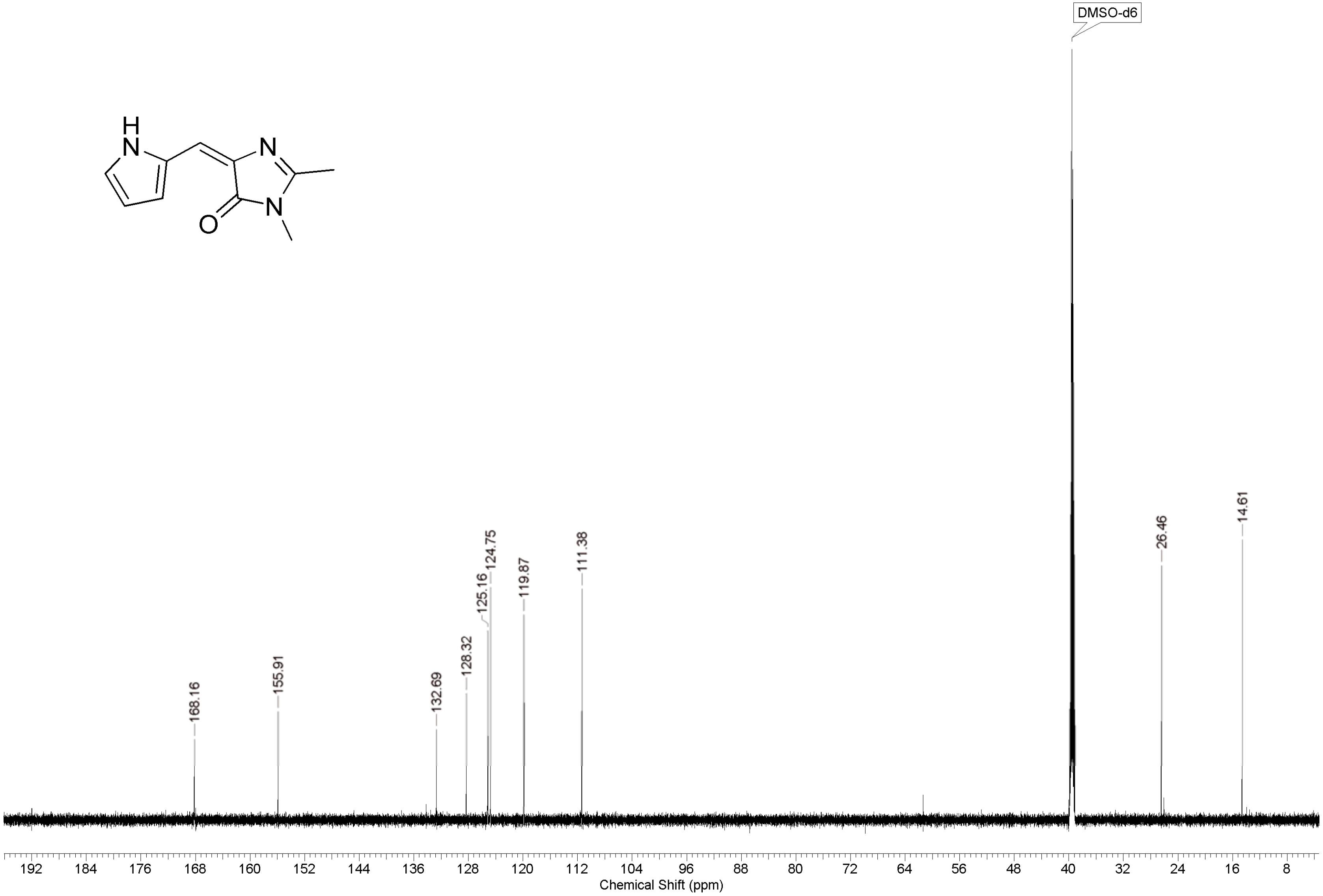
H
N
N
N
O
DMSO-d6
168.16
155.91
132.69
128.32
125.16
124.75
119.87
111.38
26.46
14.61
192 184 176 168 160 152 144 136 128 120 112 104 96 88 80 72 64 56 48 40 32 24 16 8
Chemical Shift (ppm)

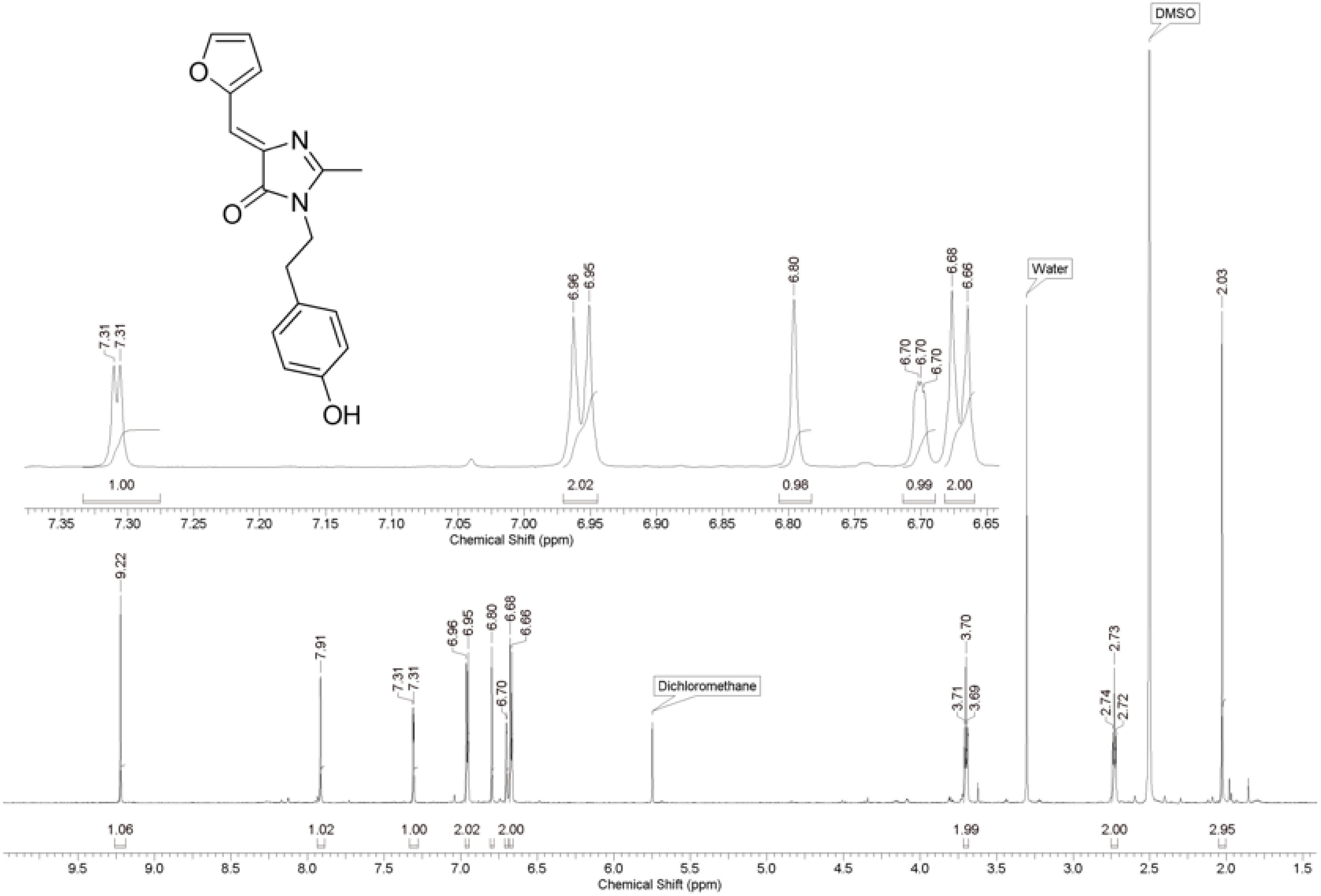
DMSO
Water
Dichloromethane
OH
N
O
Chemical Shift (ppm)
Chemical Shift (ppm)

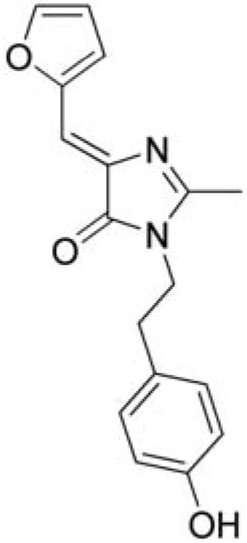

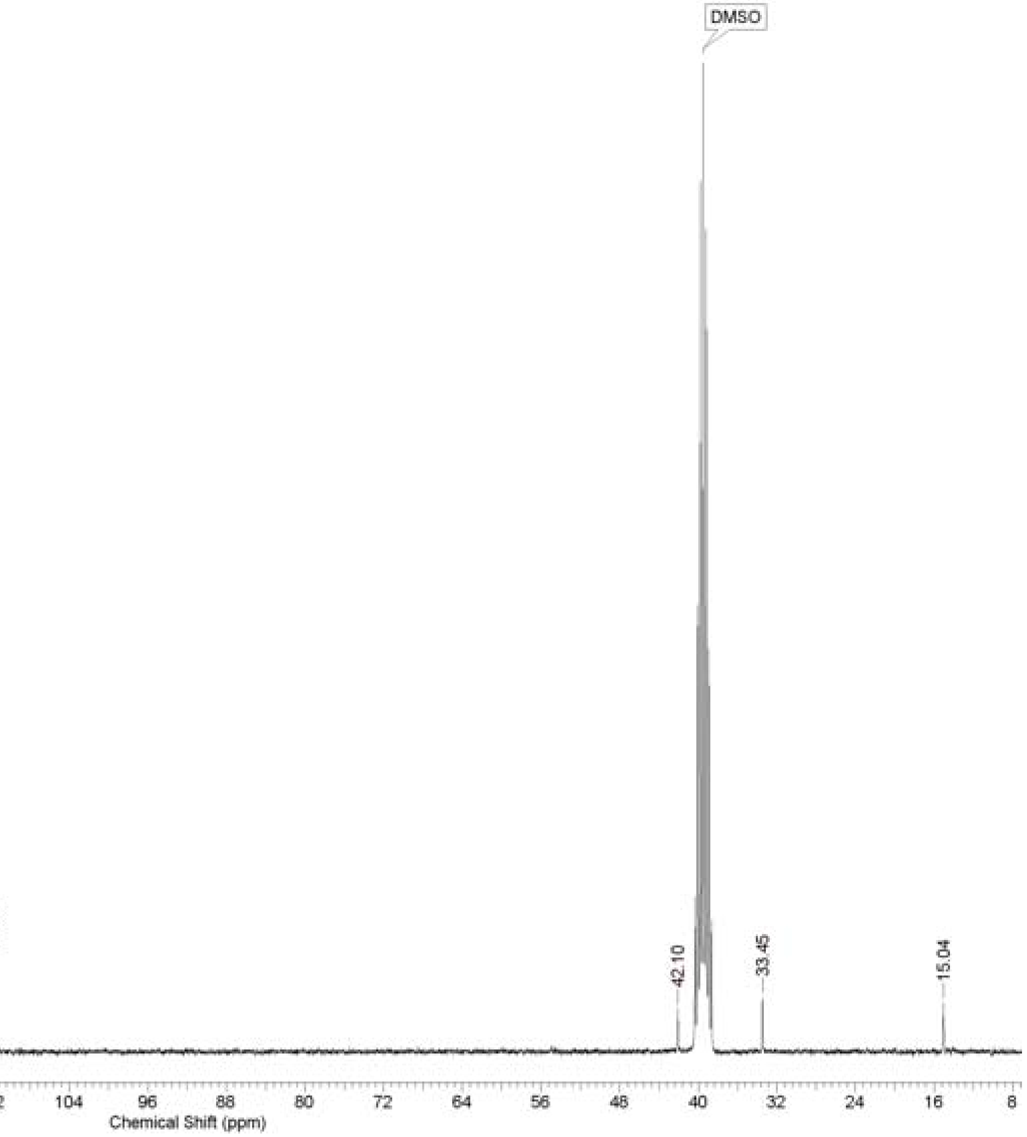
DMSO
169.22
163.30
155.97
150.28
146.26
135.96
129.84
128.23
117.92
115.27
113.44
111.96
42.10
33.45
15.04
168
160
152
144
136
128
120
112
104
96
88
80
72
64
56
48
40
32
24
16
8
Chemical Shift (ppm)

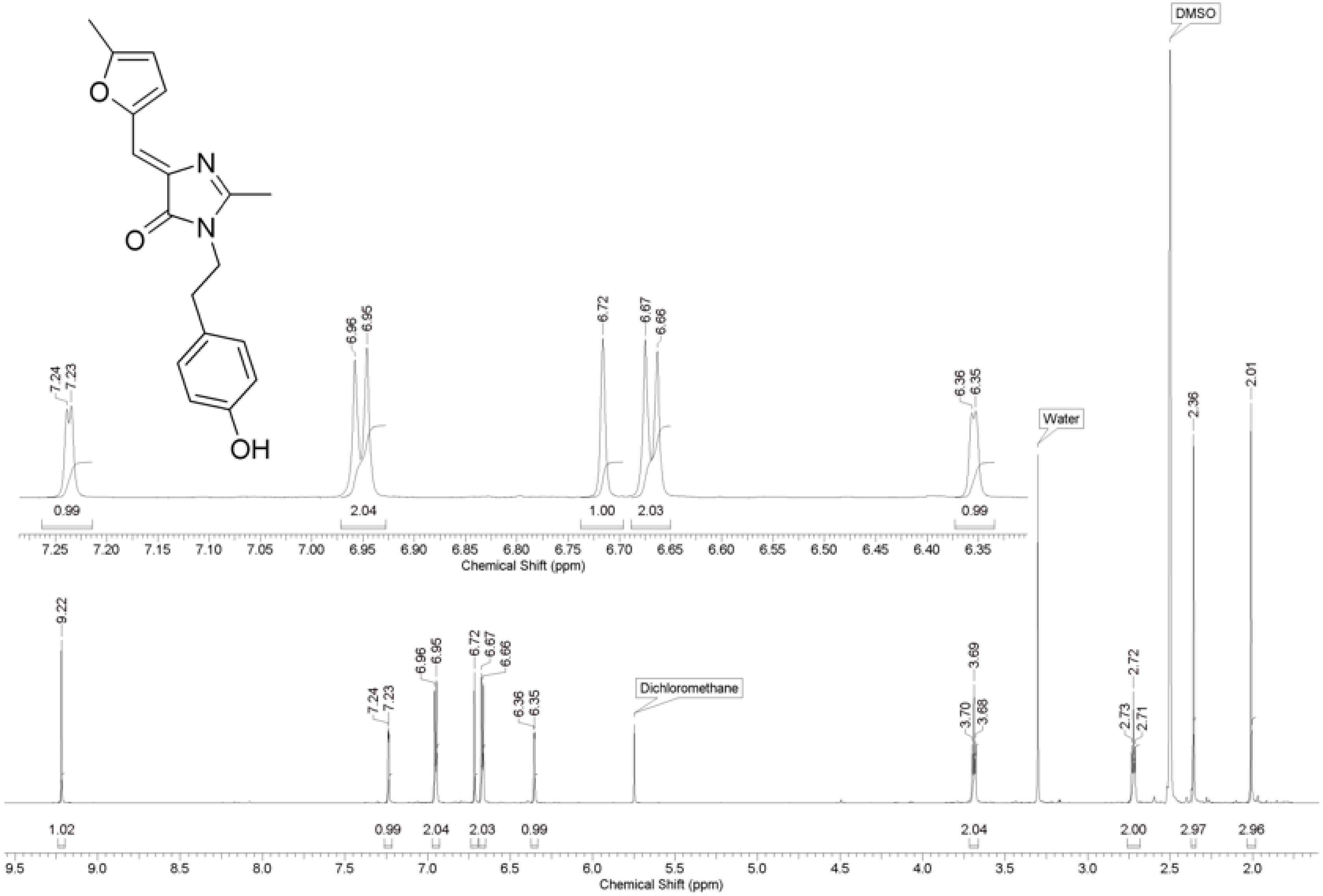
N
O
OH
DMSO
Water
Dichloromethane
Chemical Shift (ppm)
Chemical Shift (ppm)
9.22
7.24
7.23
6.96
6.95
6.72
6.67
6.66
6.36
6.35
3.70
3.69
3.68
2.73
2.72
2.71
2.36
2.01
1.02
0.99
2.04
2.03
1.00
2.00
2.97
2.96

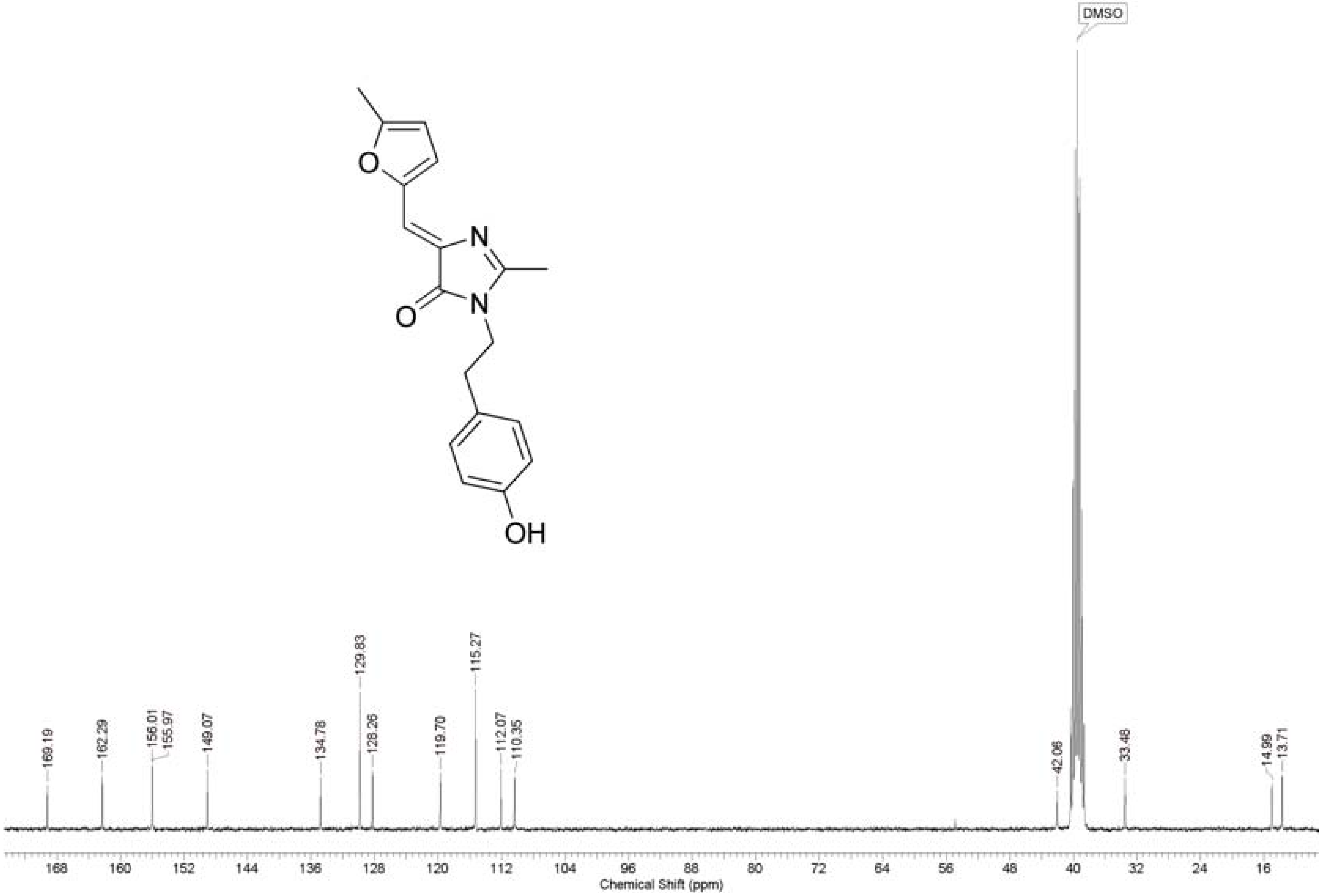
O
N
N
O
OH
DMSO
169.19
162.29
156.01
155.97
149.07
134.78
129.83
128.26
119.70
115.27
112.07
110.35
42.06
33.48
14.99
13.71
168 160 152 144 136 128 120 112 104 96 88 80 72 64 56 48 40 32 24 16
Chemical Shift (ppm)

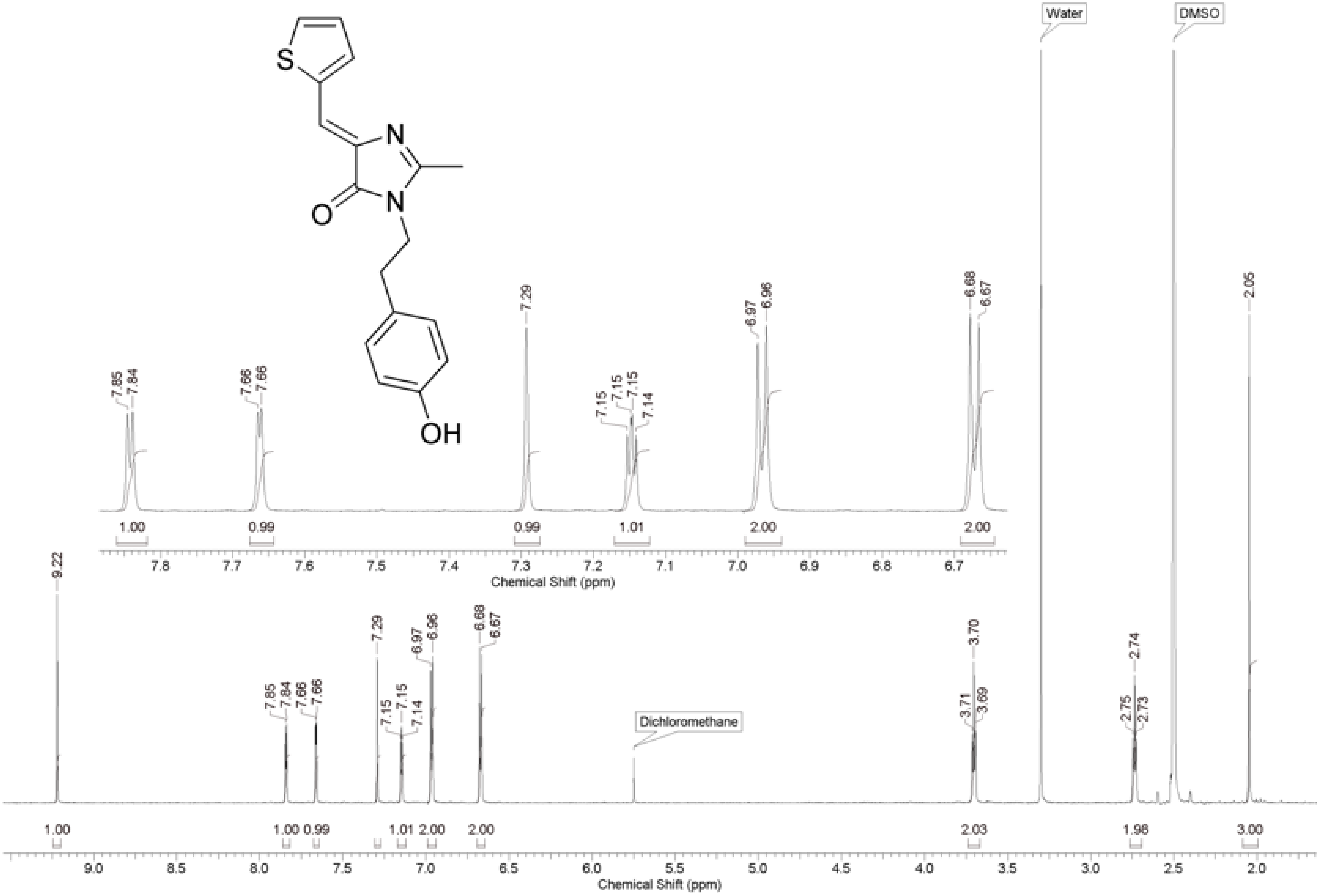

S
N
N
O
OH
Water
DMSO
Dichloromethane
9.22
7.85
7.84
7.66
7.66
7.29
7.15
7.15
7.15
7.14
6.97
6.96
6.68
6.67
3.71
3.70
3.69
2.75
2.74
2.73
2.05
1.00
1.00
0.99
0.99
1.01
2.00
2.00
2.03
1.98
3.00
Chemical Shift (ppm)

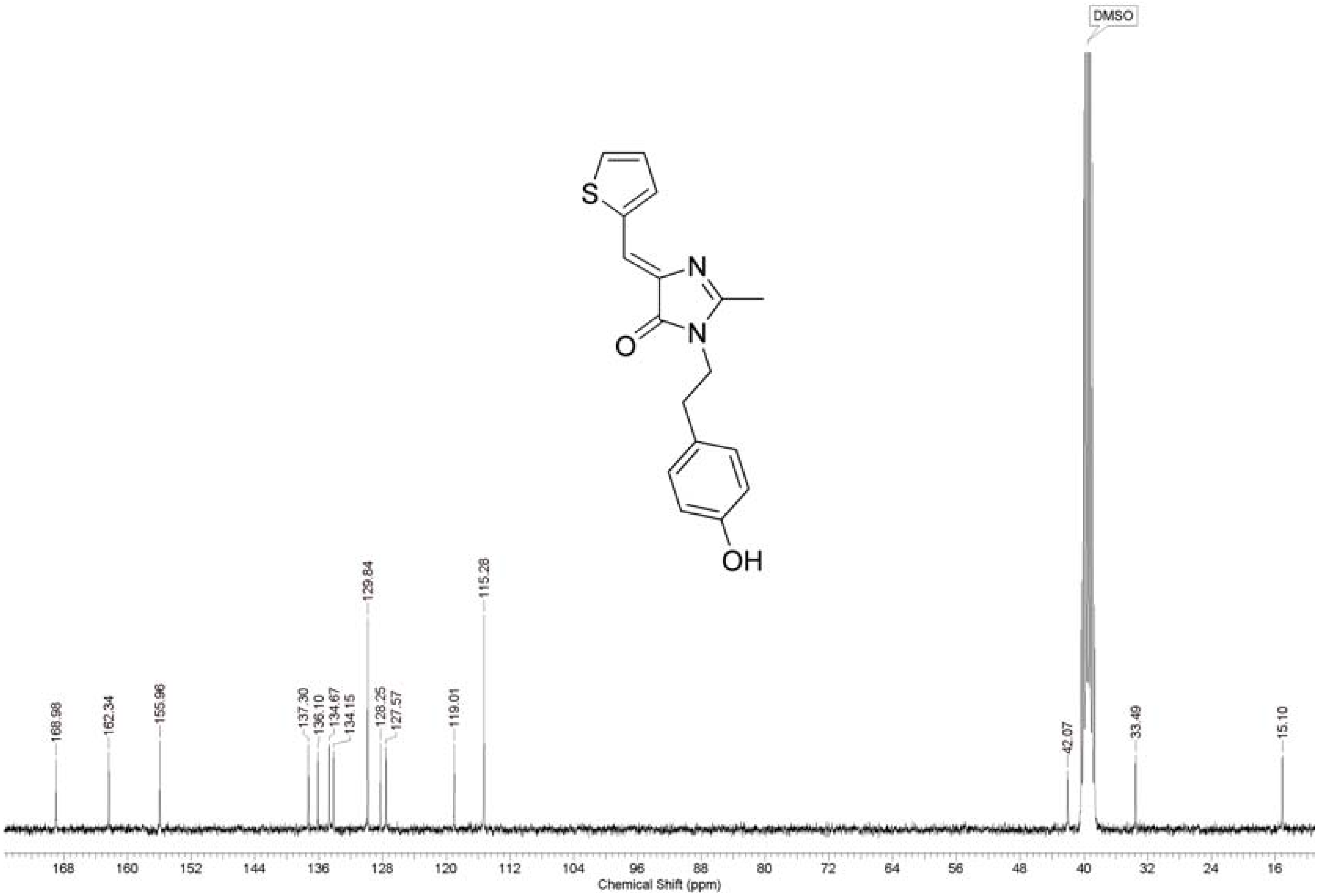
DMSO
168.98
162.34
155.96
137.30
136.10
134.67
134.15
129.84
128.25
127.57
119.01
115.28
42.07
33.49
15.10
S
N
N
O
OH
168
160
152
144
136
128
120
112
104
96
88
80
72
64
56
48
40
32
24
16
Chemical Shift (ppm)

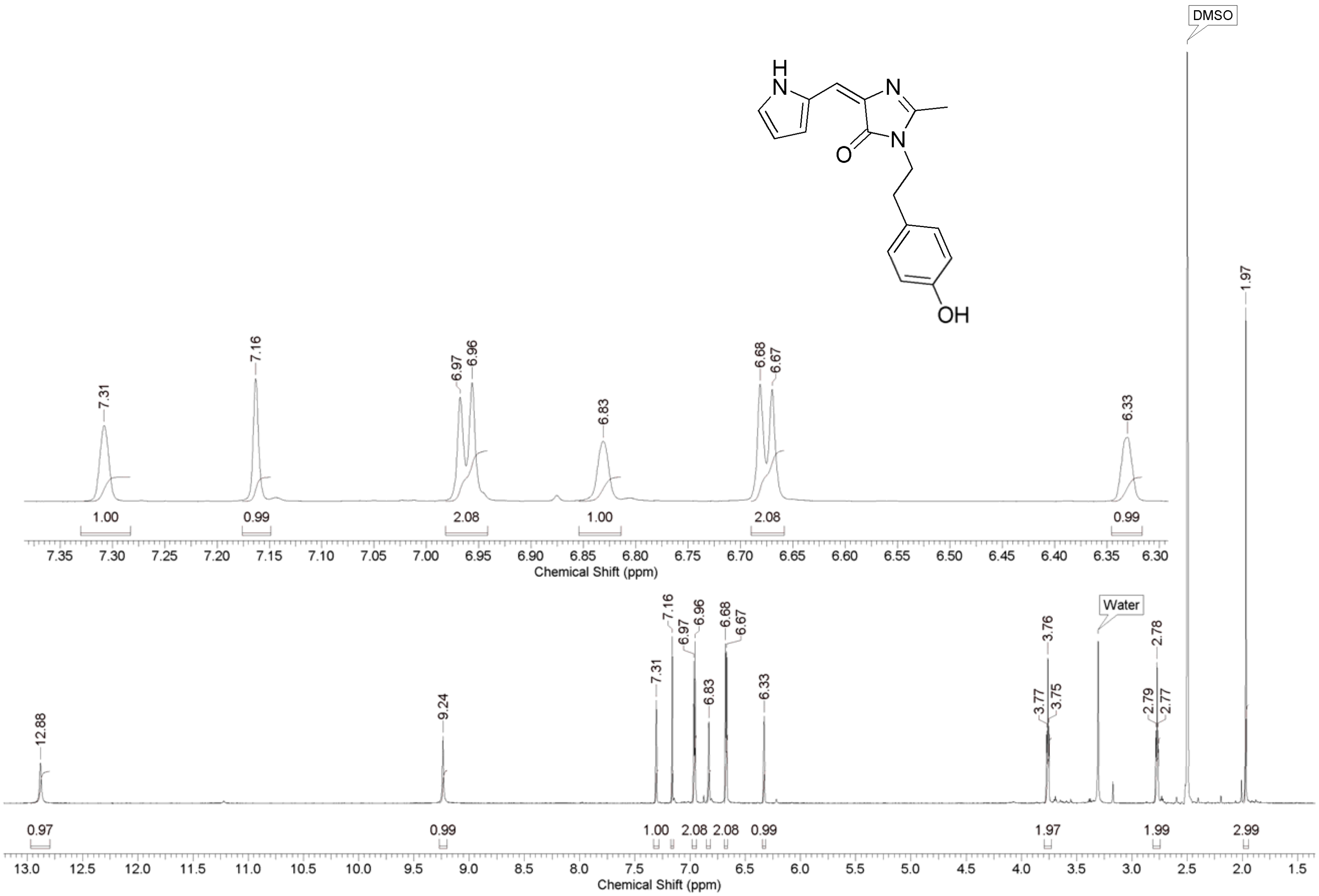
DMSO
H
N
N
O
OH
7.31
7.16
6.97
6.96
6.83
6.68
6.67
6.33
1.00 0.99 2.08 1.00 2.08 0.99
Chemical Shift (ppm)
Water
12.88
9.24
3.77
3.76
3.75
2.79
2.78
2.77
1.97
0.97 0.99 1.00 2.08 2.08 0.99 1.97 1.99 2.99
Chemical Shift (ppm)

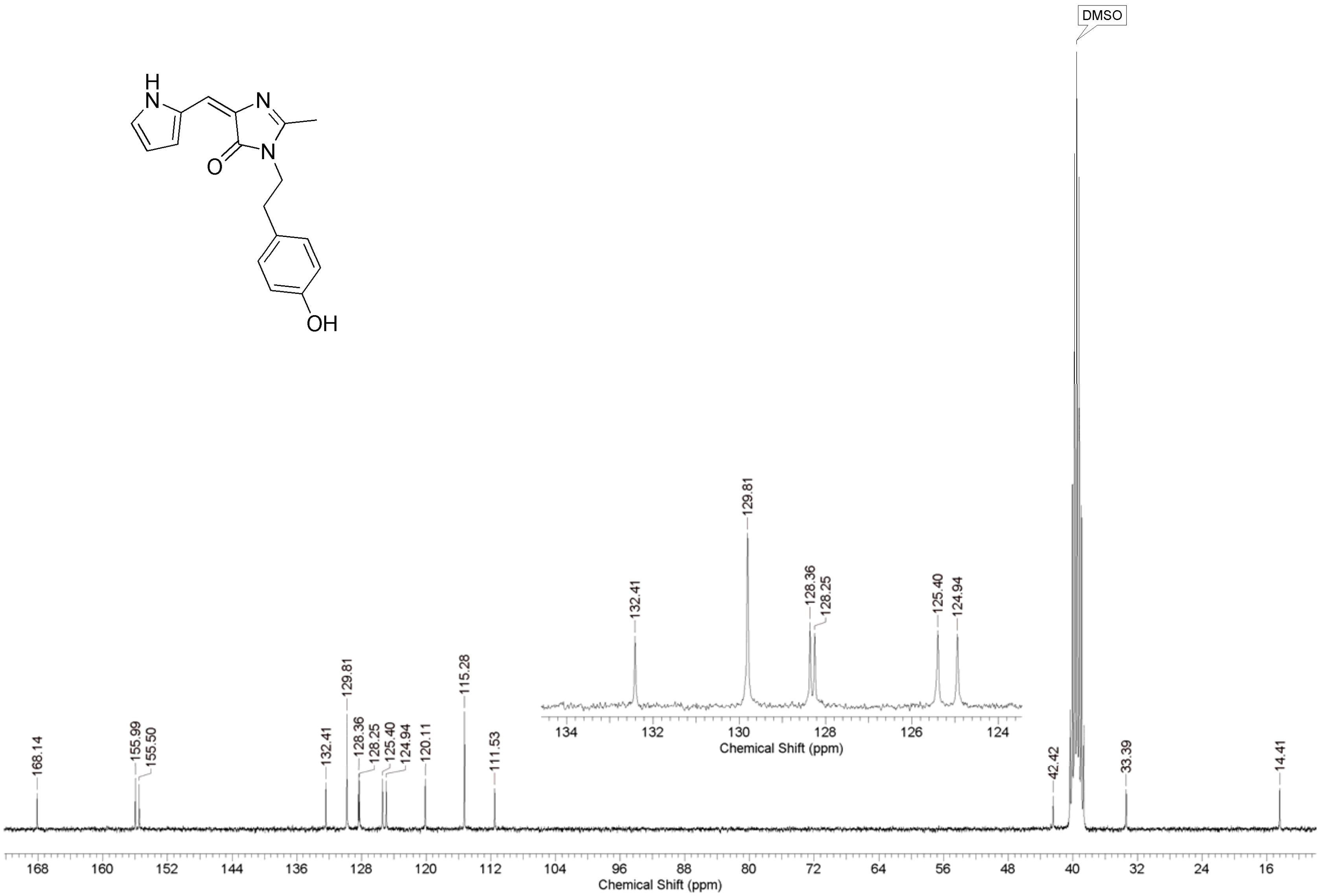
DMSO
168.14
155.99
155.50
132.41
129.81
128.36
128.25
125.40
124.94
120.11
115.28
111.53
42.42
33.39
14.41
Chemical Shift (ppm)
Chemical Shift (ppm)

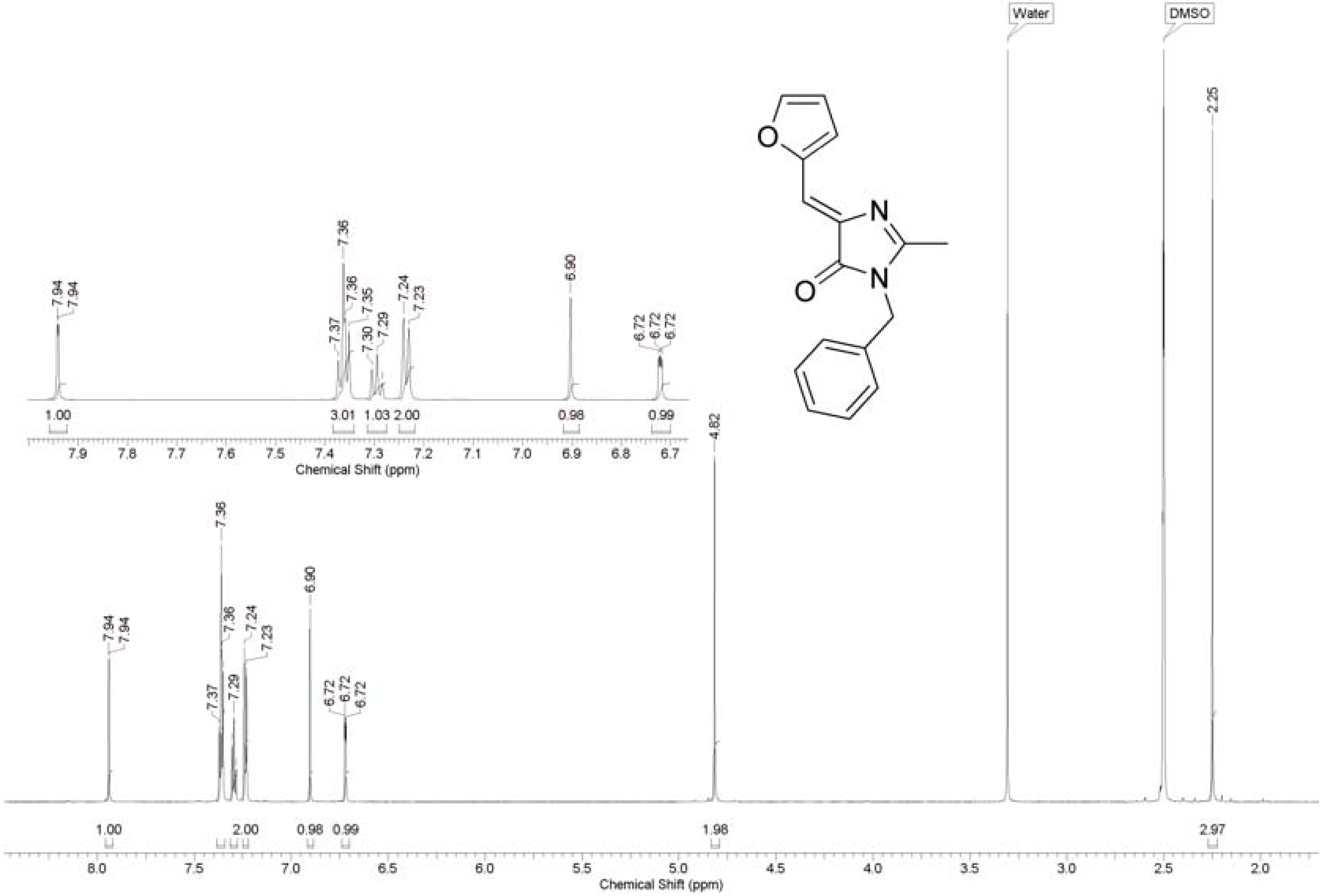
Water
DMSO
2.25
4.82
7.94
7.94
7.37
7.36
7.36
7.35
7.30
7.29
7.24
7.23
6.90
6.72
6.72
6.72
1.00
3.01
1.03
2.00
0.98
0.99
1.98
2.97
Chemical Shift (ppm)
O
N
N
O

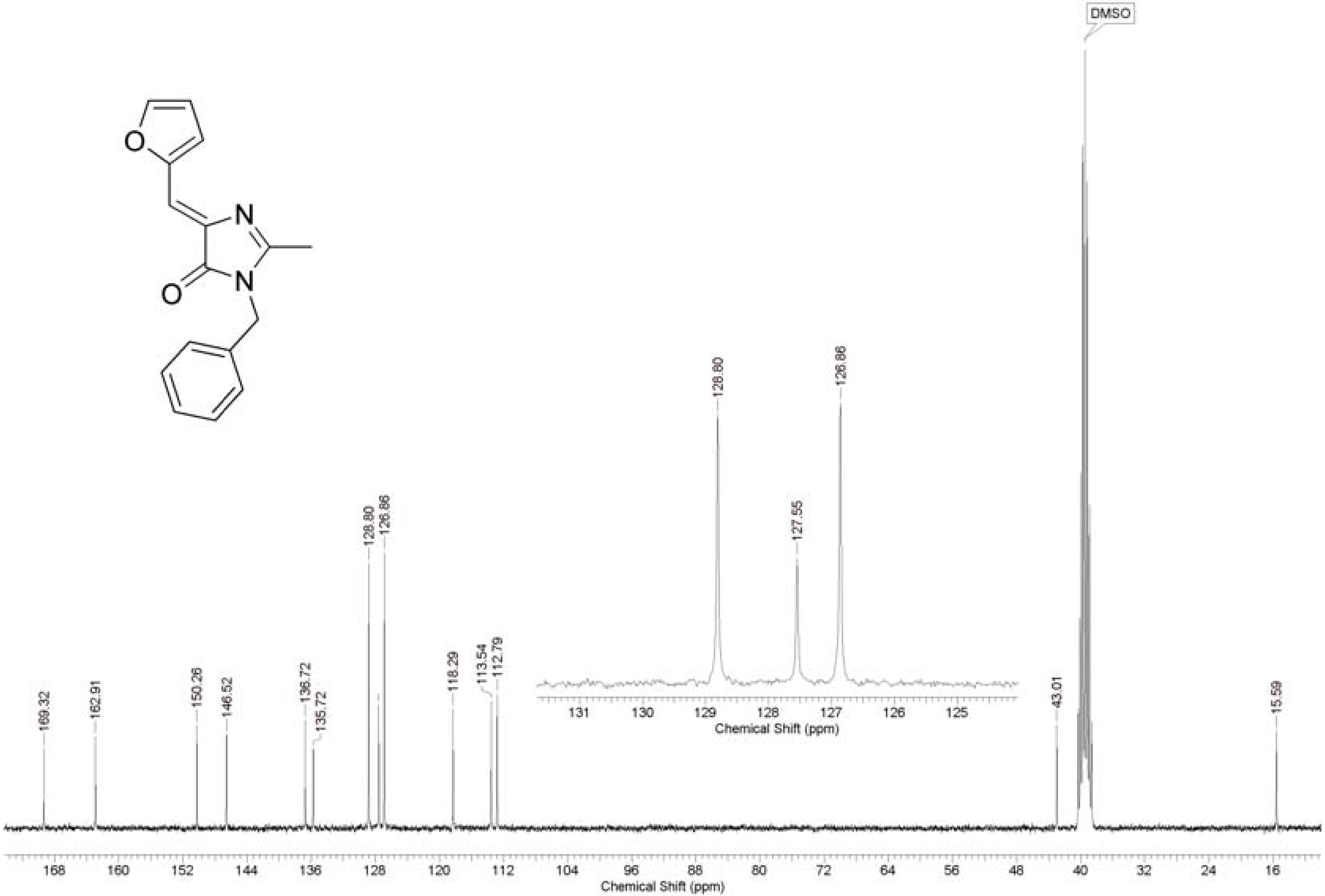
O
N
N
O
DMSO
169.32
162.91
150.26
146.52
136.72
135.72
128.80
126.86
118.29
113.54
112.79
43.01
15.59
128.80
127.55
126.86
Chemical Shift (ppm)
Chemical Shift (ppm)

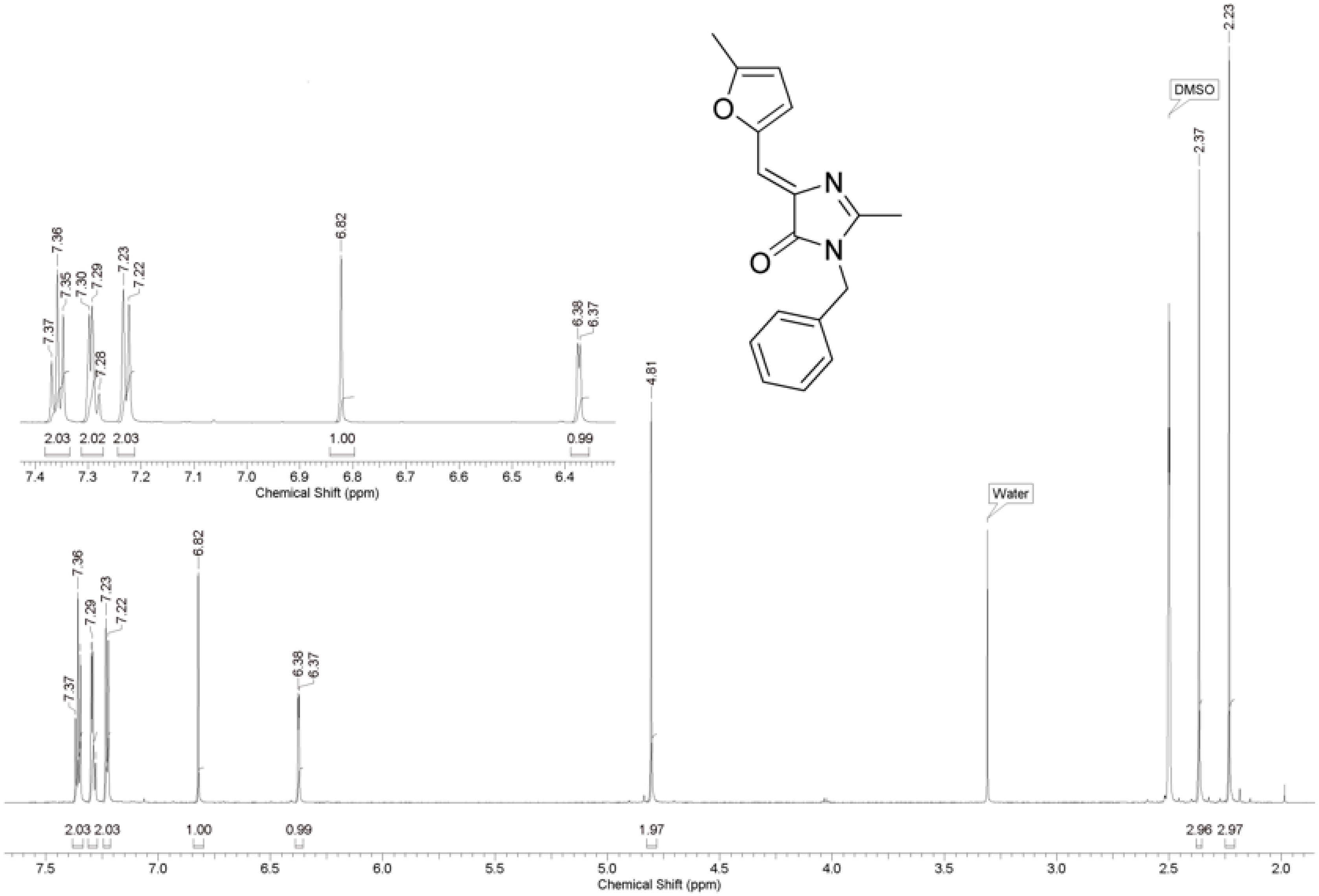
DMSO
Water
Chemical Shift (ppm)
2.23
2.37
4.81
6.82
6.38
6.37
7.36
7.35
7.37
7.30
7.29
7.28
7.23
7.22
2.03
2.02
1.00
0.99
1.97
2.96
2.97

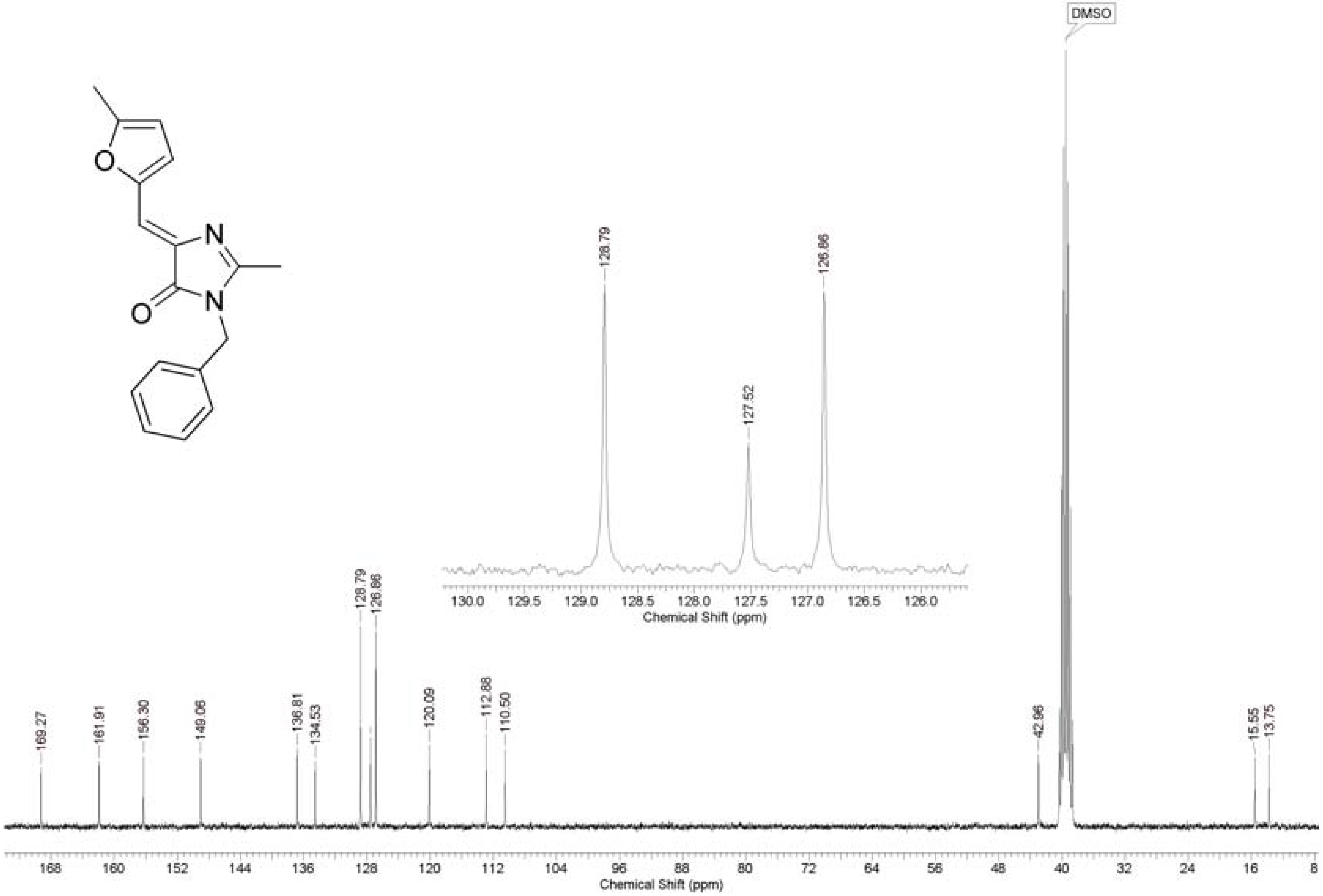

DMSO
128.79
127.52
126.86
130.0
129.5
129.0
128.5
128.0
127.5
127.0
126.5
126.0
Chemical Shift (ppm)
169.27
161.91
156.30
149.06
136.81
134.53
128.79
126.86
120.09
112.88
110.50
42.96
15.55
13.75
168
160
152
144
136
128
120
112
104
96
88
80
72
64
56
48
40
32
24
16
8
Chemical Shift (ppm)

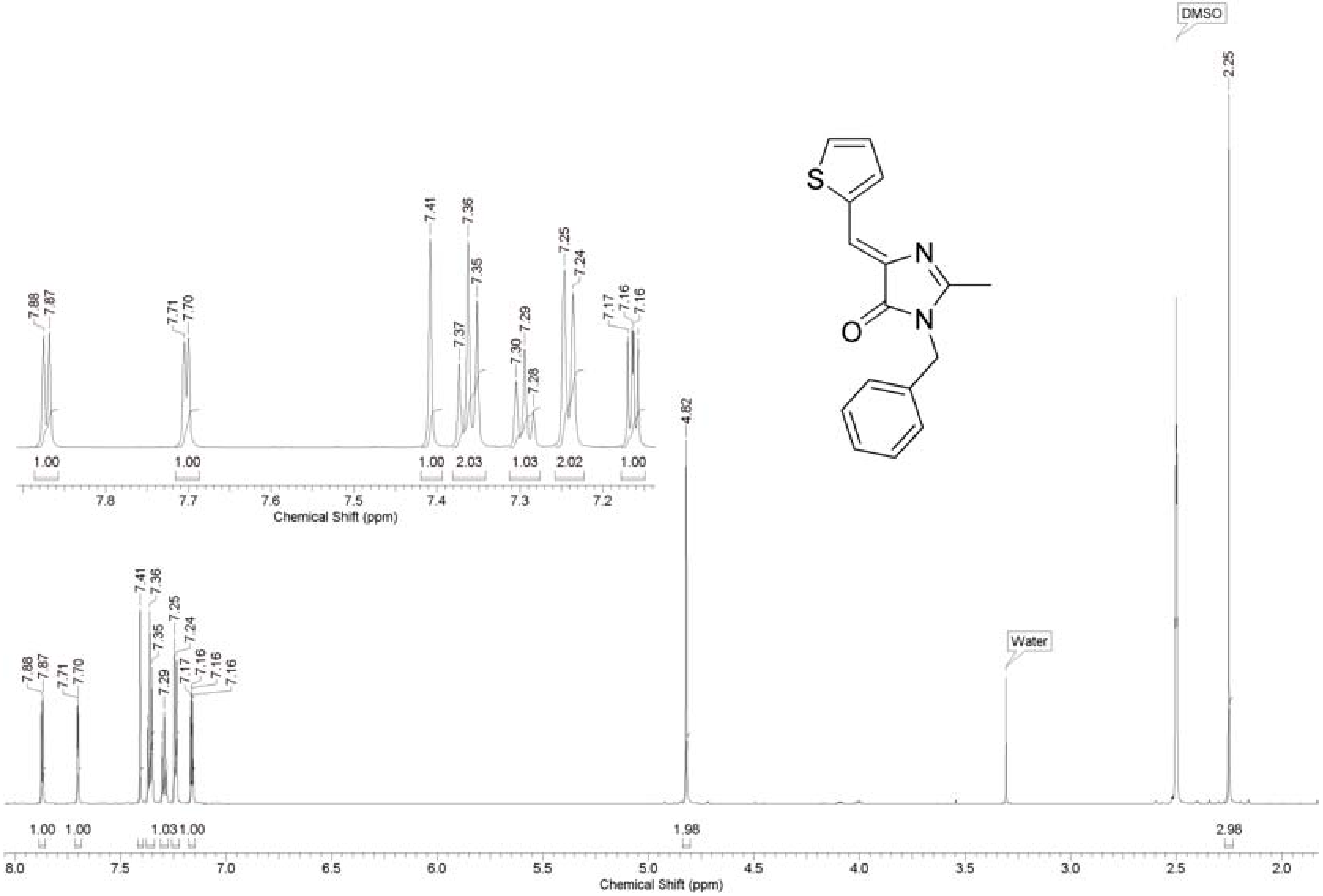
DMSO
Water
S
N
N
O
7.88
7.87
7.71
7.70
7.41
7.37
7.36
7.35
7.30
7.29
7.28
7.25
7.24
7.17
7.16
7.16
4.82
2.25
1.00
1.00
1.00
2.03
1.03
2.02
1.00
1.98
2.98
Chemical Shift (ppm)
Chemical Shift (ppm)

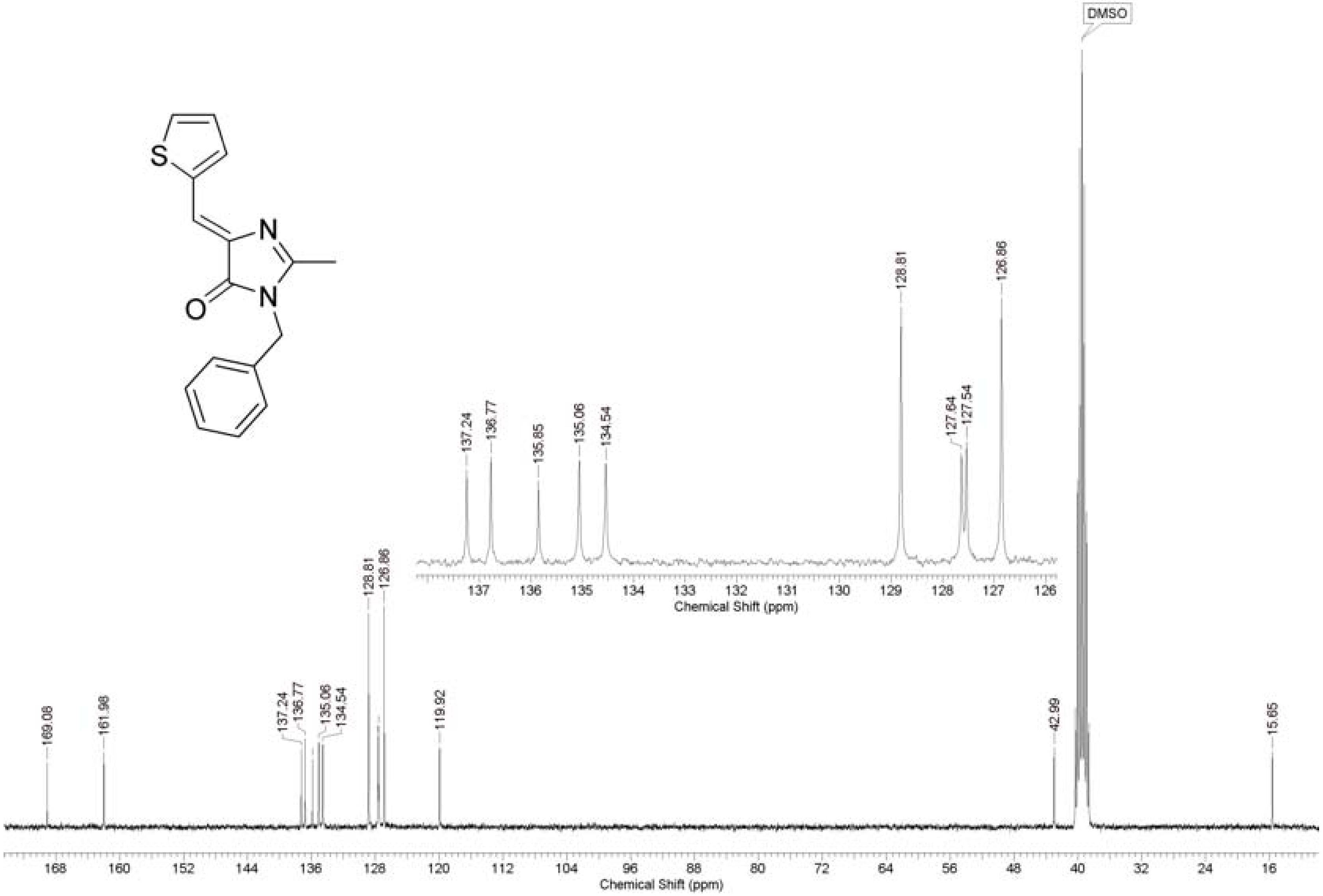
DMSO
137.24
136.77
135.85
135.06
134.54
128.81
127.64
127.54
126.86
137
136
135
134
133
132
131
130
129
128
127
126
Chemical Shift (ppm)
169.08
161.98
137.24
136.77
135.06
134.54
128.81
126.86
119.92
42.99
15.65
168
160
152
144
136
128
120
112
104
96
88
80
72
64
56
48
40
32
24
16
Chemical Shift (ppm)

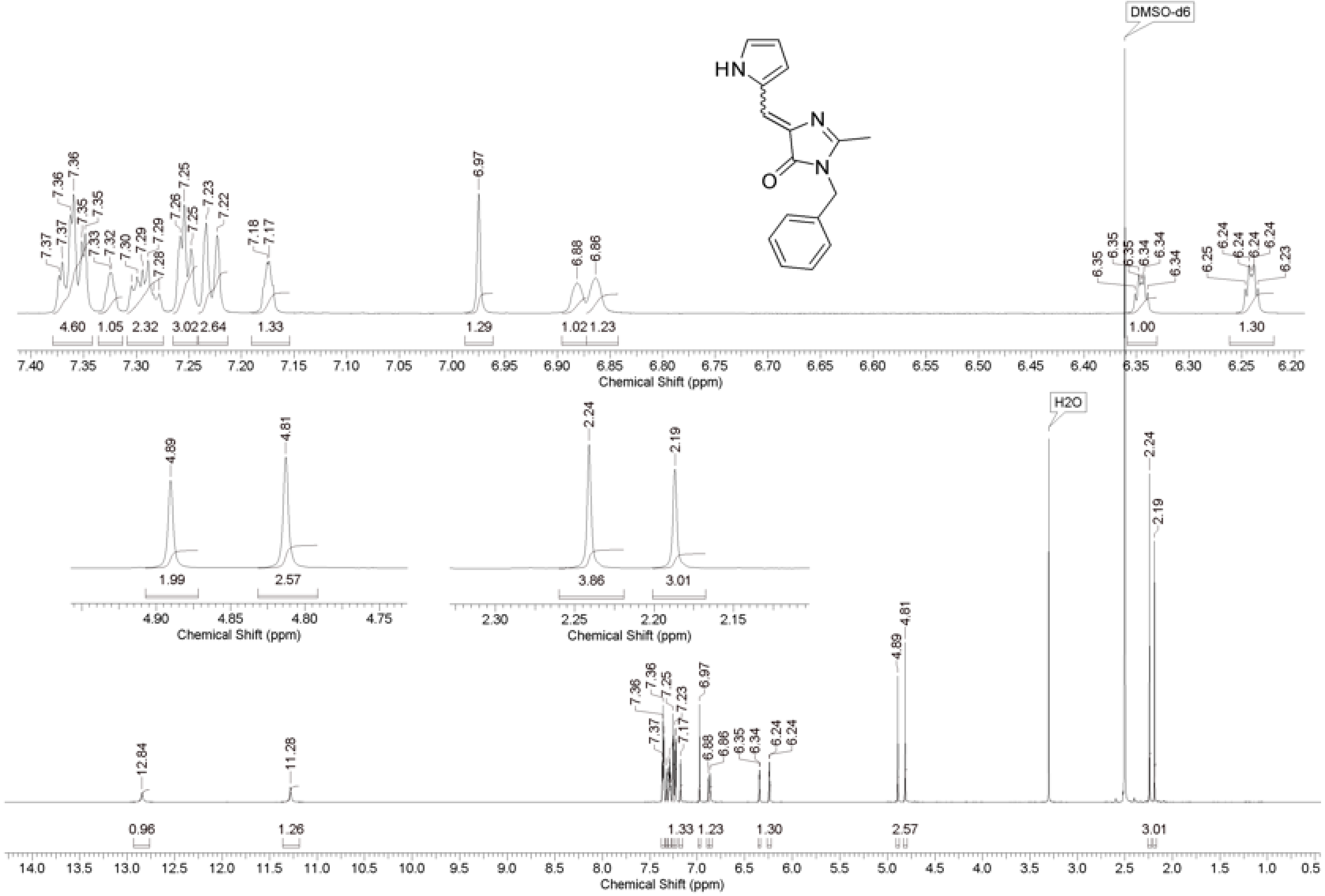
DMSO-d6
HN
N
O
N
7.37
7.37
7.36
7.36
7.35
7.35
7.33
7.32
7.30
7.29
7.29
7.28
7.26
7.25
7.25
7.23
7.22
7.18
7.17
6.97
6.88
6.86
6.35
6.35
6.35
6.34
6.34
6.34
6.25
6.24
6.24
6.24
6.24
6.23
4.60
1.05
2.32
3.02
2.64
1.33
1.29
1.02
1.23
1.00
1.30
Chemical Shift (ppm)
H2O
4.89
4.81
1.99
2.57
2.24
2.19
3.86
3.01
12.84
11.28
0.96
1.26

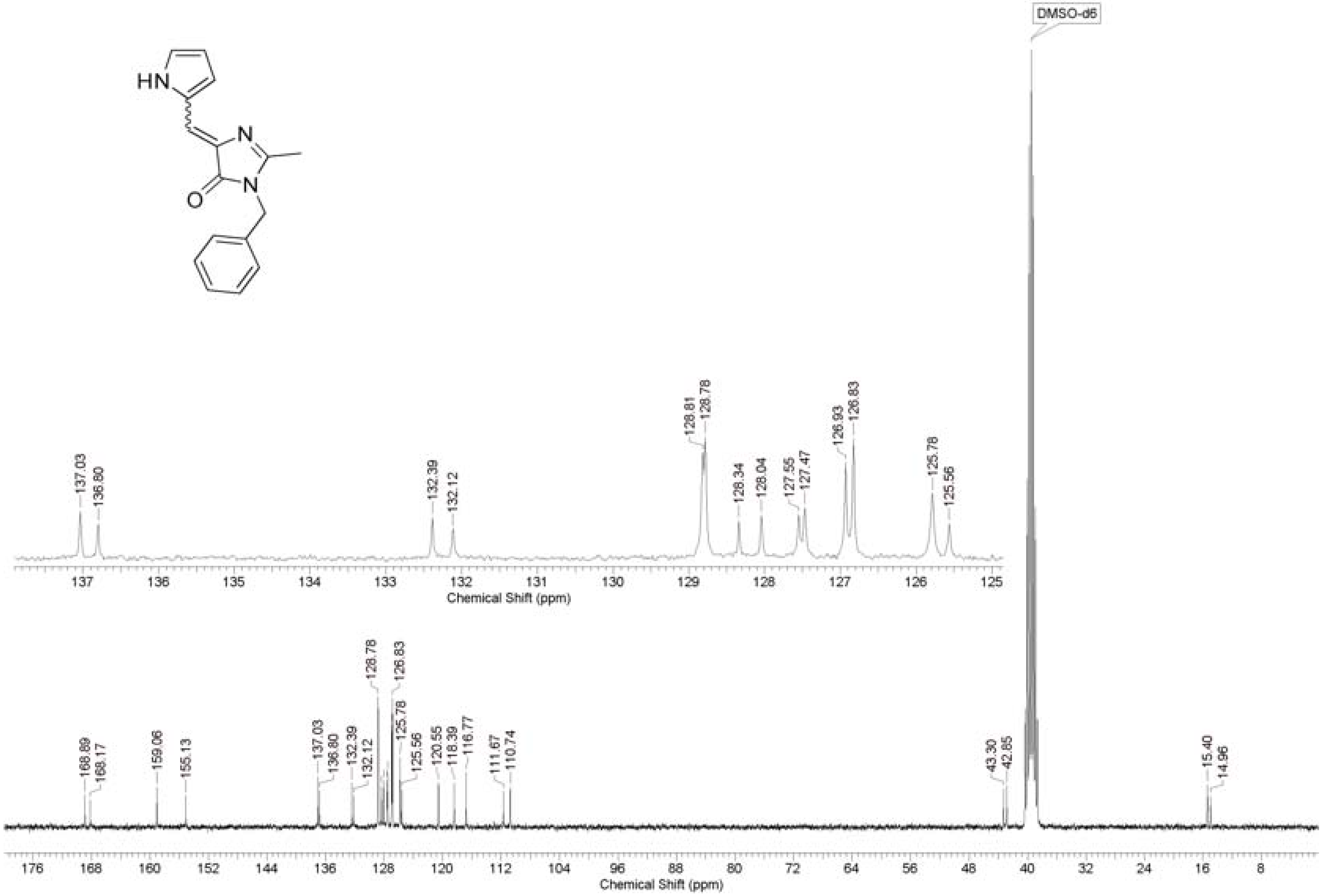
HN
N
N
O
DMSO-d6
137.03
136.80
132.39
132.12
128.81
128.78
128.34
128.04
127.55
127.47
126.93
126.83
125.78
125.56
137 136 135 134 133 132 131 130 129 128 127 126 125
Chemical Shift (ppm)
168.89
168.17
159.06
155.13
137.03
136.80
132.39
132.12
128.78
126.83
125.78
125.56
120.55
118.39
116.77
111.67
110.74
43.30
42.85
15.40
14.96
176 168 160 152 144 136 128 120 112 104 96 88 80 72 64 56 48 40 32 24 16 8
Chemical Shift (ppm)

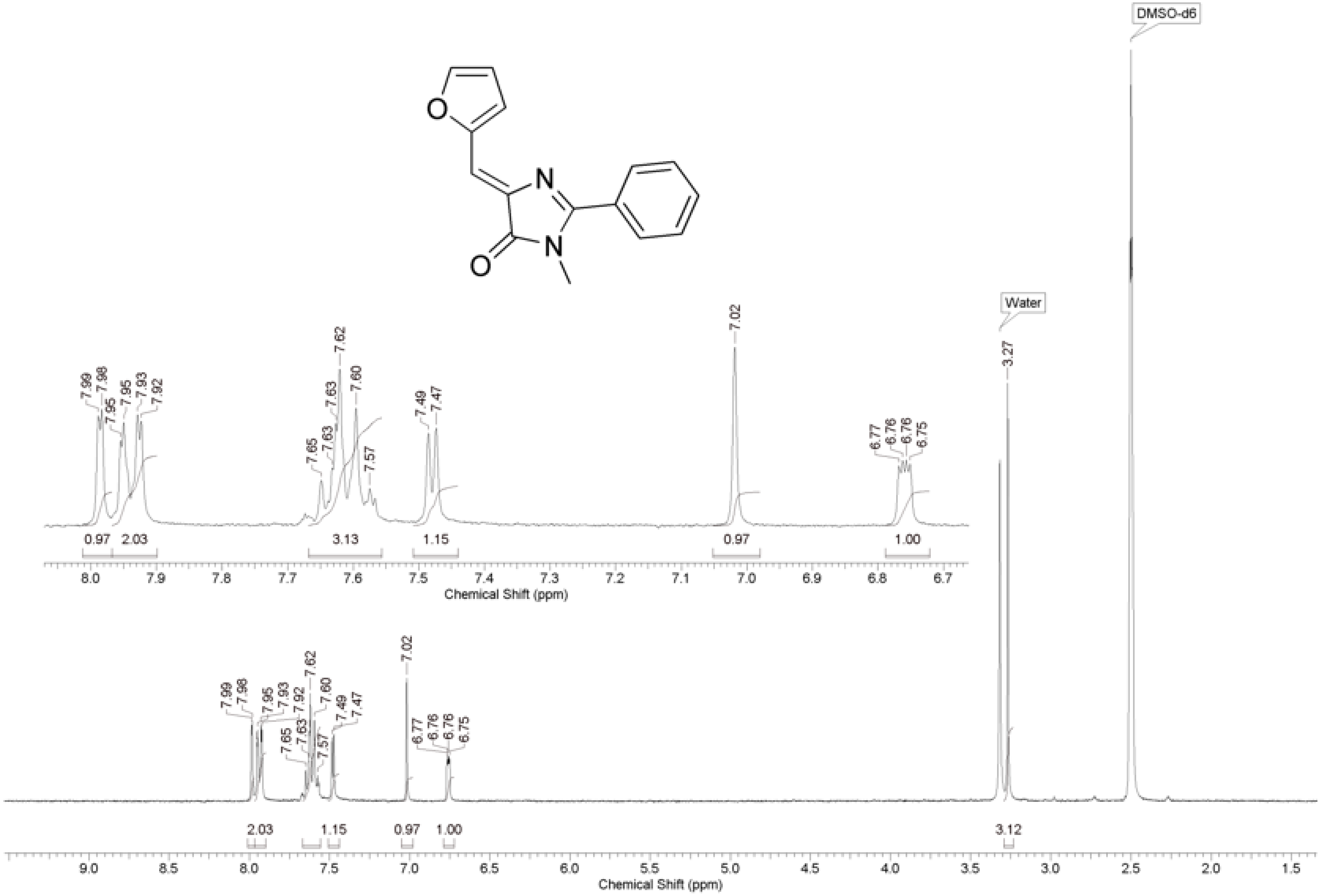
DMSO-d6
Water
3.27
7.02
7.99 7.98 7.95 7.93 7.92
7.65 7.63 7.63 7.62 7.60 7.57
7.49 7.47
6.77 6.76 6.76 6.75
0.97 2.03 3.13 1.15 0.97 1.00
2.03 1.15 0.97 1.00 3.12
Chemical Shift (ppm)
Chemical Shift (ppm)

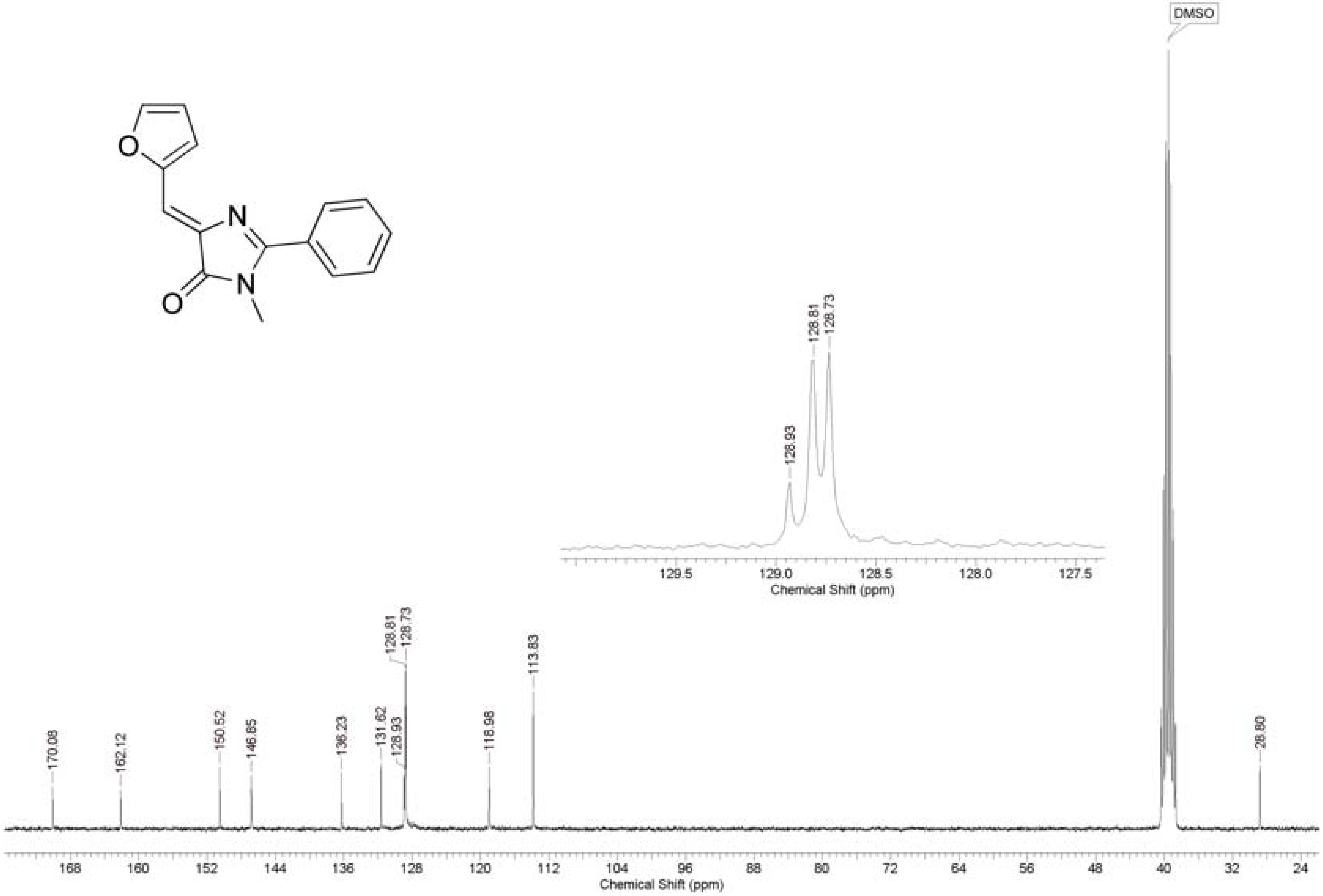
DMSO
170.08
162.12
150.52
146.85
136.23
131.62
128.93
128.81
128.73
118.98
113.83
28.80
168
160
152
144
136
128
120
112
104
96
88
80
72
64
56
48
40
32
24
Chemical Shift (ppm)
129.5
129.0
128.5
128.0
127.5

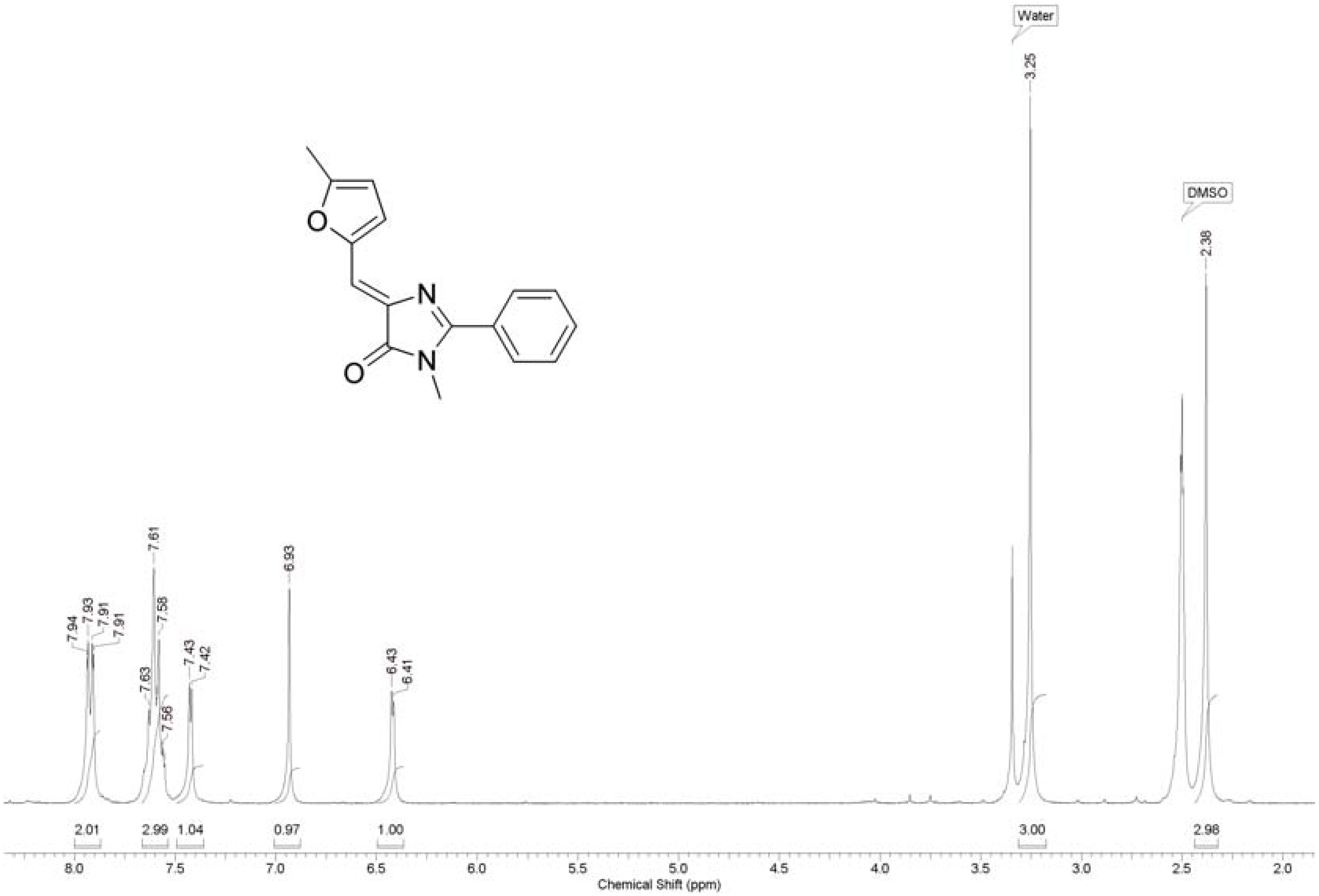
Water
DMSO
7.94
7.93
7.91
7.91
7.61
7.58
7.63
7.56
7.43
7.42
6.93
6.43
6.41
3.25
2.38
2.01
2.99
1.04
0.97
1.00
3.00
2.98
8.0
7.5
7.0
6.5
6.0
5.5
5.0
4.5
4.0
3.5
3.0
2.5
2.0
Chemical Shift (ppm)
O
N
N
O

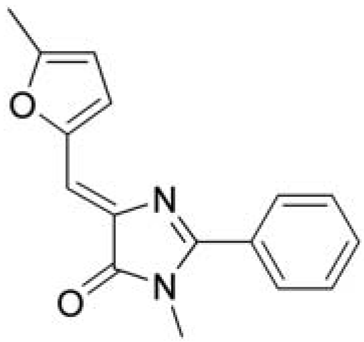

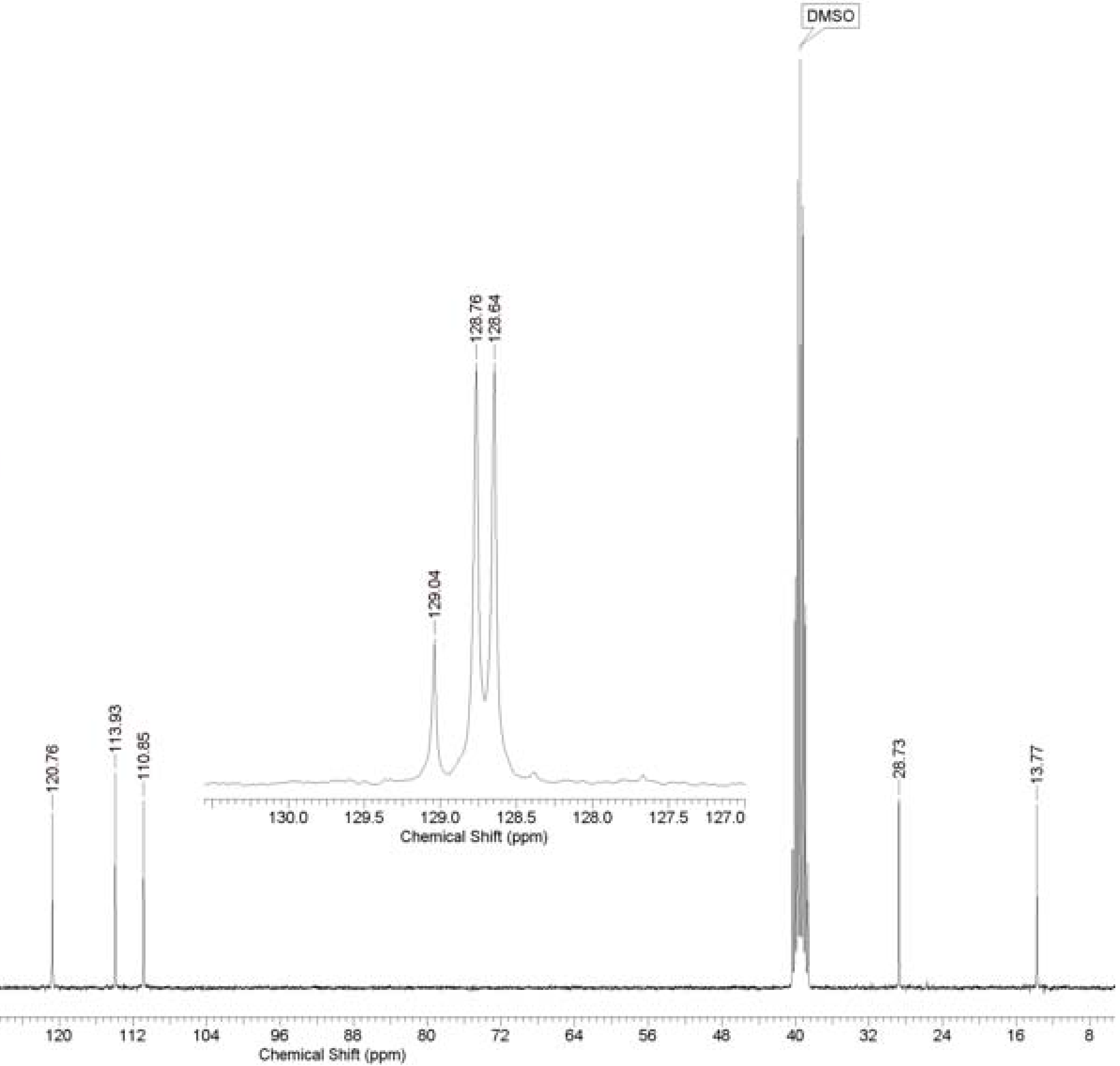
DMSO
128.76
128.64
129.04
169.95
161.11
156.62
149.35
135.08
131.42
120.76
113.93
110.85
28.73
13.77
Chemical Shift (ppm)

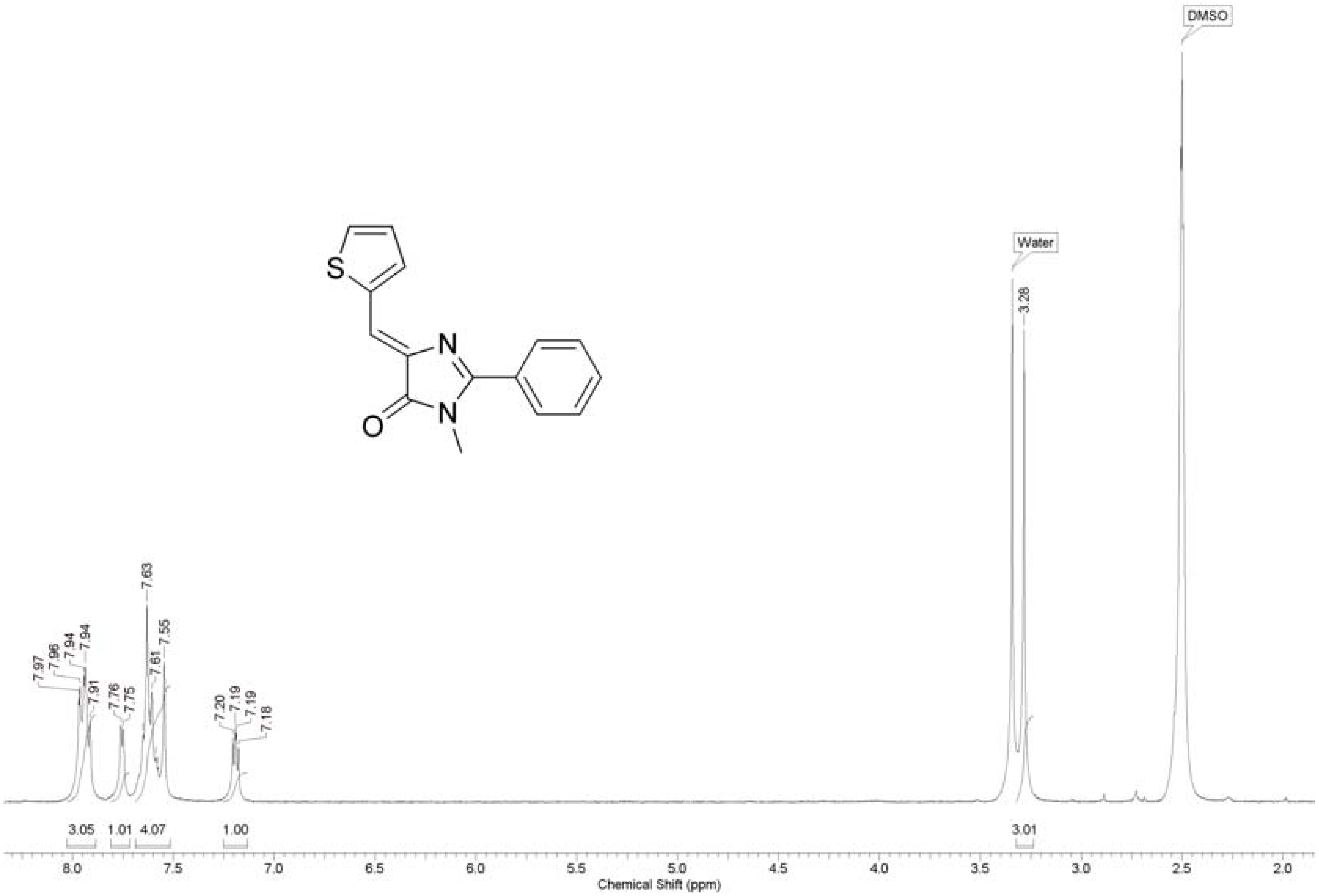
DMSO
Water
3.28
7.97
7.96
7.94
7.94
7.91
7.76
7.75
7.63
7.61
7.55
7.20
7.19
7.19
7.18
3.05
1.01
4.07
1.00
3.01
8.0
7.5
7.0
6.5
6.0
5.5
5.0
4.5
4.0
3.5
3.0
2.5
2.0
Chemical Shift (ppm)
S
N
N
O

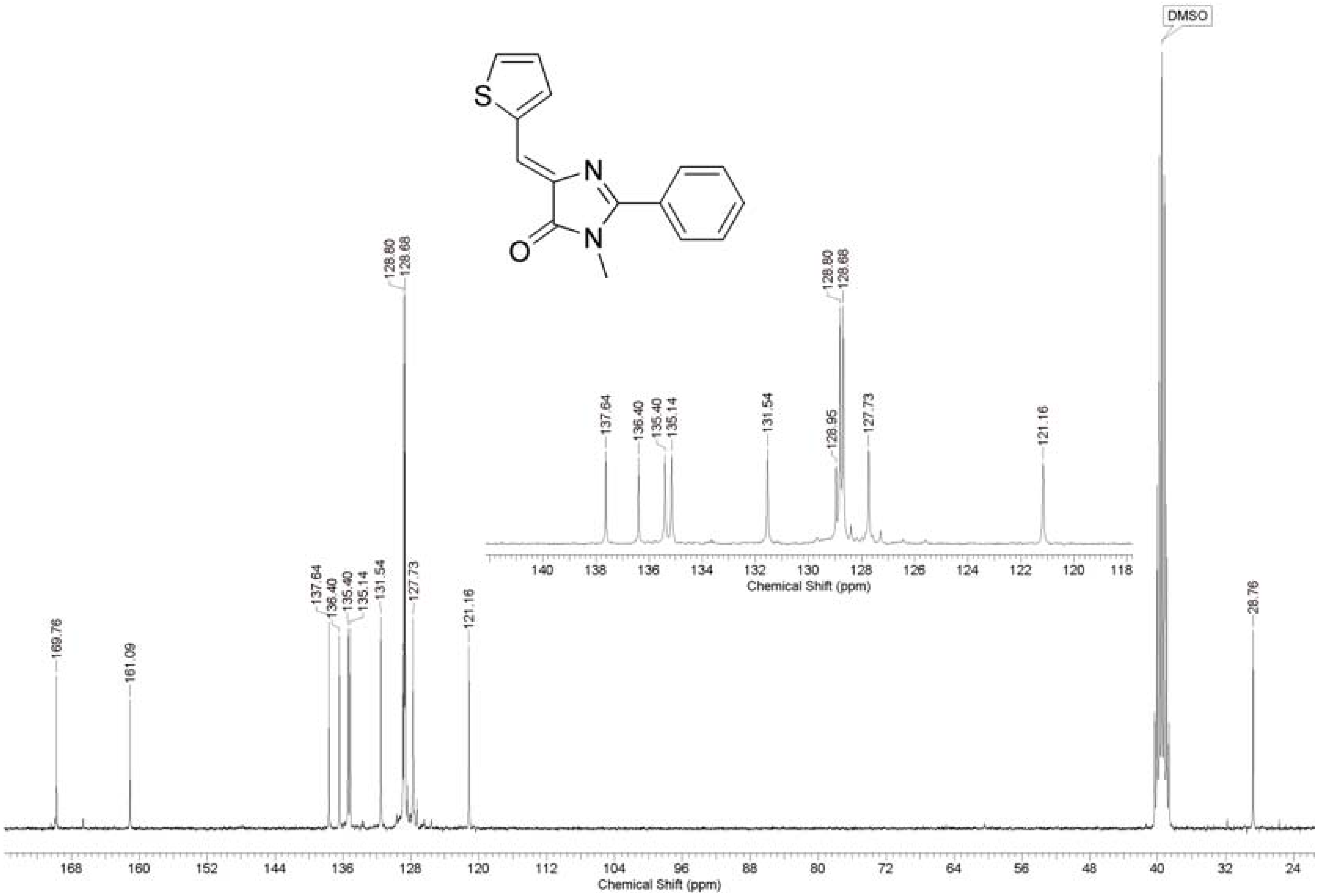

S
N
N
O
DMSO
169.76
161.09
137.64
136.40
135.40
135.14
131.54
128.80
128.68
127.73
121.16
28.76
128.95
Chemical Shift (ppm)
Chemical Shift (ppm)

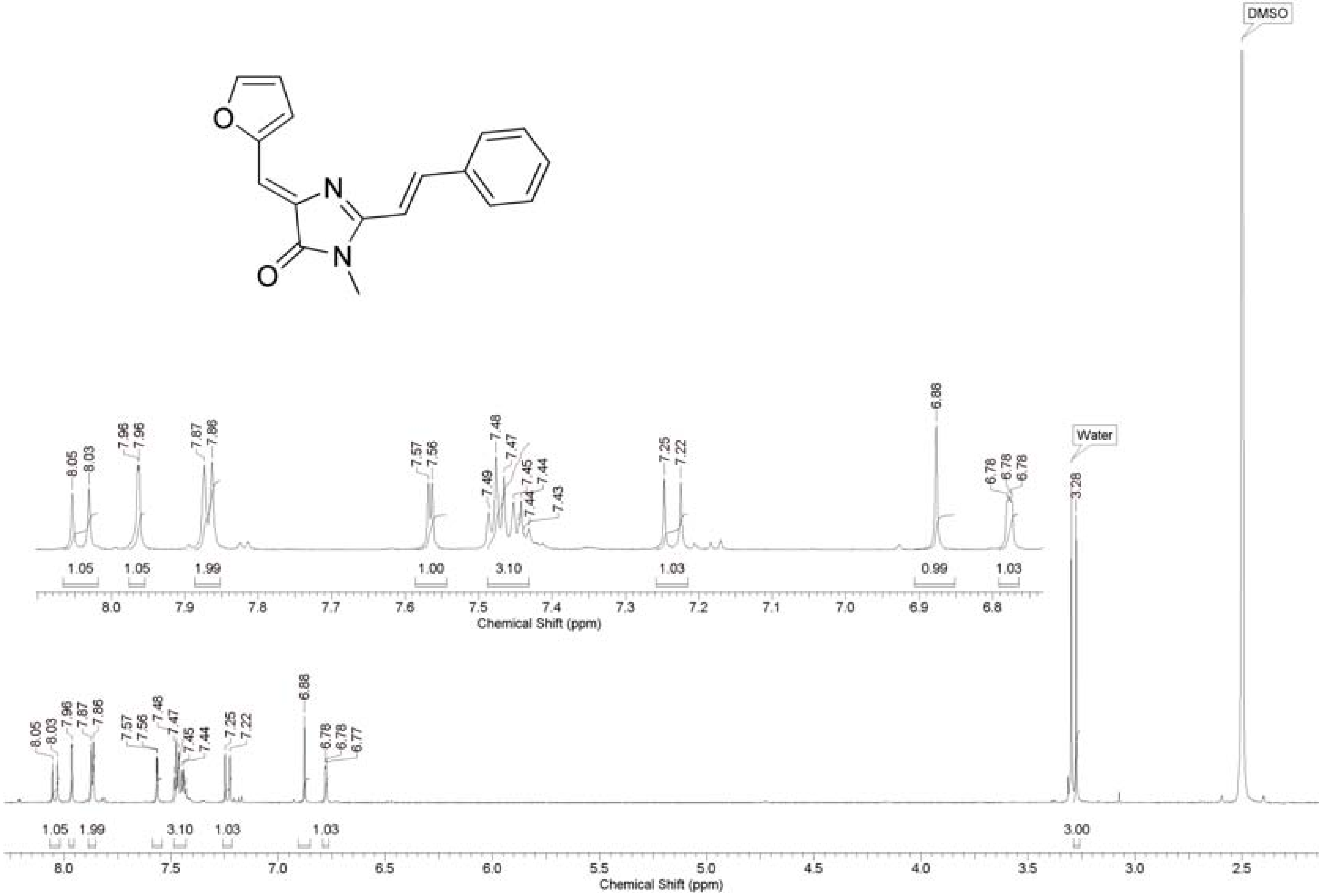
O
N
N
O
DMSO
Water
8.05
8.03
7.96
7.96
7.87
7.86
7.57
7.56
7.49
7.48
7.47
7.45
7.44
7.44
7.43
7.25
7.22
6.88
6.78
6.78
6.78
3.28
1.05
1.05
1.99
1.00
3.10
1.03
0.99
1.03
Chemical Shift (ppm)
8.05
8.03
7.96
7.87
7.86
7.57
7.56
7.48
7.47
7.45
7.44
7.25
7.22
6.88
6.78
6.78
6.77
1.05
1.99
3.10
1.03
1.03
3.00
Chemical Shift (ppm)

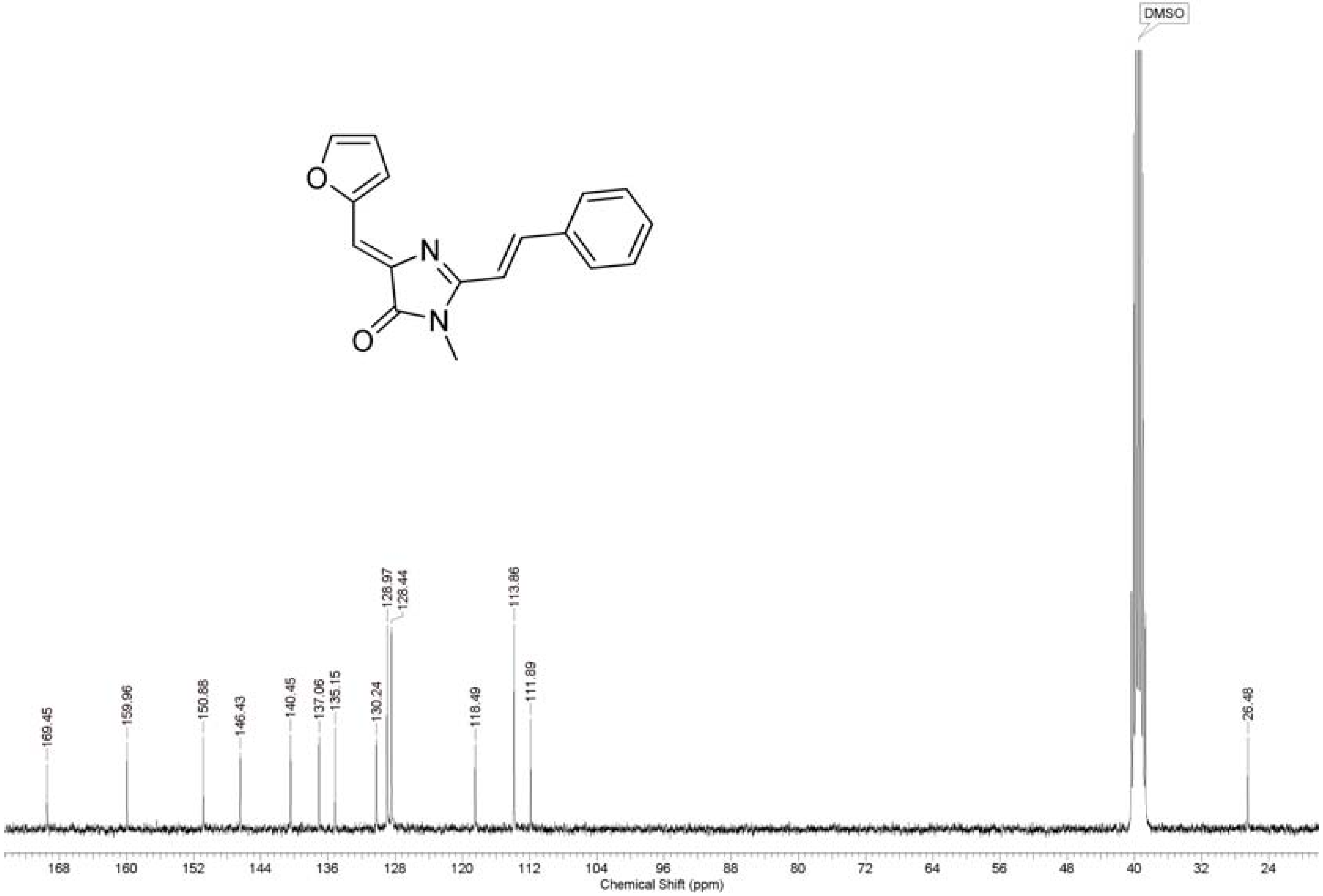

DMSO
169.45
159.96
150.88
146.43
140.45
137.06
135.15
130.24
128.97
128.44
118.49
113.86
111.89
26.48
Chemical Shift (ppm)

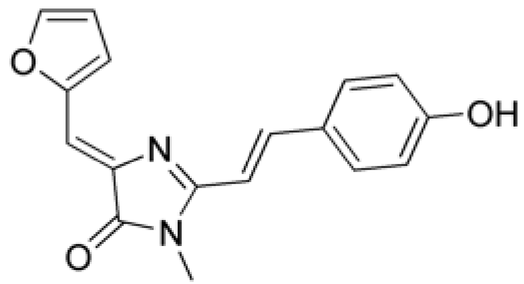

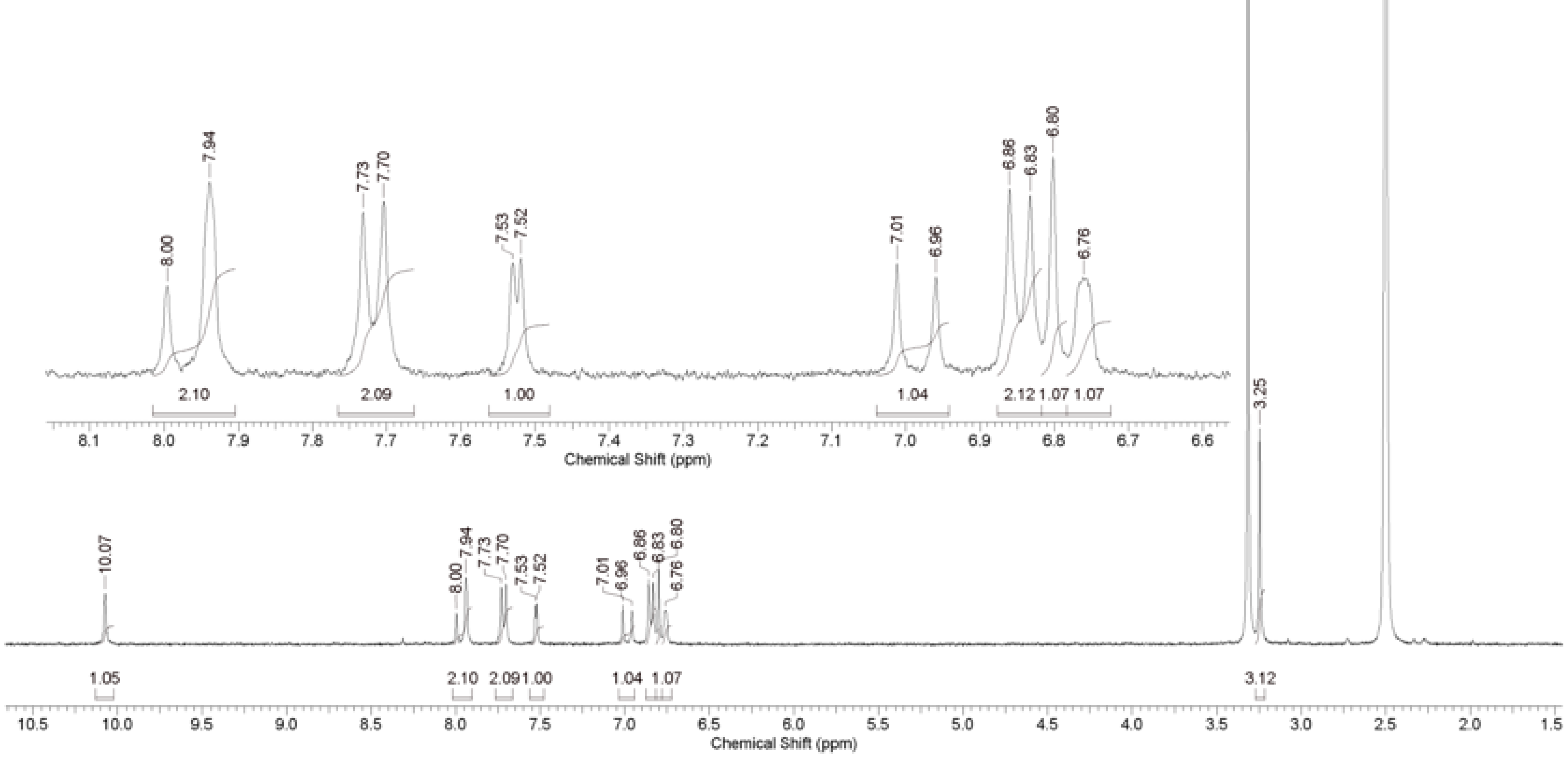
DMSO-d6
H2O
8.00
7.94
7.73
7.70
7.53
7.52
7.01
6.96
6.86
6.83
6.80
6.76
2.10
2.09
1.00
1.04
2.12
1.07
1.07
Chemical Shift (ppm)
10.07
3.25
1.05
3.12
Chemical Shift (ppm)

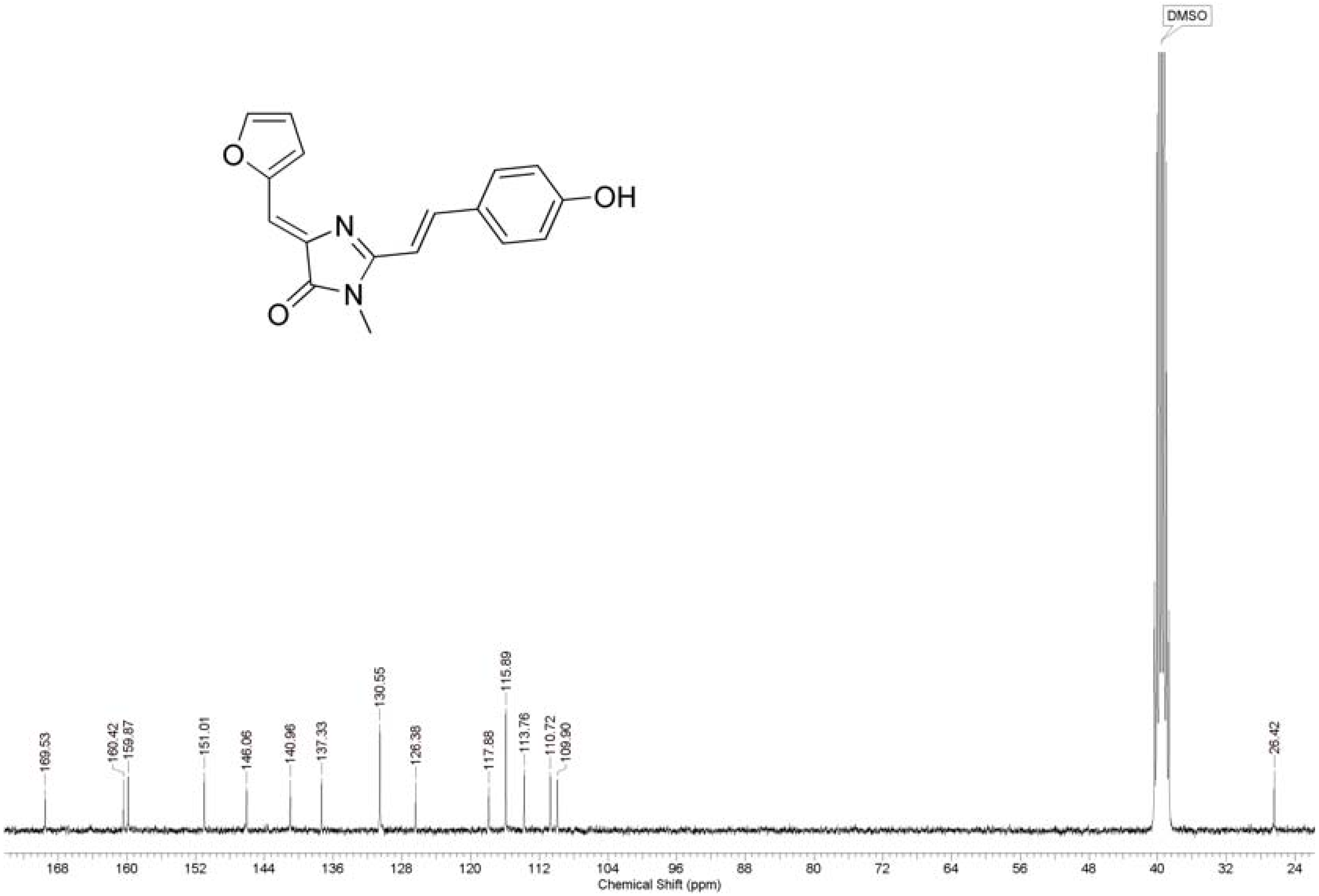
DMSO
169.53
160.42
159.87
151.01
146.06
140.96
137.33
130.55
126.38
117.88
115.89
113.76
110.72
109.90
26.42
Chemical Shift (ppm)

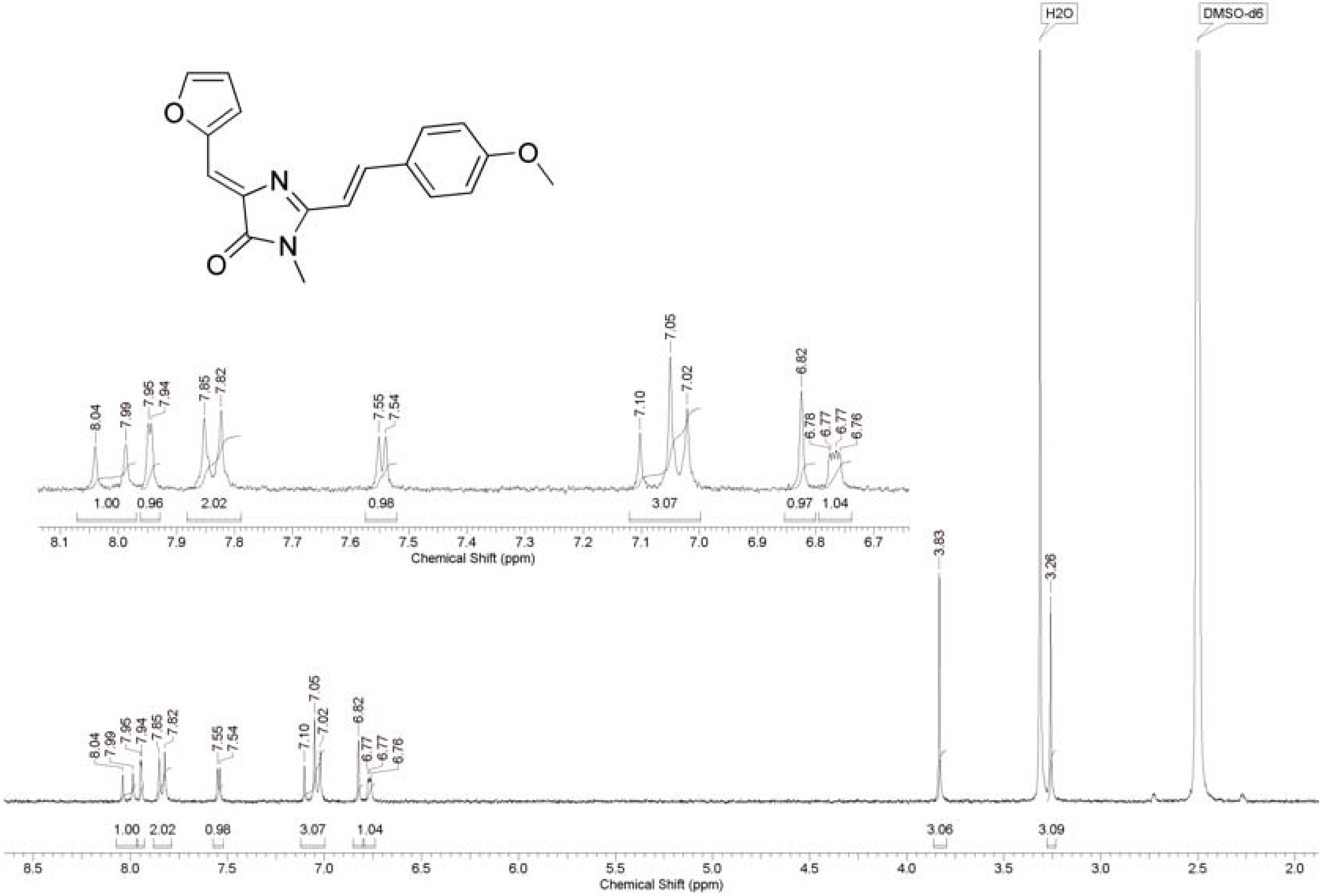

H2O
DMSO-d6
8.04
7.99
7.95
7.94
7.85
7.82
7.55
7.54
7.10
7.05
7.02
6.82
6.78
6.77
6.77
6.76
1.00
0.96
2.02
0.98
3.07
0.97
1.04
Chemical Shift (ppm)
3.83
3.26
3.06
3.09
Chemical Shift (ppm)

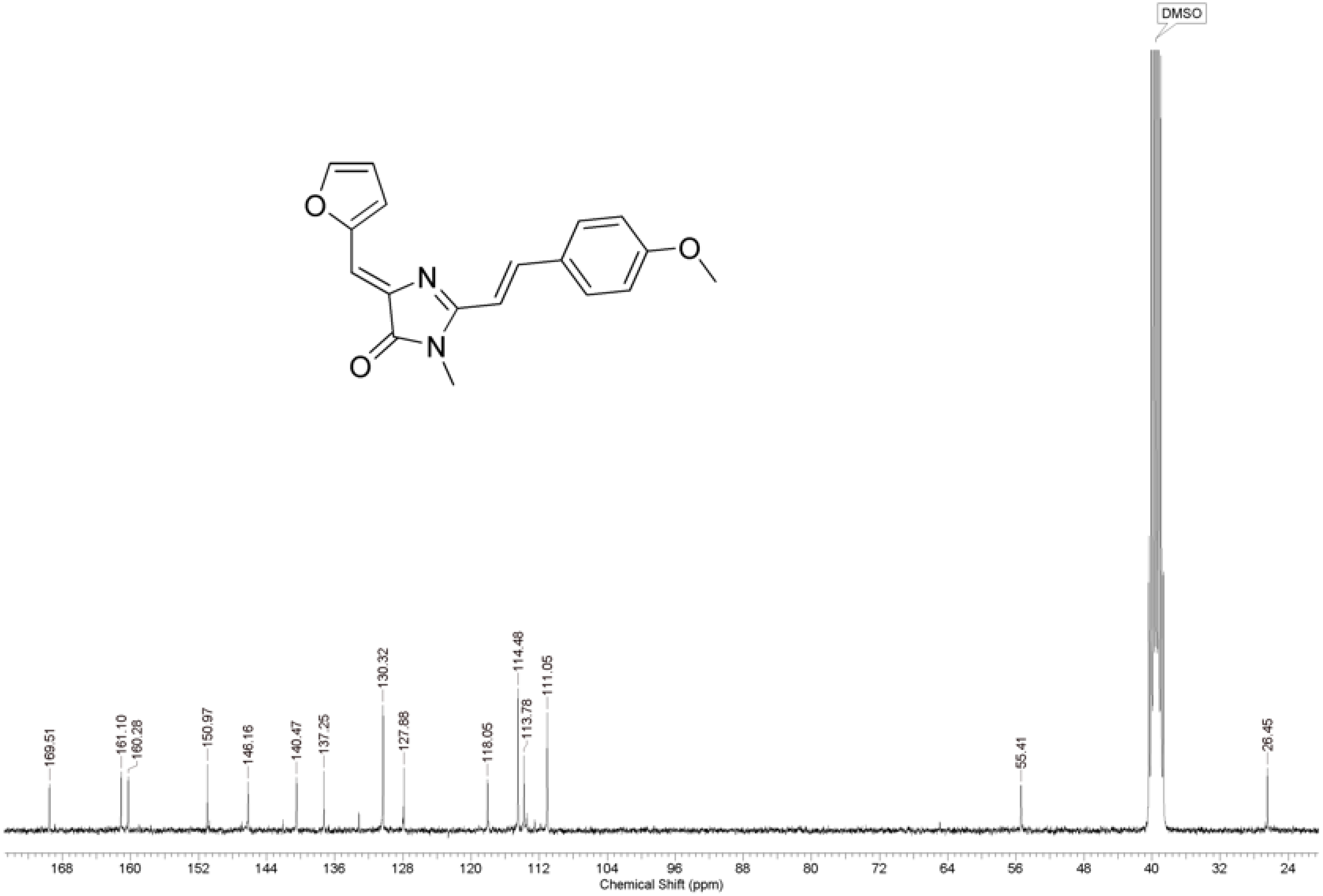

DMSO
169.51
161.10
160.28
150.97
146.16
140.47
137.25
130.32
127.88
118.05
114.48
113.78
111.05
55.41
26.45
168
160
152
144
136
128
120
112
104
96
88
80
72
64
56
48
40
32
24
Chemical Shift (ppm)

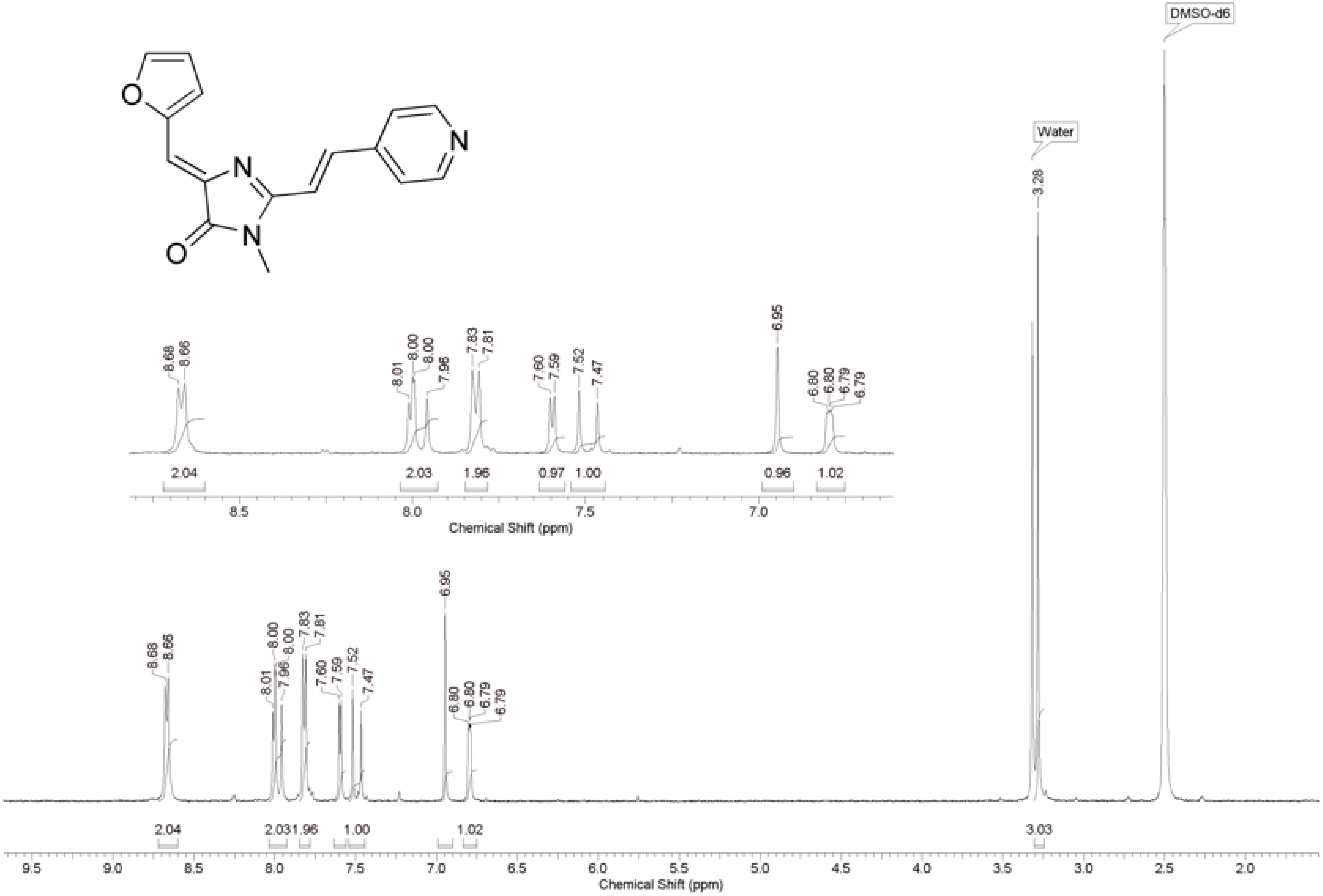

O
N
N
N
O
DMSO-d6
Water
3.28
8.68
8.66
8.01
8.00
8.00
7.96
7.83
7.81
7.60
7.59
7.52
7.47
6.95
6.80
6.80
6.79
6.79
2.04
2.03
1.96
0.97
1.00
0.96
1.02
3.03
Chemical Shift (ppm)

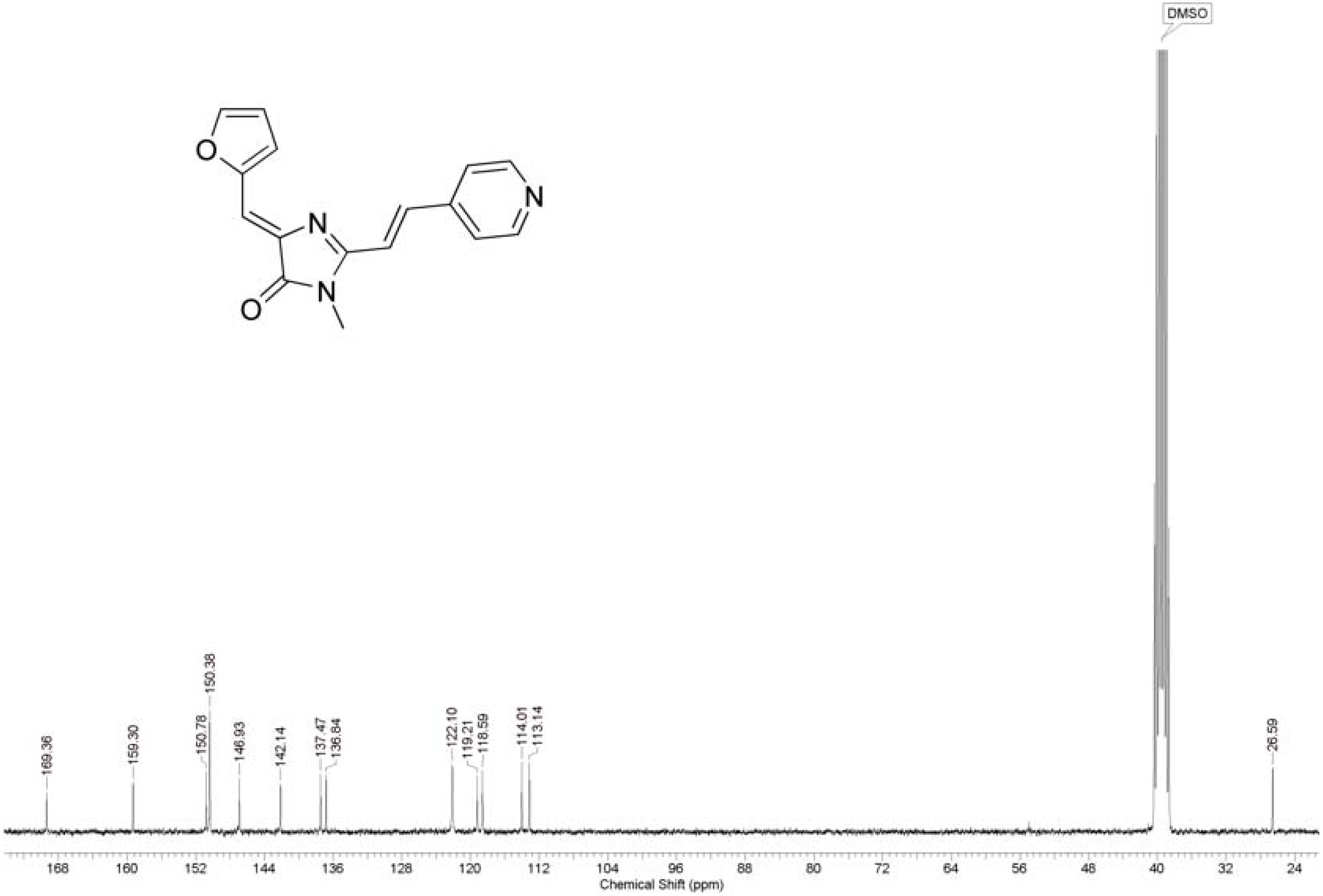
DMSO
169.36
159.30
150.78
150.38
146.93
142.14
137.47
136.84
122.10
119.21
118.59
114.01
113.14
26.59
168
160
152
144
136
128
120
112
104
96
88
80
72
64
56
48
40
32
24
Chemical Shift (ppm)
O
N
N
O
N

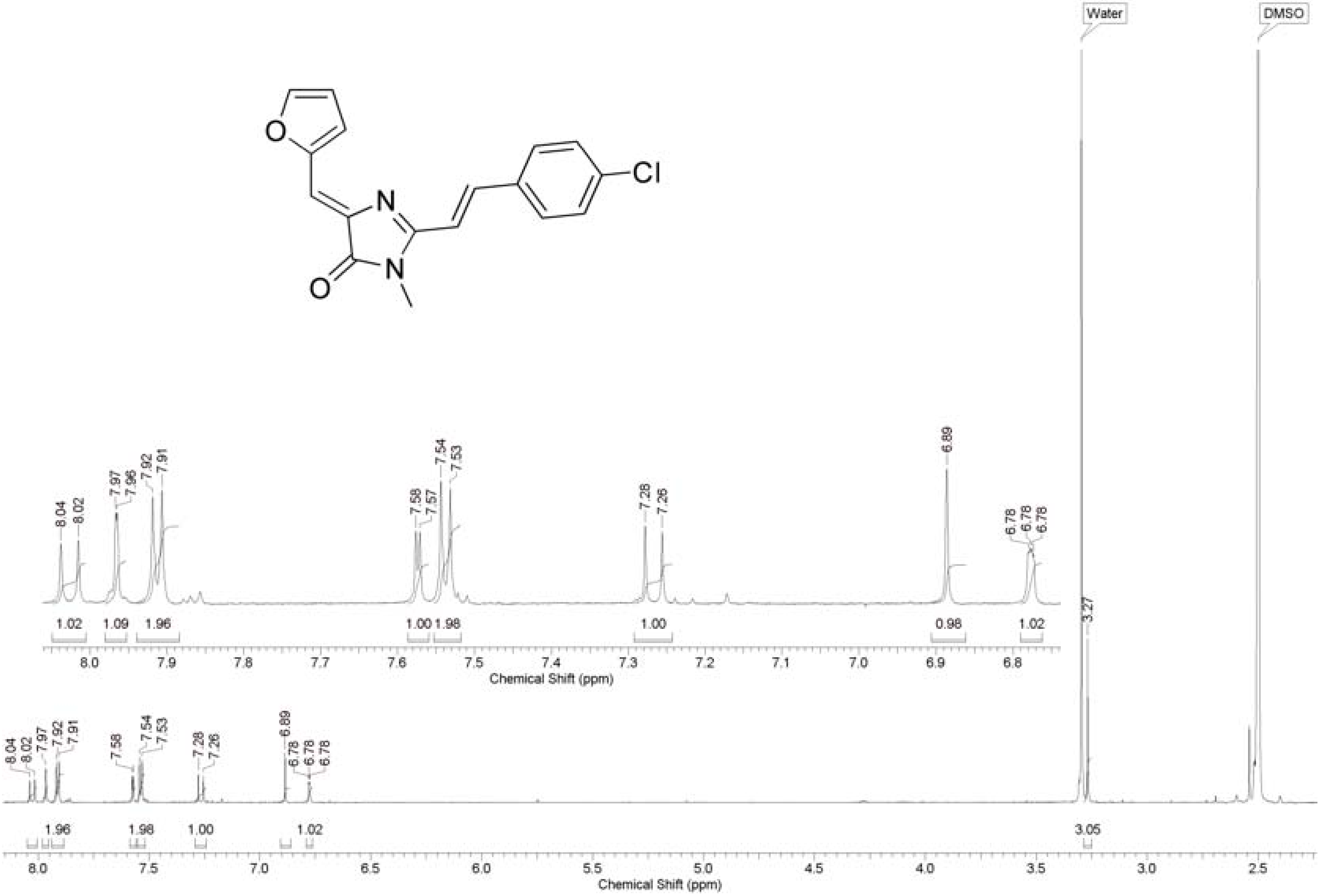

Water
DMSO
O
N
N
O
Cl
8.04
8.02
7.97
7.96
7.92
7.91
7.58
7.57
7.54
7.53
7.28
7.26
6.89
6.78
6.78
6.78
3.27
1.02 1.09 1.96 1.00 1.98 1.00 0.98 1.02
Chemical Shift (ppm)
1.96 1.98 1.00 1.02 3.05
Chemical Shift (ppm)

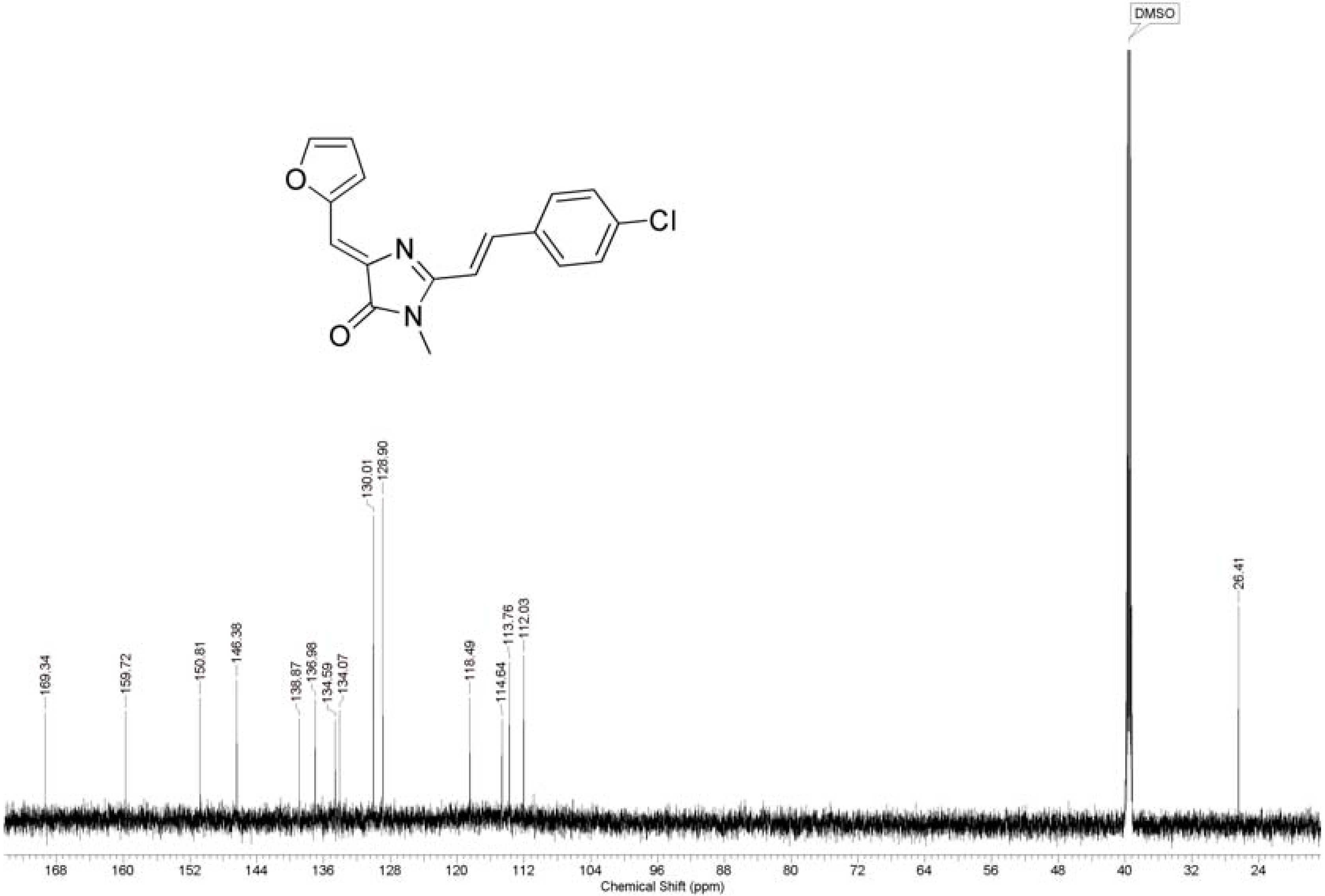

DMSO
169.34
159.72
150.81
146.38
138.87
136.98
134.59
134.07
130.01
128.90
118.49
114.64
113.76
112.03
26.41
168
160
152
144
136
128
120
112
104
96
88
80
72
64
56
48
40
32
24
Chemical Shift (ppm)
N
O
Cl

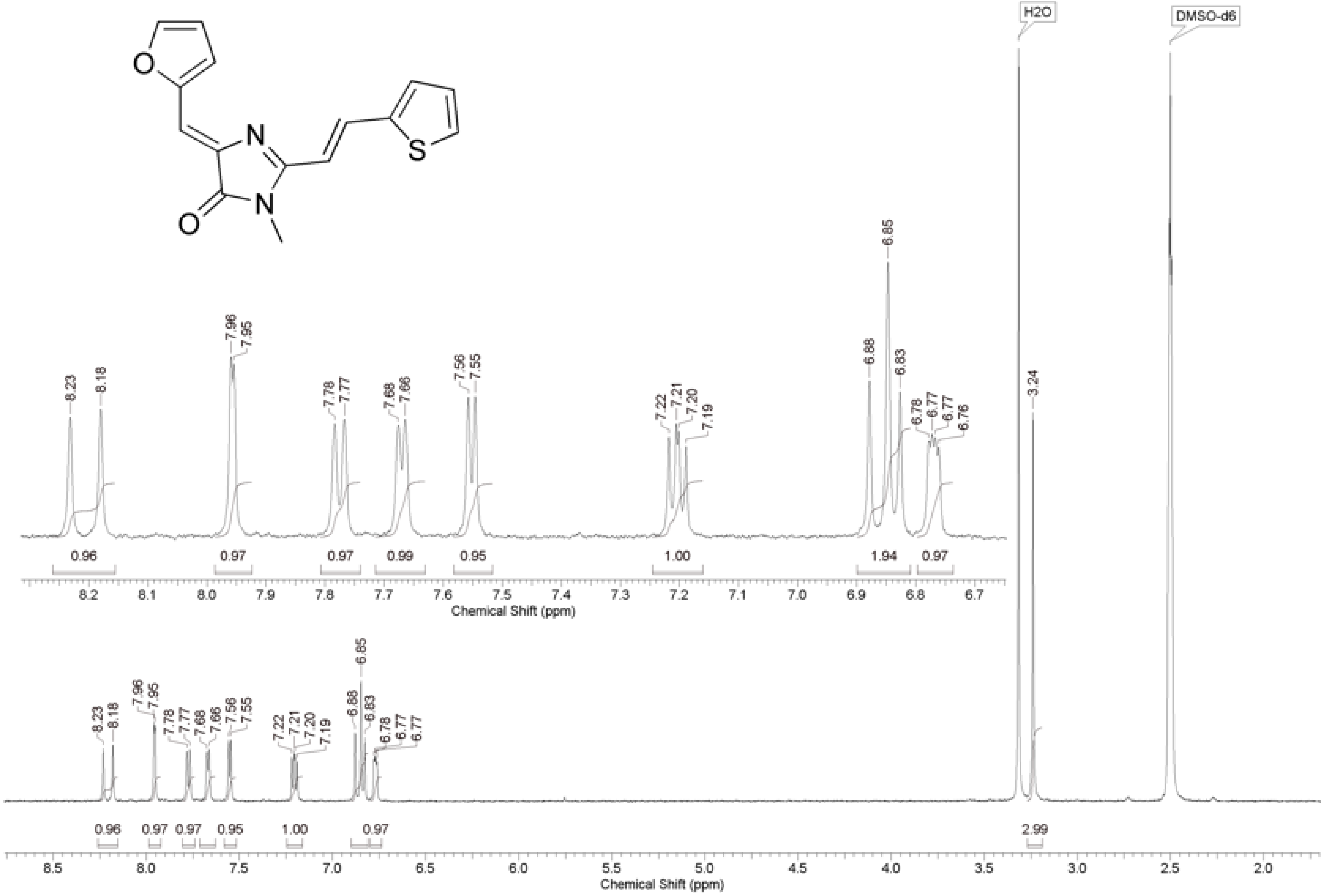

O
N
S
O
N
H2O
DMSO-d6
Chemical Shift (ppm)
Chemical Shift (ppm)

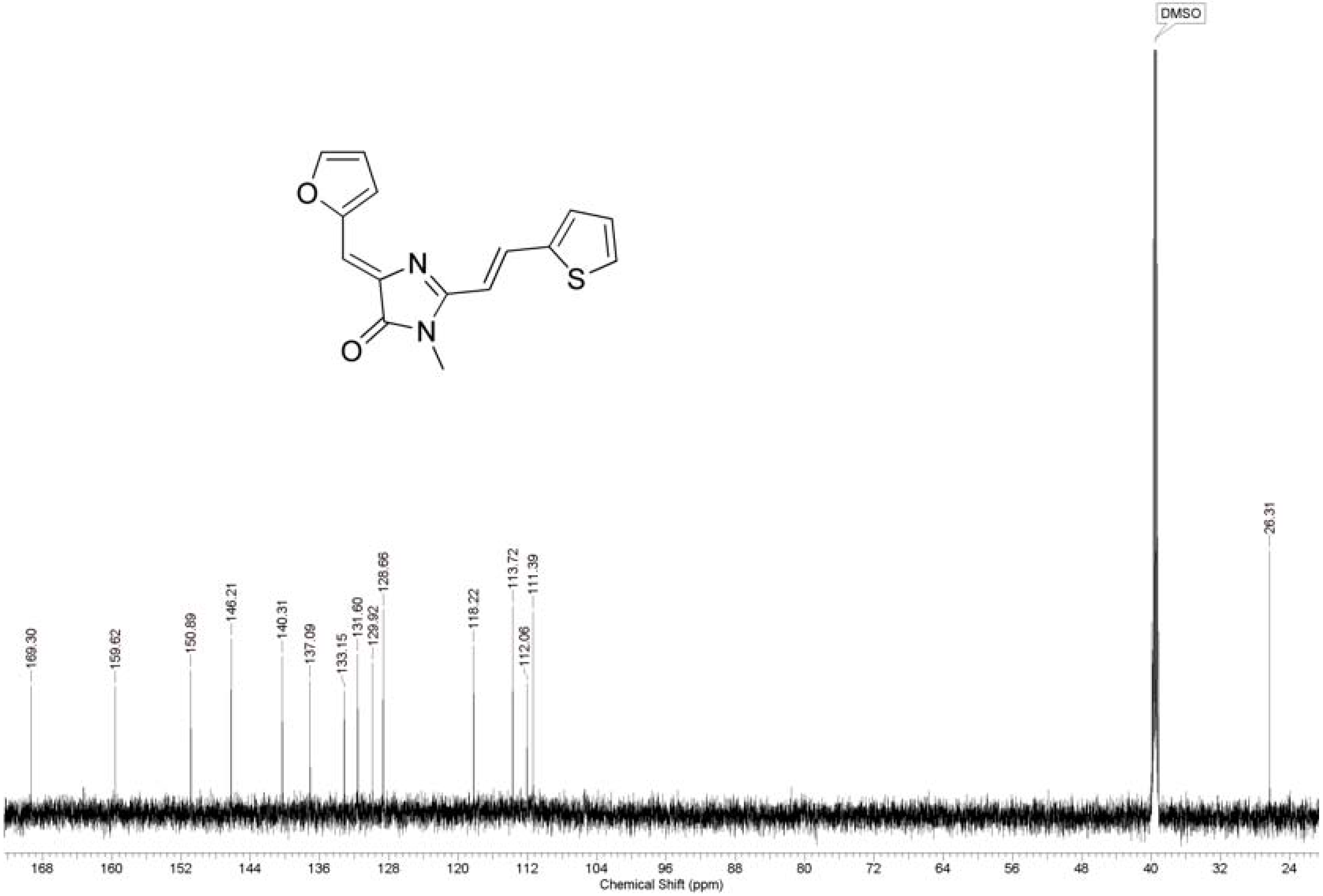
DMSO
169.30
159.62
150.89
146.21
140.31
137.09
133.15
131.60
129.92
128.66
118.22
113.72
112.06
111.39
26.31
168
160
152
144
136
128
120
112
104
96
88
80
72
64
56
48
40
32
24
Chemical Shift (ppm)
O
N
N
O
S

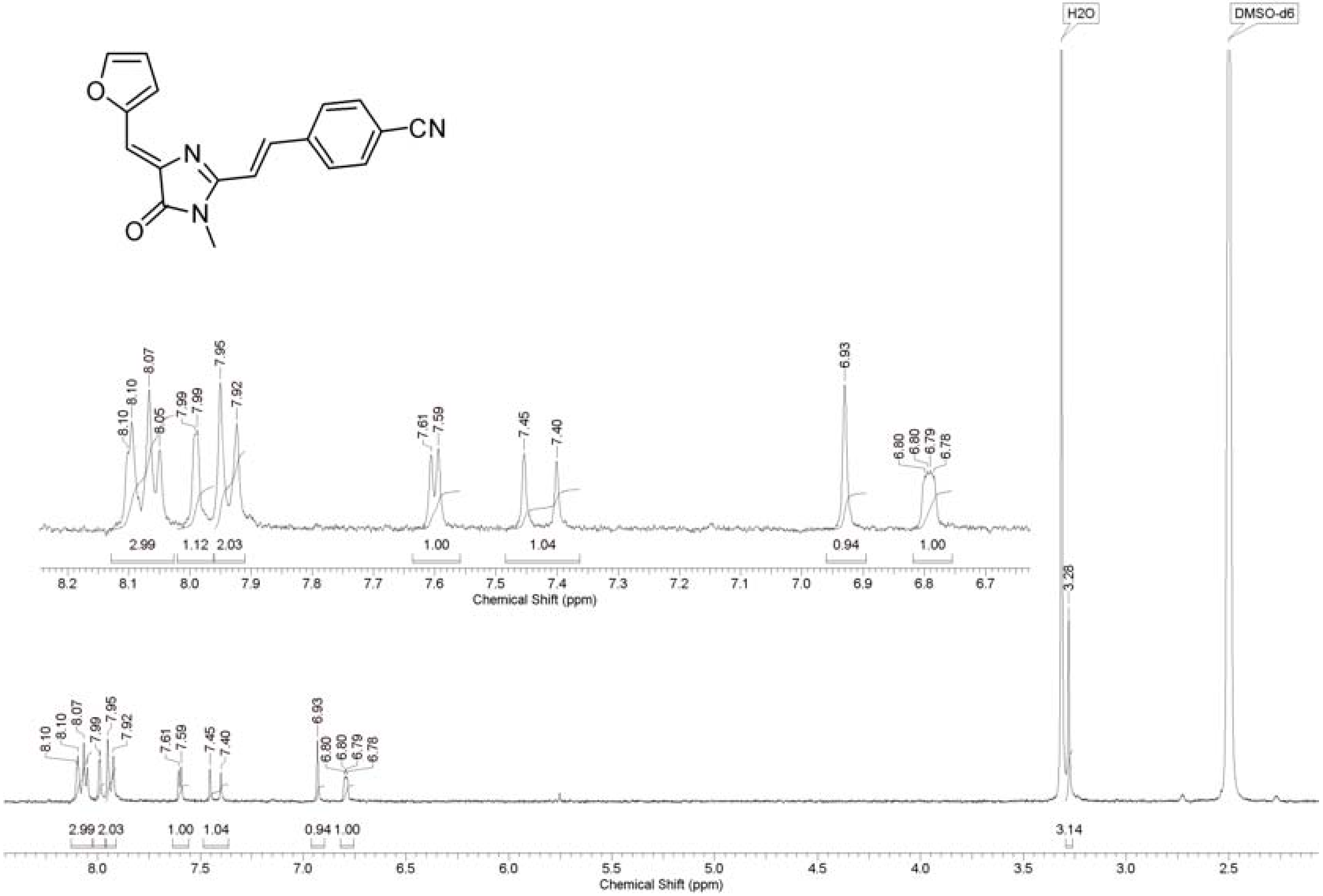

CN
N
N
O
O
H2O
DMSO-d6
8.10 8.10 8.07 8.05 7.99 7.99 7.95 7.92
7.61 7.59
7.45 7.40
6.93
6.80 6.80 6.79 6.78
2.99 1.12 2.03 1.00 1.04 0.94 1.00
Chemical Shift (ppm)
3.28
2.99 2.03 1.00 1.04 0.94 1.00 3.14
Chemical Shift (ppm)

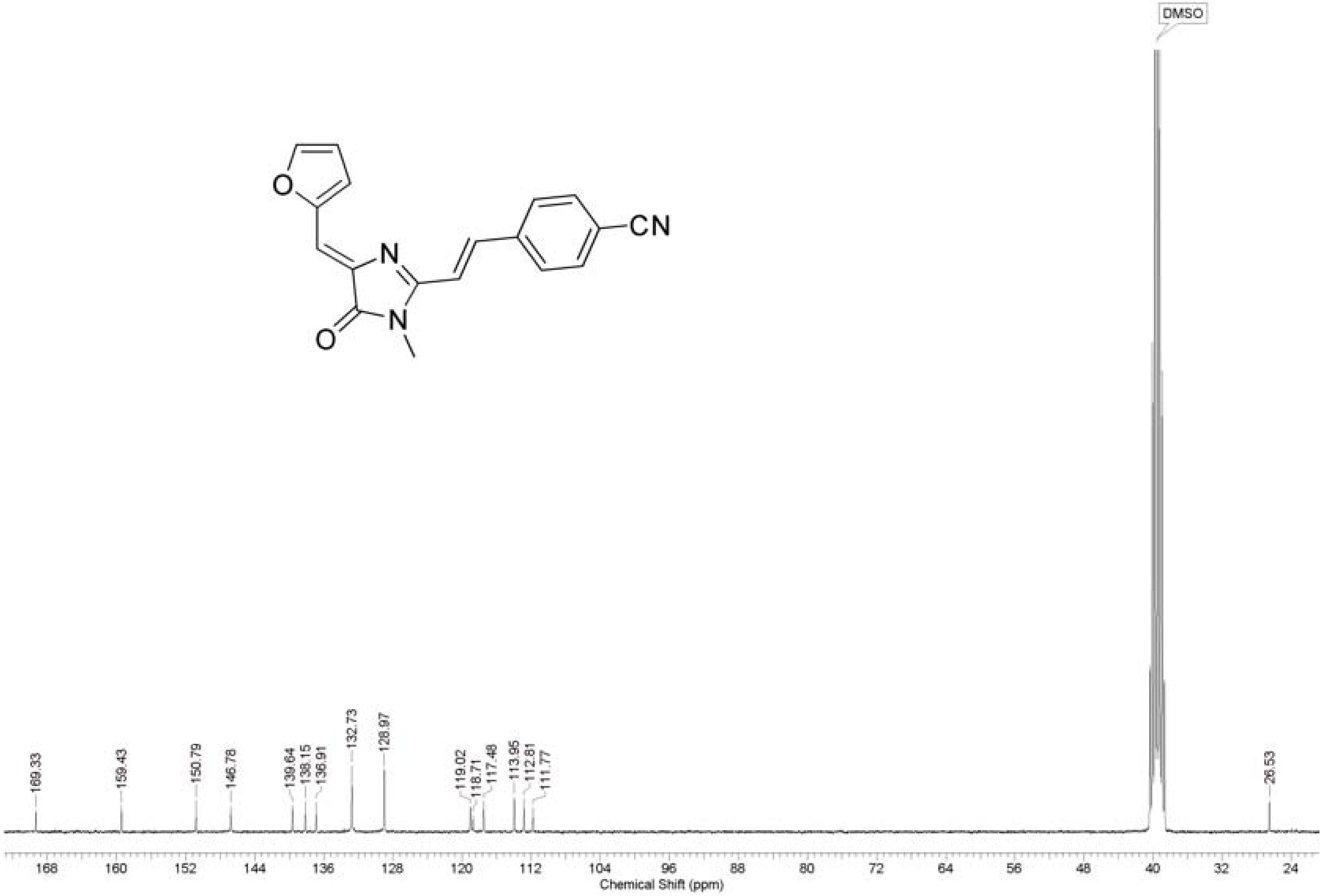
DMSO
CN
N
O
169.33
159.43
150.79
146.78
139.64
138.15
136.91
132.73
128.97
119.02
118.71
117.48
113.95
112.81
111.77
26.53
168
160
152
144
136
128
120
112
104
96
88
80
72
64
56
48
40
32
24
Chemical Shift (ppm)

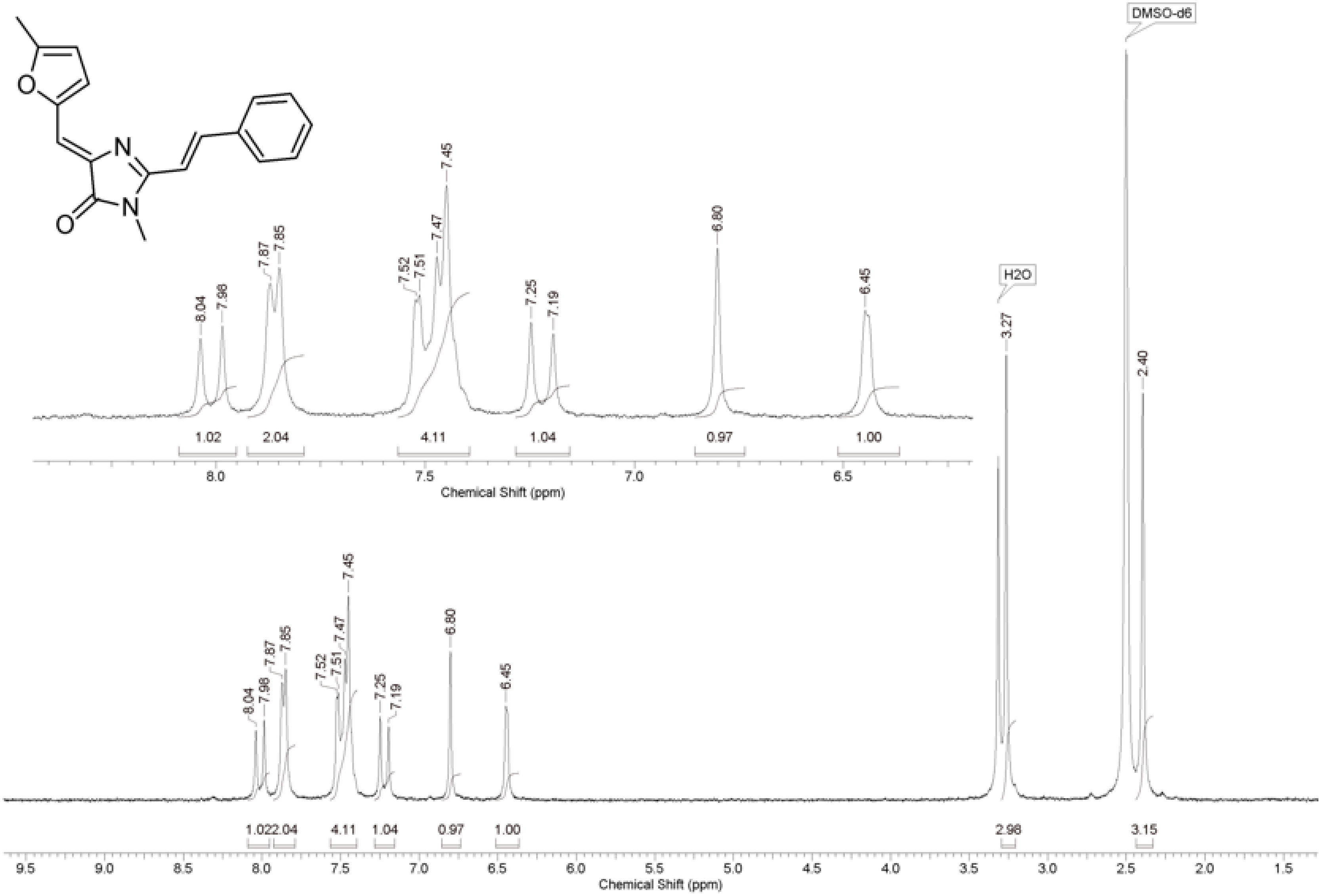

DMSO-d6
H2O
3.27
2.40
8.04
7.98
7.87
7.85
7.52
7.51
7.47
7.45
7.25
7.19
6.80
6.45
1.02
2.04
4.11
1.04
0.97
1.00
2.98
3.15
Chemical Shift (ppm)

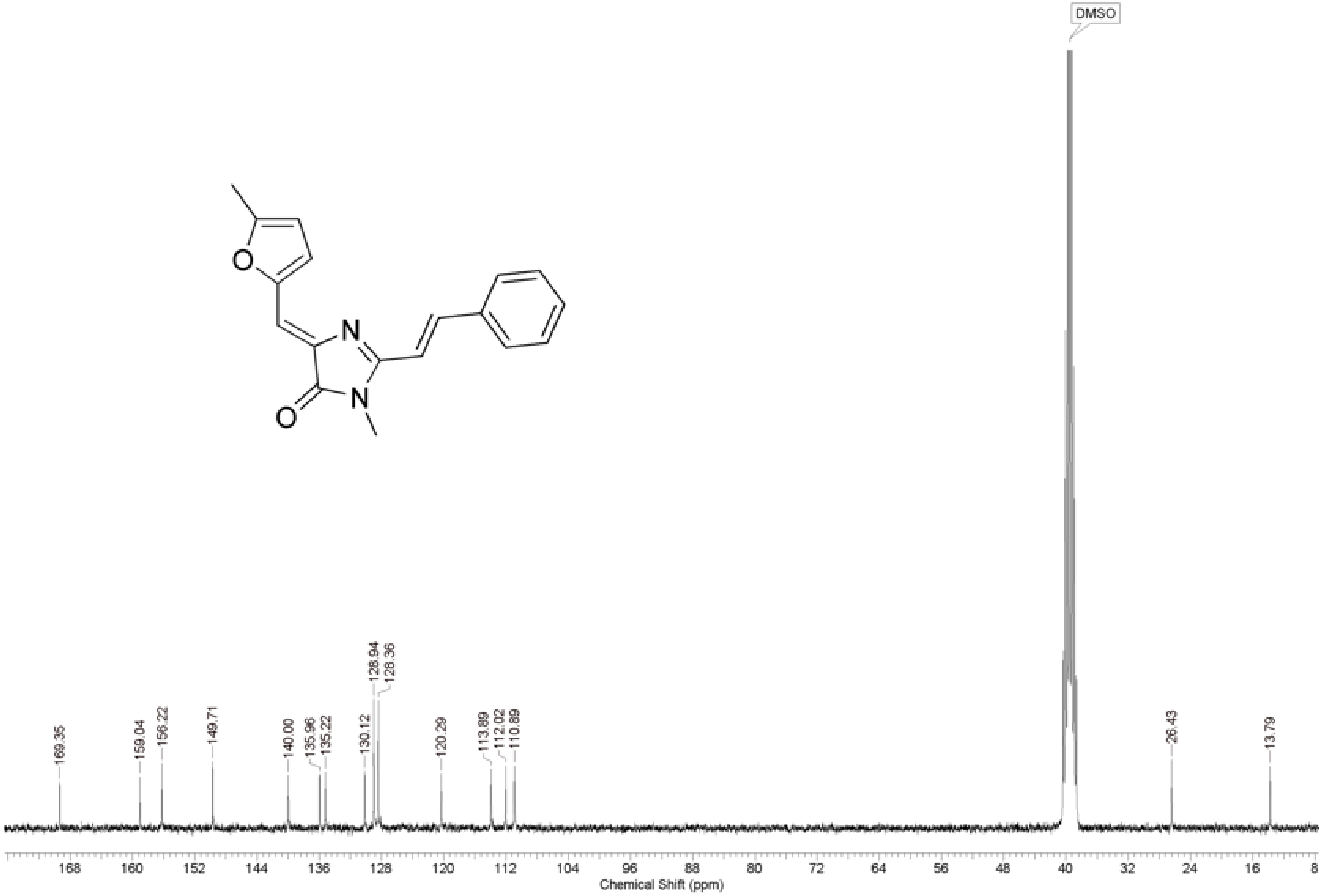
DMSO
169.35
159.04
156.22
149.71
140.00
135.96
135.22
130.12
128.94
128.36
120.29
113.89
112.02
110.89
26.43
13.79
168 160 152 144 136 128 120 112 104 96 88 80 72 64 56 48 40 32 24 16 8
Chemical Shift (ppm)
O
N
N
O

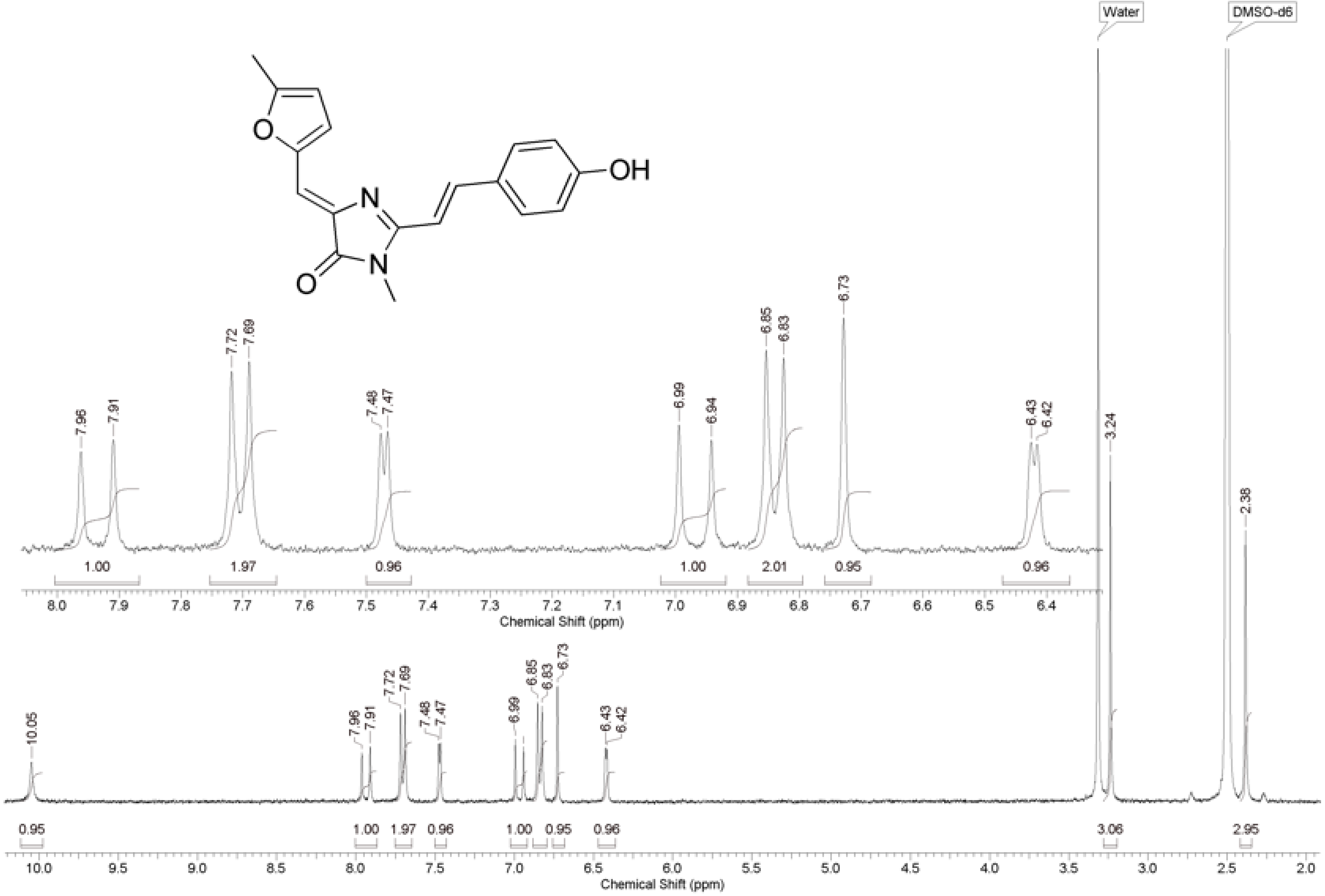

O
N
N
OH
O
Water
DMSO-d6
7.96
7.91
7.72
7.69
7.48
7.47
6.99
6.94
6.85
6.83
6.73
6.43
6.42
3.24
2.38
10.05
1.00
1.97
0.96
1.00
2.01
0.95
0.96
0.95
3.06
2.95
Chemical Shift (ppm)
Chemical Shift (ppm)

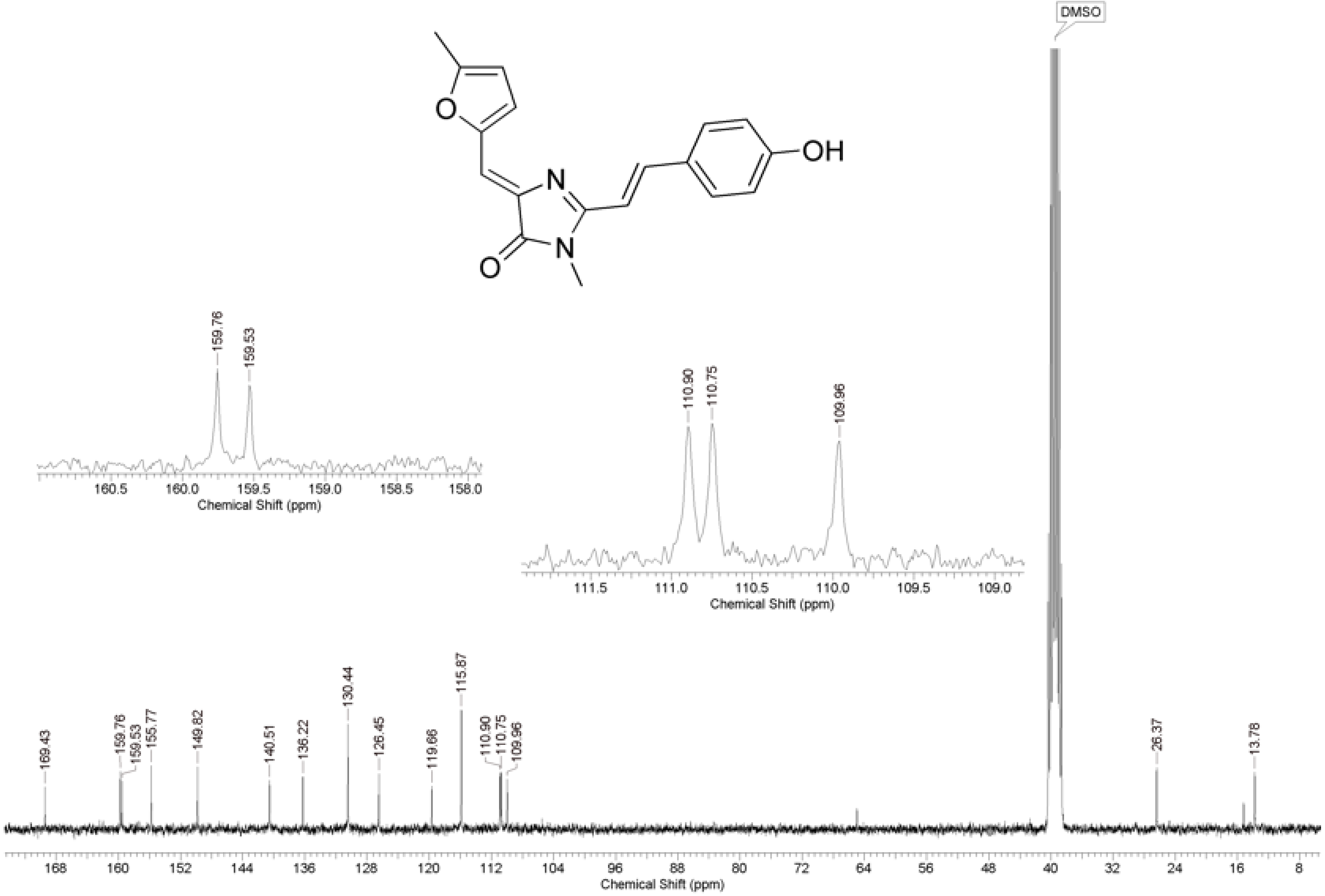
OH
N
N
O
O
DMSO
159.76
159.53
160.5
160.0
159.5
159.0
158.5
158.0
Chemical Shift (ppm)
110.90
110.75
109.96
111.5
111.0
110.5
110.0
109.5
109.0
Chemical Shift (ppm)
169.43
159.76
159.53
155.77
149.82
140.51
136.22
130.44
126.45
119.66
115.87
110.90
110.75
109.96
26.37
13.78
168
160
152
144
136
128
120
112
104
96
88
80
72
64
56
48
40
32
24
16
8
Chemical Shift (ppm)

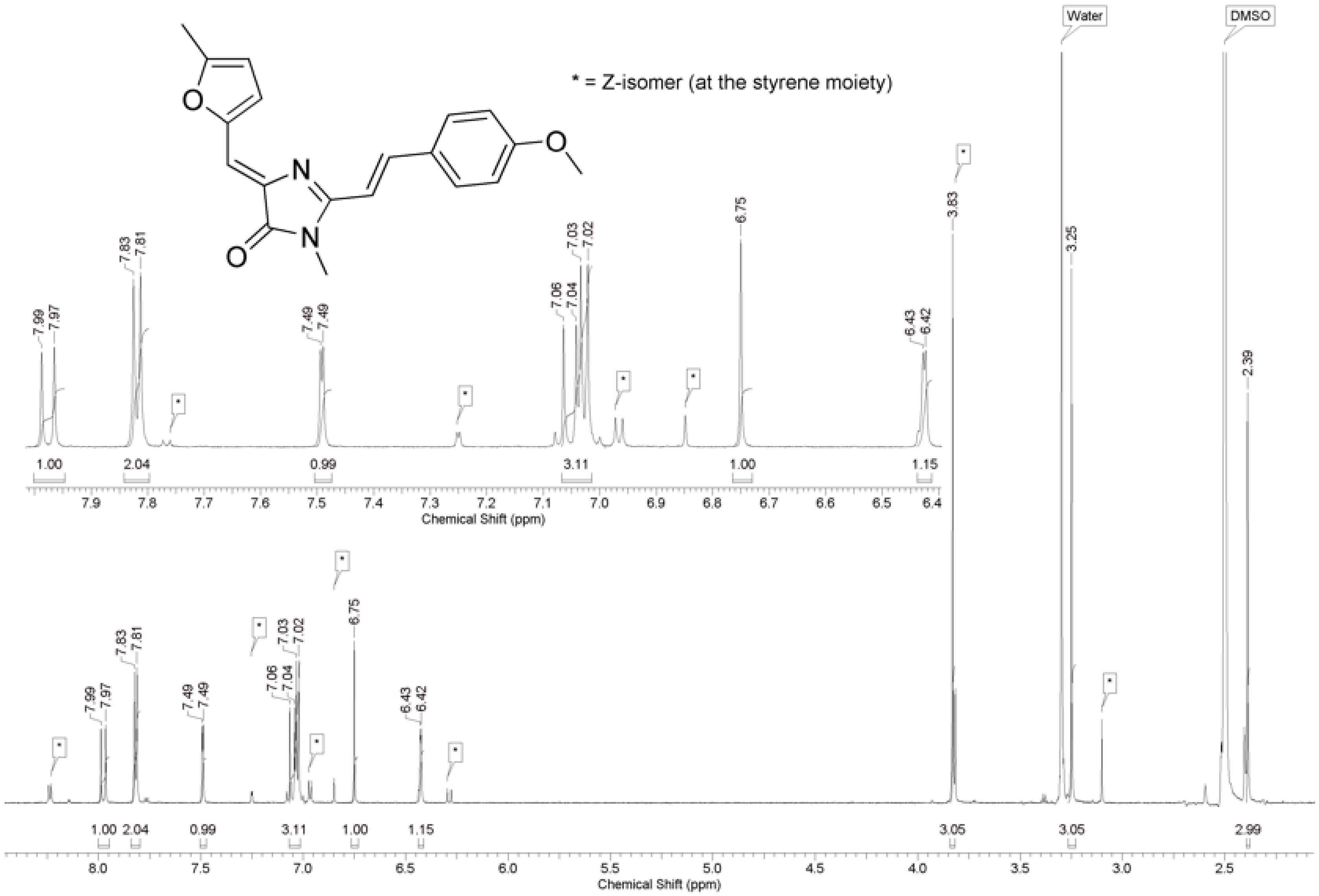
* = Z-isomer (at the styrene moiety)
Water
DMSO
N
N
O
O
Chemical Shift (ppm)
Chemical Shift (ppm)

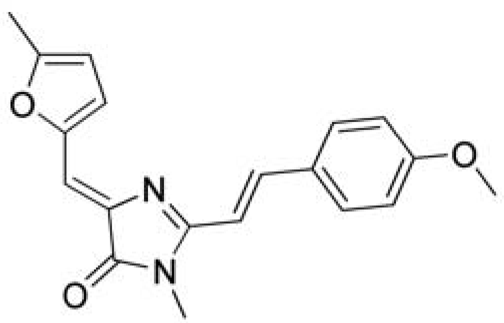

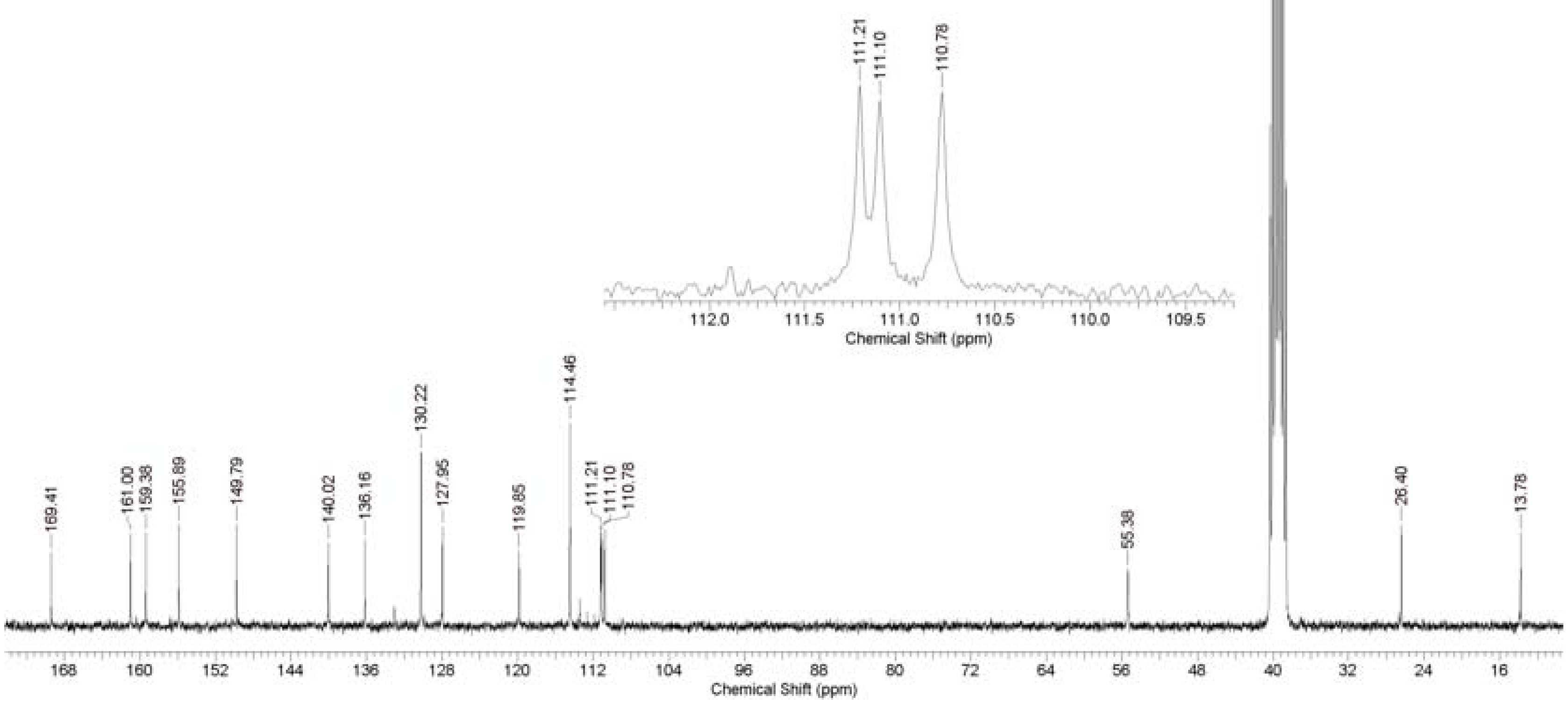
DMSO
111.21
111.10
110.78
112.0
111.5
111.0
110.5
110.0
109.5
Chemical Shift (ppm)
169.41
161.00
159.38
155.89
149.79
140.02
136.16
130.22
127.95
119.85
114.46
111.21
111.10
110.78
55.38
26.40
13.78
168
160
152
144
136
128
120
112
104
96
88
80
72
64
56
48
40
32
24
16
Chemical Shift (ppm)

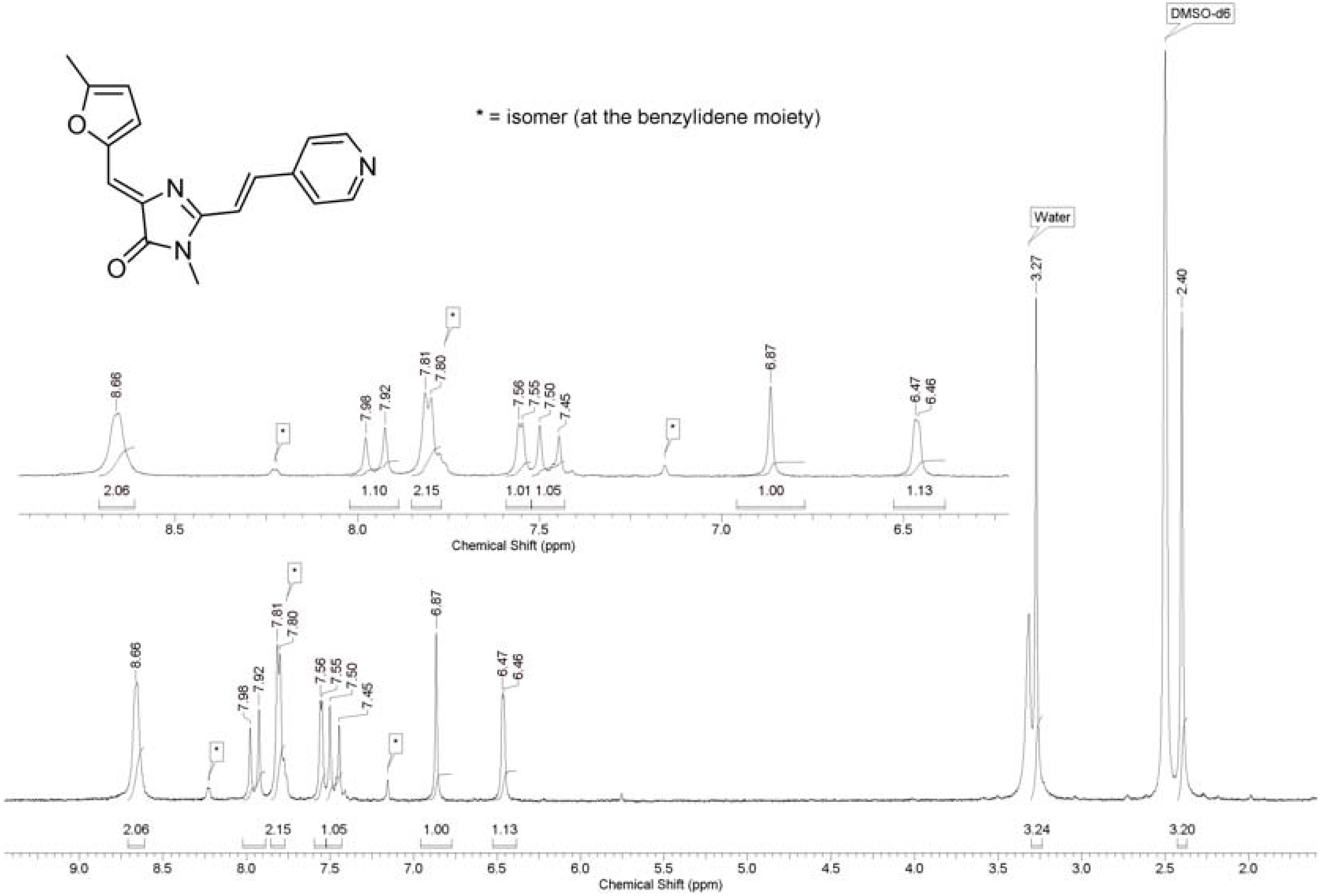

* = isomer (at the benzylidene moiety)
DMSO-d6
Water
8.66
7.98
7.92
7.81
7.80
7.56
7.55
7.50
7.45
6.87
6.47
6.46
3.27
2.40
2.06
1.10
2.15
1.01
1.05
1.00
1.13
3.24
3.20
Chemical Shift (ppm)

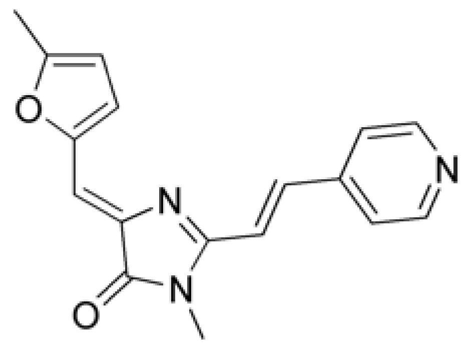

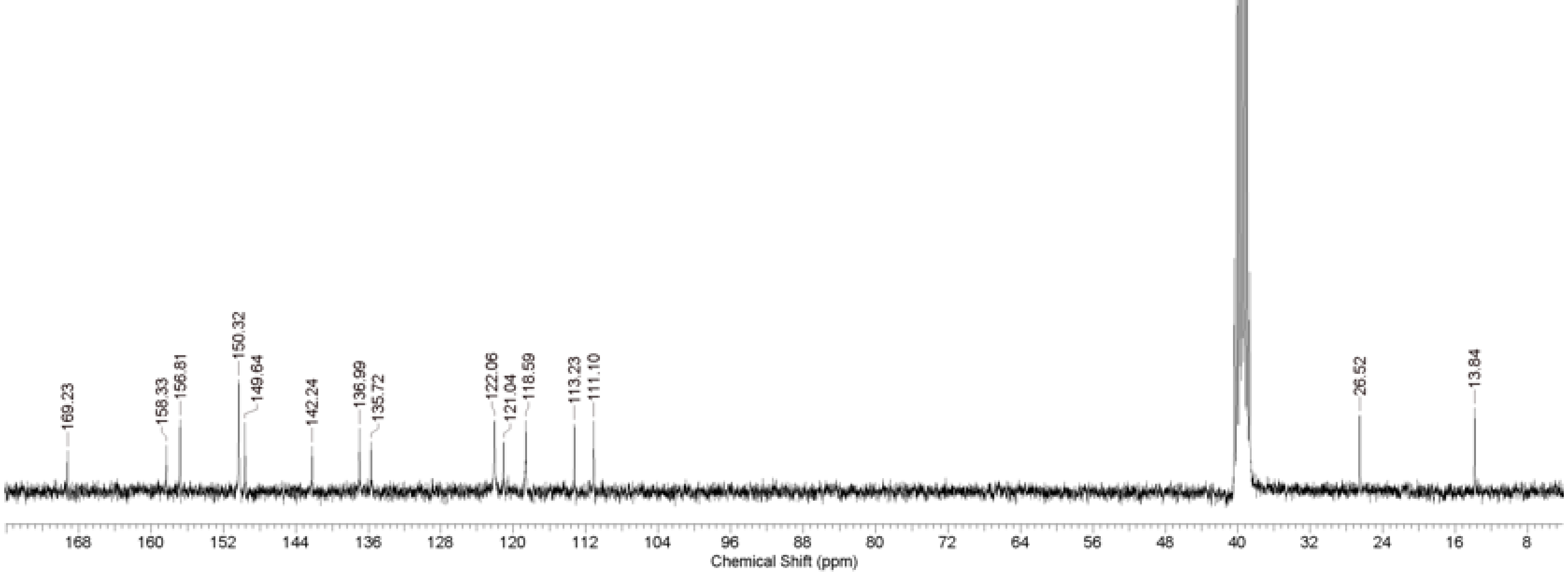
DMSO
169.23
158.33
156.81
150.32
149.64
142.24
136.99
135.72
122.06
121.04
118.59
113.23
111.10
26.52
13.84
168
160
152
144
136
128
120
112
104
96
88
80
72
64
56
48
40
32
24
16
8
Chemical Shift (ppm)

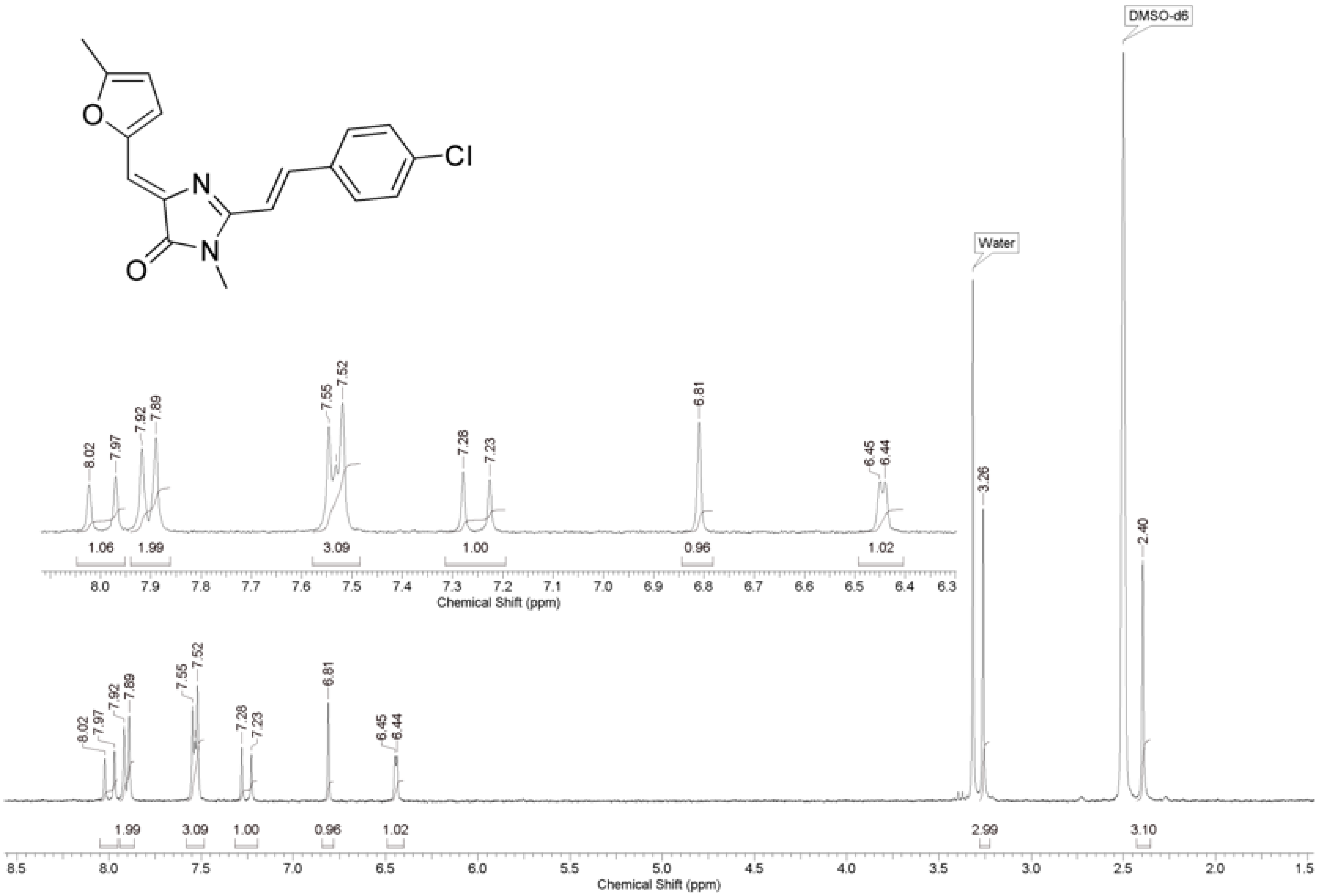

DMSO-d6
Water
O
N
Cl
8.02
7.97
7.92
7.89
7.55
7.52
7.28
7.23
6.81
6.45
6.44
3.26
2.40
1.06
1.99
3.09
1.00
0.96
1.02
2.99
3.10
Chemical Shift (ppm)
Chemical Shift (ppm)

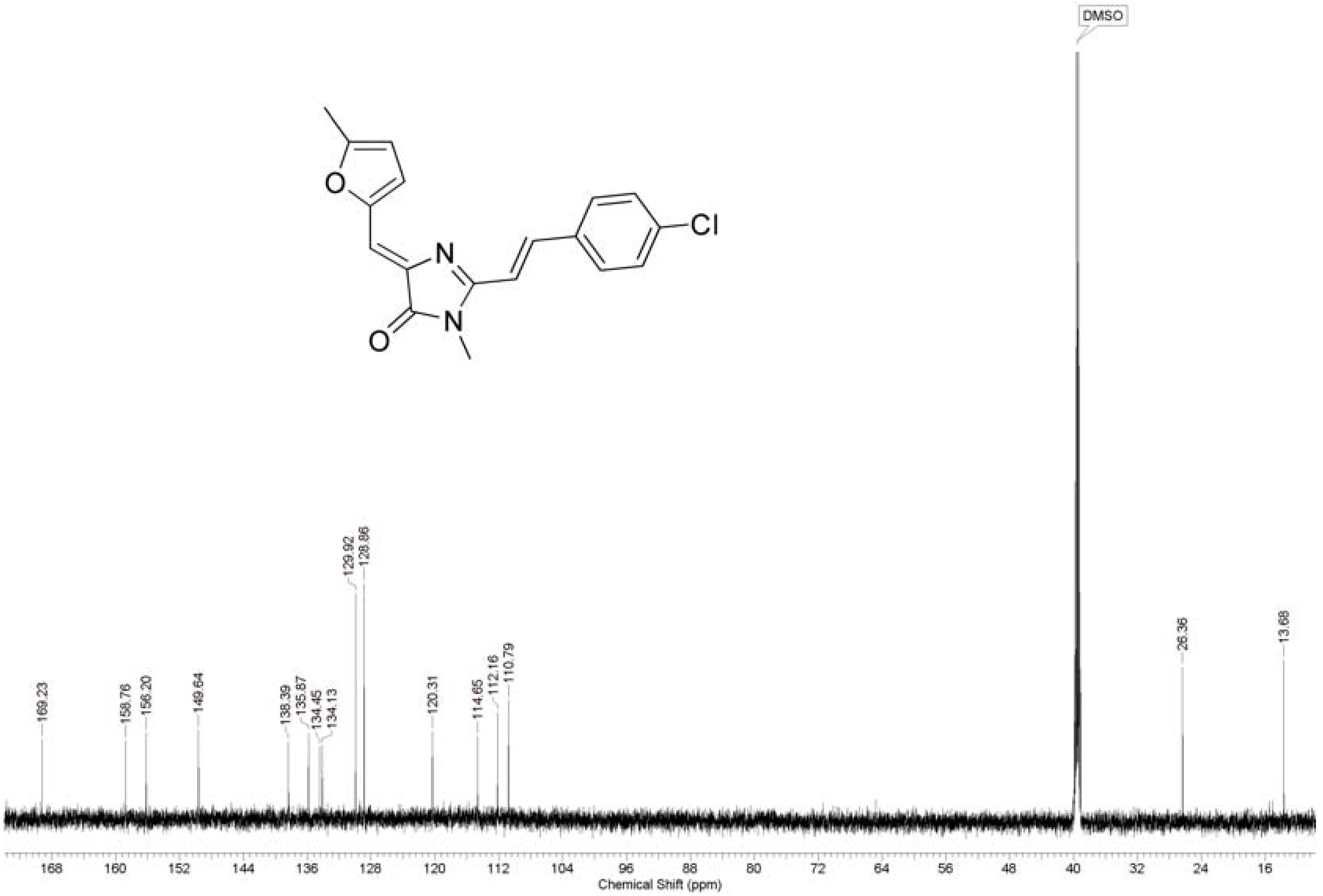

O
N
N
O
Cl
DMSO
169.23
158.76
156.20
149.64
138.39
135.87
134.45
134.13
129.92
128.86
120.31
114.65
112.16
110.79
26.36
13.68
168
160
152
144
136
128
120
112
104
96
88
80
72
64
56
48
40
32
24
16
Chemical Shift (ppm)

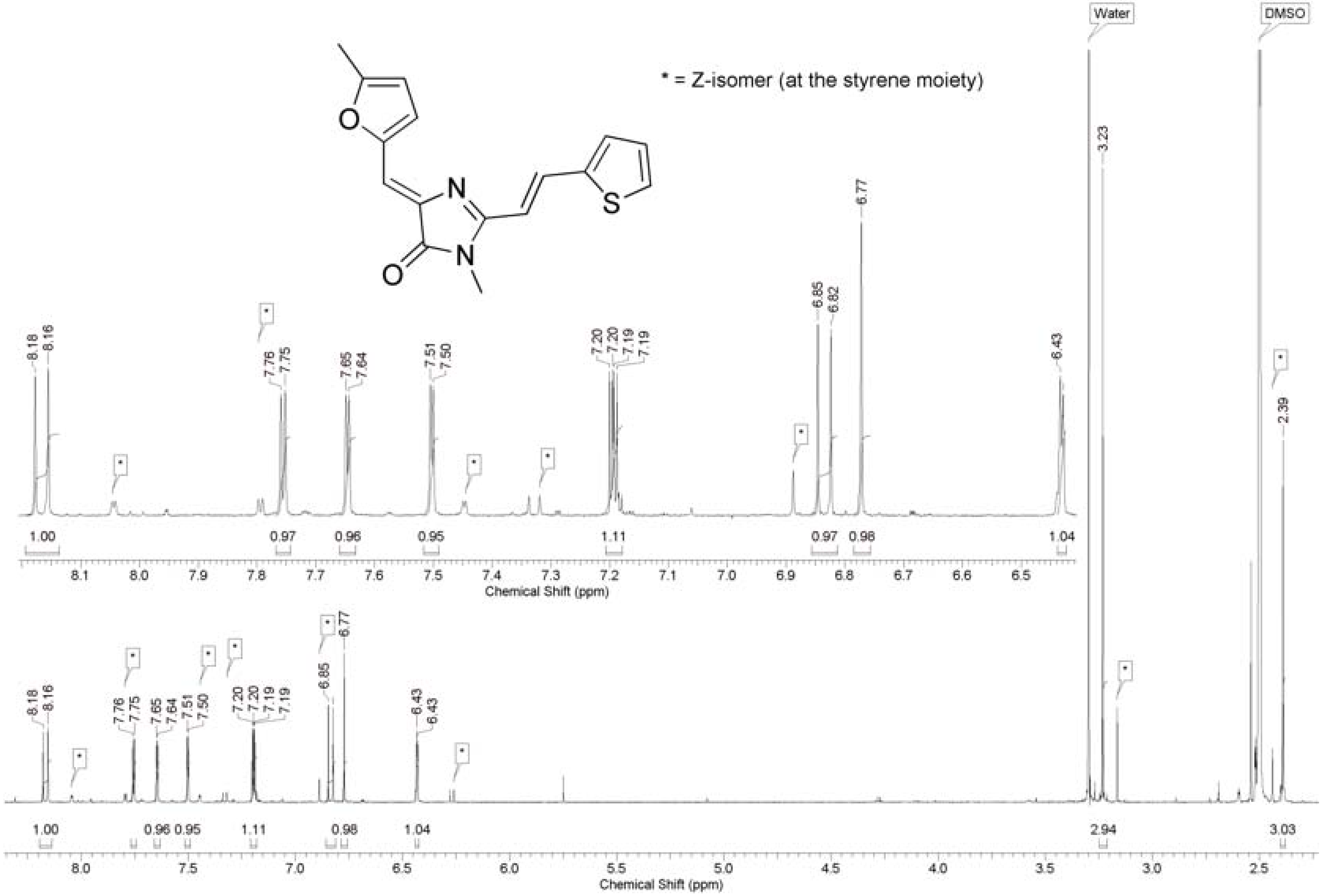
* = Z-isomer (at the styrene moiety)
Water
DMSO
Chemical Shift (ppm)
Chemical Shift (ppm)

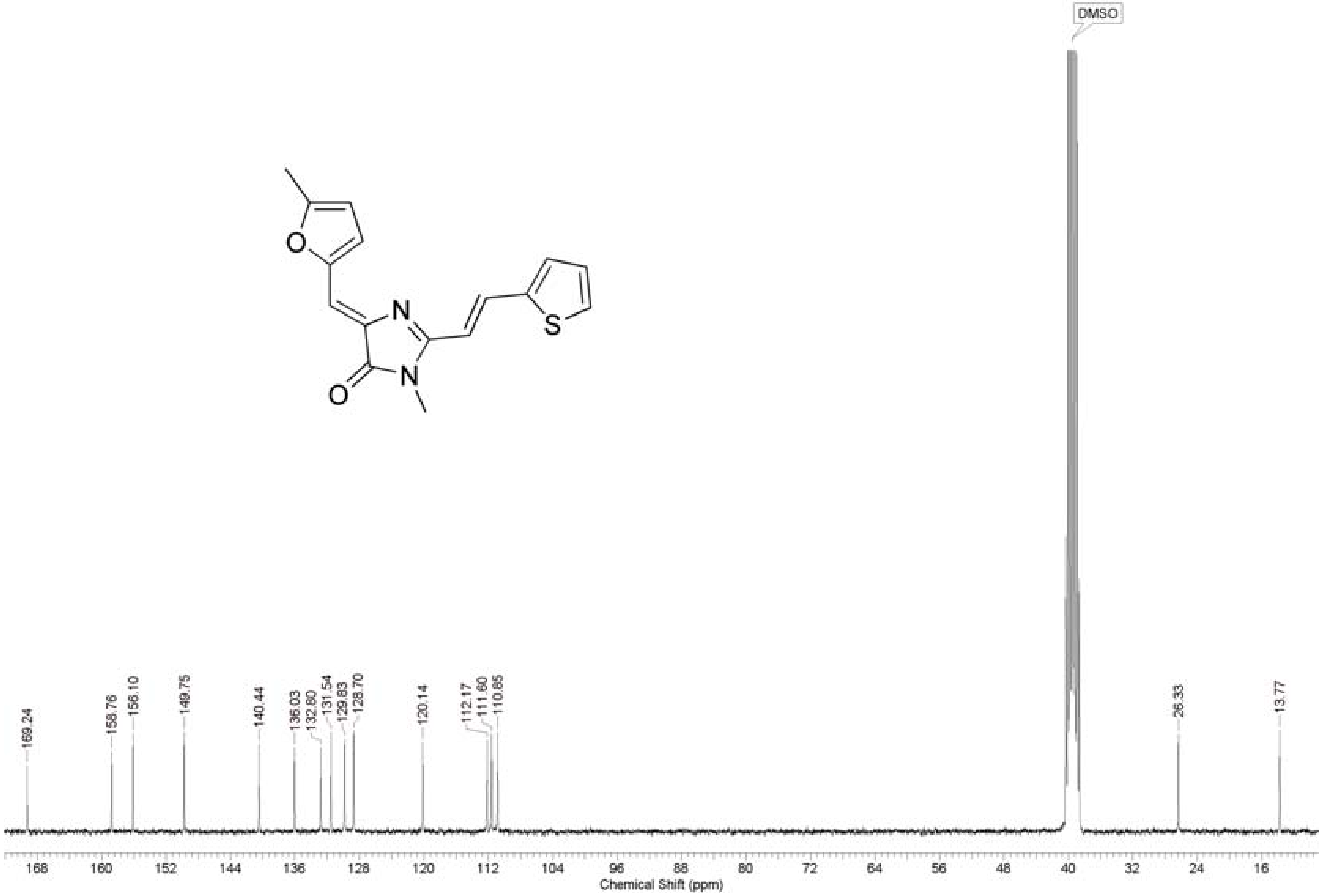
DMSO
169.24
158.76
156.10
149.75
140.44
136.03
132.80
131.54
129.83
128.70
120.14
112.17
111.60
110.85
26.33
13.77
O
N
N
S
O
168
160
152
144
136
128
120
112
104
96
88
80
72
64
56
48
40
32
24
16
Chemical Shift (ppm)

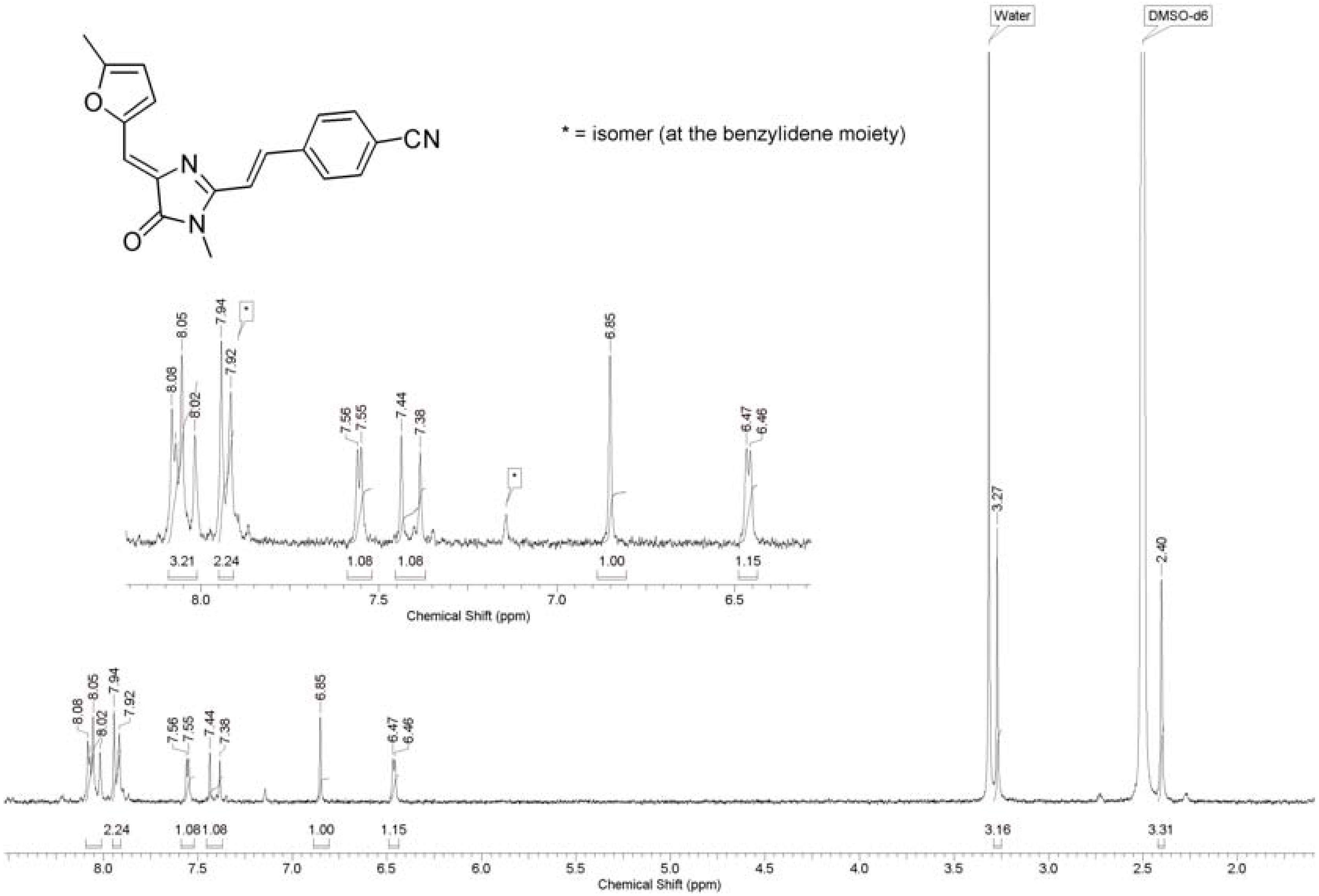

O
N
N
O
CN
* = isomer (at the benzylidene moiety)
Water
DMSO-d6
*
8.08
8.05
8.02
7.94
7.92
7.56
7.55
7.44
7.38
6.85
6.47
6.46
3.27
2.40
3.21
2.24
1.08
1.08
1.00
1.15
3.16
3.31
Chemical Shift (ppm)
Chemical Shift (ppm)

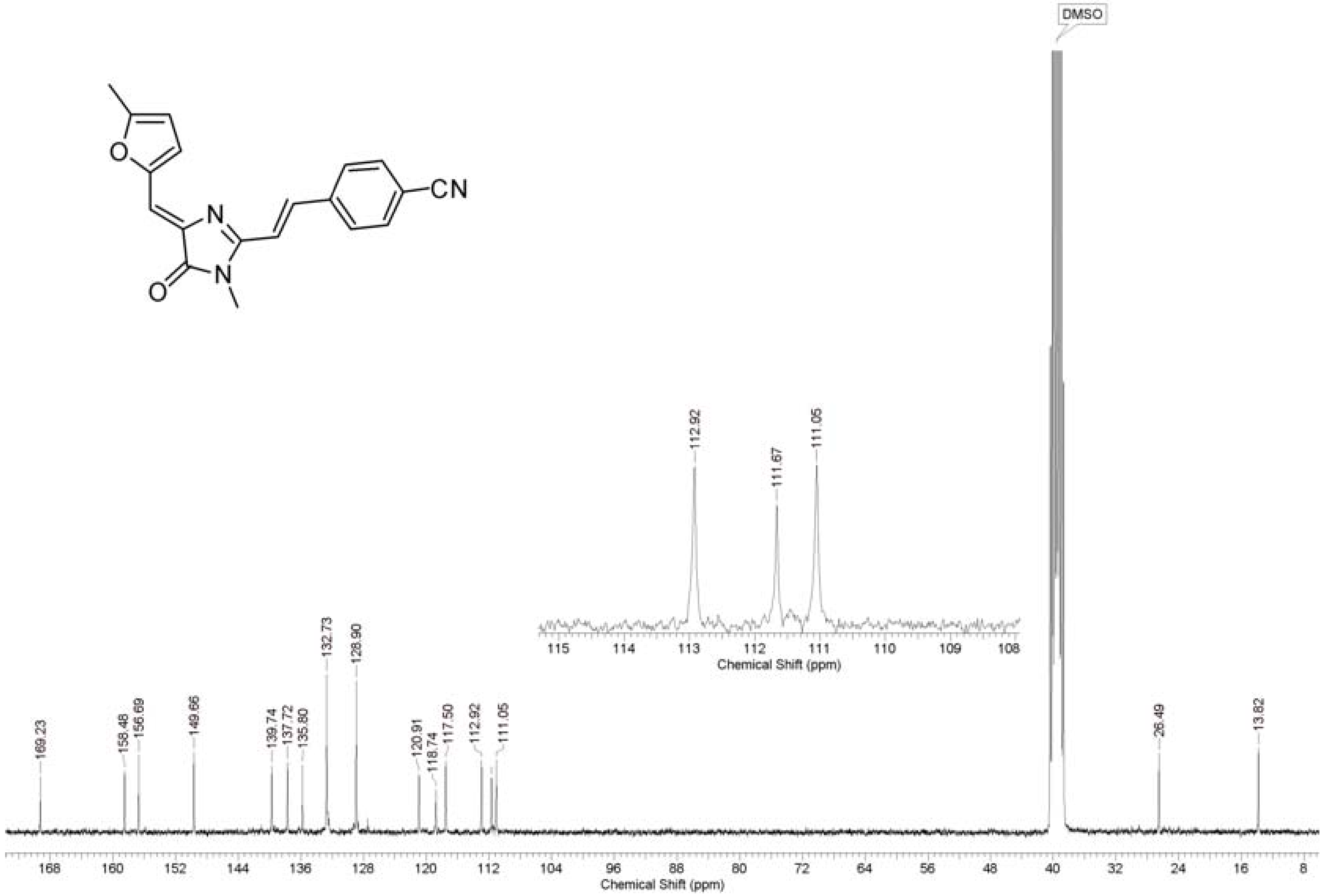
O
N
CN
O
N
DMSO
112.92
111.67
111.05
115
114
113
112
111
110
109
108
Chemical Shift (ppm)
169.23
158.48
156.69
149.66
139.74
137.72
135.80
132.73
128.90
120.91
118.74
117.50
112.92
111.05
26.49
13.82
168
160
152
144
136
128
120
112
104
96
88
80
72
64
56
48
40
32
24
16
8
Chemical Shift (ppm)

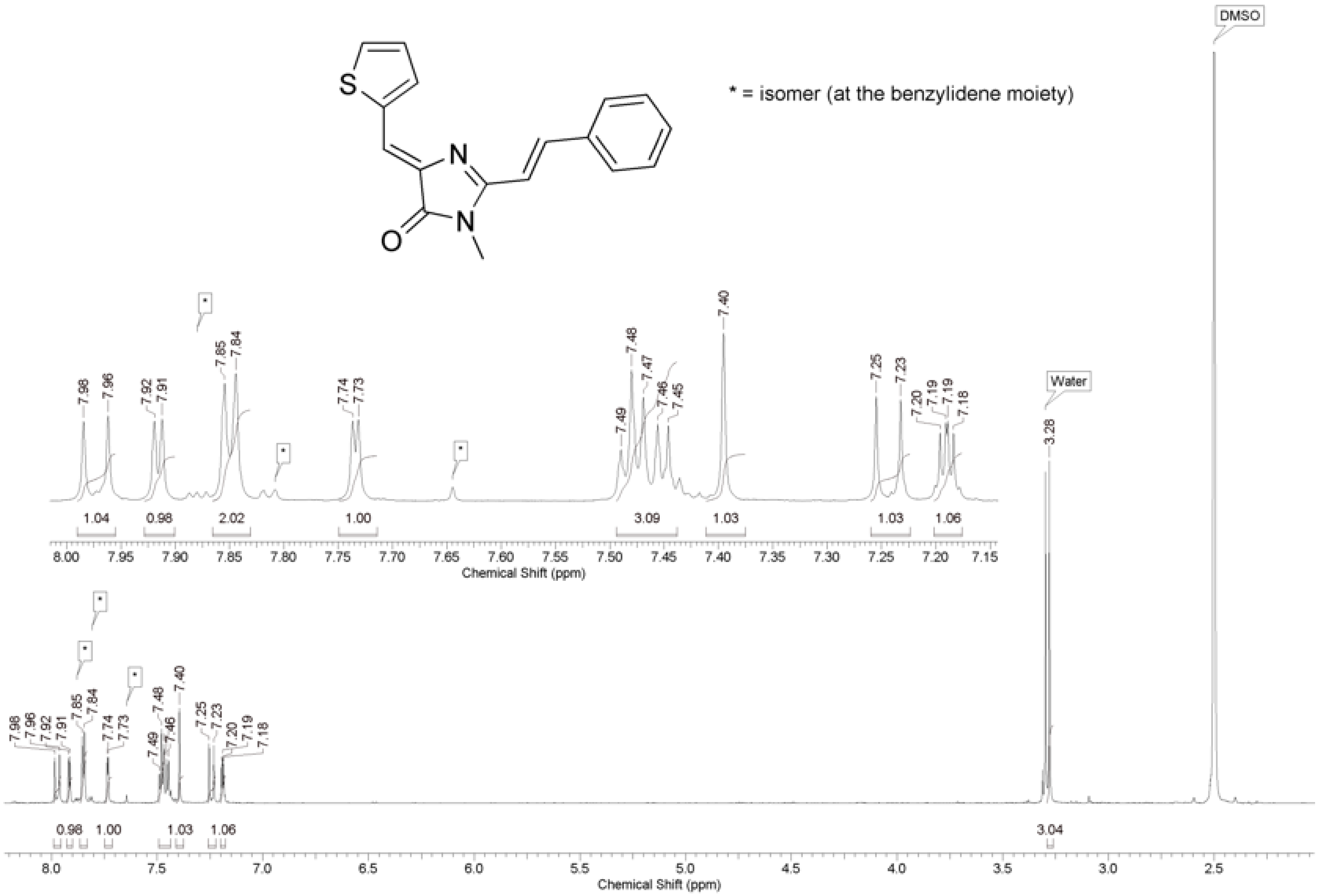
* = isomer (at the benzylidene moiety)
DMSO
Water
3.28
7.98 7.96 7.92 7.91 7.85 7.84 7.74 7.73 7.49 7.48 7.47 7.46 7.45 7.40 7.25 7.23 7.20 7.19 7.19 7.18
1.04 0.98 2.02 1.00 3.09 1.03 1.03 1.06
0.98 1.00 1.03 1.06 3.04
Chemical Shift (ppm)

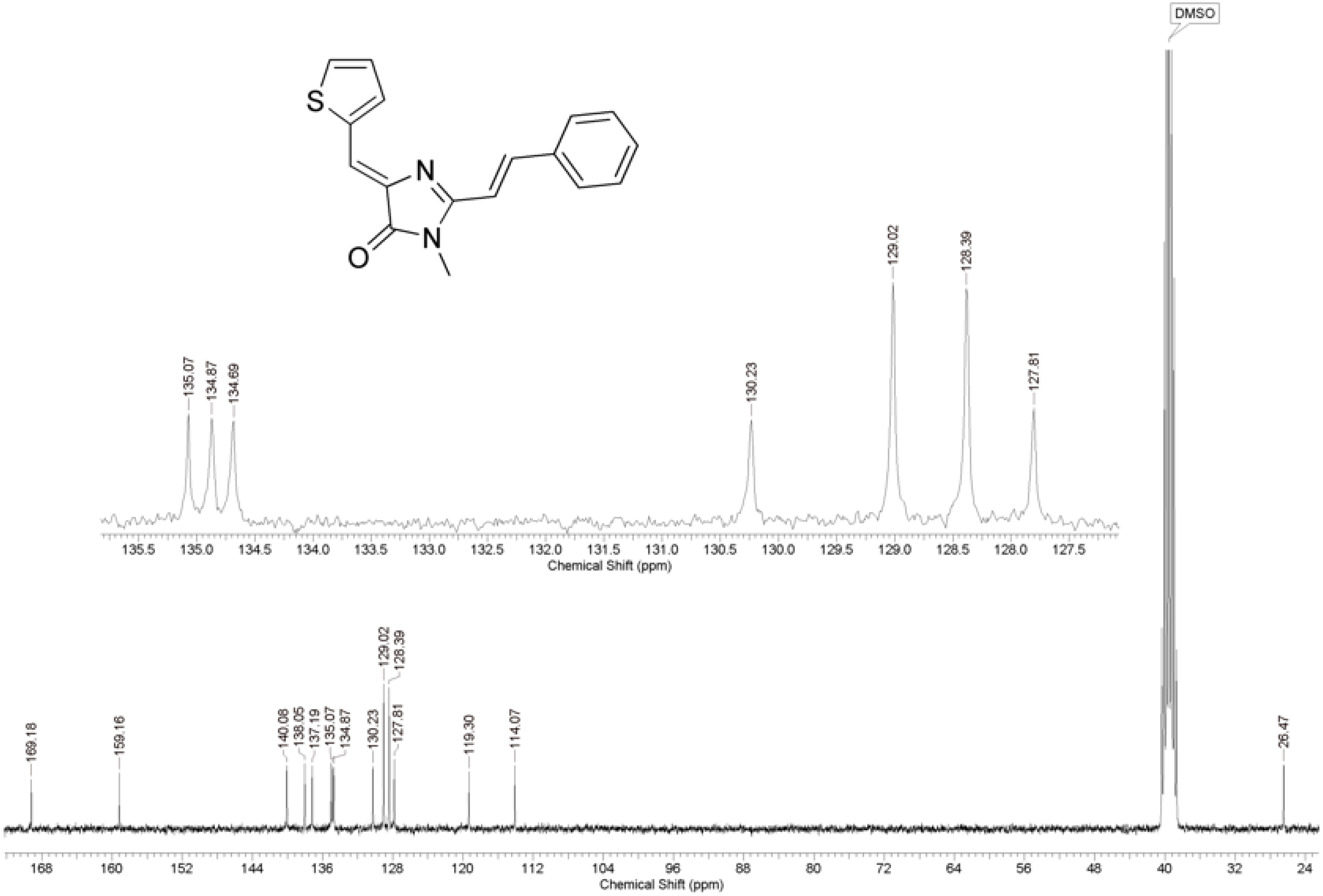
S
N
N
O
DMSO
135.07
134.87
134.69
130.23
129.02
128.39
127.81
Chemical Shift (ppm)
169.18
159.16
140.08
138.05
137.19
135.07
134.87
130.23
129.02
128.39
127.81
119.30
114.07
26.47
Chemical Shift (ppm)

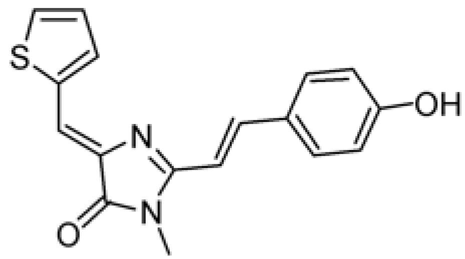
S
N
N
O
OH

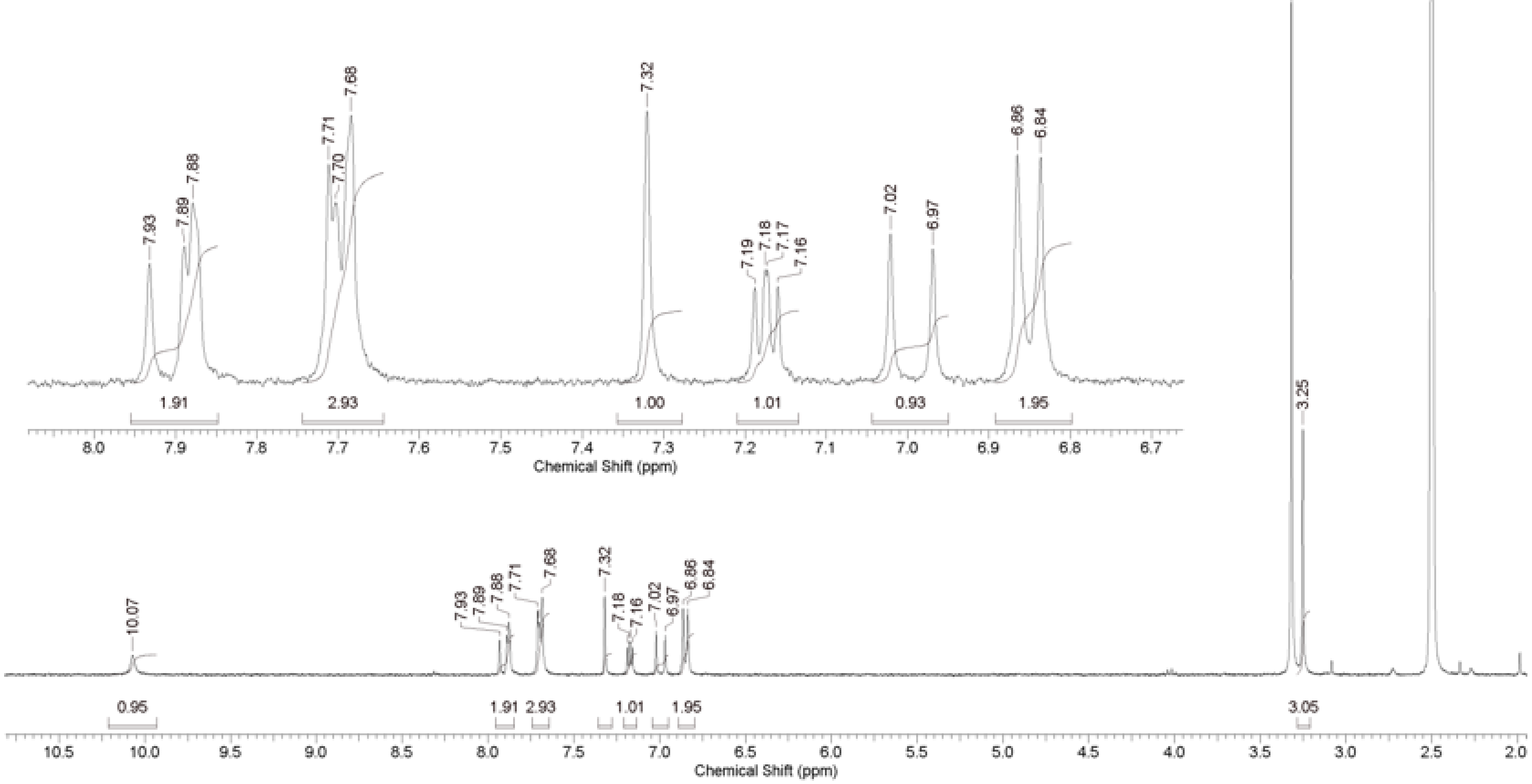
DMSO-d6
H2O
7.93
7.89
7.88
7.71
7.70
7.68
7.32
7.19
7.18
7.17
7.16
7.02
6.97
6.86
6.84
1.91
2.93
1.00
1.01
0.93
1.95
Chemical Shift (ppm)
10.07
3.25
0.95
3.05
Chemical Shift (ppm)

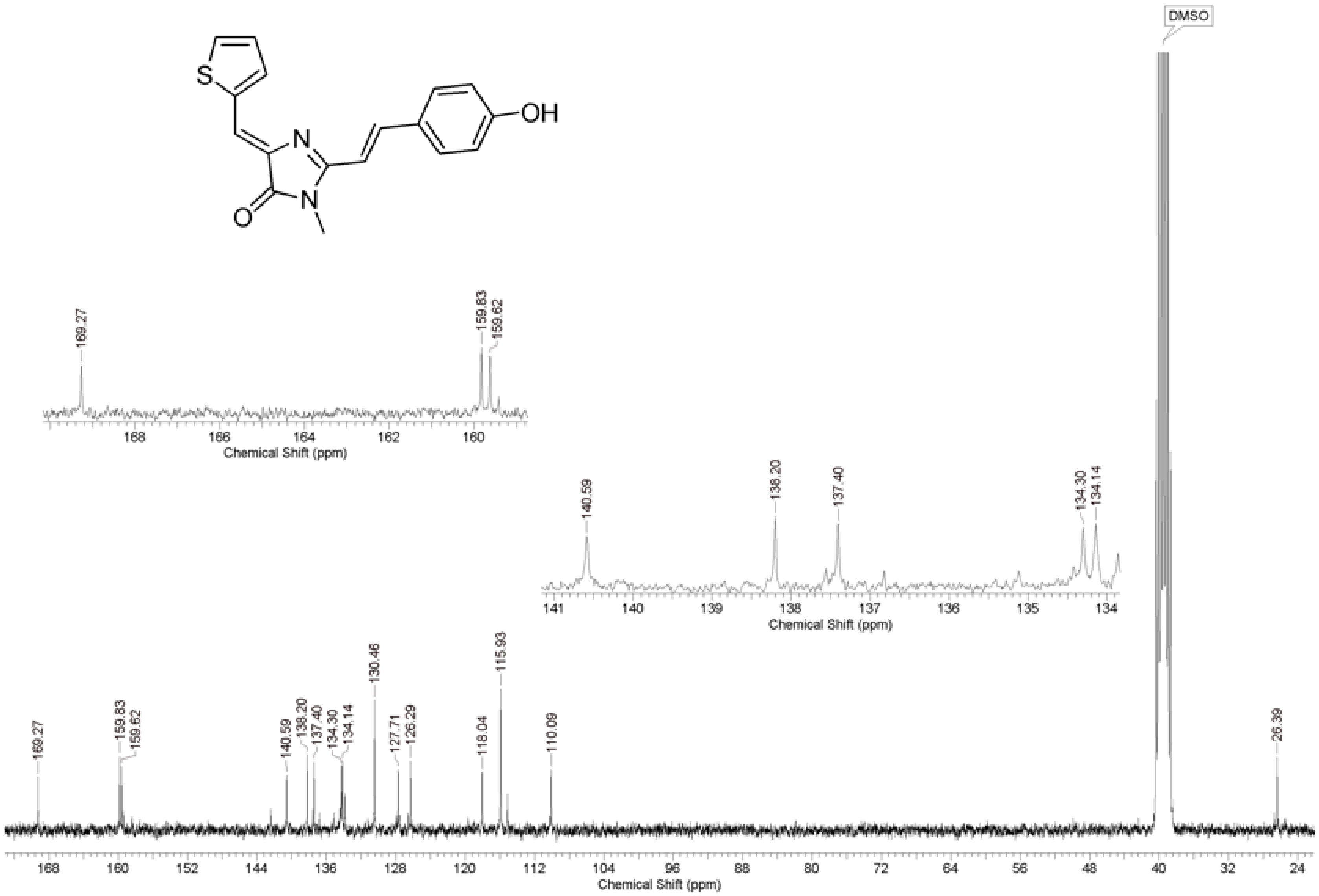

S
N
N
O
OH
DMSO
169.27
159.83
159.62
168
166
164
162
160
Chemical Shift (ppm)
140.59
138.20
137.40
134.30
134.14
141
140
139
138
137
136
135
134
Chemical Shift (ppm)
130.46
127.71
126.29
118.04
115.93
110.09
26.39
168
160
152
144
136
128
120
112
104
96
88
80
72
64
56
48
40
32
24
Chemical Shift (ppm)

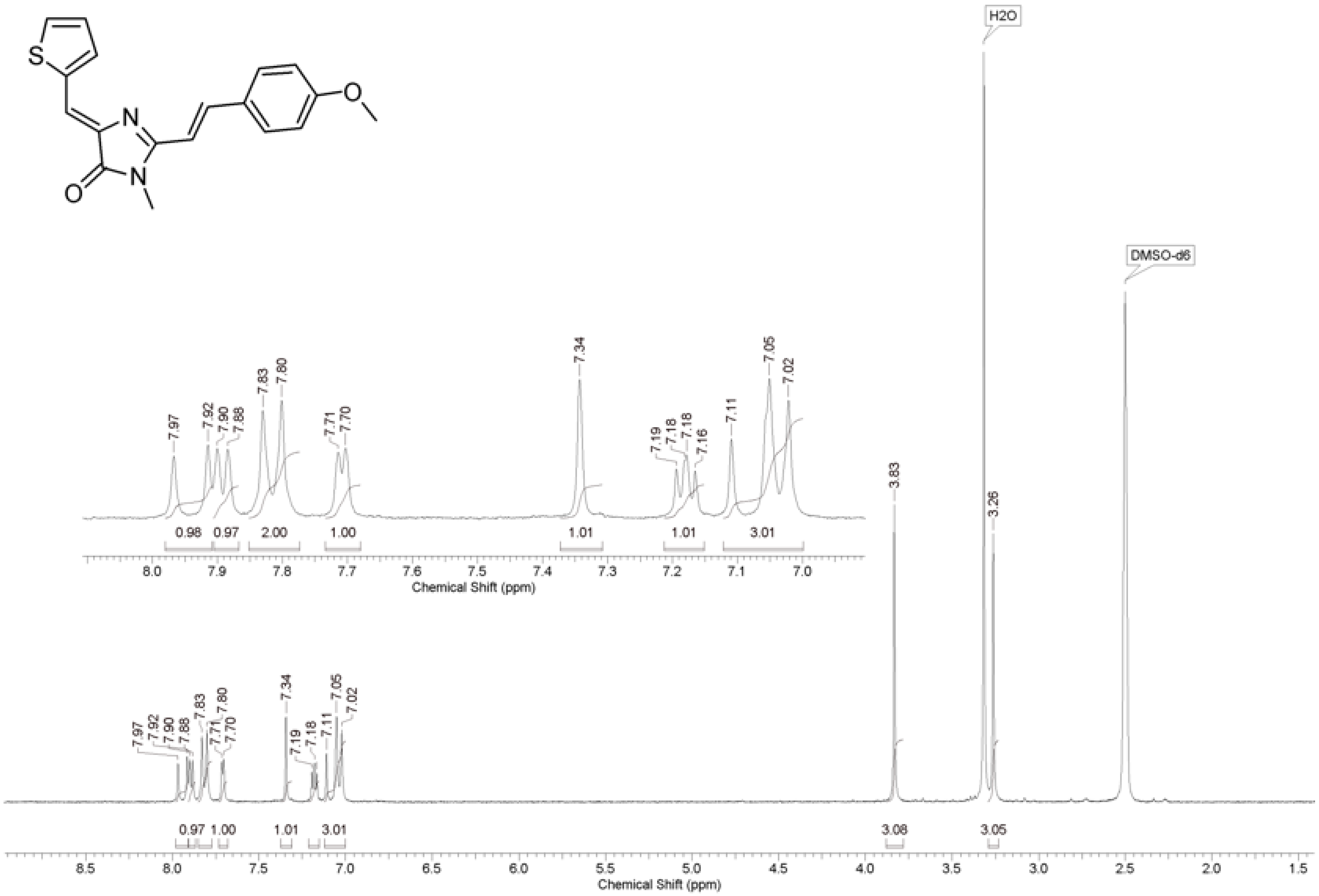

S
N
N
O
H2O
DMSO-d6
7.97
7.92
7.90
7.88
7.83
7.80
7.71
7.70
7.34
7.19
7.18
7.18
7.16
7.11
7.05
7.02
0.98
0.97
2.00
1.00
1.01
1.01
3.01
Chemical Shift (ppm)
3.83
3.26
0.97
1.00
1.01
3.01
3.08
3.05
Chemical Shift (ppm)

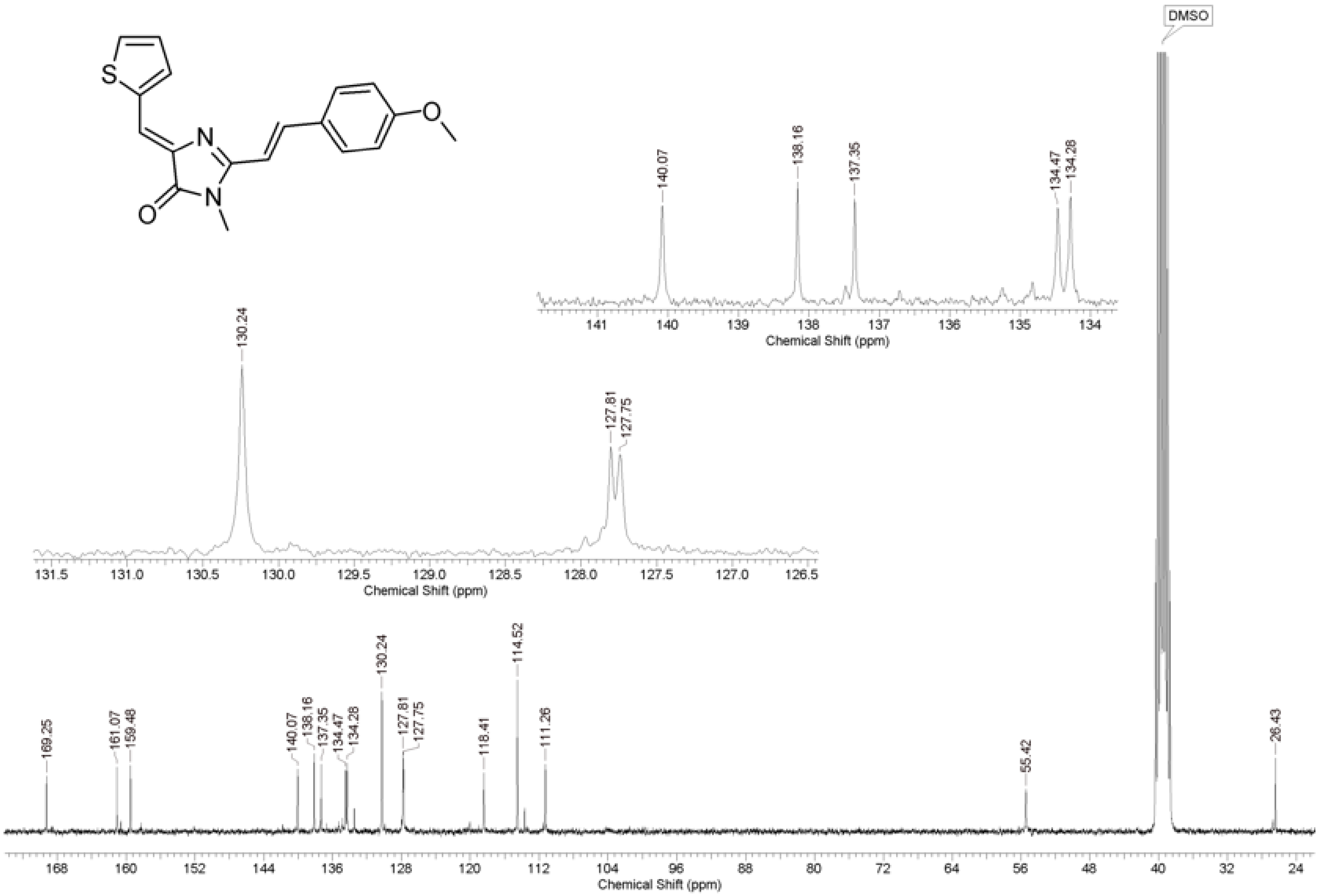

S
N
N
O
O
DMSO
140.07
138.16
137.35
134.47
134.28
Chemical Shift (ppm)
130.24
127.81
127.75
Chemical Shift (ppm)
169.25
161.07
159.48
140.07
138.16
137.35
134.47
134.28
130.24
127.81
127.75
118.41
114.52
111.26
55.42
26.43
Chemical Shift (ppm)

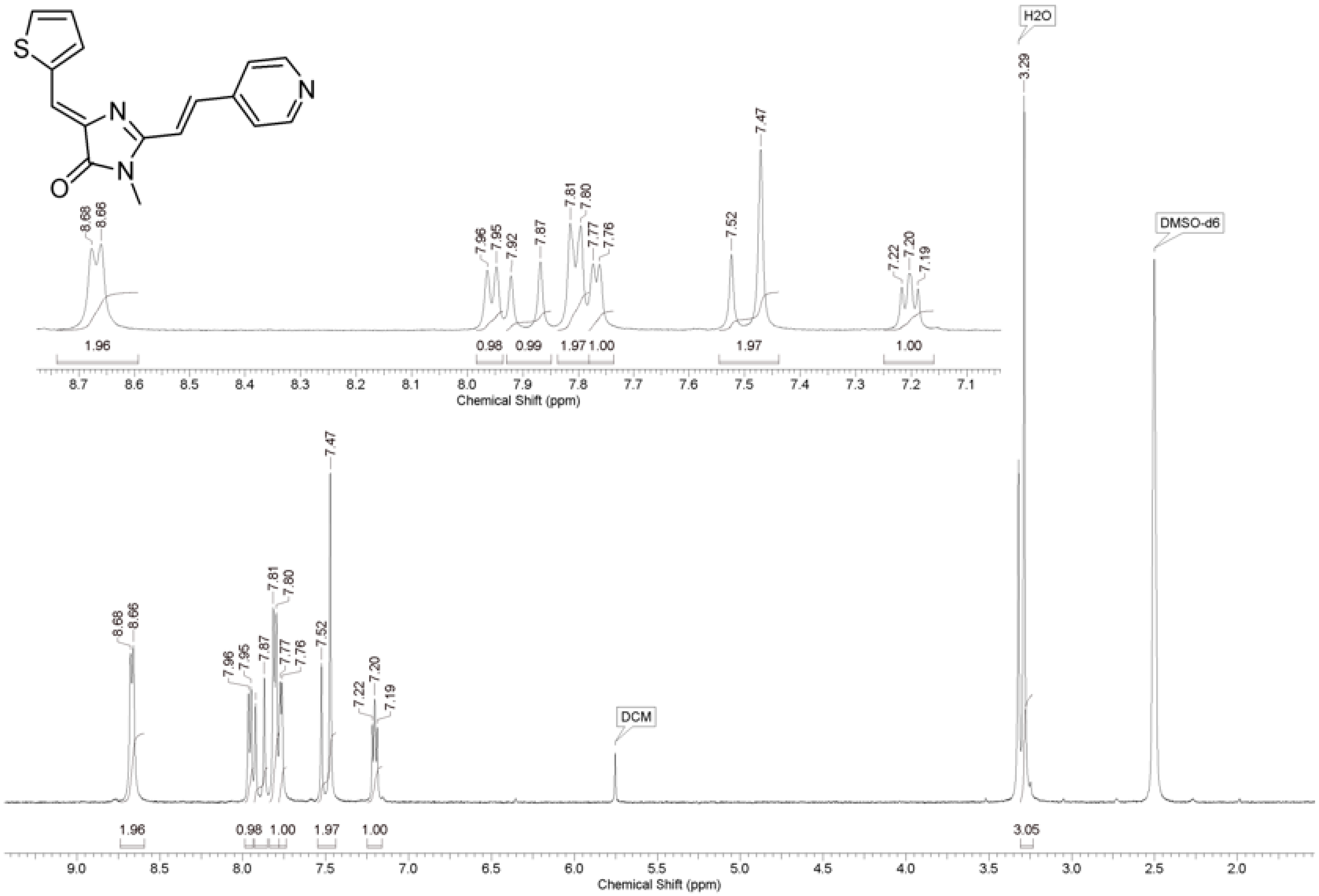

H2O
3.29
7.47
8.68
8.66
7.96
7.95
7.92
7.87
7.81
7.80
7.77
7.76
7.52
7.22
7.20
7.19
DMSO-d6
1.96
0.98
0.99
1.97
1.00
1.97
1.00
Chemical Shift (ppm)
DCM
3.05
Chemical Shift (ppm)

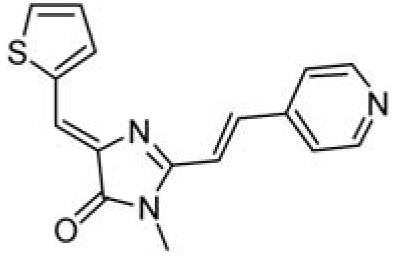

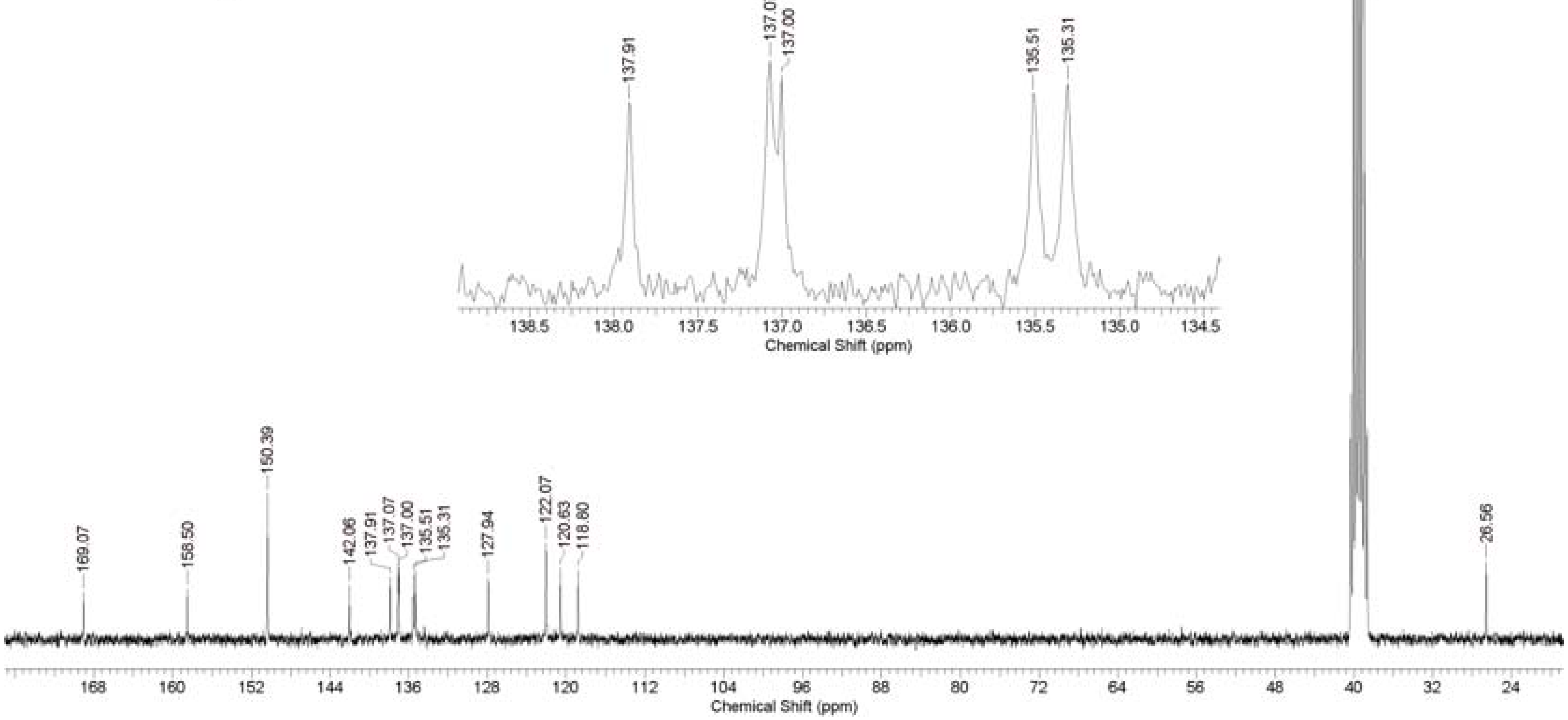
DMSO
137.91
137.07
137.00
135.51
135.31
Chemical Shift (ppm)
169.07
158.50
150.39
142.06
137.91
137.07
137.00
135.51
135.31
127.94
122.07
120.63
118.80
26.56
Chemical Shift (ppm)

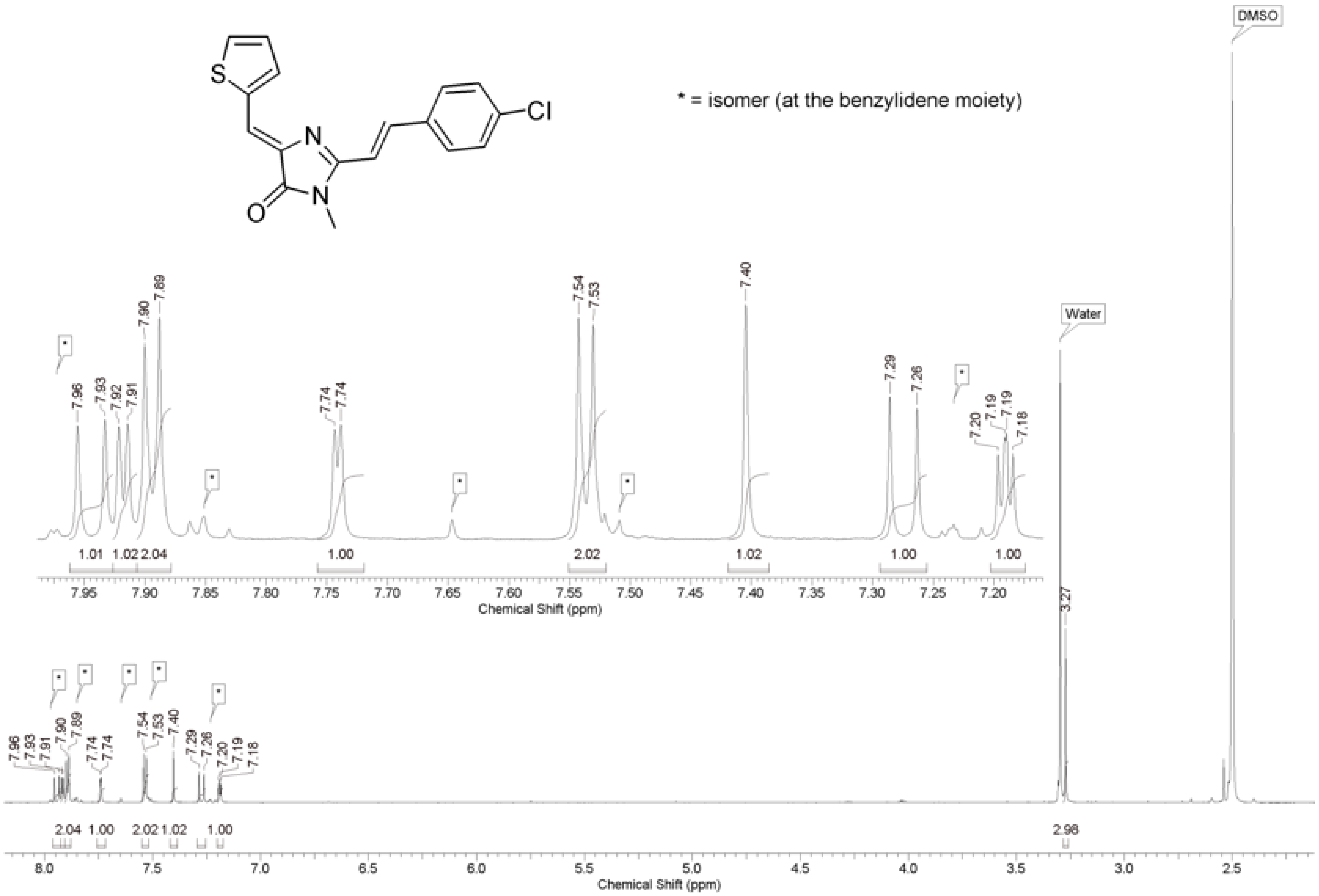

S
N
N
O
Cl
* = isomer (at the benzylidene moiety)
DMSO
Water
7.96
7.93
7.92
7.91
7.90
7.89
7.74
7.74
7.54
7.53
7.40
7.29
7.26
7.20
7.19
7.19
7.18
3.27
1.01
1.02
2.04
1.00
2.02
1.02
1.00
1.00
2.98
Chemical Shift (ppm)

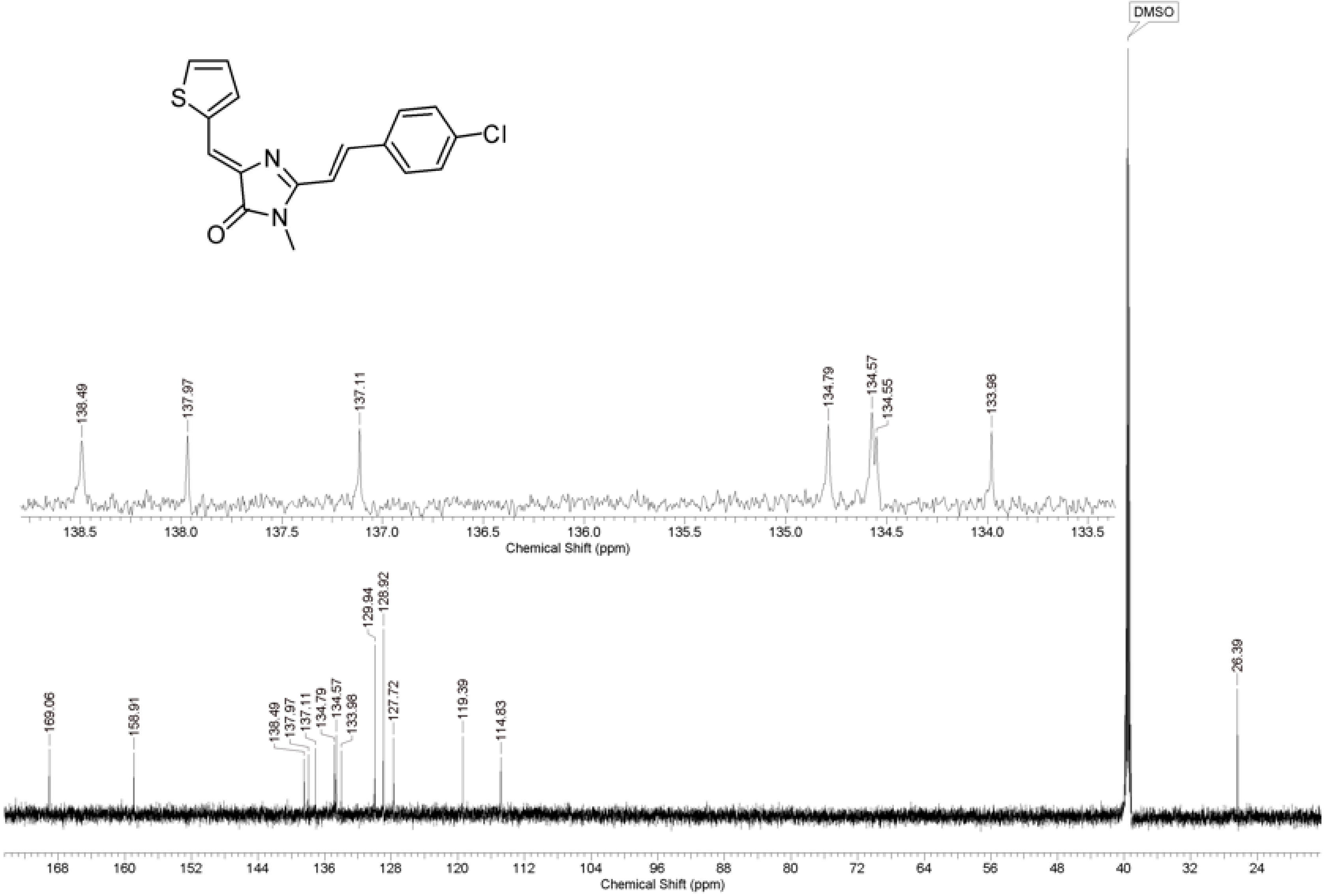
S
N
O
N
Cl
DMSO
138.49
137.97
137.11
134.79
134.57
134.55
133.98
Chemical Shift (ppm)
169.06
158.91
129.94
128.92
127.72
119.39
114.83
26.39
Chemical Shift (ppm)

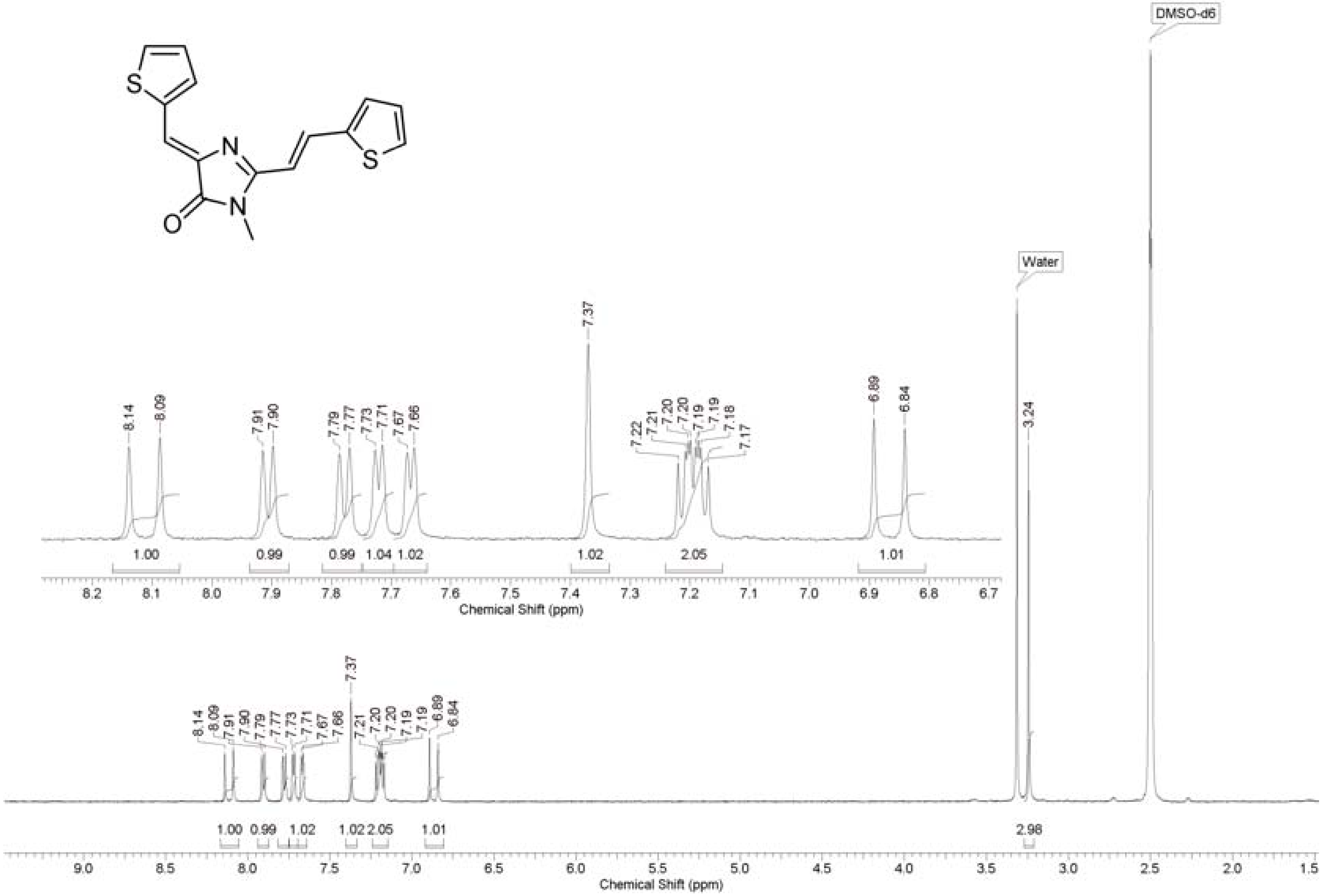
S
N
S
O
N
DMSO-d6
Water
8.14
8.09
7.91
7.90
7.79
7.77
7.73
7.71
7.67
7.66
7.37
7.22
7.21
7.20
7.20
7.19
7.19
7.18
7.17
6.89
6.84
3.24
1.00
0.99
0.99
1.04
1.02
1.02
2.05
1.01
2.98
Chemical Shift (ppm)

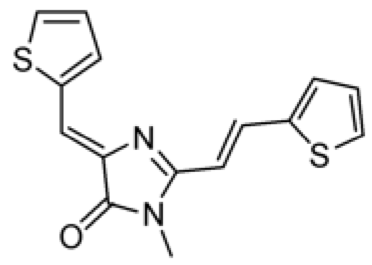
S
N
S
O
N

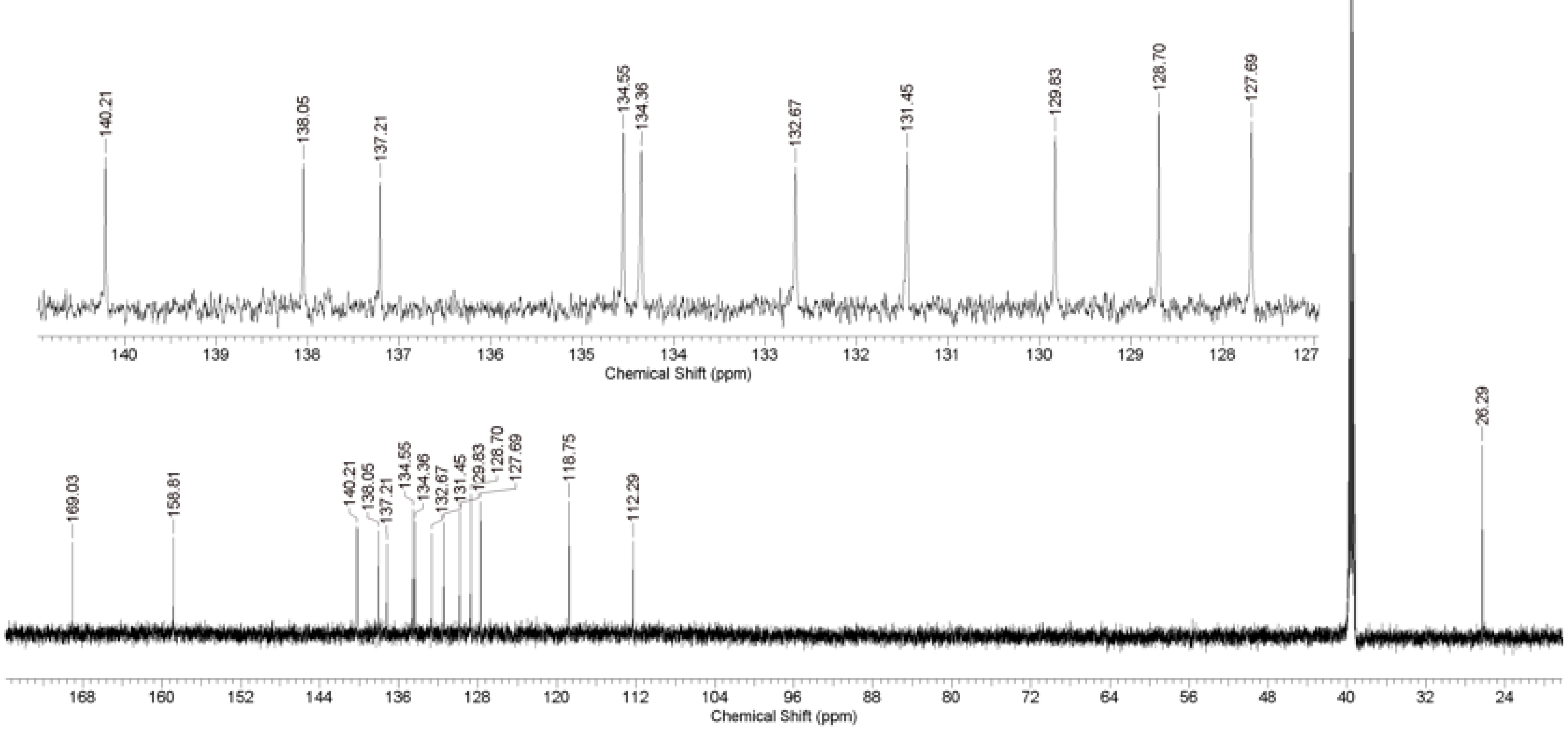
DMSO
140.21
138.05
137.21
134.55
134.36
132.67
131.45
129.83
128.70
127.69
Chemical Shift (ppm)
169.03
158.81
118.75
112.29
26.29
Chemical Shift (ppm)

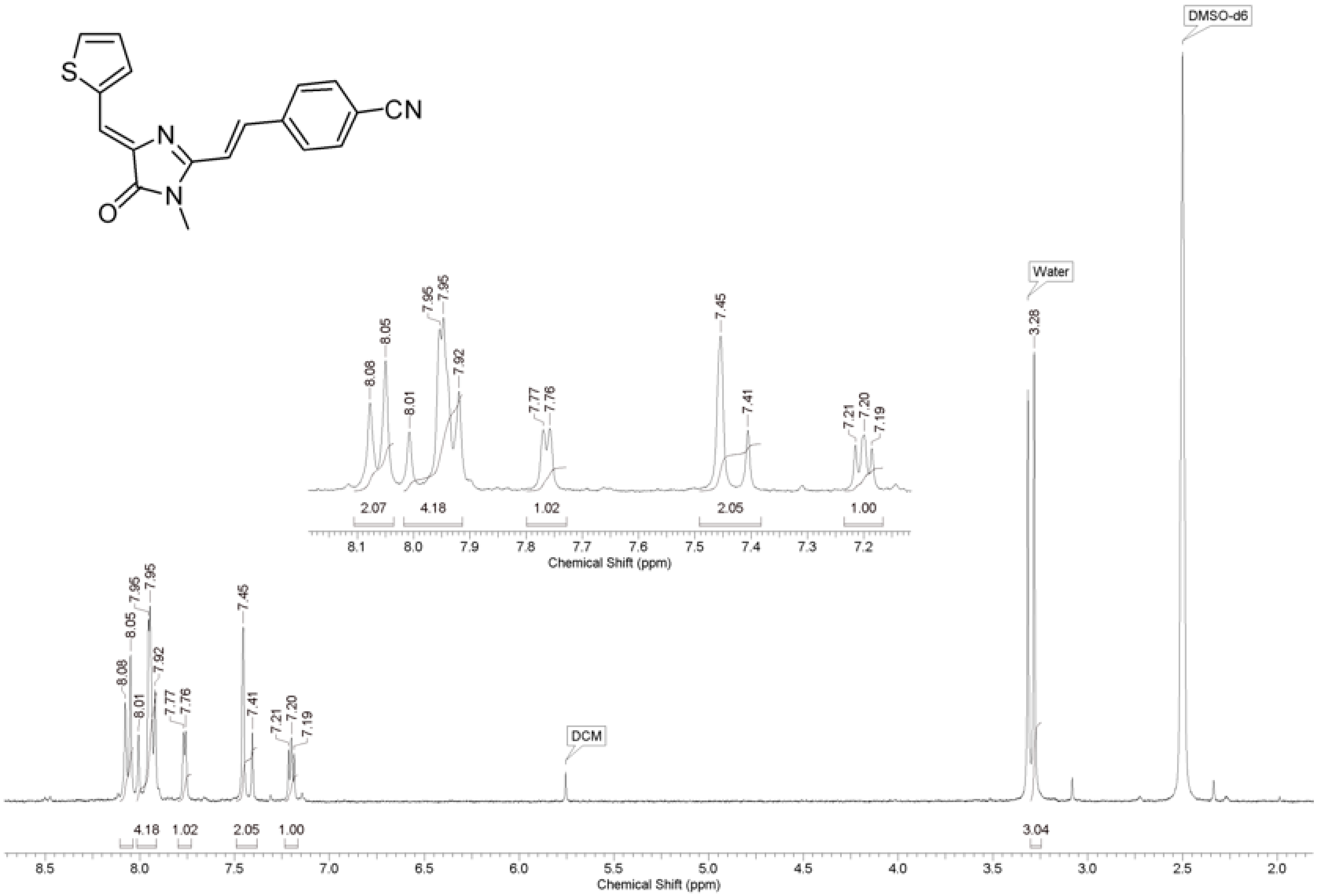
S
N
CN
N
O
Water
DMSO-d6
DCM
3.28
Chemical Shift (ppm)
2.07
4.18
1.02
2.05
1.00
3.04

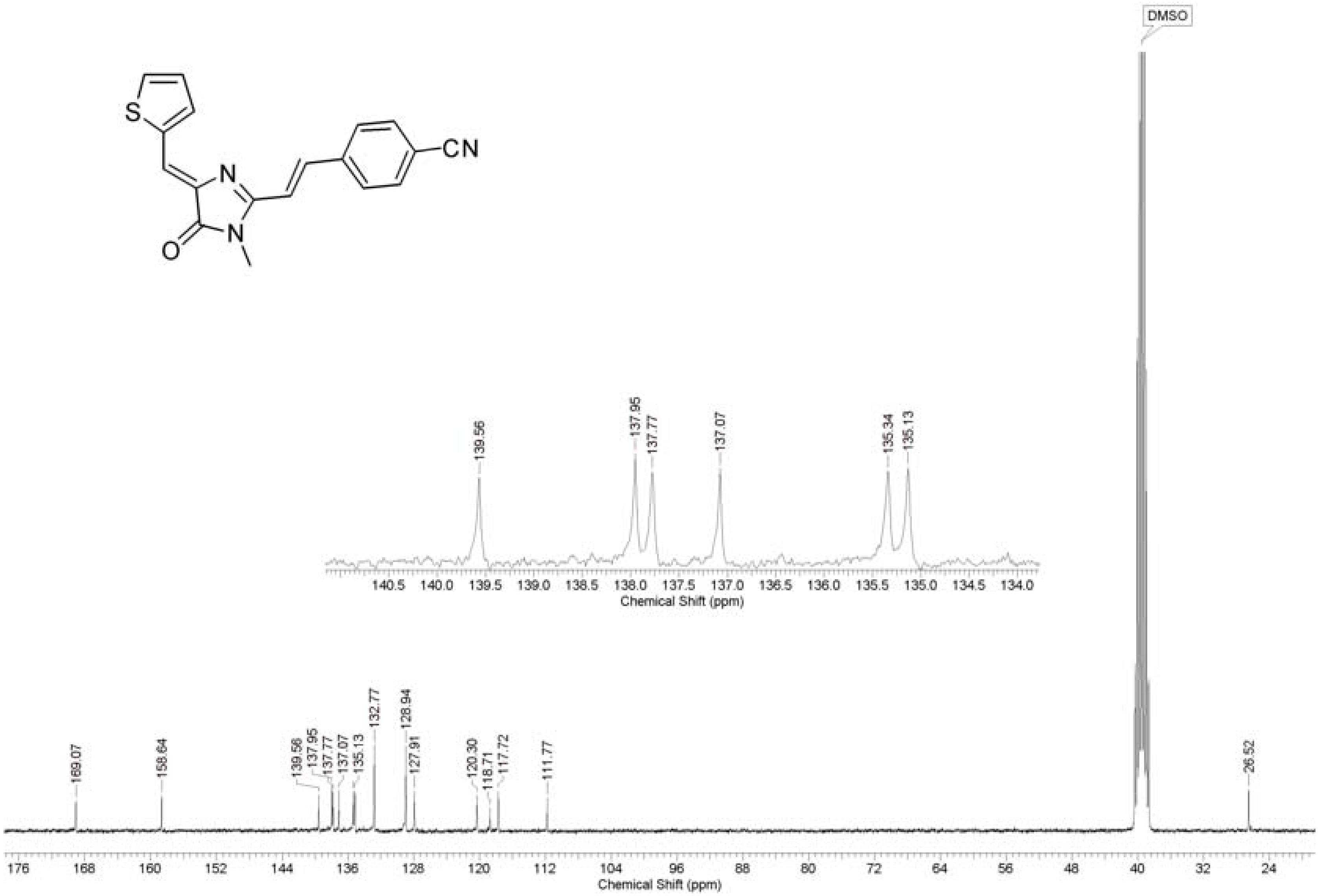
S
N
CN
O
N
DMSO
139.56
137.95
137.77
137.07
135.34
135.13
140.5 140.0 139.5 139.0 138.5 138.0 137.5 137.0 136.5 136.0 135.5 135.0 134.5 134.0
Chemical Shift (ppm)
169.07
158.64
139.56
137.95
137.77
137.07
135.13
132.77
128.94
127.91
120.30
118.71
117.72
111.77
26.52
176 168 160 152 144 136 128 120 112 104 96 88 80 72 64 56 48 40 32 24
Chemical Shift (ppm)

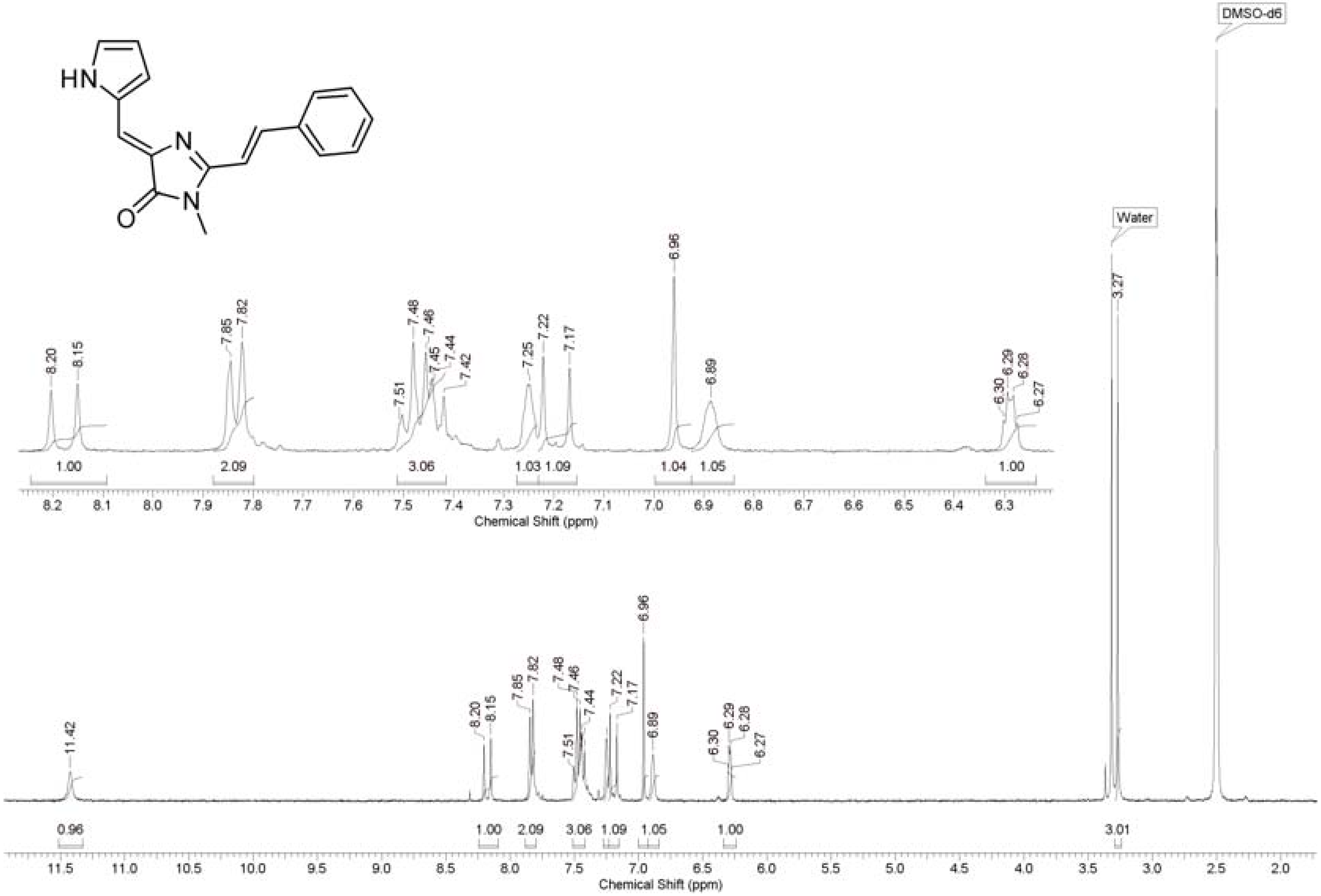
HN
N
N
O
DMSO-d6
Water
3.27
8.20
8.15
7.85
7.82
7.51
7.48
7.46
7.45
7.44
7.42
7.25
7.22
7.17
6.96
6.89
6.30
6.29
6.28
6.27
1.00
2.09
3.06
1.03
1.09
1.04
1.05
1.00
Chemical Shift (ppm)
11.42
0.96
3.01
Chemical Shift (ppm)

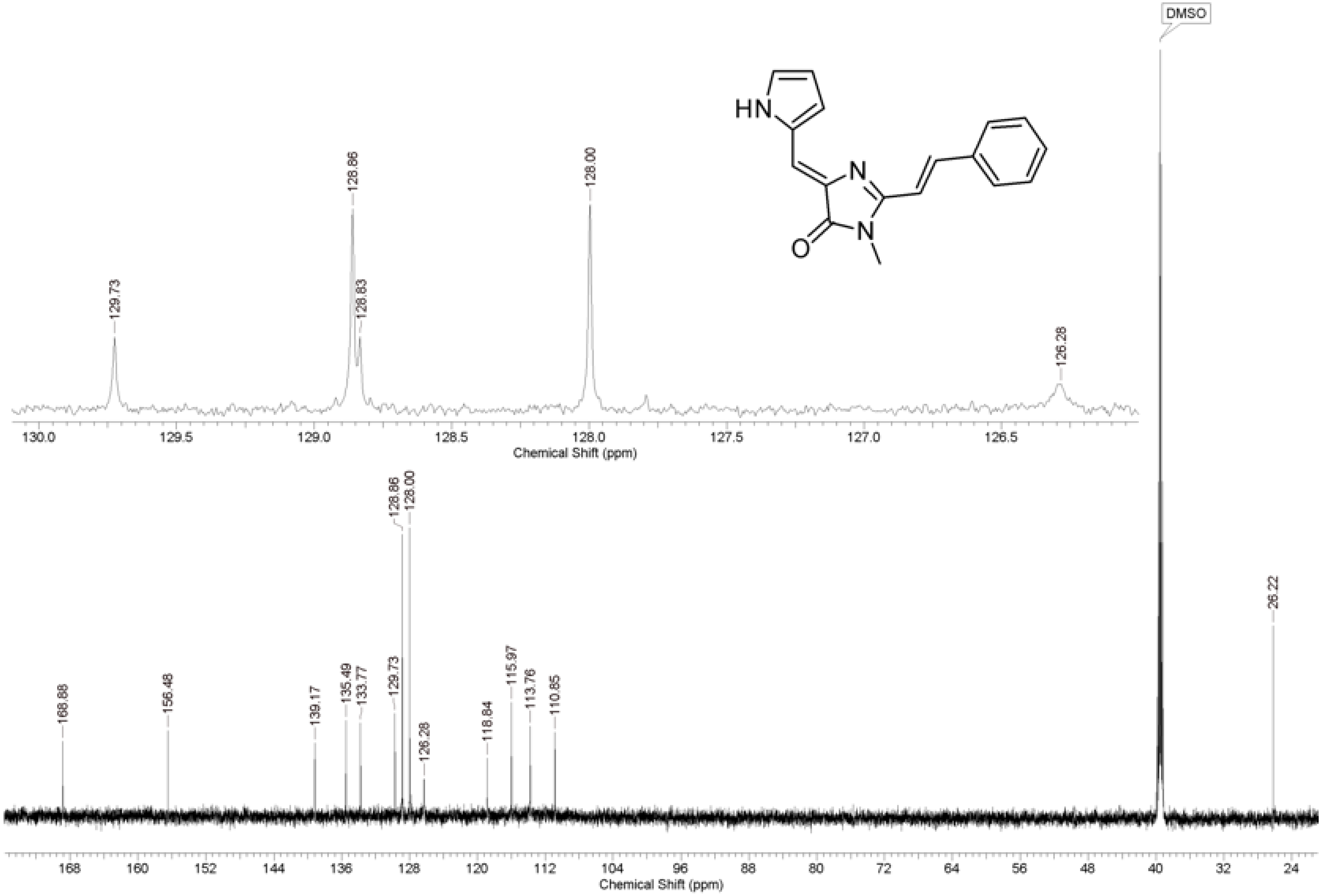
DMSO
HN
N
N
O
129.73
128.86
128.83
128.00
126.28
130.0
129.5
129.0
128.5
128.0
127.5
127.0
126.5
Chemical Shift (ppm)
168.88
156.48
139.17
135.49
133.77
129.73
128.86
128.00
126.28
118.84
115.97
113.76
110.85
26.22
168
160
152
144
136
128
120
112
104
96
88
80
72
64
56
48
40
32
24
Chemical Shift (ppm)

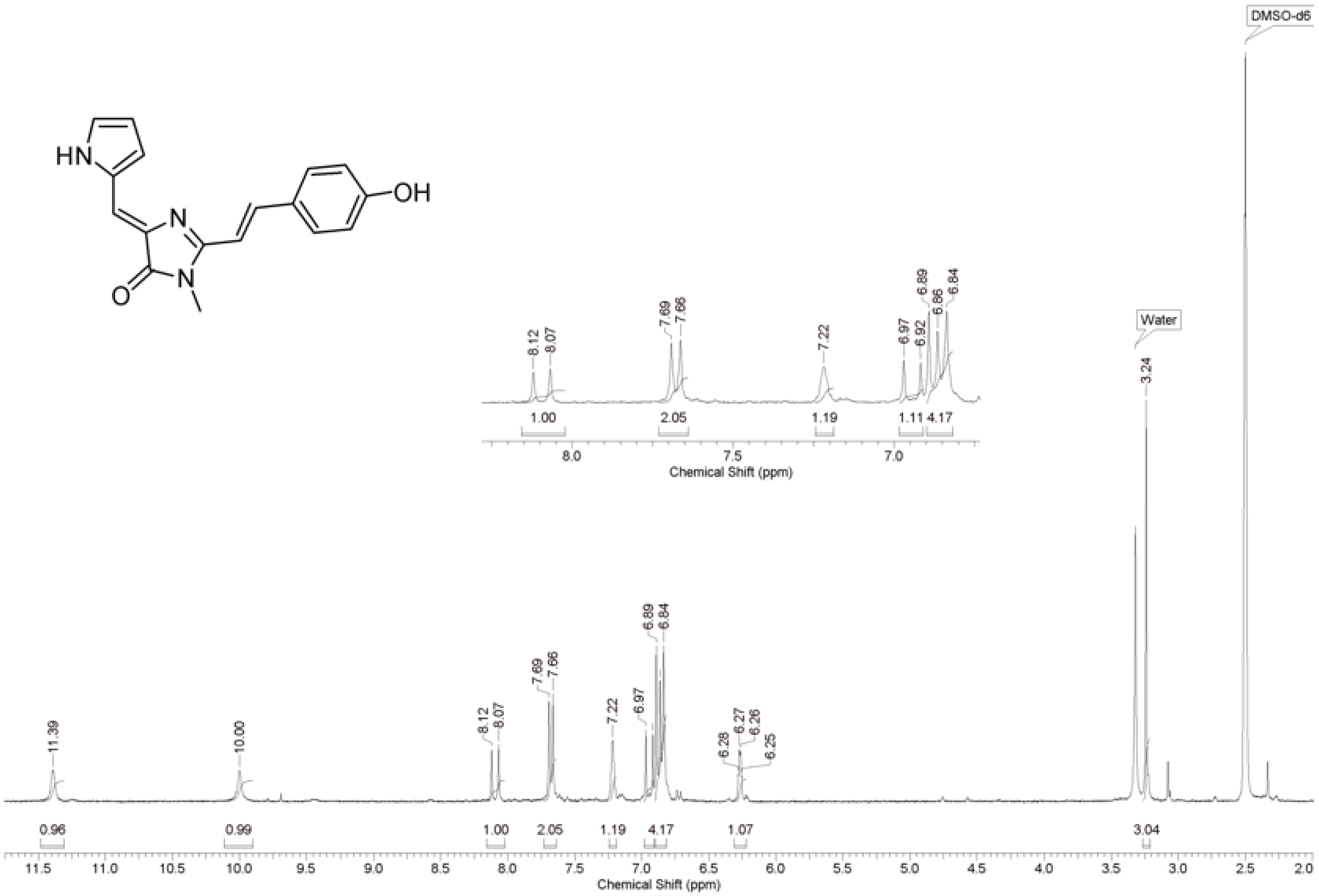
HN
N
OH
O
N
DMSO-d6
Water
3.24
8.12
8.07
7.69
7.66
7.22
6.97
6.92
6.89
6.86
6.84
1.00
2.05
1.19
1.11
4.17
8.0
7.5
7.0
Chemical Shift (ppm)
11.39
10.00
6.28
6.27
6.26
6.25
0.96
0.99
1.07
3.04
11.5
11.0
10.5
10.0
9.5
9.0
8.5
6.5
6.0
5.5
5.0
4.5
4.0
3.5
3.0
2.5
2.0
Chemical Shift (ppm)

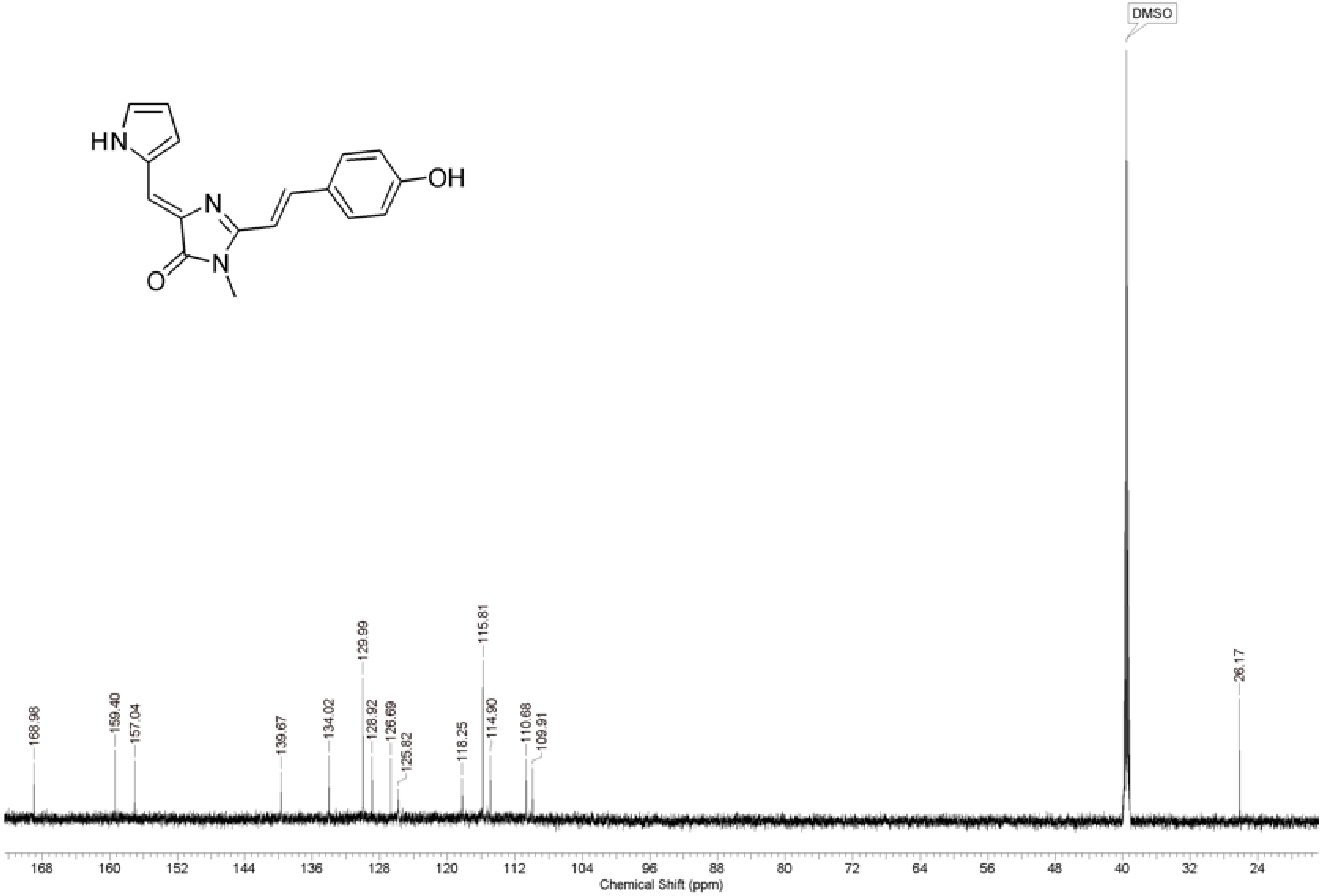
HN
N
N
O
OH
DMSO
168.98
159.40
157.04
139.67
134.02
129.99
128.92
126.69
125.82
118.25
115.81
114.90
110.68
109.91
26.17
168 160 152 144 136 128 120 112 104 96 88 80 72 64 56 48 40 32 24
Chemical Shift (ppm)

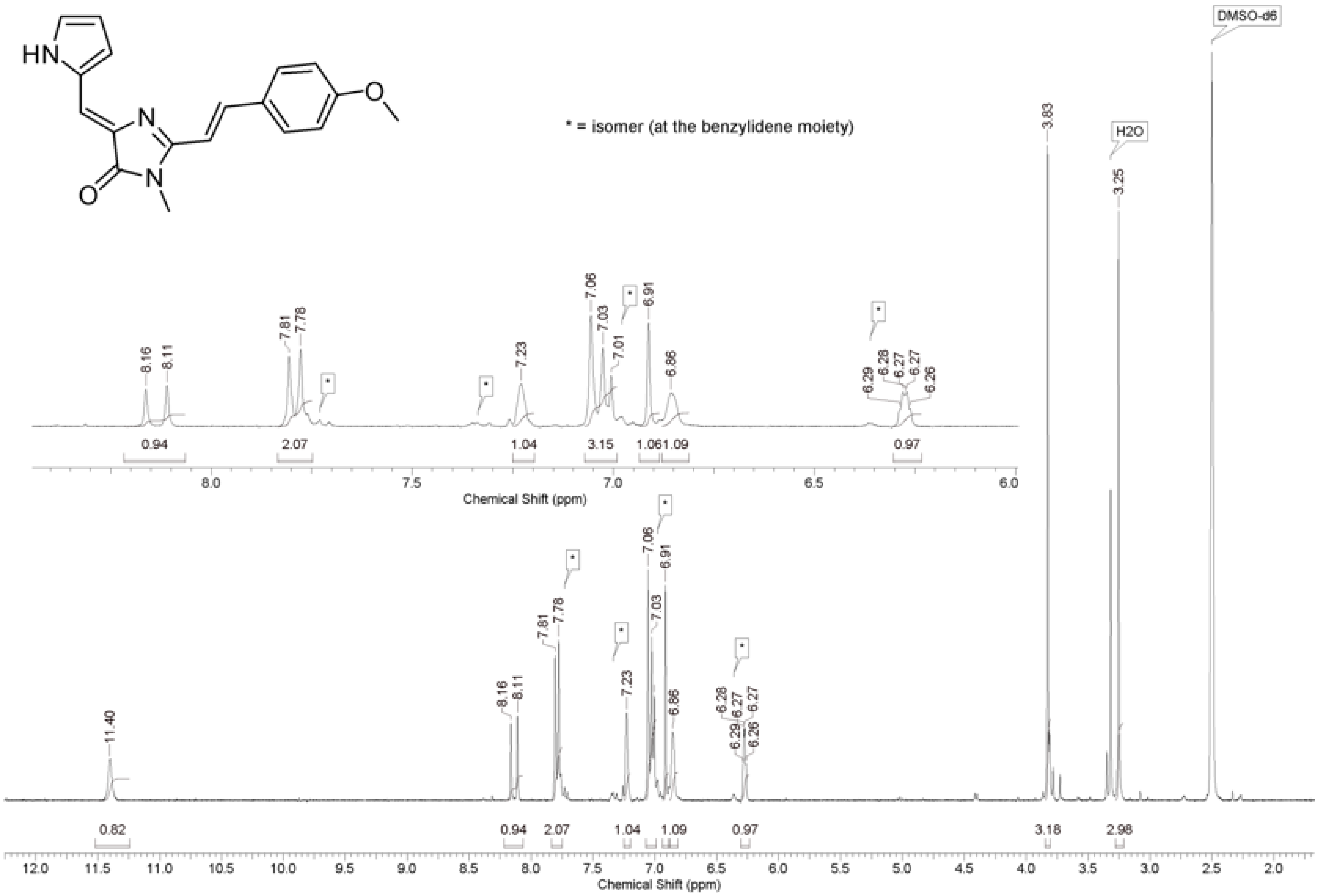
* = isomer (at the benzylidene moiety)
DMSO-d6
H2O
3.83
3.25
Chemical Shift (ppm)

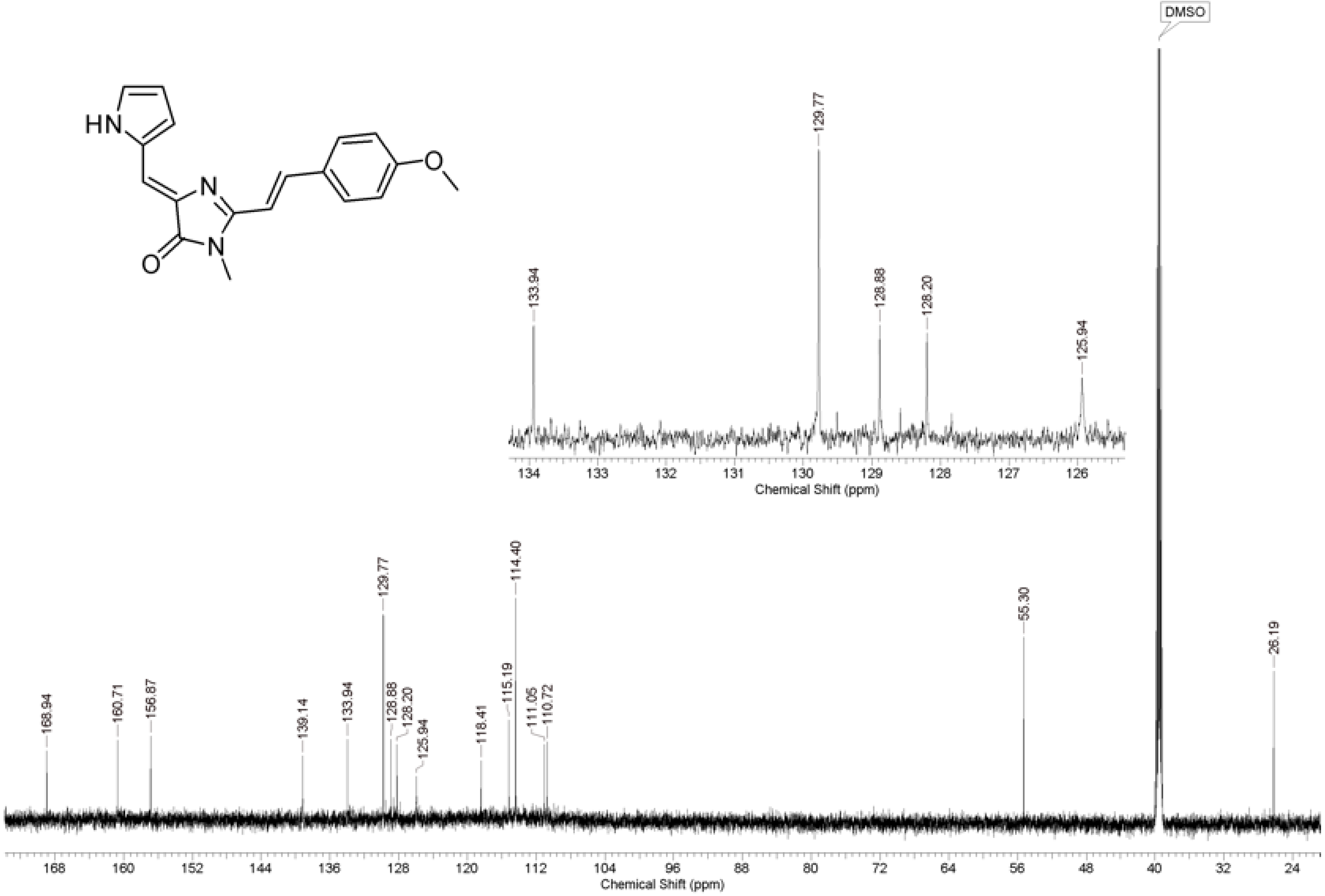

DMSO
129.77
133.94
128.88
128.20
125.94
134
133
132
131
130
129
128
127
126
Chemical Shift (ppm)
168.94
160.71
156.87
139.14
133.94
129.77
128.88
128.20
125.94
118.41
115.19
114.40
111.05
110.72
55.30
26.19
168
160
152
144
136
128
120
112
104
96
88
80
72
64
56
48
40
32
24
Chemical Shift (ppm)

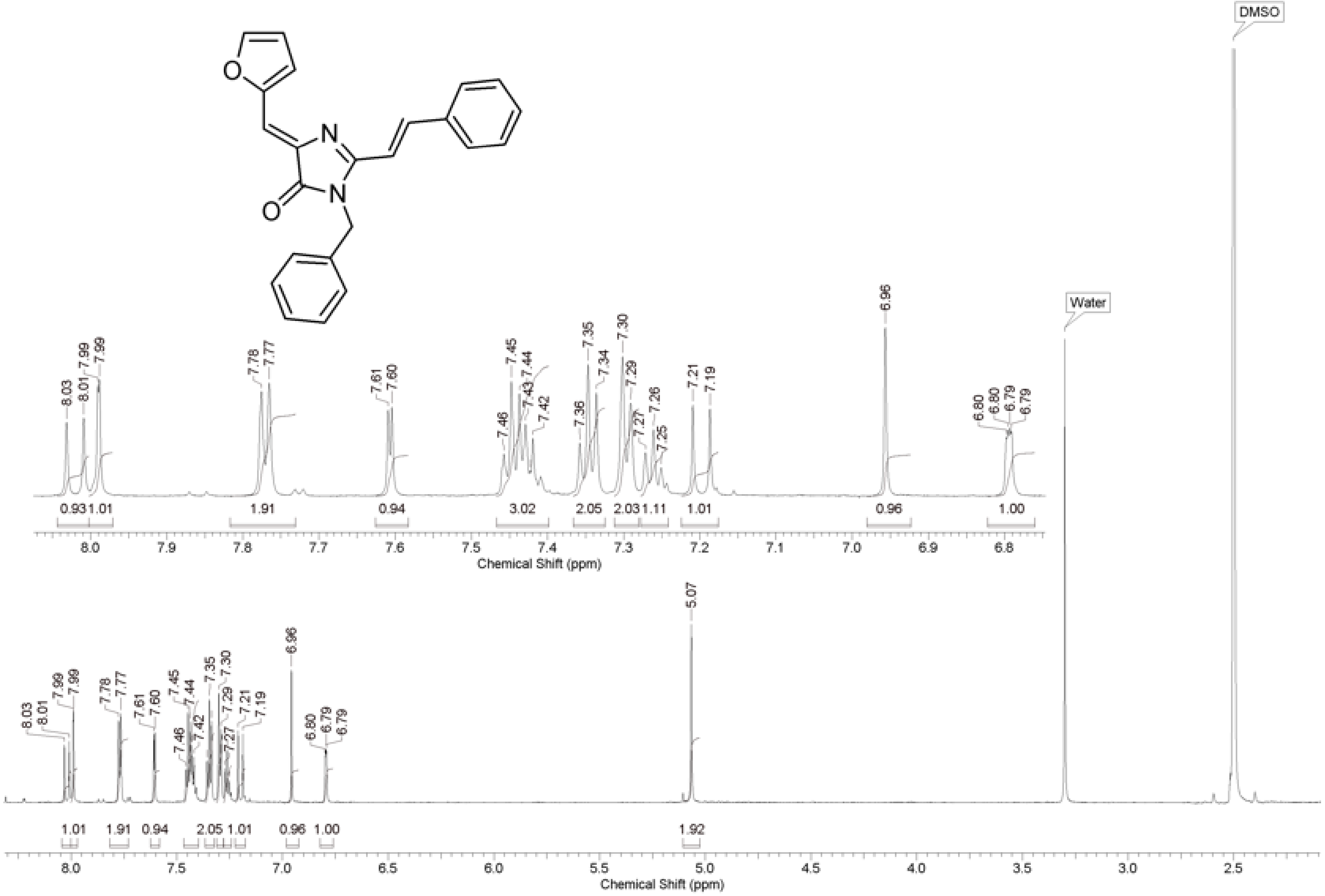

DMSO
Water
Chemical Shift (ppm)
Chemical Shift (ppm)

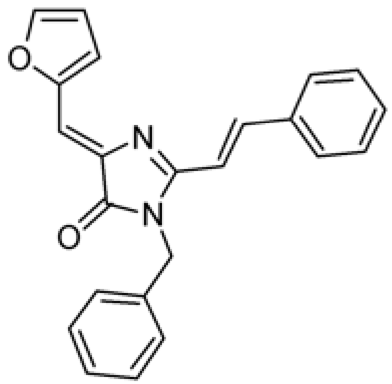

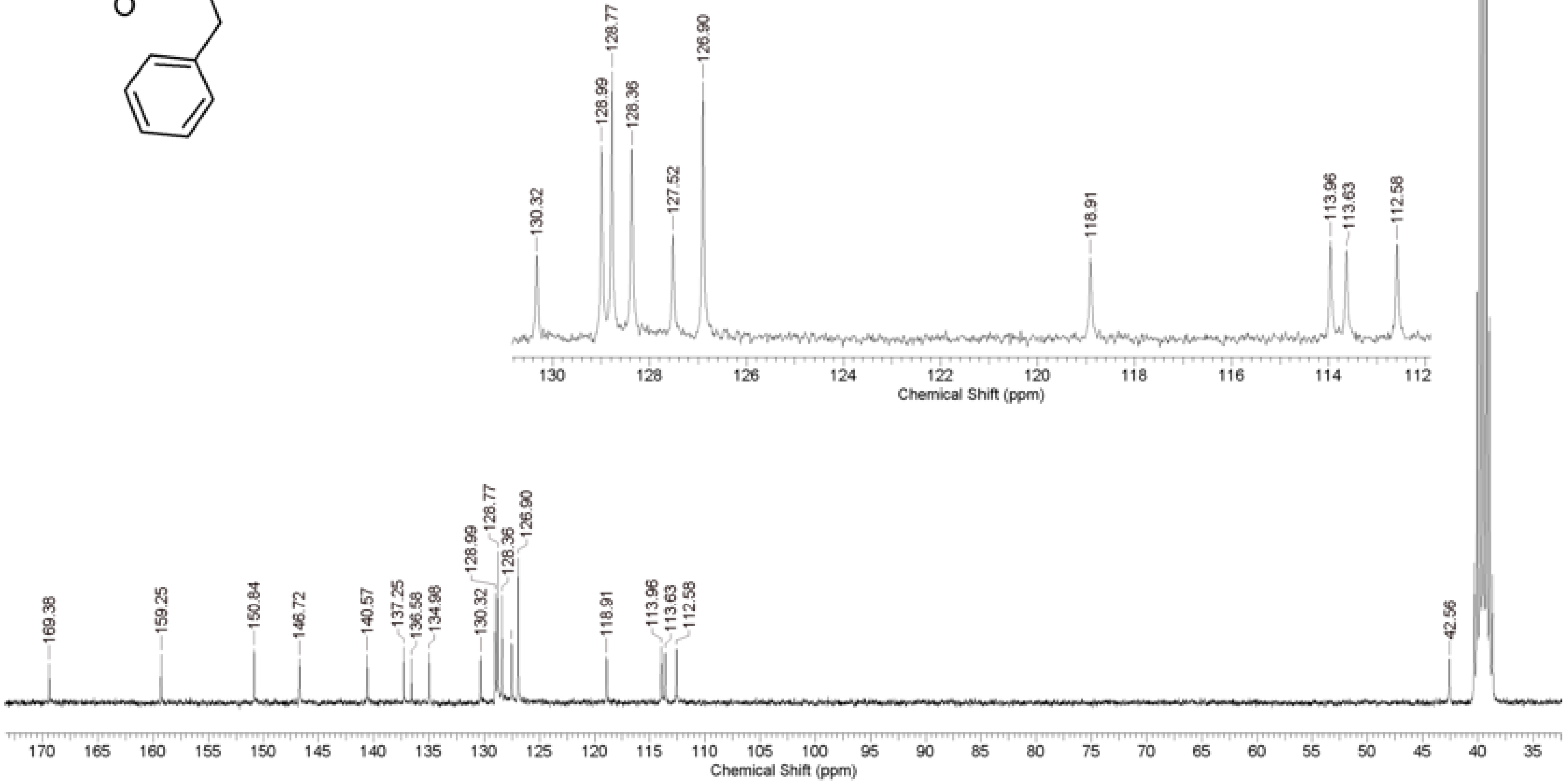
DMSO
130.32
128.99
128.77
128.36
127.52
126.90
118.91
113.96
113.63
112.58
130
128
126
124
122
120
118
116
114
112
Chemical Shift (ppm)
169.38
159.25
150.84
146.72
140.57
137.25
136.58
134.98
130.32
128.99
128.77
128.36
126.90
118.91
113.96
113.63
112.58
42.56
170
165
160
155
150
145
140
135
130
125
120
115
110
105
100
95
90
85
80
75
70
65
60
55
50
45
40
35
Chemical Shift (ppm)

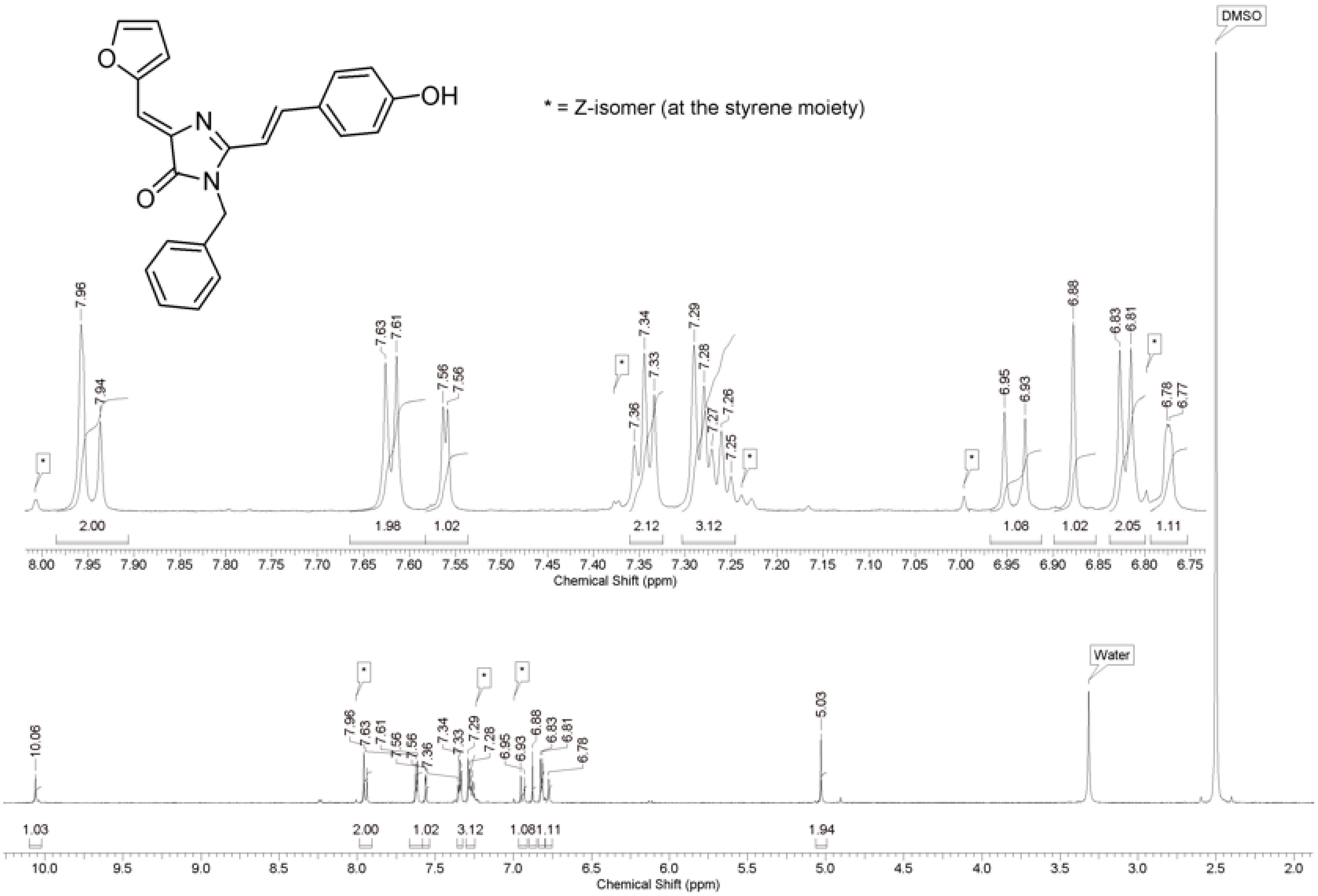

O
N
N
O
OH
* = Z-isomer (at the styrene moiety)
DMSO
Water
7.96
7.94
7.63
7.61
7.56
7.56
7.36
7.34
7.33
7.29
7.28
7.27
7.26
7.25
6.95
6.93
6.88
6.83
6.81
6.78
6.77
10.06
5.03
2.00
1.98
1.02
2.12
3.12
1.08
1.02
2.05
1.11
1.03
1.94
Chemical Shift (ppm)
Chemical Shift (ppm)

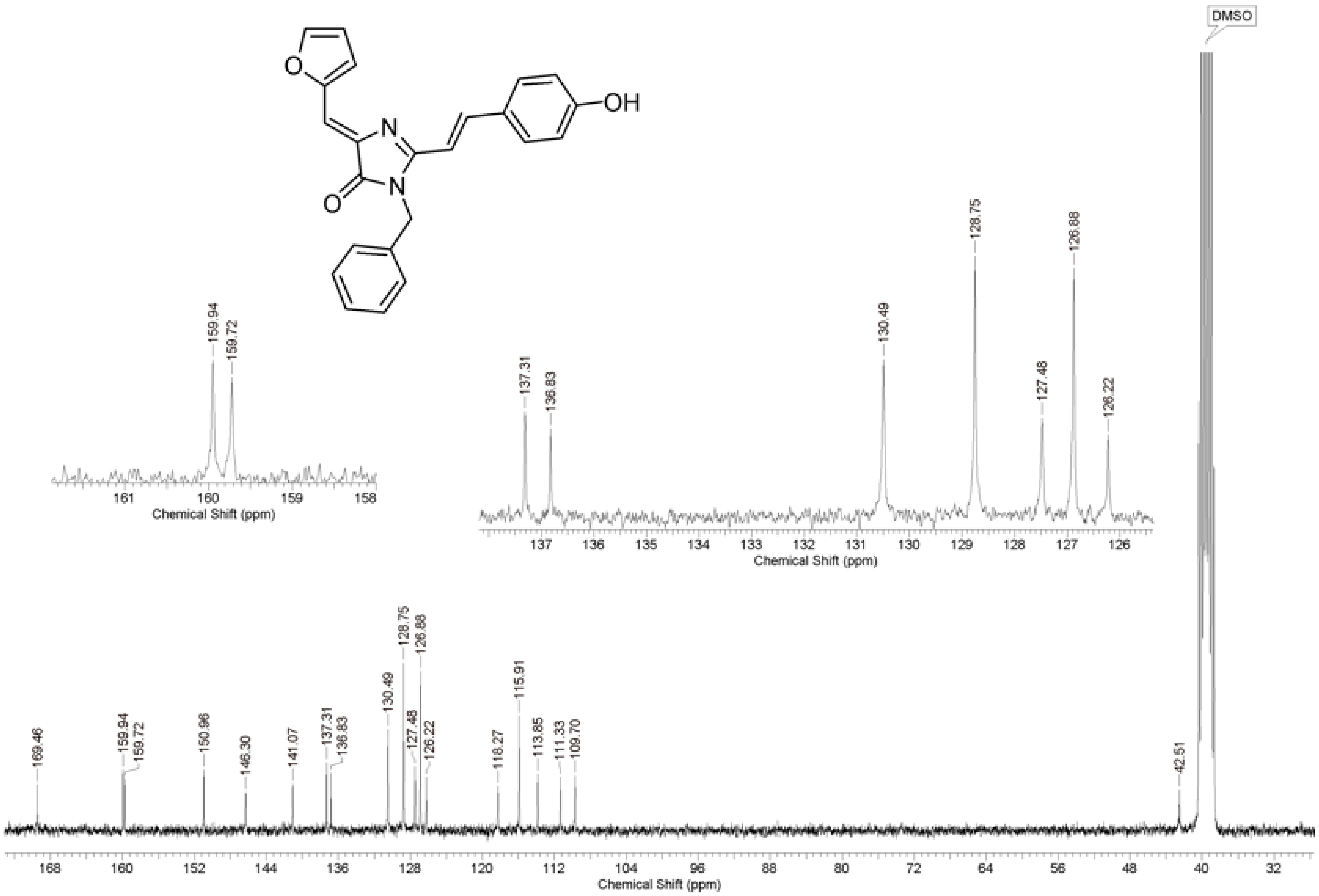
O
N
N
O
OH
DMSO
159.94
159.72
161 160 159 158
Chemical Shift (ppm)
137.31
136.83
130.49
128.75
127.48
126.88
126.22
137 136 135 134 133 132 131 130 129 128 127 126
Chemical Shift (ppm)
169.46
159.94
159.72
150.96
146.30
141.07
137.31
136.83
130.49
128.75
127.48
126.88
126.22
118.27
115.91
113.85
111.33
109.70
42.51
168 160 152 144 136 128 120 112 104 96 88 80 72 64 56 48 40 32
Chemical Shift (ppm)

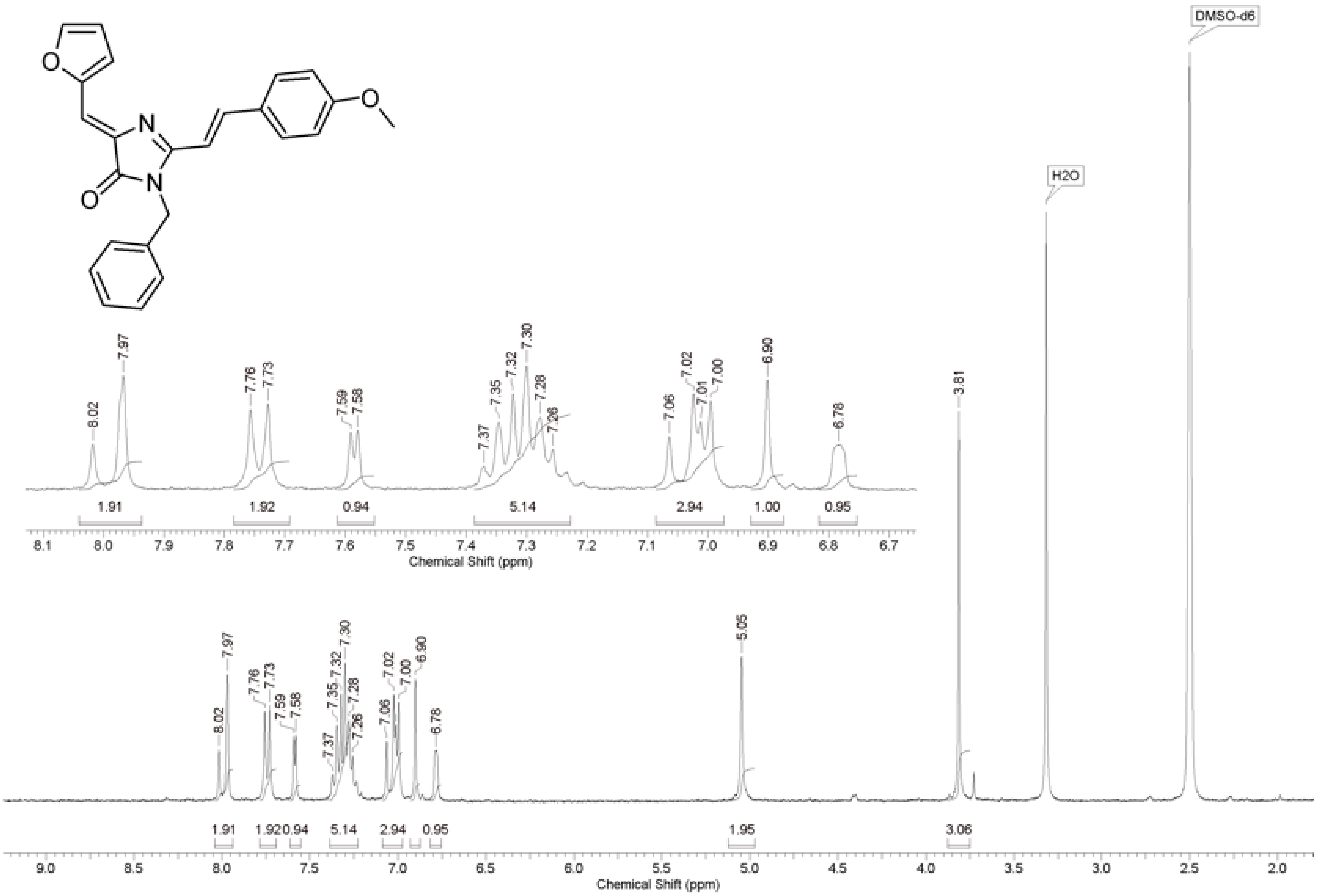

DMSO-d6
H2O
8.02
7.97
7.76
7.73
7.59
7.58
7.37
7.35
7.32
7.30
7.28
7.26
7.06
7.02
7.01
7.00
6.90
6.78
5.05
3.81
1.91
1.92
0.94
5.14
2.94
1.00
0.95
1.95
3.06
Chemical Shift (ppm)

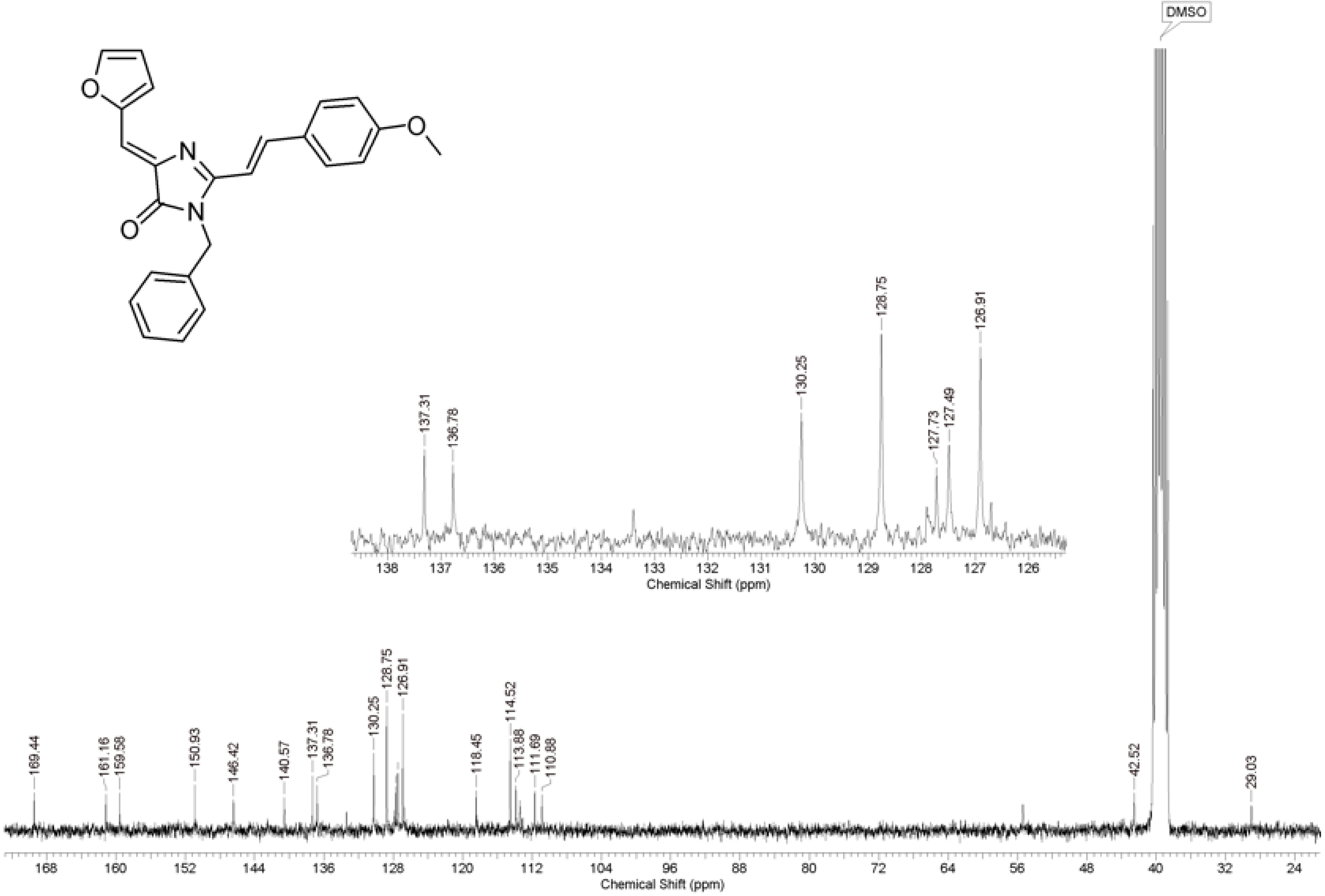
O
N
N
O
O
DMSO
137.31
136.78
130.25
128.75
127.73
127.49
126.91
138 137 136 135 134 133 132 131 130 129 128 127 126
Chemical Shift (ppm)
169.44
161.16
159.58
150.93
146.42
140.57
137.31
136.78
130.25
128.75
126.91
118.45
114.52
113.88
111.69
110.88
42.52
29.03
168 160 152 144 136 128 120 112 104 96 88 80 72 64 56 48 40 32 24
Chemical Shift (ppm)

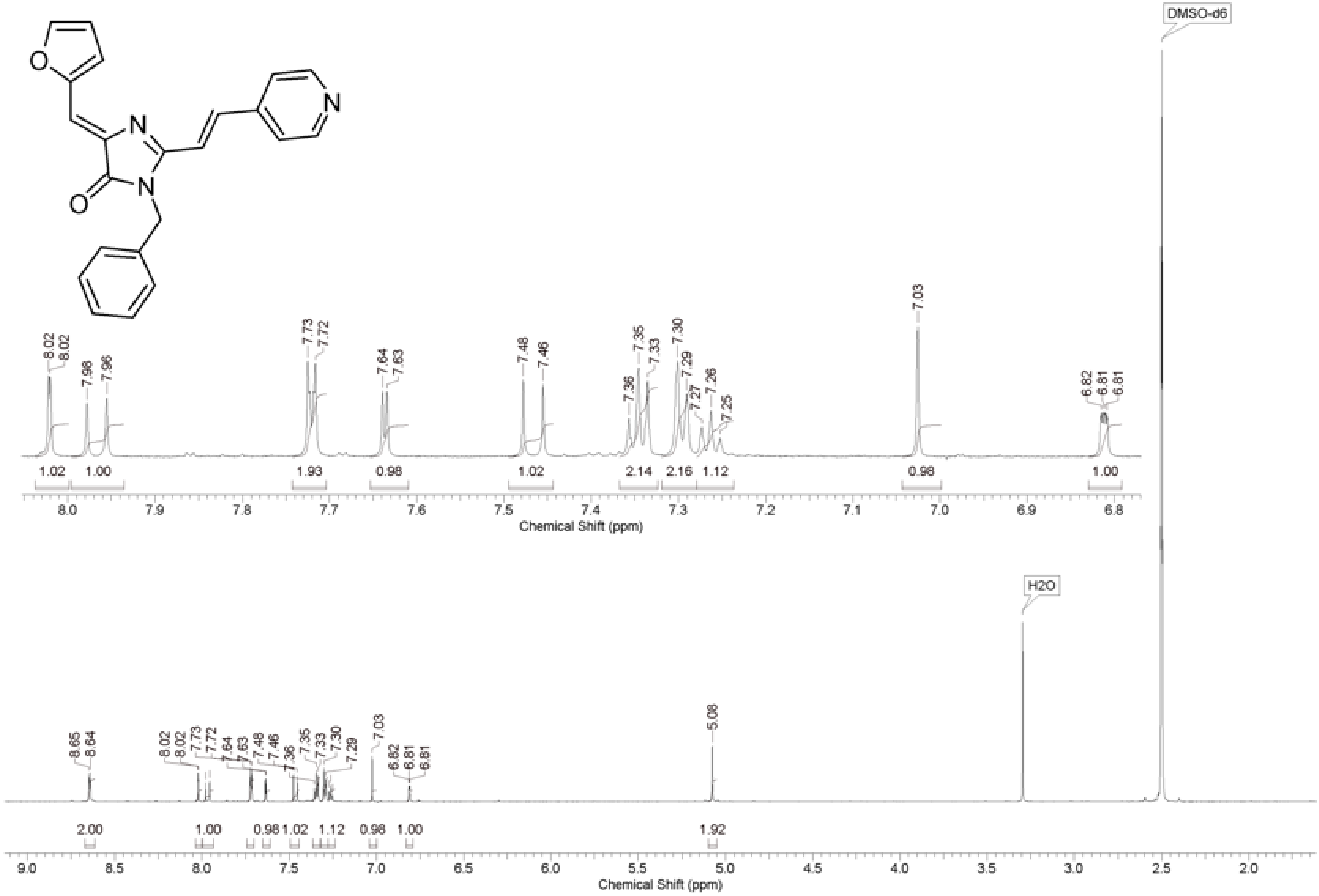
DMSO-d6
H2O
Chemical Shift (ppm)
Chemical Shift (ppm)

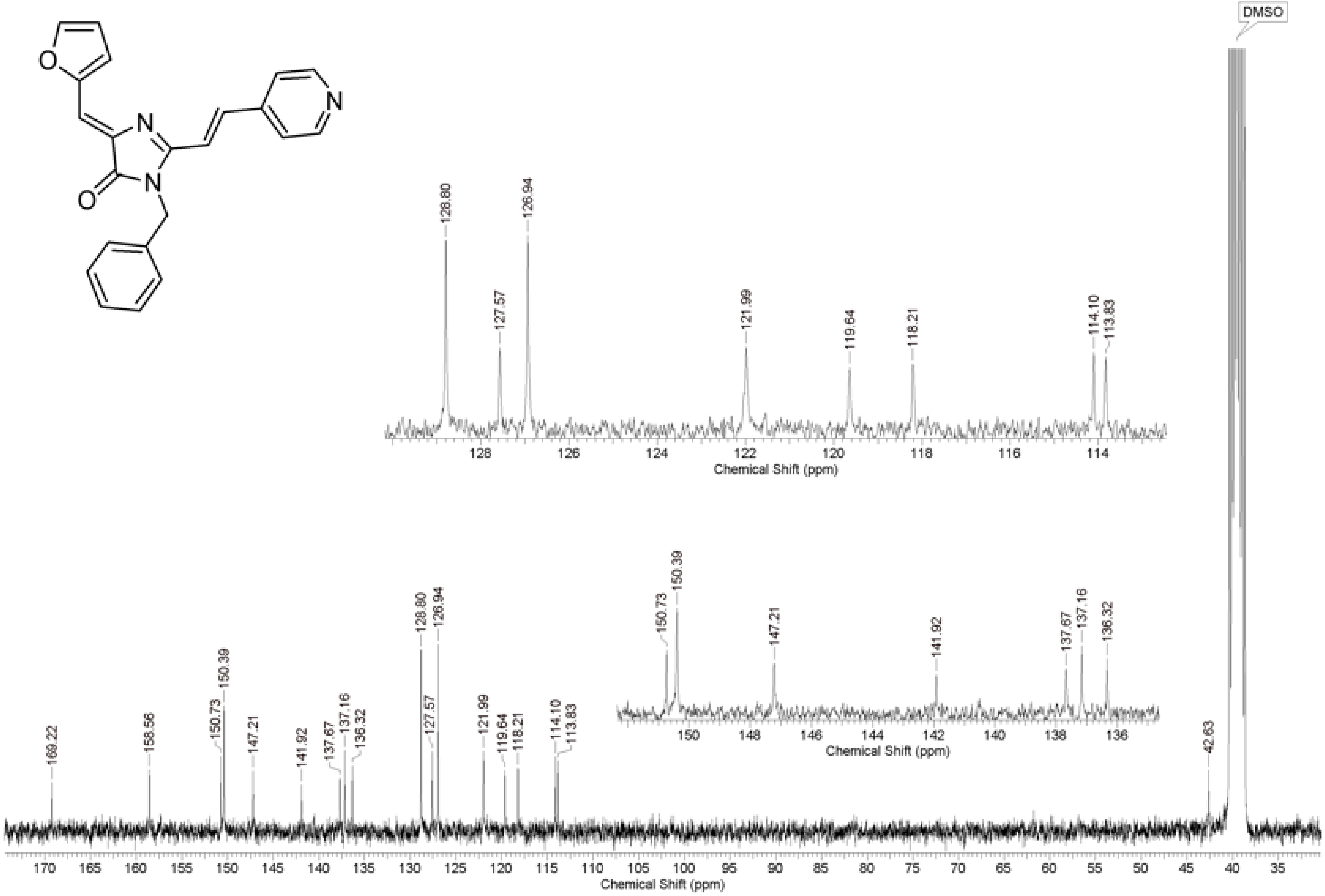
O
N
N
N
O
DMSO
128.80
127.57
126.94
121.99
119.64
118.21
114.10
113.83
Chemical Shift (ppm)
150.73
150.39
147.21
141.92
137.67
137.16
136.32
Chemical Shift (ppm)
169.22
158.56
42.63
Chemical Shift (ppm)

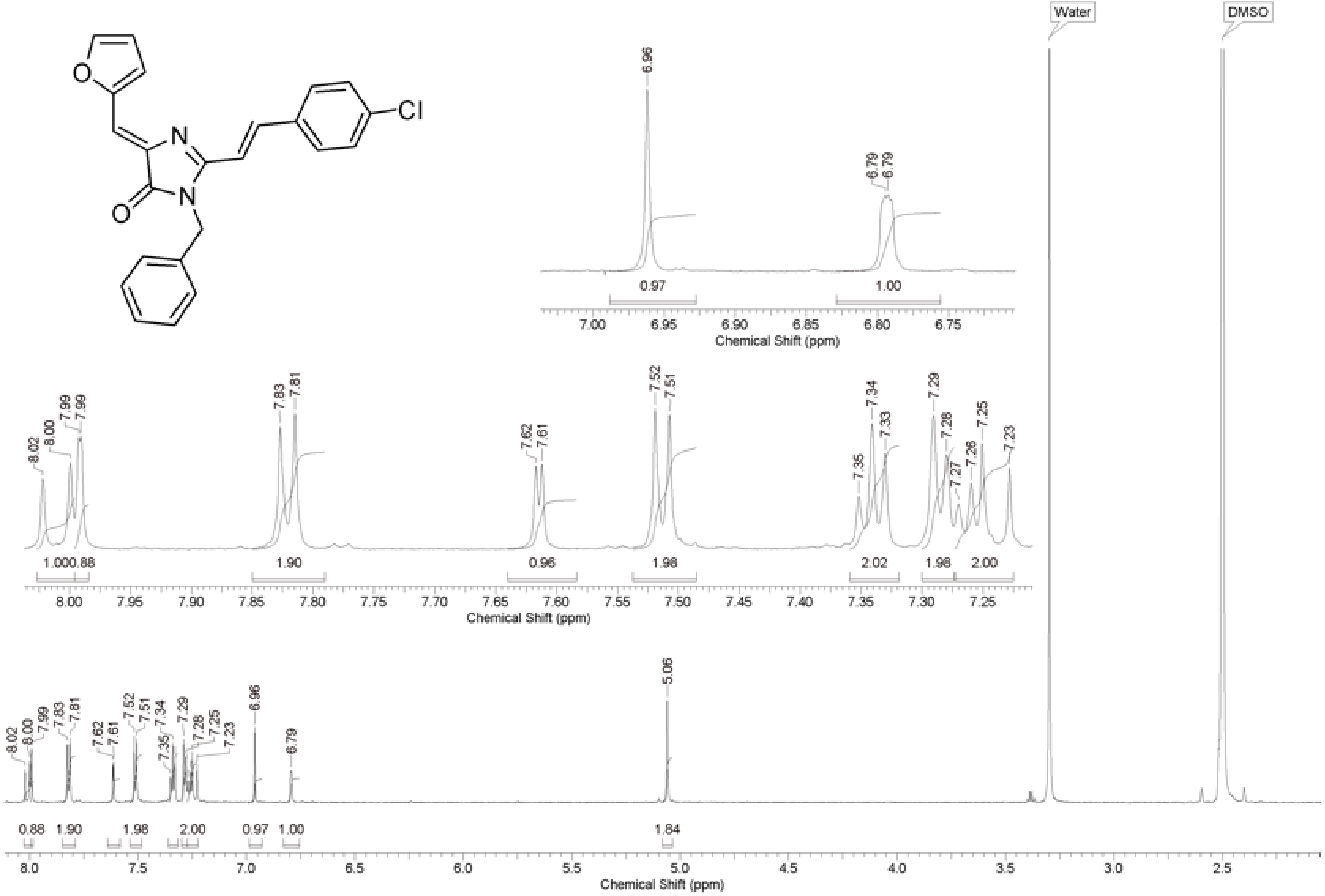
Water
DMSO
Cl
N
O
Chemical Shift (ppm)
Chemical Shift (ppm)
Chemical Shift (ppm)
0.97
1.00
1.00 0.88
1.90
0.96
1.98
2.02
1.98
2.00
0.88 1.90
1.98
2.00
0.97 1.00
1.84

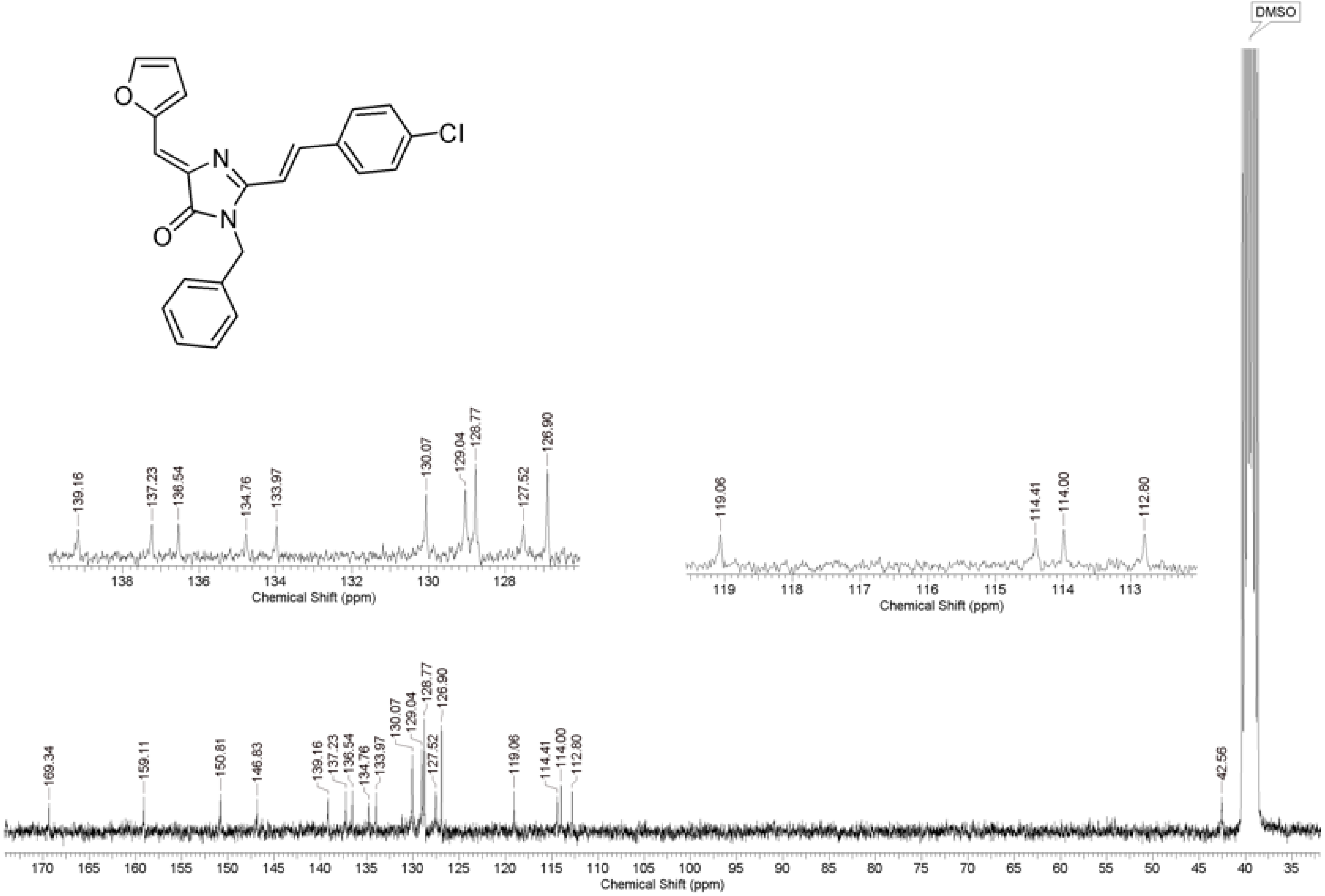
Cl
N
N
O
O
DMSO
139.16
137.23
136.54
134.76
133.97
130.07
129.04
128.77
127.52
126.90
Chemical Shift (ppm)
119.06
114.41
114.00
112.80
Chemical Shift (ppm)
169.34
159.11
150.81
146.83
139.16
137.23
136.54
134.76
133.97
130.07
129.04
128.77
127.52
126.90
119.06
114.41
114.00
112.80
42.56
Chemical Shift (ppm)

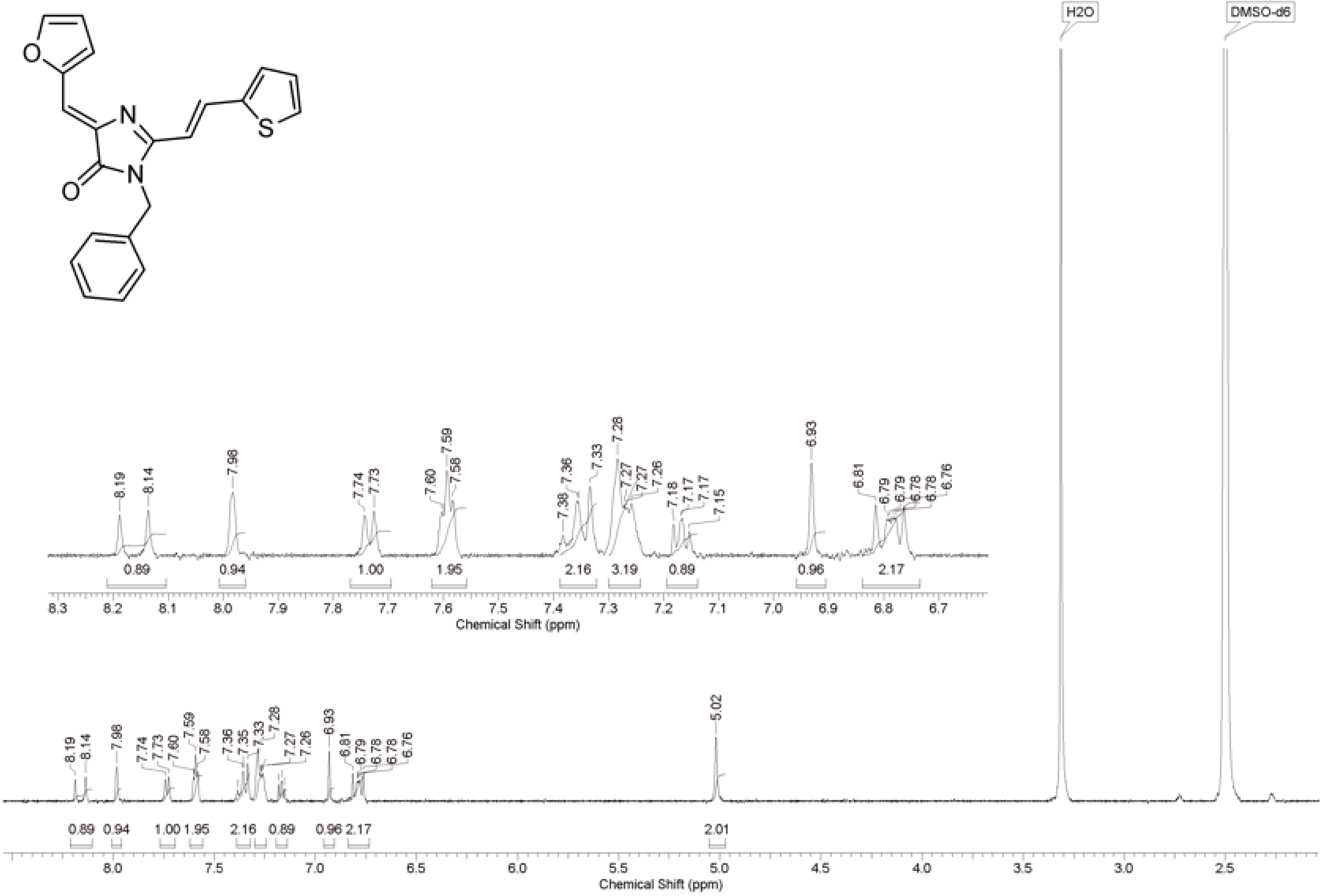
O
N
S
O
N
H2O
DMSO-d6
Chemical Shift (ppm)
Chemical Shift (ppm)
5.02
2.01

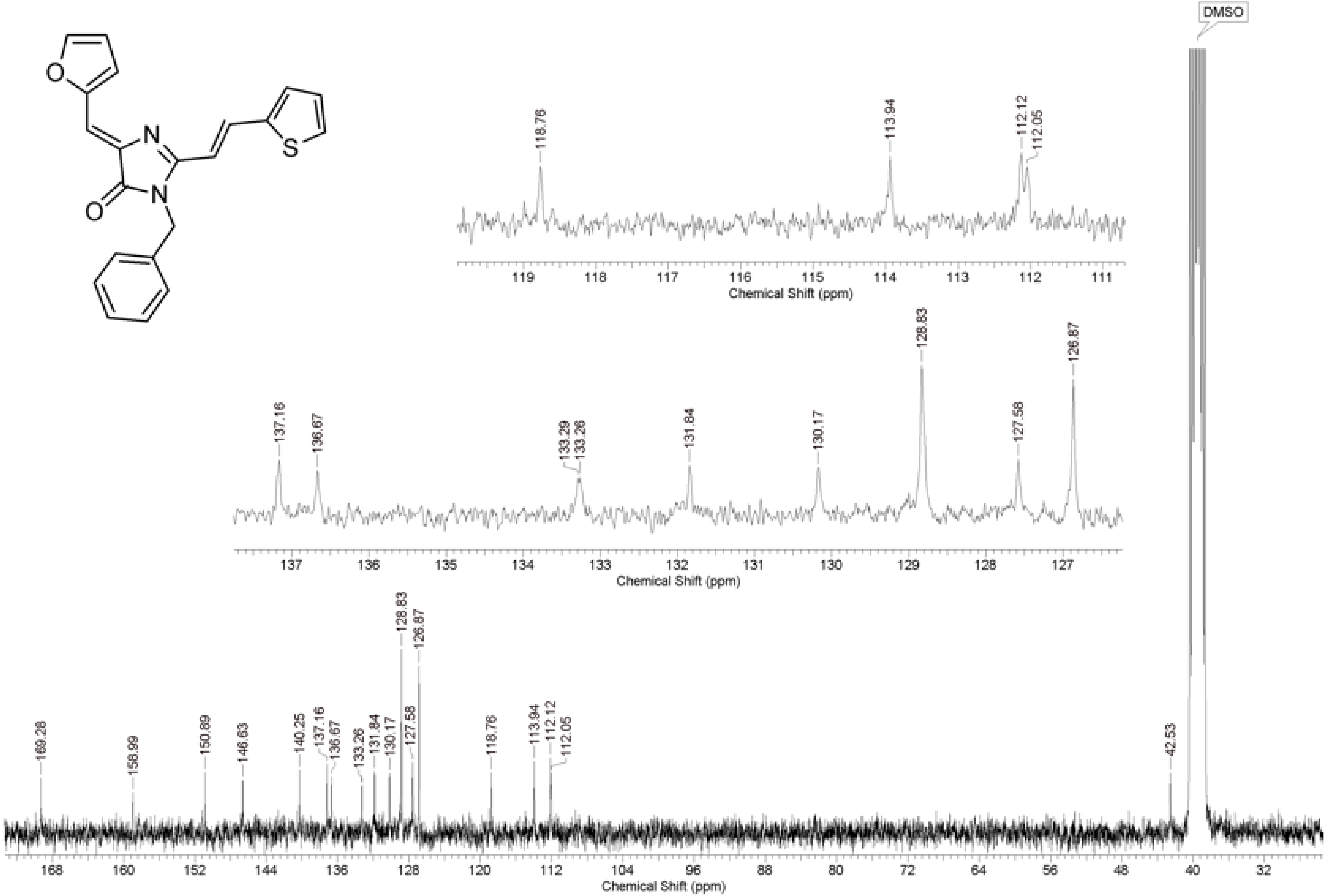
DMSO
118.76
113.94
112.12
112.05
Chemical Shift (ppm)
137.16
136.67
133.29
133.26
131.84
130.17
128.83
127.58
126.87
Chemical Shift (ppm)
169.28
158.99
150.89
146.63
140.25
137.16
136.67
133.26
131.84
130.17
128.83
127.58
126.87
118.76
113.94
112.12
112.05
42.53
Chemical Shift (ppm)

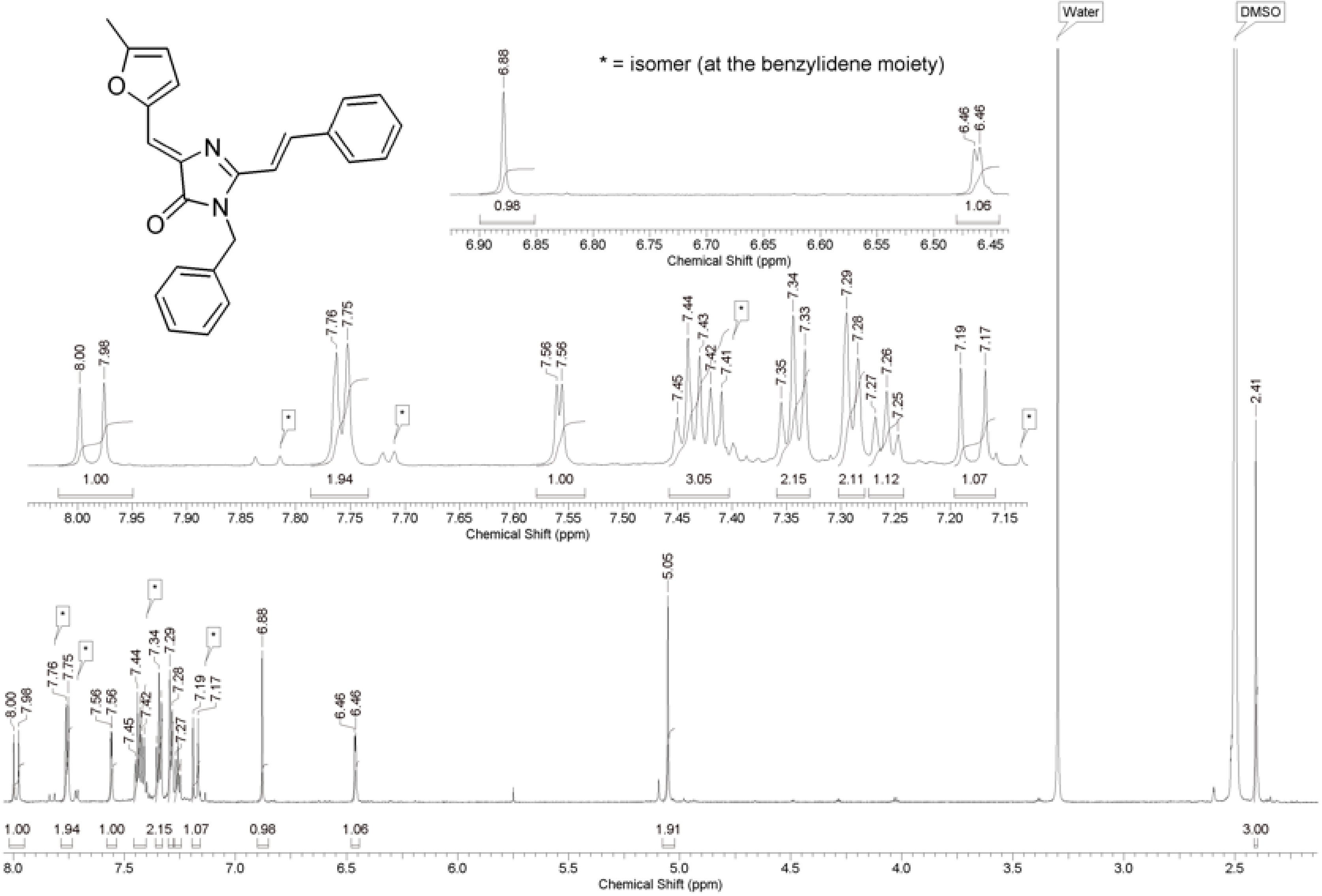
* = isomer (at the benzylidene moiety)
Water
DMSO
Chemical Shift (ppm)
Chemical Shift (ppm)
Chemical Shift (ppm)

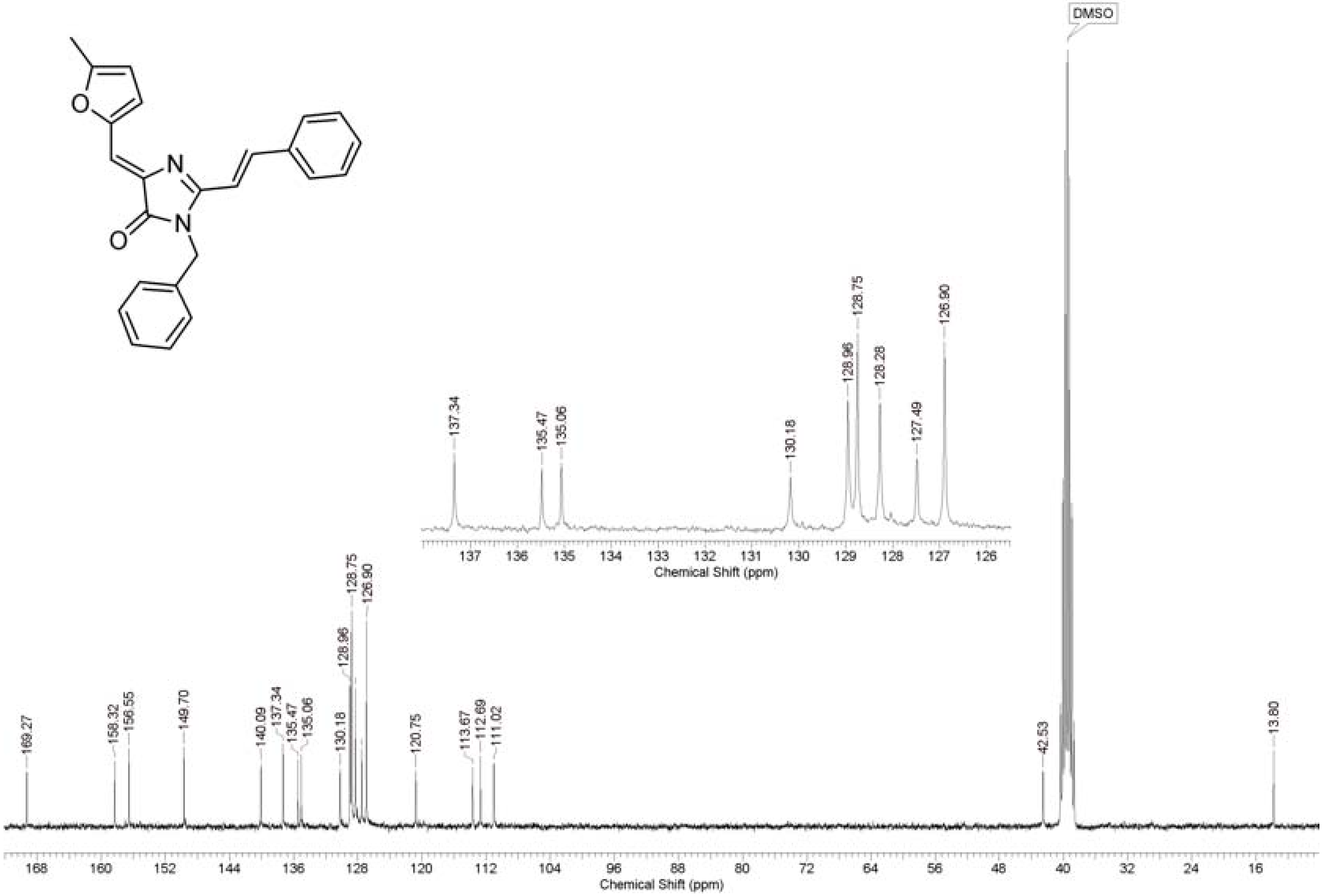
DMSO
137.34
135.47
135.06
130.18
128.96
128.75
128.28
127.49
126.90
137 136 135 134 133 132 131 130 129 128 127 126
Chemical Shift (ppm)
169.27
158.32
156.55
149.70
140.09
137.34
135.47
135.06
130.18
128.96
128.75
126.90
120.75
113.67
112.69
111.02
42.53
13.80
168 160 152 144 136 128 120 112 104 96 88 80 72 64 56 48 40 32 24 16
Chemical Shift (ppm)

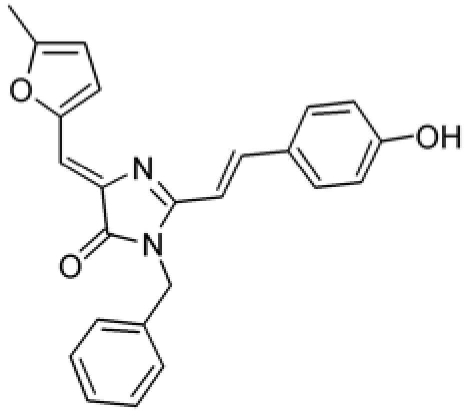
OH
N
O
N
O

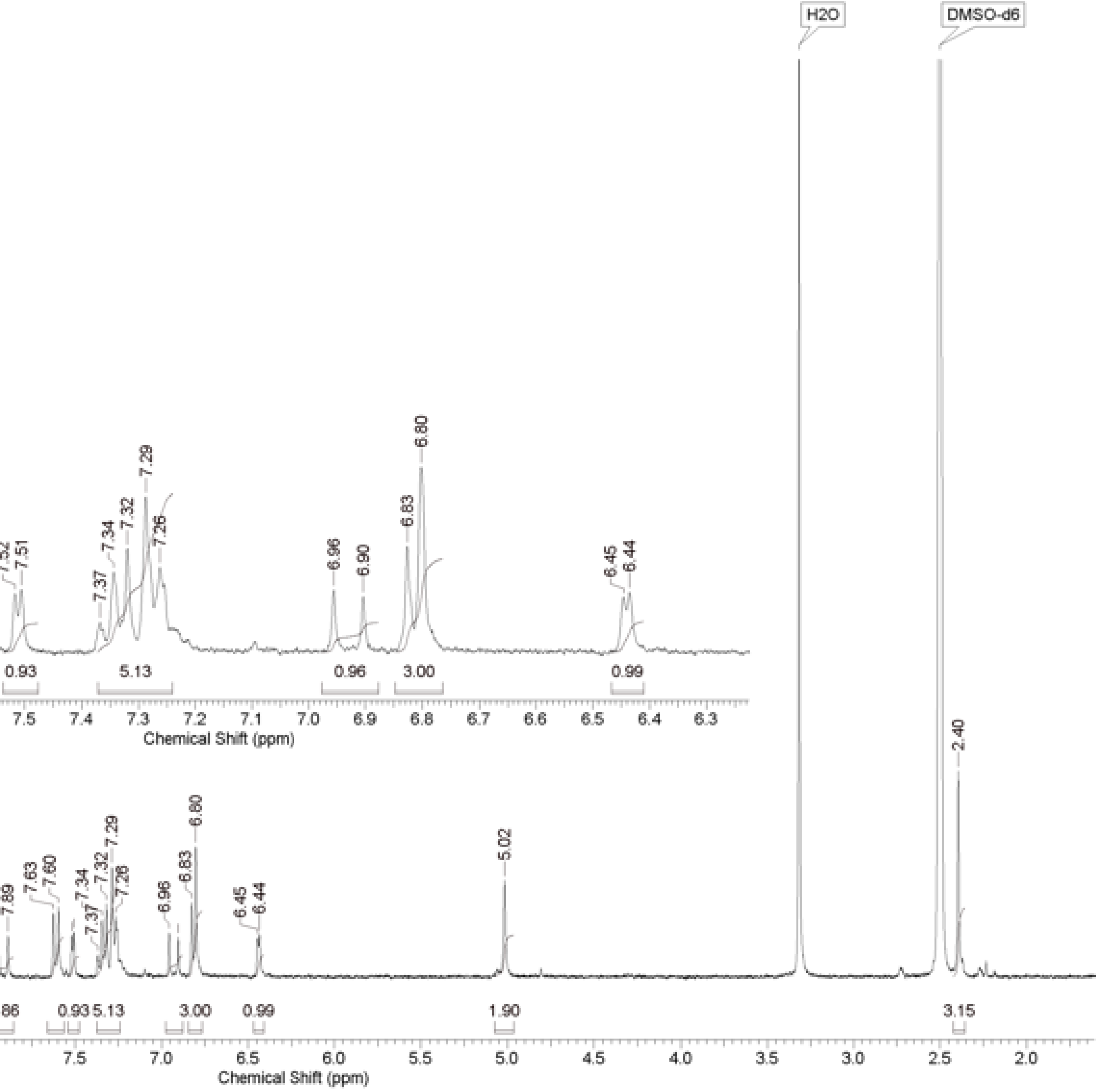
H2O
DMSO-d6
7.94
7.89
7.63
7.60
7.52
7.51
7.37
7.34
7.32
7.29
7.26
6.96
6.90
6.83
6.80
6.45
6.44
0.86
1.80
0.93
5.13
0.96
3.00
0.99
Chemical Shift (ppm)
10.05
5.02
2.40
0.88
1.90
3.15
Chemical Shift (ppm)

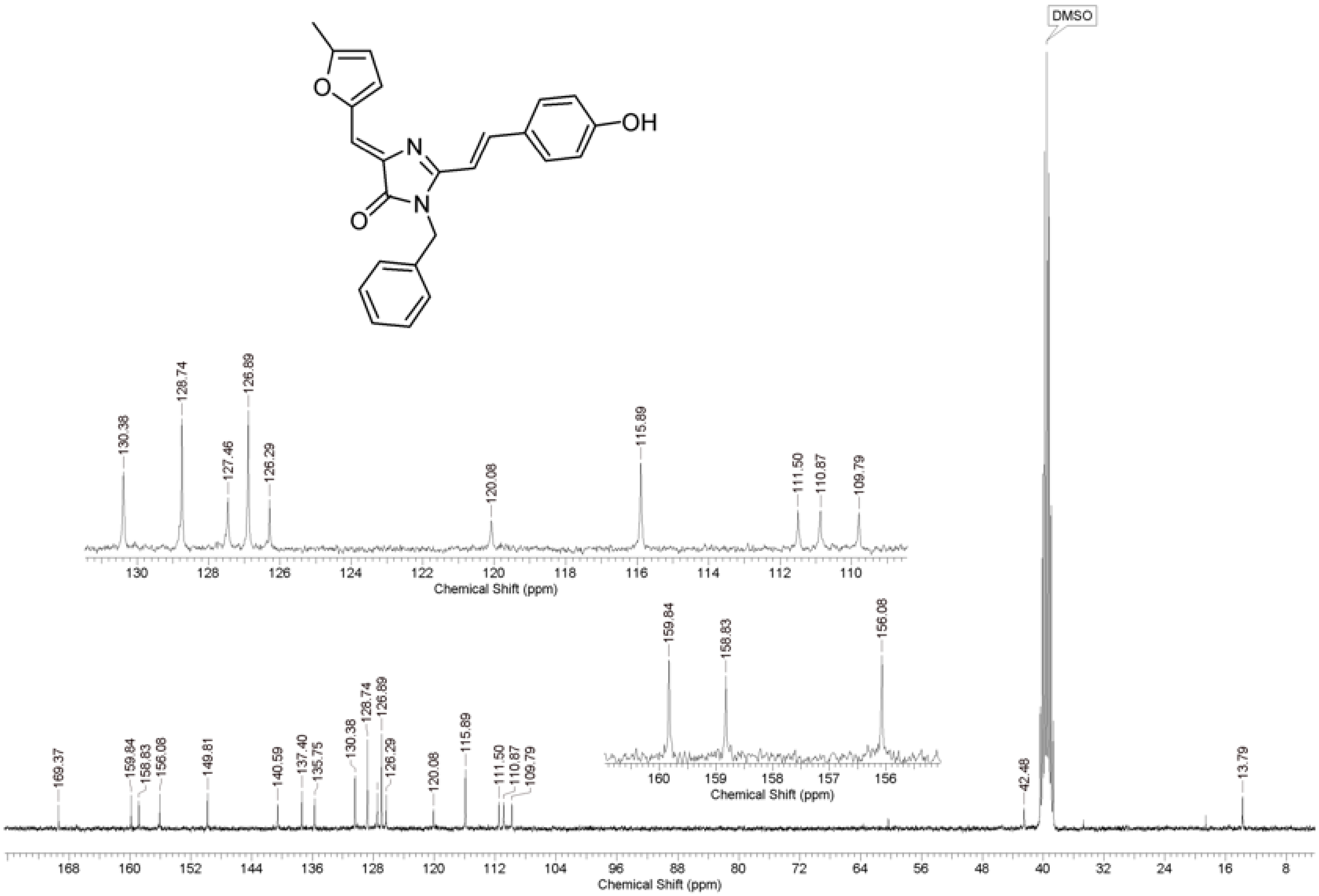
DMSO
OH
N
O
169.37
159.84
158.83
156.08
149.81
140.59
137.40
135.75
130.38
128.74
127.46
126.89
126.29
120.08
115.89
111.50
110.87
109.79
42.48
13.79
Chemical Shift (ppm)

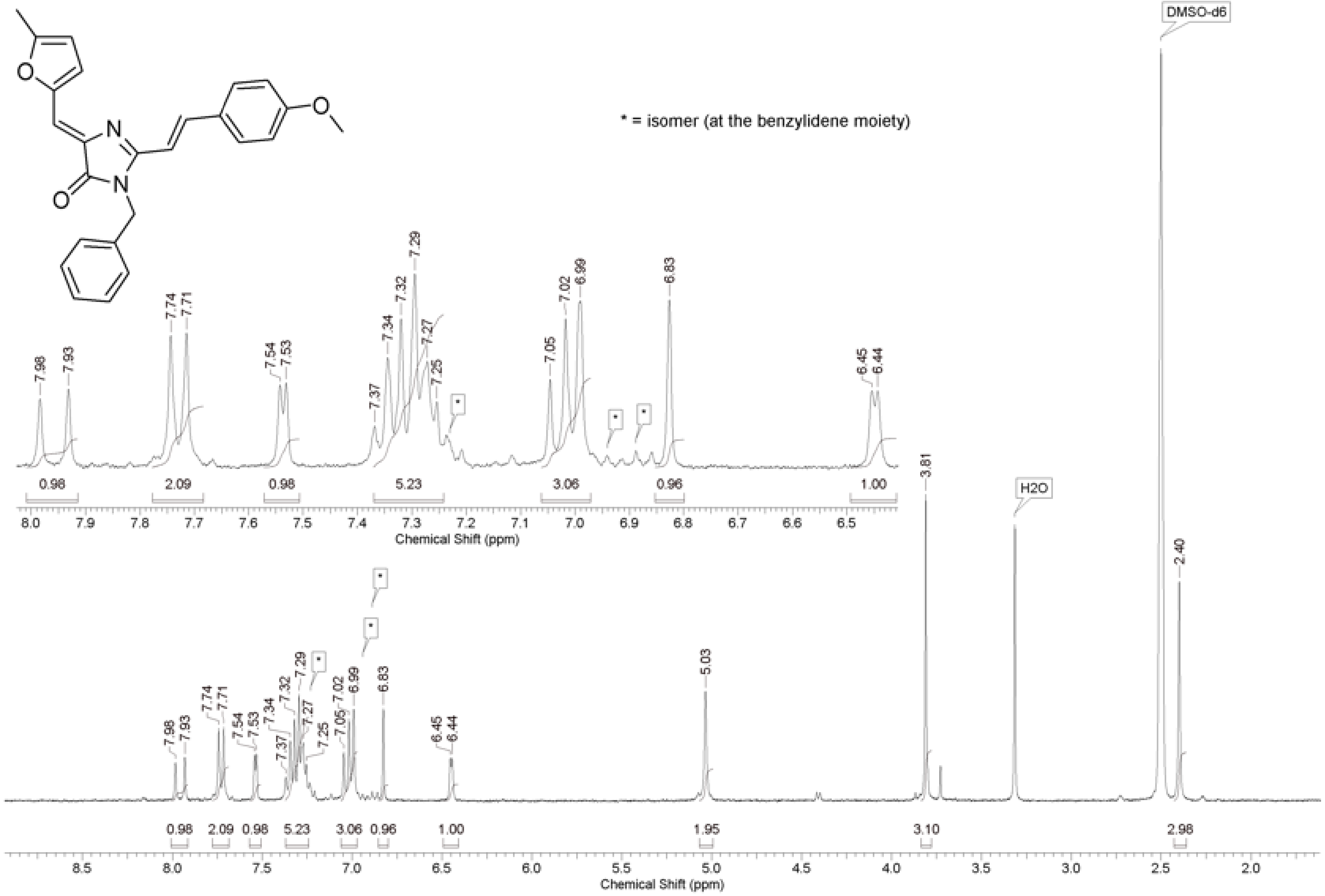
* = isomer (at the benzylidene moiety)
DMSO-d6
H2O
7.98
7.93
7.74
7.71
7.54
7.53
7.37
7.34
7.32
7.29
7.27
7.25
7.05
7.02
6.99
6.83
6.45
6.44
5.03
3.81
2.40
0.98
2.09
0.98
5.23
3.06
0.96
1.00
1.95
3.10
2.98
Chemical Shift (ppm)

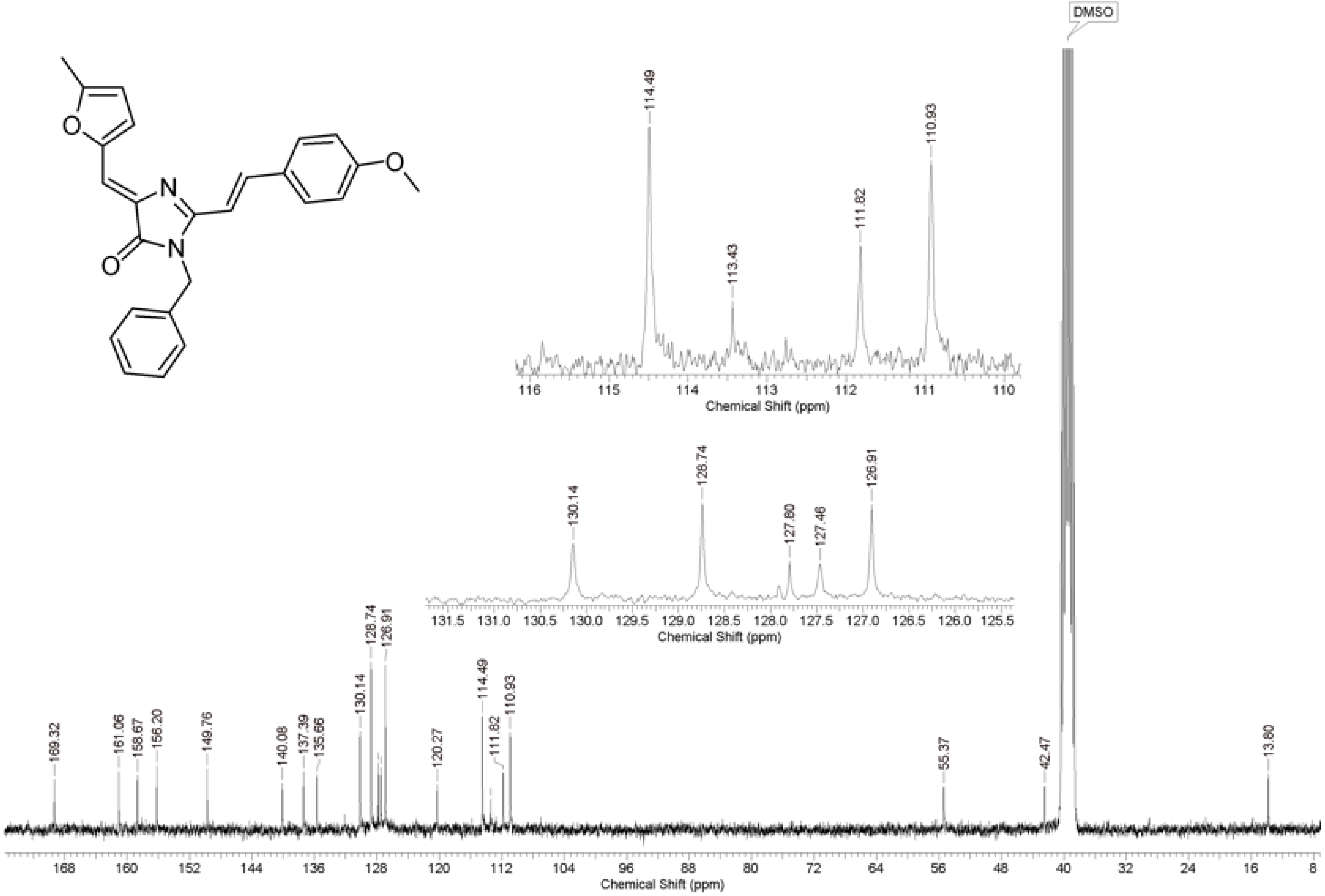
O
N
N
O
DMSO
114.49
113.43
111.82
110.93
116 115 114 113 112 111 110
Chemical Shift (ppm)
130.14
128.74
127.80
127.46
126.91
131.5 131.0 130.5 130.0 129.5 129.0 128.5 128.0 127.5 127.0 126.5 126.0 125.5
Chemical Shift (ppm)
169.32
161.06
158.67
156.20
149.76
140.08
137.39
135.66
130.14
128.74
126.91
120.27
114.49
111.82
110.93
55.37
42.47
13.80
168 160 152 144 136 128 120 112 104 96 88 80 72 64 56 48 40 32 24 16 8
Chemical Shift (ppm)

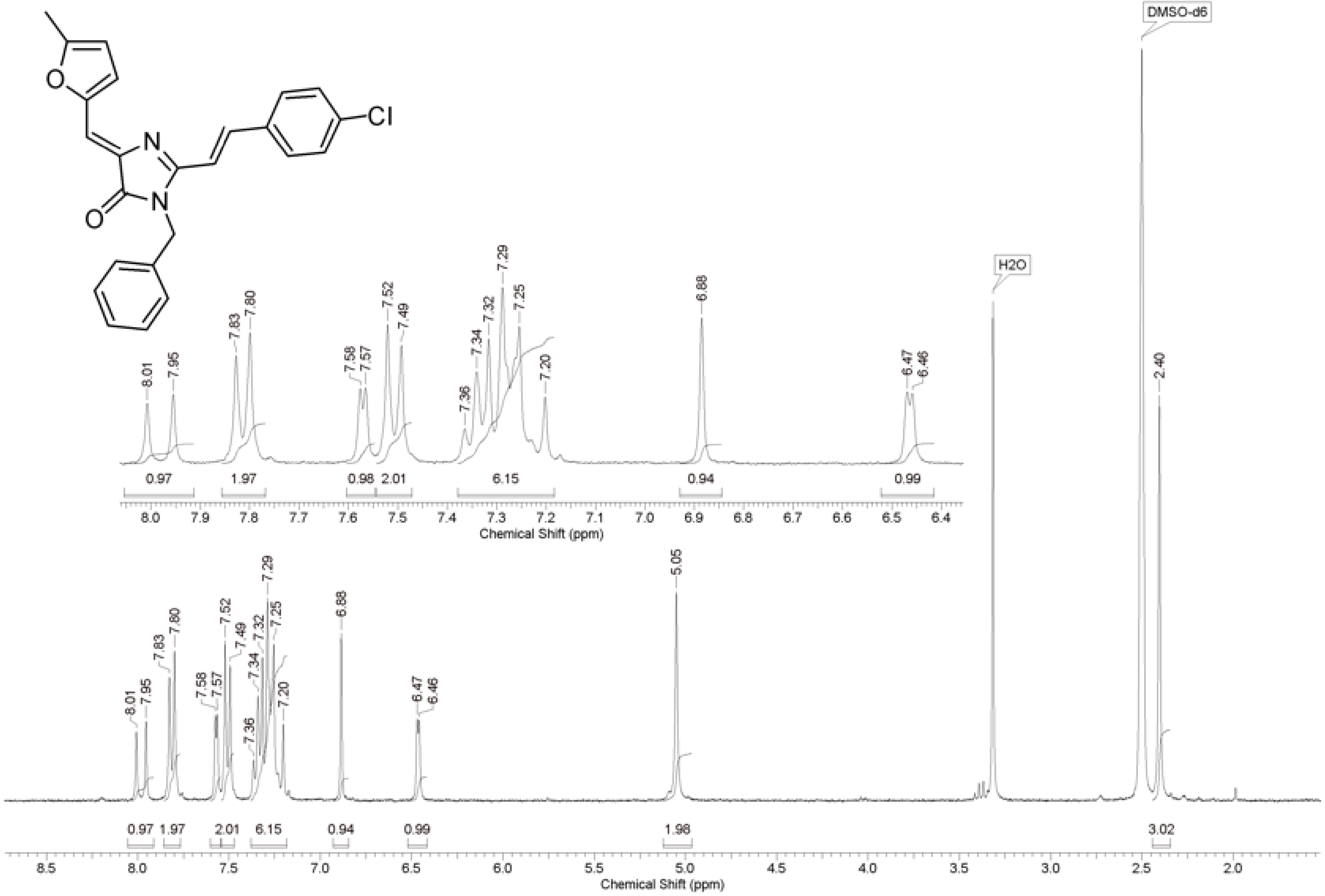
Cl
N
O
N
O
DMSO-d6
H2O
8.01
7.95
7.83
7.80
7.58
7.57
7.52
7.49
7.36
7.34
7.32
7.29
7.25
7.20
6.88
6.47
6.46
5.05
2.40
0.97
1.97
0.98
2.01
6.15
0.94
0.99
1.98
3.02
Chemical Shift (ppm)

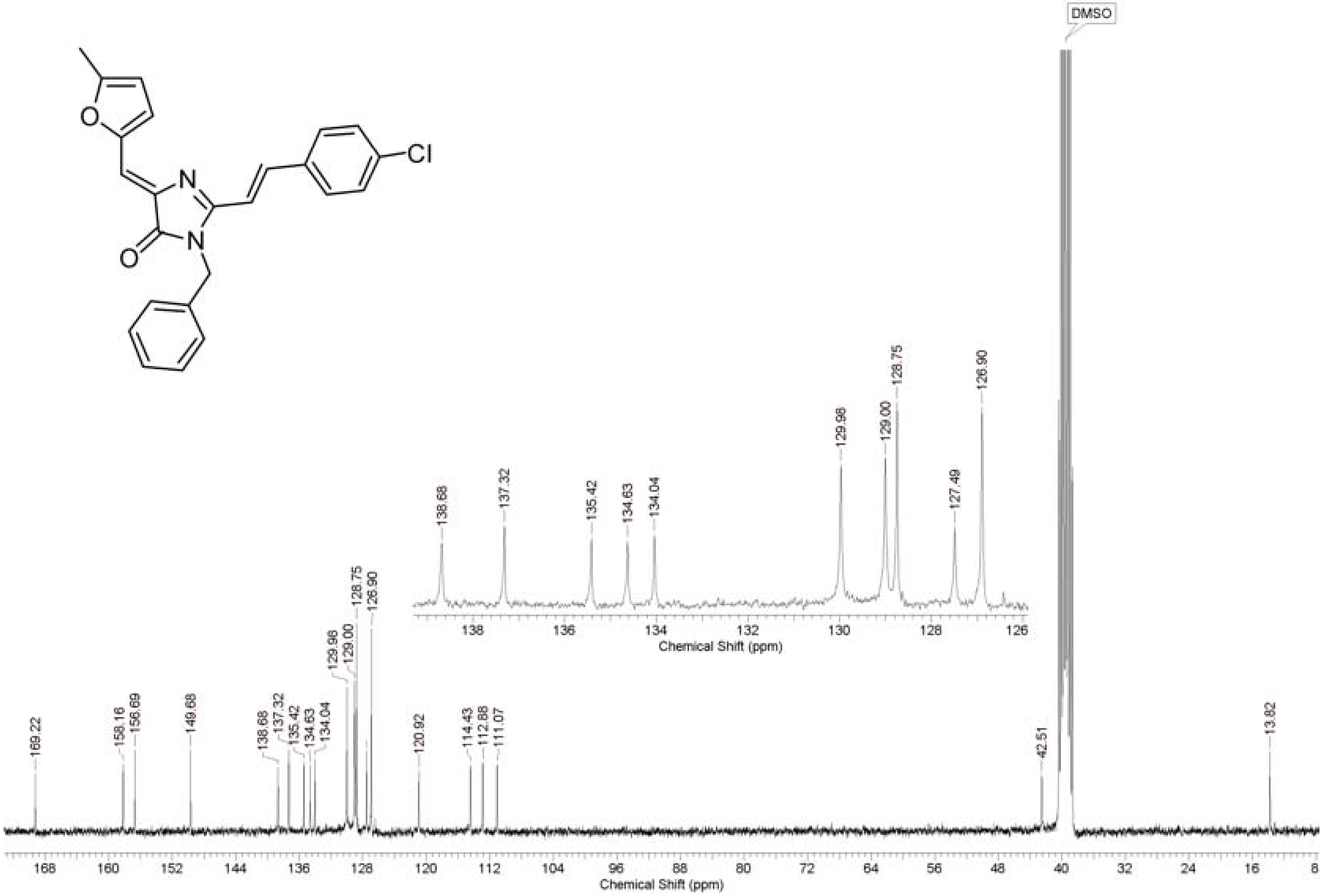
O
N
N
O
Cl
DMSO
169.22
158.16
156.69
149.68
138.68
137.32
135.42
134.63
134.04
129.98
129.00
128.75
126.90
127.49
120.92
114.43
112.88
111.07
42.51
13.82
Chemical Shift (ppm)

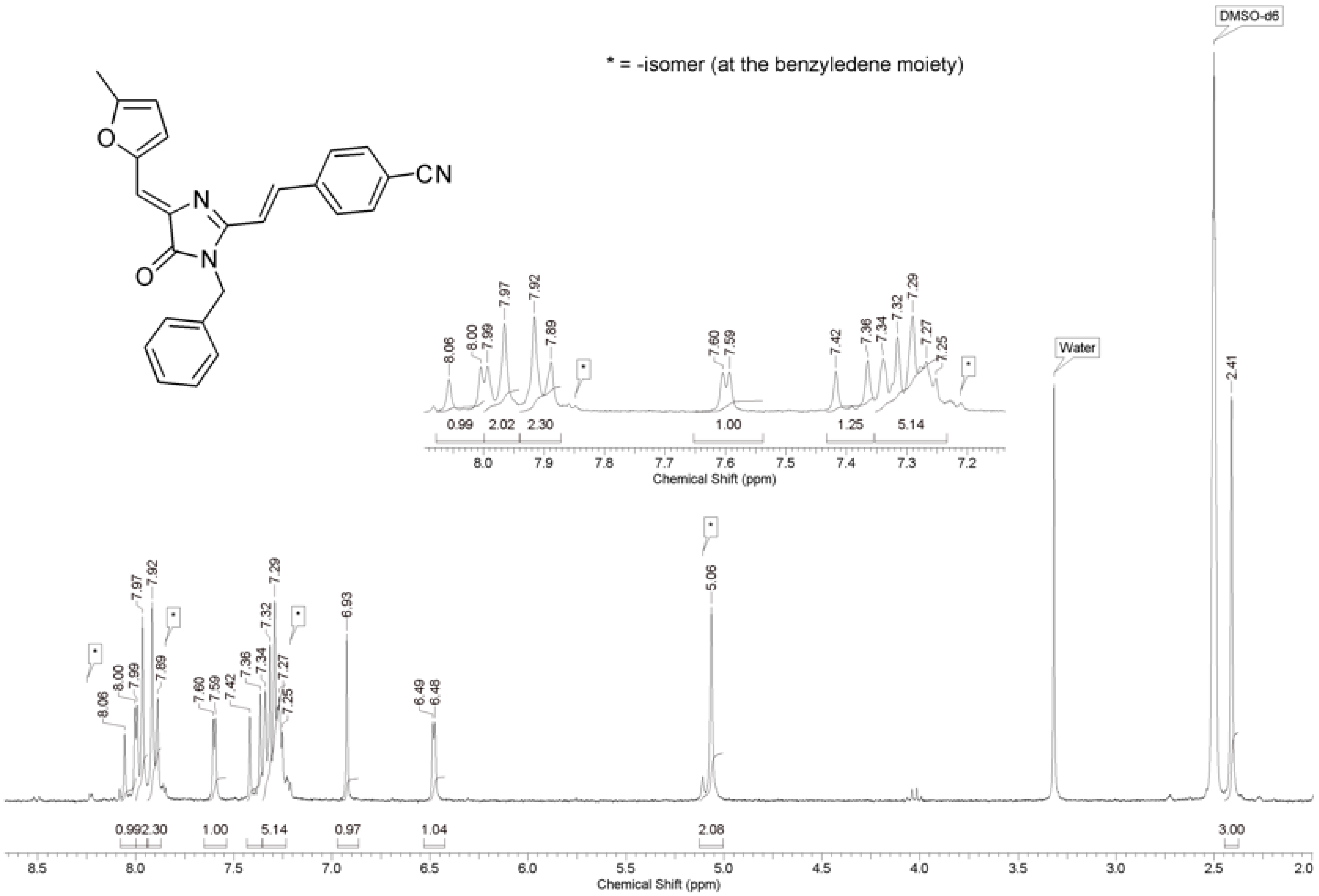

* = -isomer (at the benzyledene moiety)
CN
N
N
O
O
DMSO-d6
Water
2.41
5.06
6.93
6.49
6.48
8.06
8.00
7.99
7.97
7.92
7.89
7.60
7.59
7.42
7.36
7.34
7.32
7.29
7.27
7.25
0.99
2.02
2.30
1.00
1.25
5.14
0.97
1.04
2.08
3.00
Chemical Shift (ppm)

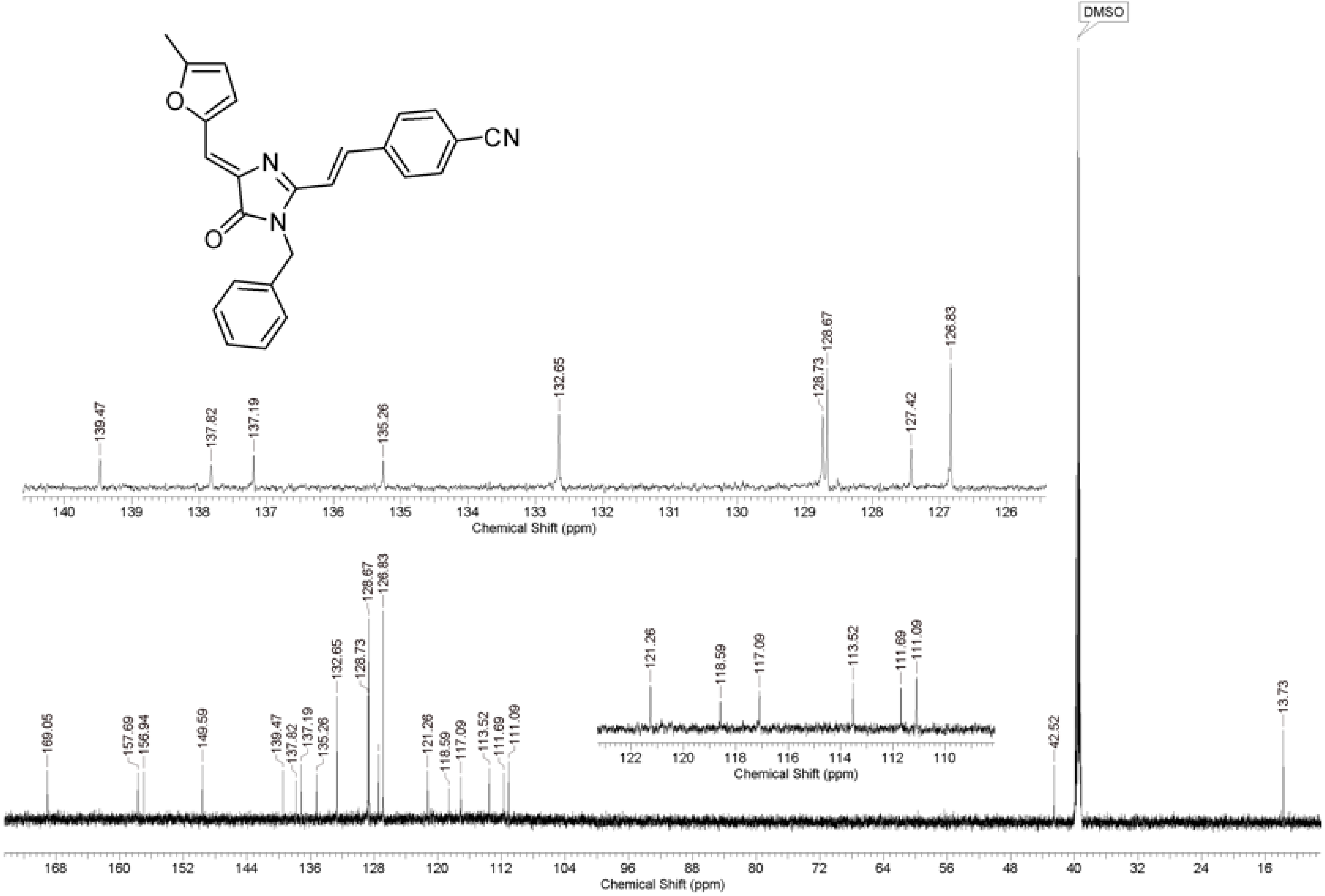
CN
N
N
O
O
DMSO
169.05
157.69
156.94
149.59
139.47
137.82
137.19
135.26
132.65
128.73
128.67
126.83
127.42
121.26
118.59
117.09
113.52
111.69
111.09
42.52
13.73
Chemical Shift (ppm)

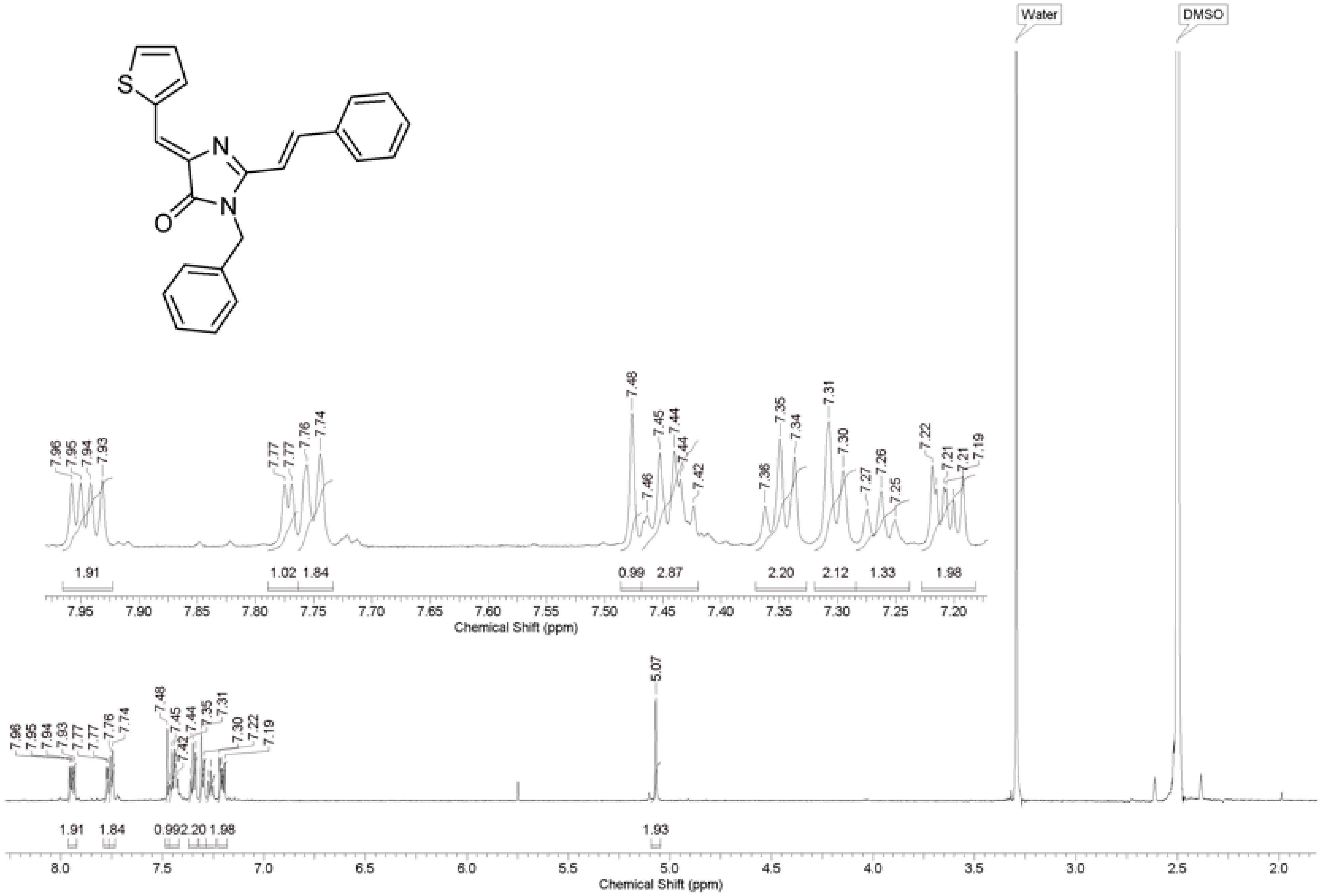
Water
DMSO
Chemical Shift (ppm)
Chemical Shift (ppm)

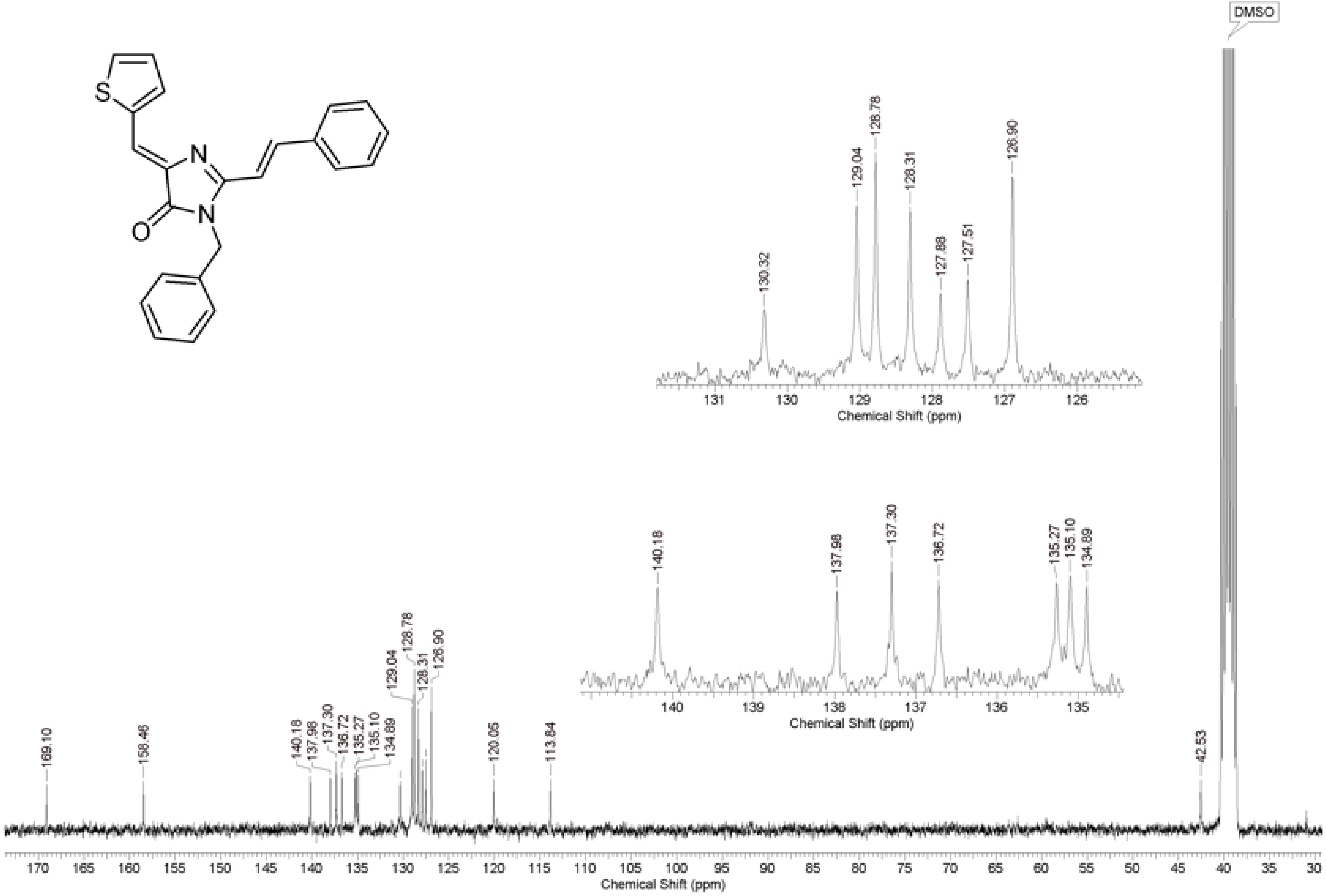
S
N
N
O
DMSO
169.10
158.46
140.18
137.98
137.30
136.72
135.27
135.10
134.89
129.04
128.78
128.31
126.90
120.05
113.84
42.53
130.32
127.88
127.51
Chemical Shift (ppm)

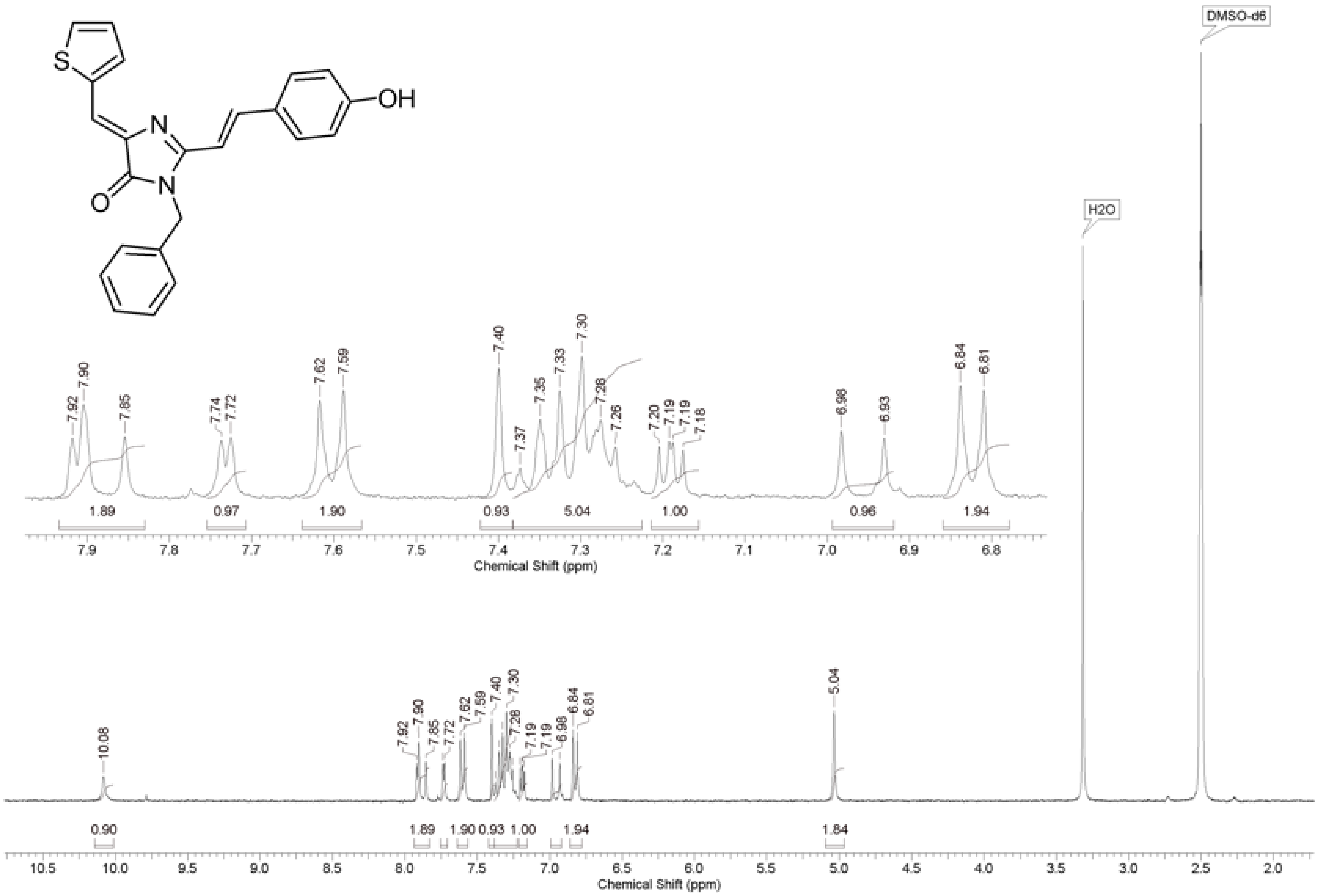

S
N
N
O
OH
DMSO-d6
H2O
7.92 7.90 7.85 7.74 7.72 7.62 7.59 7.40 7.37 7.35 7.33 7.30 7.28 7.26 7.20 7.19 7.19 7.18 6.98 6.93 6.84 6.81
1.89 0.97 1.90 0.93 5.04 1.00 0.96 1.94
Chemical Shift (ppm)
10.08 7.92 7.90 7.85 7.72 7.62 7.59 7.40 7.30 7.28 7.19 7.19 6.98 6.84 6.81 5.04
0.90 1.89 1.90 0.93 1.00 1.94 1.84
Chemical Shift (ppm)

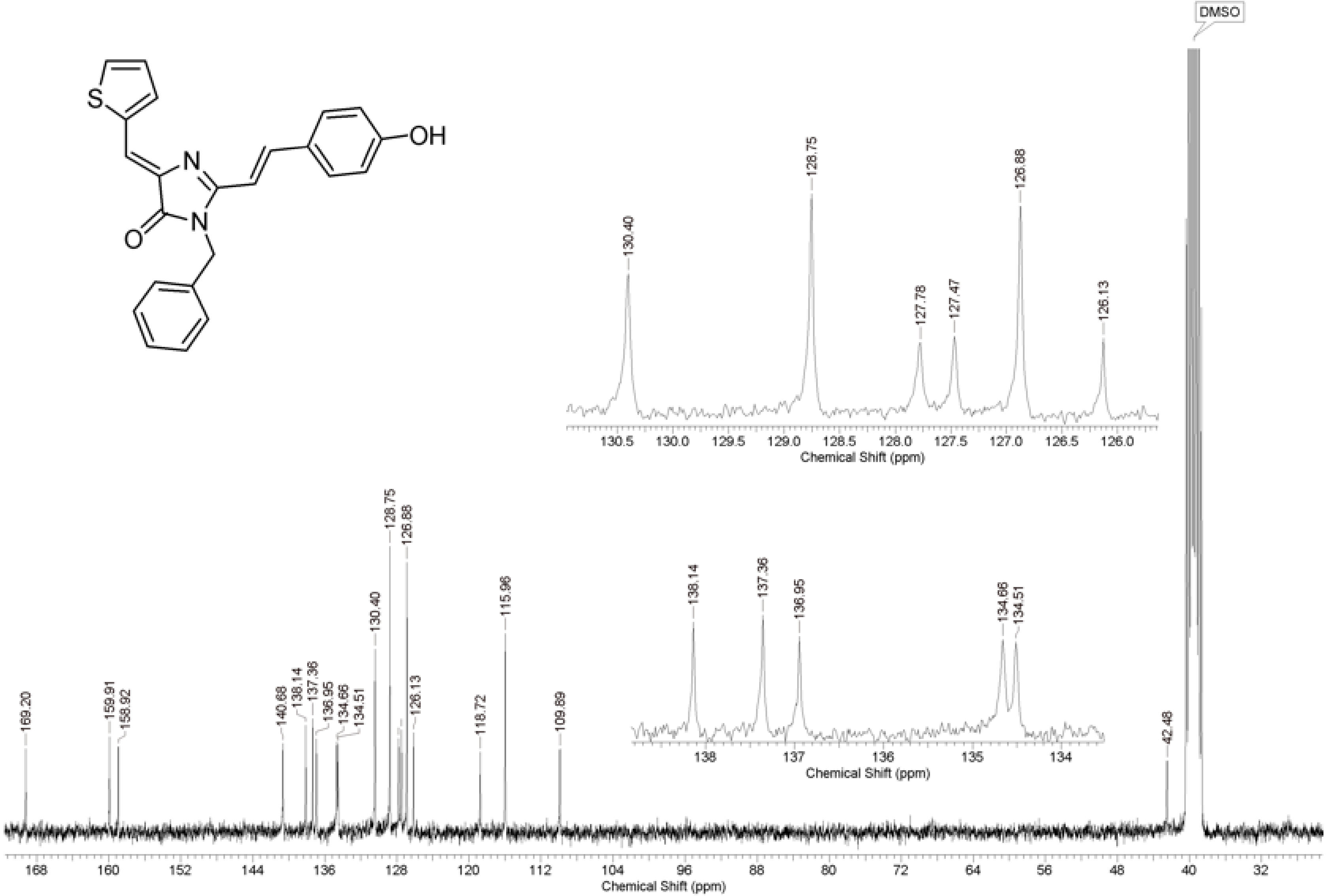
S
N
OH
O
N
DMSO
169.20
159.91
158.92
140.68
138.14
137.36
136.95
134.66
134.51
130.40
128.75
126.88
126.13
118.72
115.96
109.89
42.48
Chemical Shift (ppm)
Chemical Shift (ppm)
Chemical Shift (ppm)

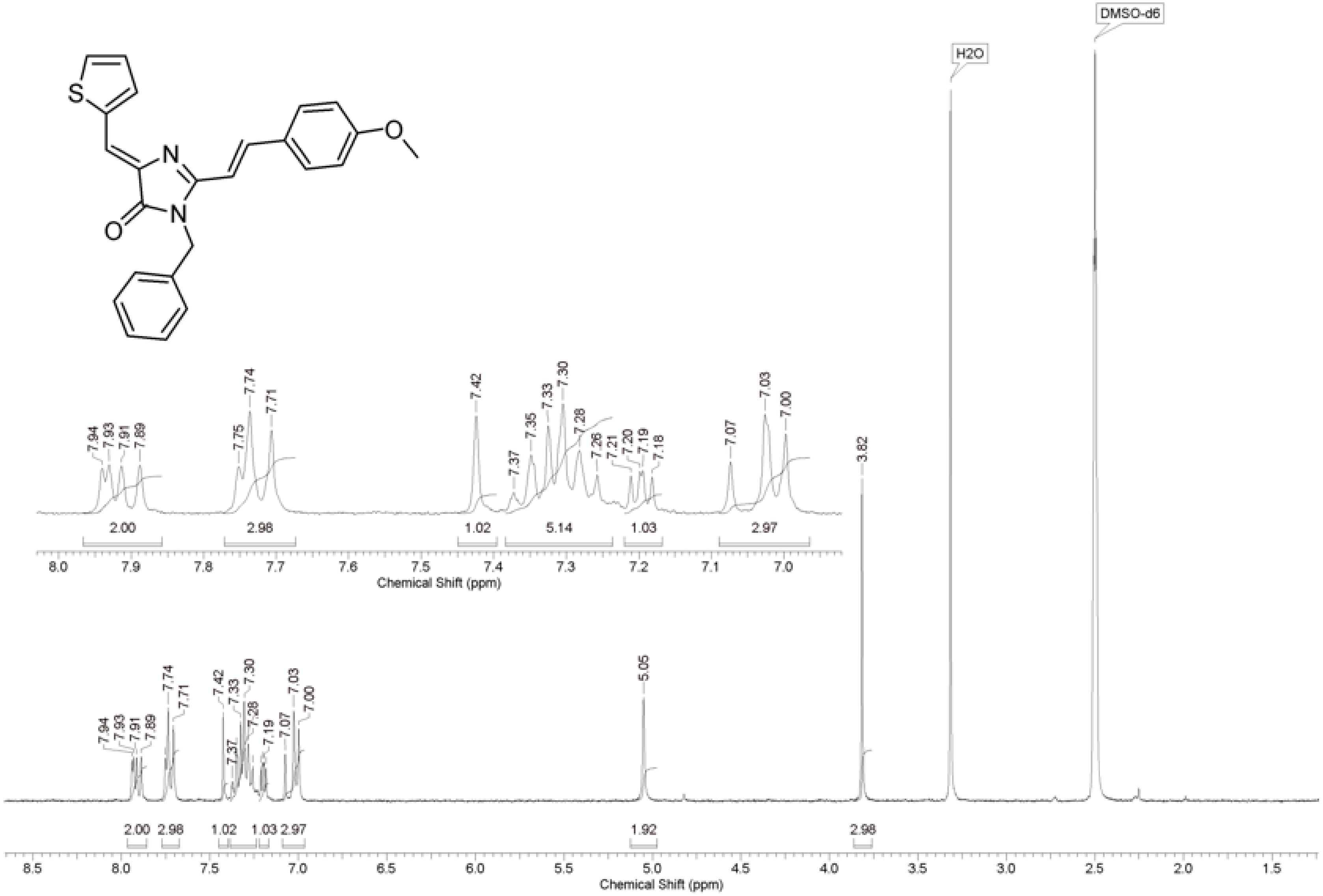
DMSO-d6
H2O
3.82
5.05
2.00
2.98
1.02
5.14
1.03
2.97
1.92
Chemical Shift (ppm)

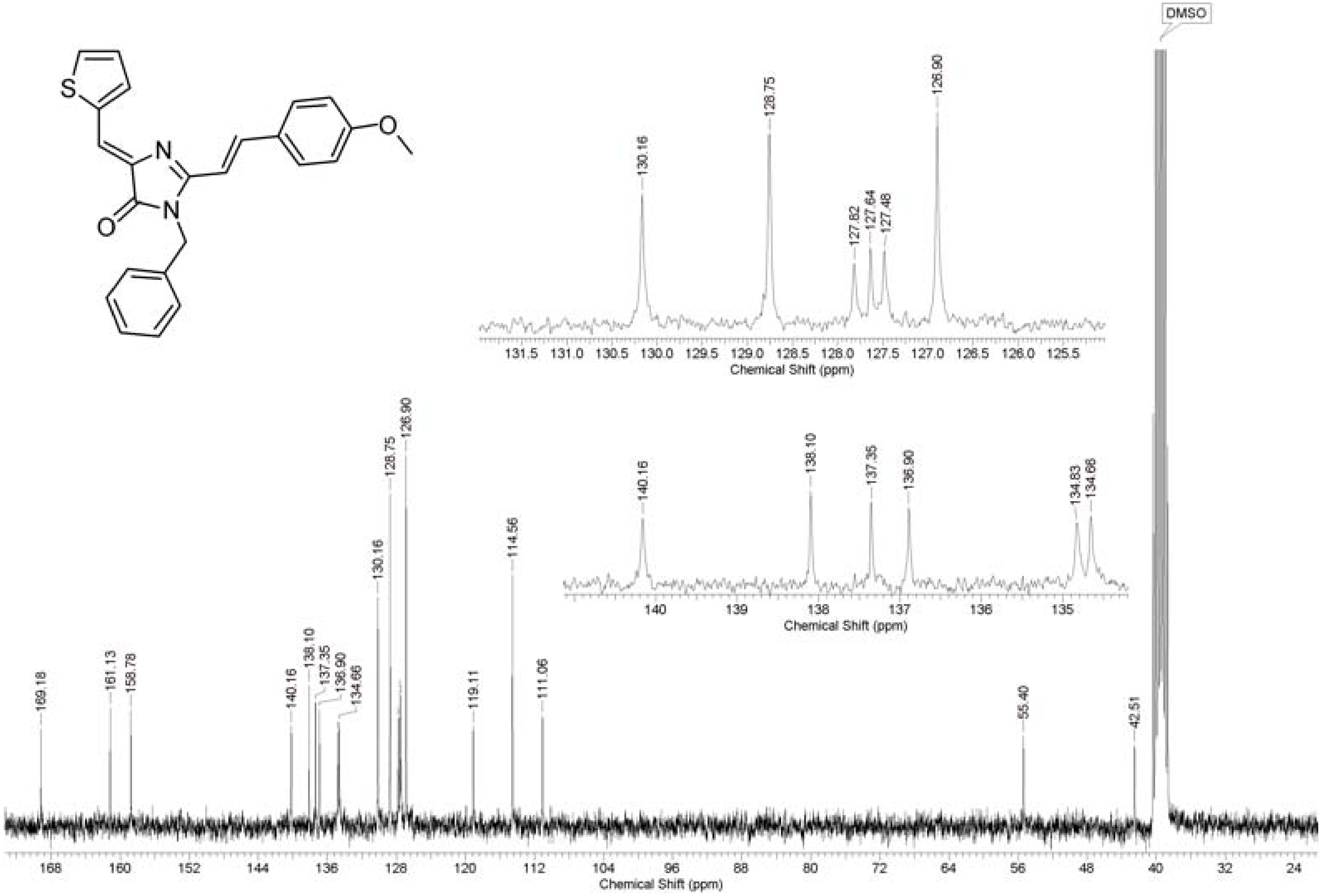
DMSO
130.16
128.75
127.82
127.64
127.48
126.90
131.5 131.0 130.5 130.0 129.5 129.0 128.5 128.0 127.5 127.0 126.5 126.0 125.5
Chemical Shift (ppm)
140.16
138.10
137.35
136.90
134.83
134.68
140 139 138 137 136 135
Chemical Shift (ppm)
169.18
161.13
158.78
140.16
138.10
137.35
136.90
134.66
130.16
128.75
126.90
119.11
114.56
111.06
55.40
42.51
168 160 152 144 136 128 120 112 104 96 88 80 72 64 56 48 40 32 24
Chemical Shift (ppm)

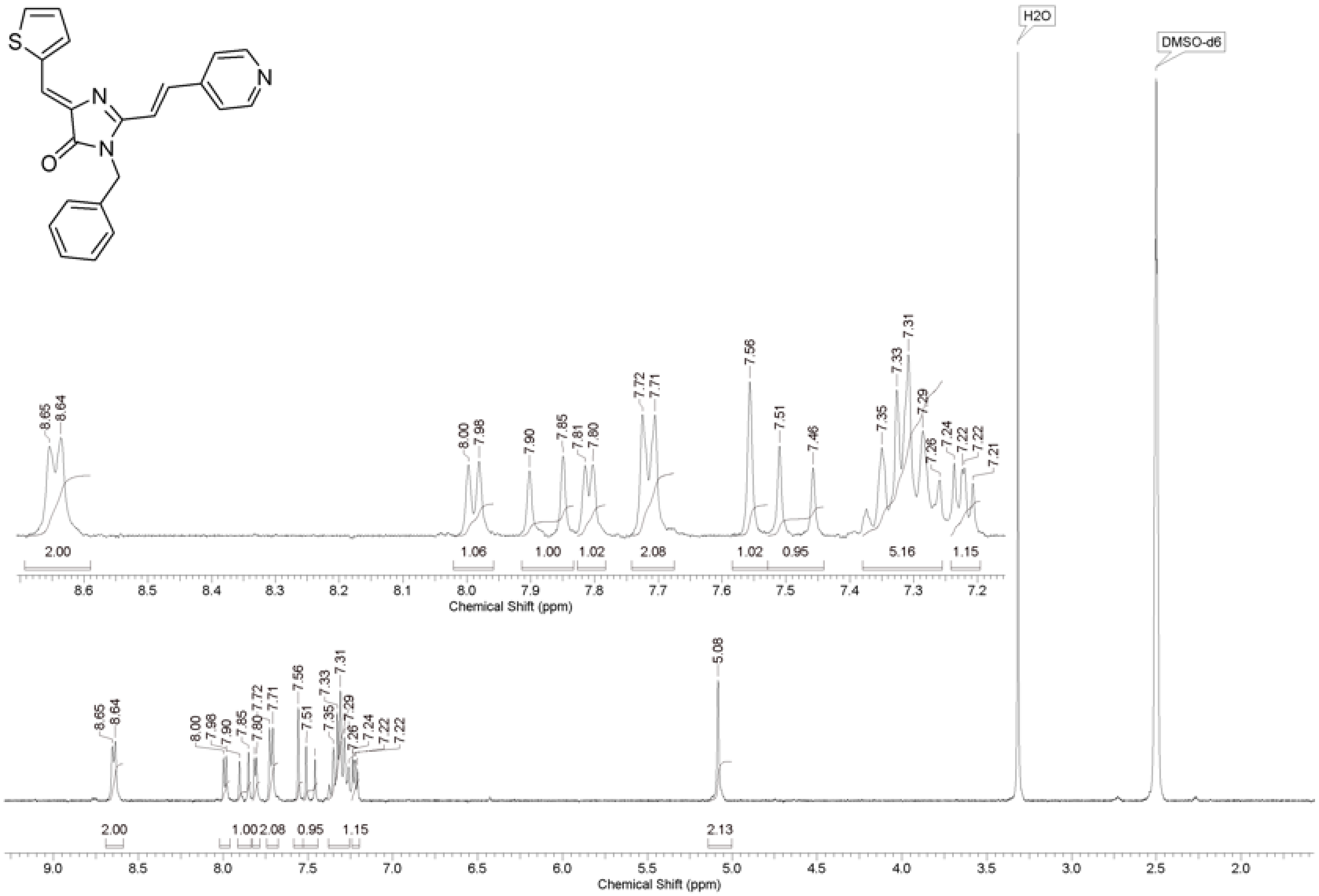
S
N
N
N
O
H2O
DMSO-d6
8.65 8.64
8.00 7.98
7.90
7.85
7.81 7.80
7.72 7.71
7.56
7.51
7.46
7.35
7.33
7.31
7.29
7.26
7.24
7.22
7.22
7.21
2.00
1.06
1.00
1.02
2.08
1.02
0.95
5.16
1.15
Chemical Shift (ppm)
5.08
2.13
Chemical Shift (ppm)

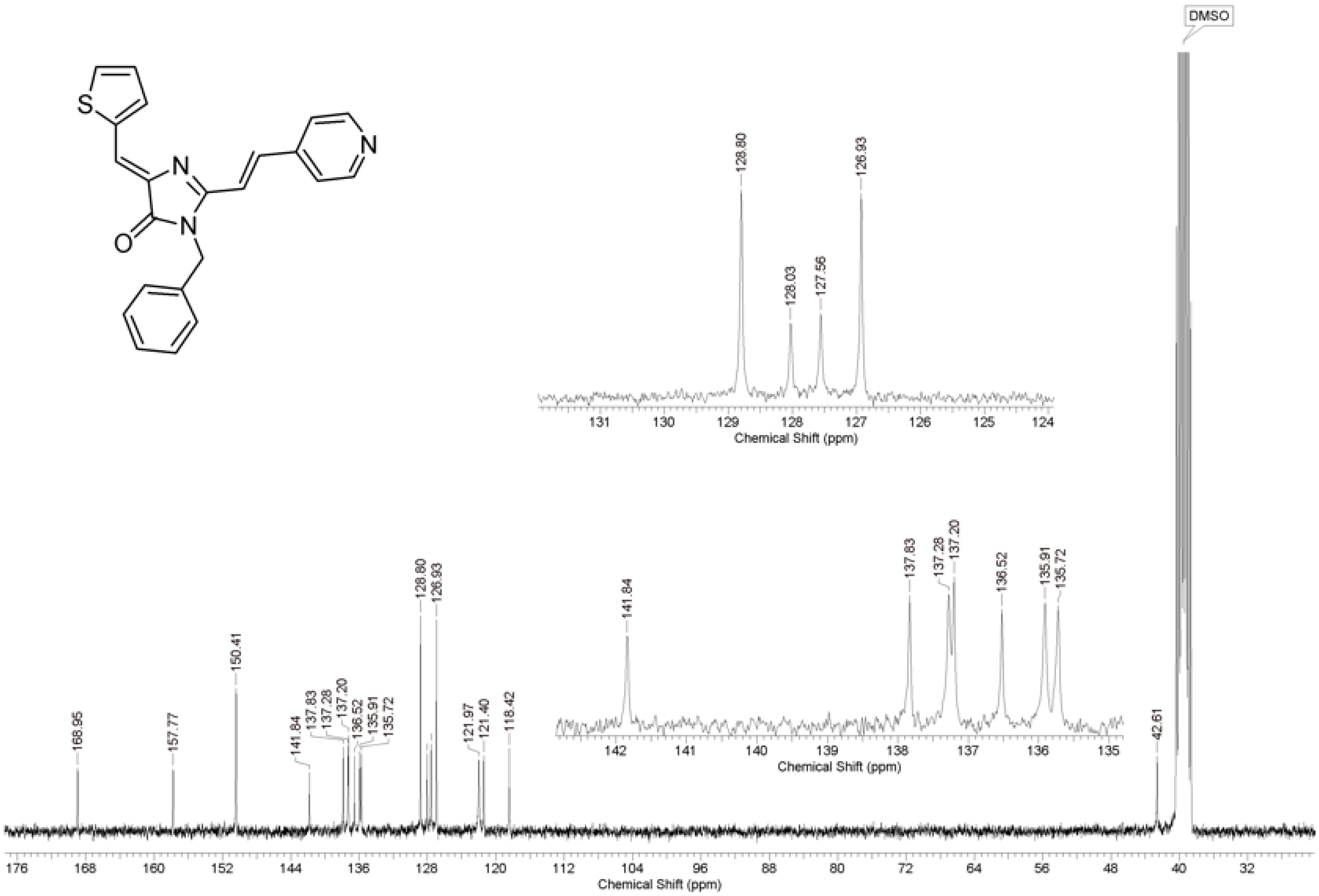
DMSO
168.95
157.77
150.41
141.84
137.83
137.28
137.20
136.52
135.91
135.72
128.80
126.93
121.97
121.40
118.42
42.61
128.03
127.56
Chemical Shift (ppm)

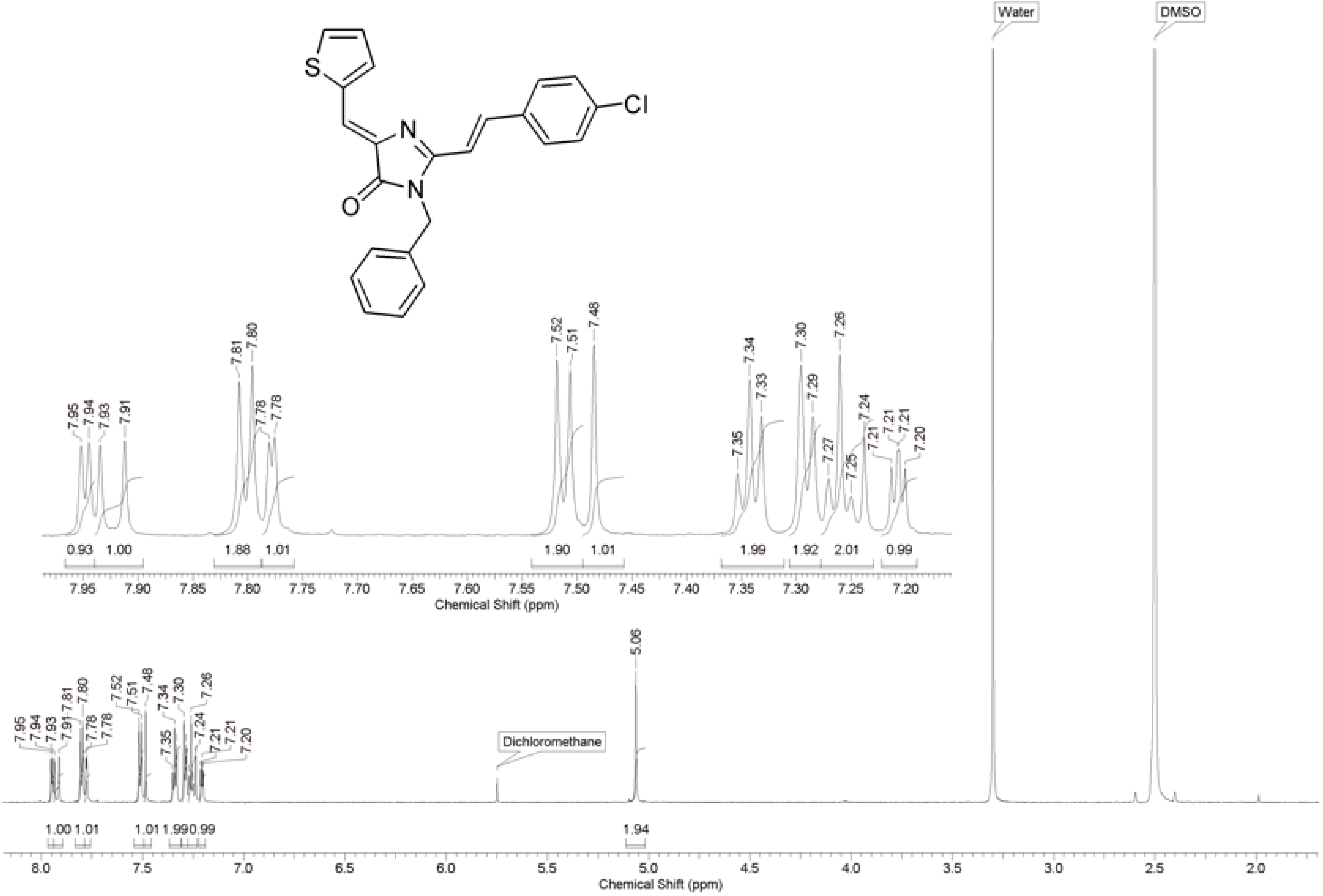
S
N
N
O
Cl
Water
DMSO
Dichloromethane
7.95 7.94 7.93 7.91
7.81 7.80 7.78 7.78
7.52 7.51 7.48
7.35 7.34 7.33
7.30 7.29 7.27 7.26 7.25 7.24
7.21 7.21 7.20
0.93 1.00 1.88 1.01 1.90 1.01 1.99 1.92 2.01 0.99
Chemical Shift (ppm)
5.06
1.00 1.01 1.01 1.99 0.99
1.94
Chemical Shift (ppm)

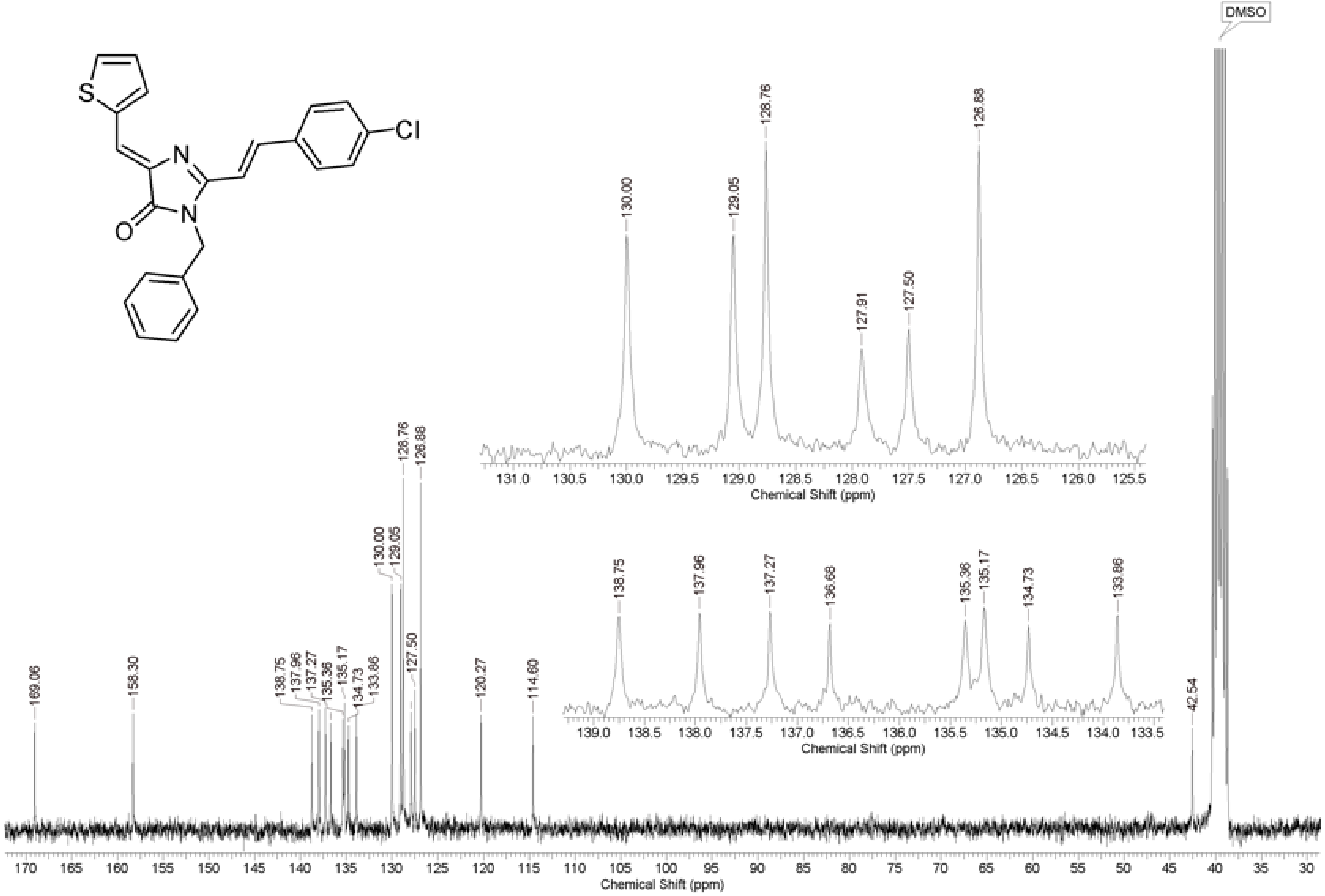
DMSO
169.06
158.30
138.75
137.96
137.27
135.36
135.17
134.73
133.86
130.00
129.05
128.76
127.50
126.88
120.27
114.60
42.54
Chemical Shift (ppm)
130.00
129.05
128.76
127.91
127.50
126.88
Chemical Shift (ppm)
138.75
137.96
137.27
136.68
135.36
135.17
134.73
133.86
Chemical Shift (ppm)

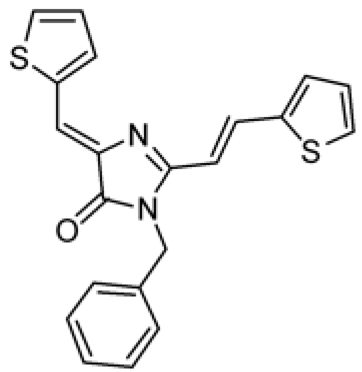

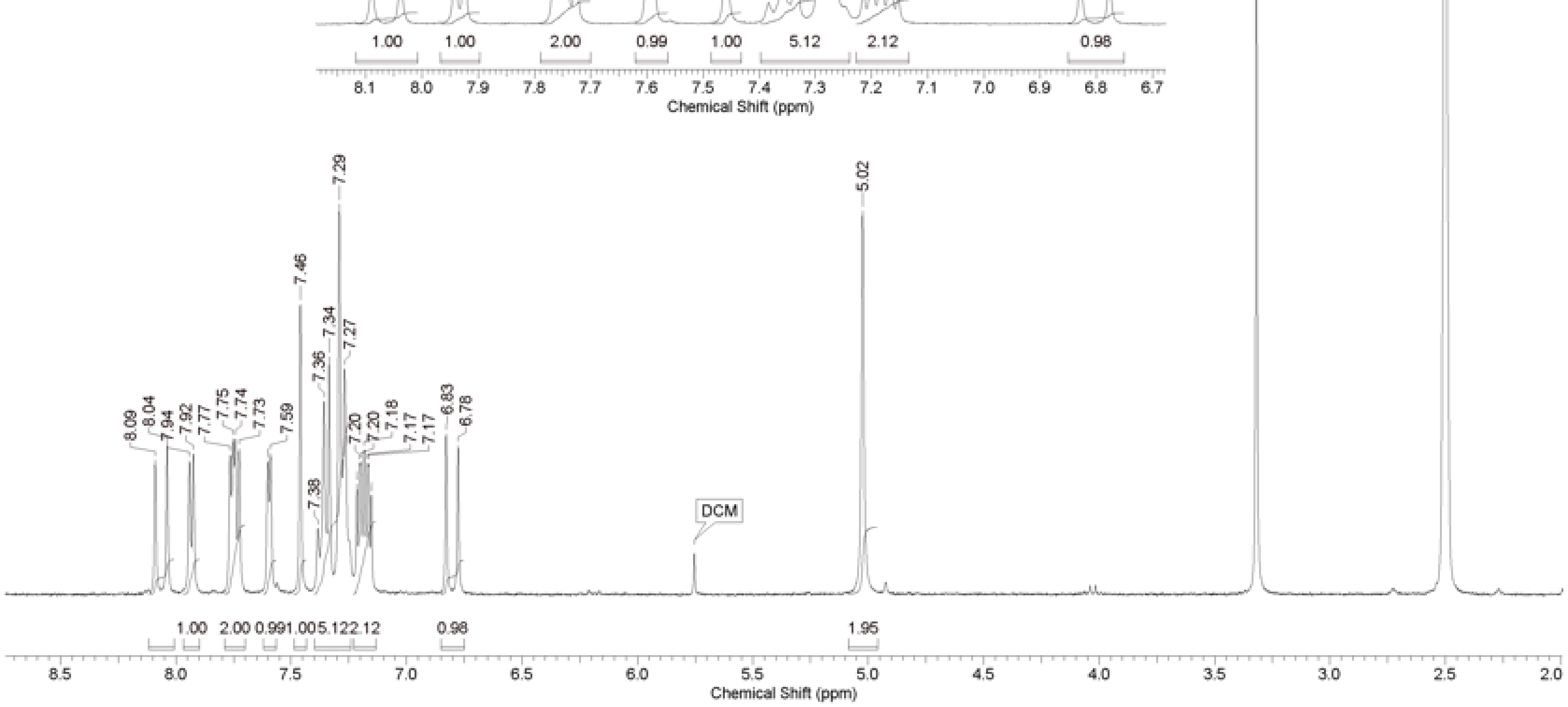

DMSO-d6
H2O
DCM
5.02
1.95
Chemical Shift (ppm)

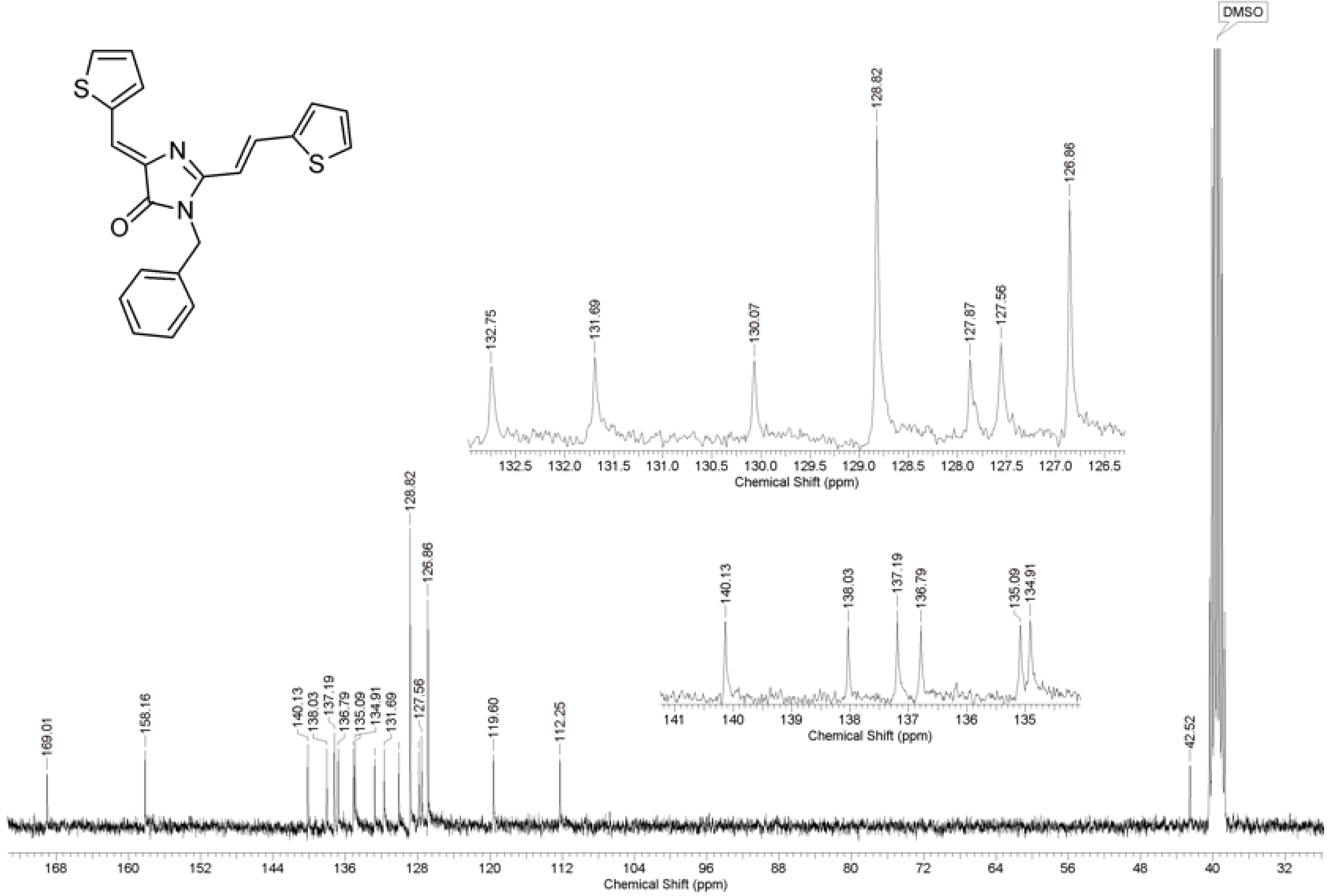
DMSO
128.82
126.86
132.75
131.69
130.07
127.87
127.56
132.5 132.0 131.5 131.0 130.5 130.0 129.5 129.0 128.5 128.0 127.5 127.0 126.5
Chemical Shift (ppm)
140.13
138.03
137.19
136.79
135.09
134.91
141 140 139 138 137 136 135
Chemical Shift (ppm)
169.01
158.16
140.13
138.03
137.19
136.79
135.09
134.91
131.69
127.56
119.60
112.25
42.52
168 160 152 144 136 128 120 112 104 96 88 80 72 64 56 48 40 32
Chemical Shift (ppm)

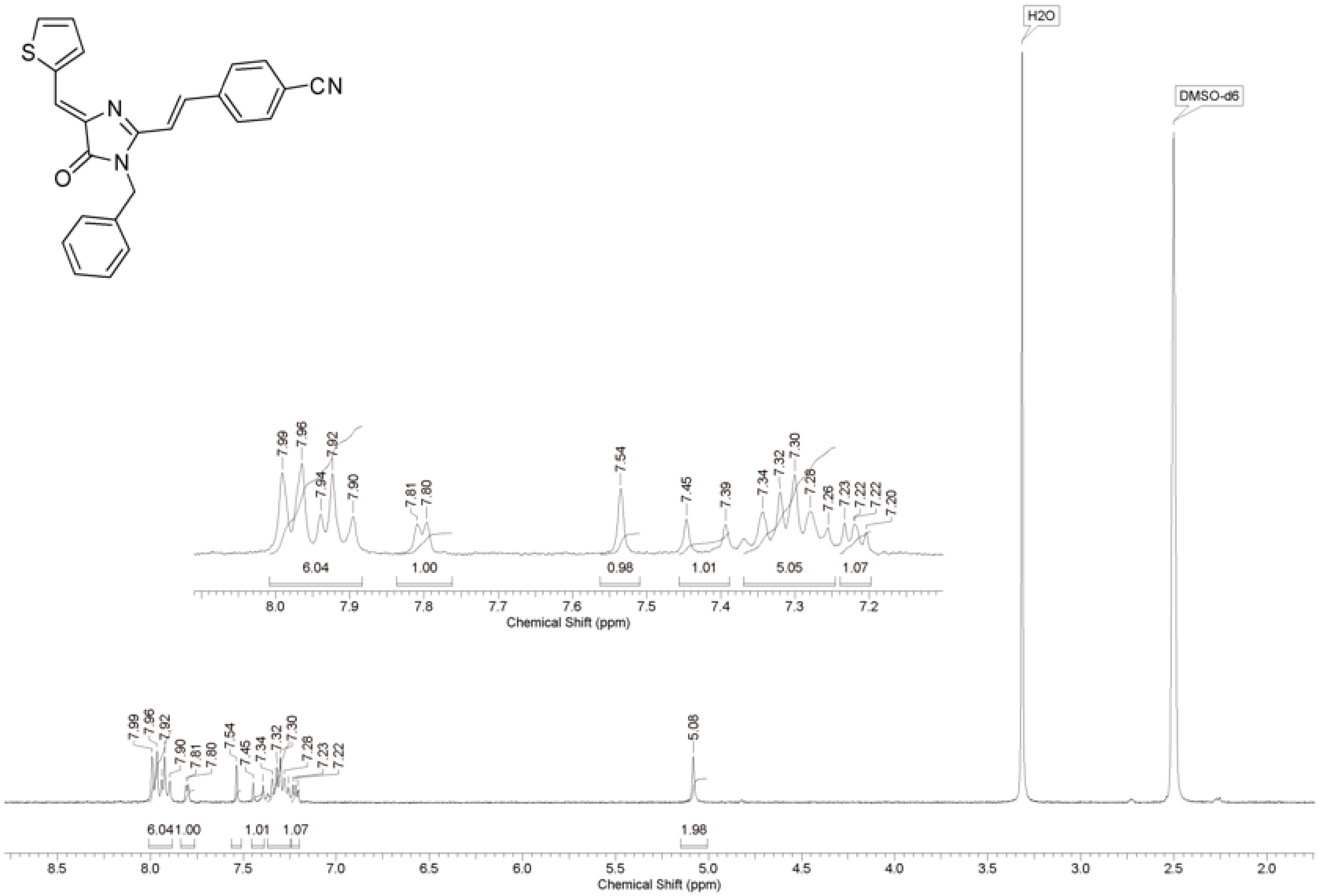
S
N
CN
O
N
H2O
DMSO-d6
7.99 7.96 7.92 7.94 7.90 7.81 7.80 7.54 7.45 7.39 7.34 7.32 7.30 7.28 7.26 7.23 7.22 7.22 7.20
6.04 1.00 0.98 1.01 5.05 1.07
Chemical Shift (ppm)
5.08
6.04 1.00 1.01 1.07 1.98
8.5 8.0 7.5 7.0 6.5 6.0 5.5 5.0 4.5 4.0 3.5 3.0 2.5 2.0
Chemical Shift (ppm)

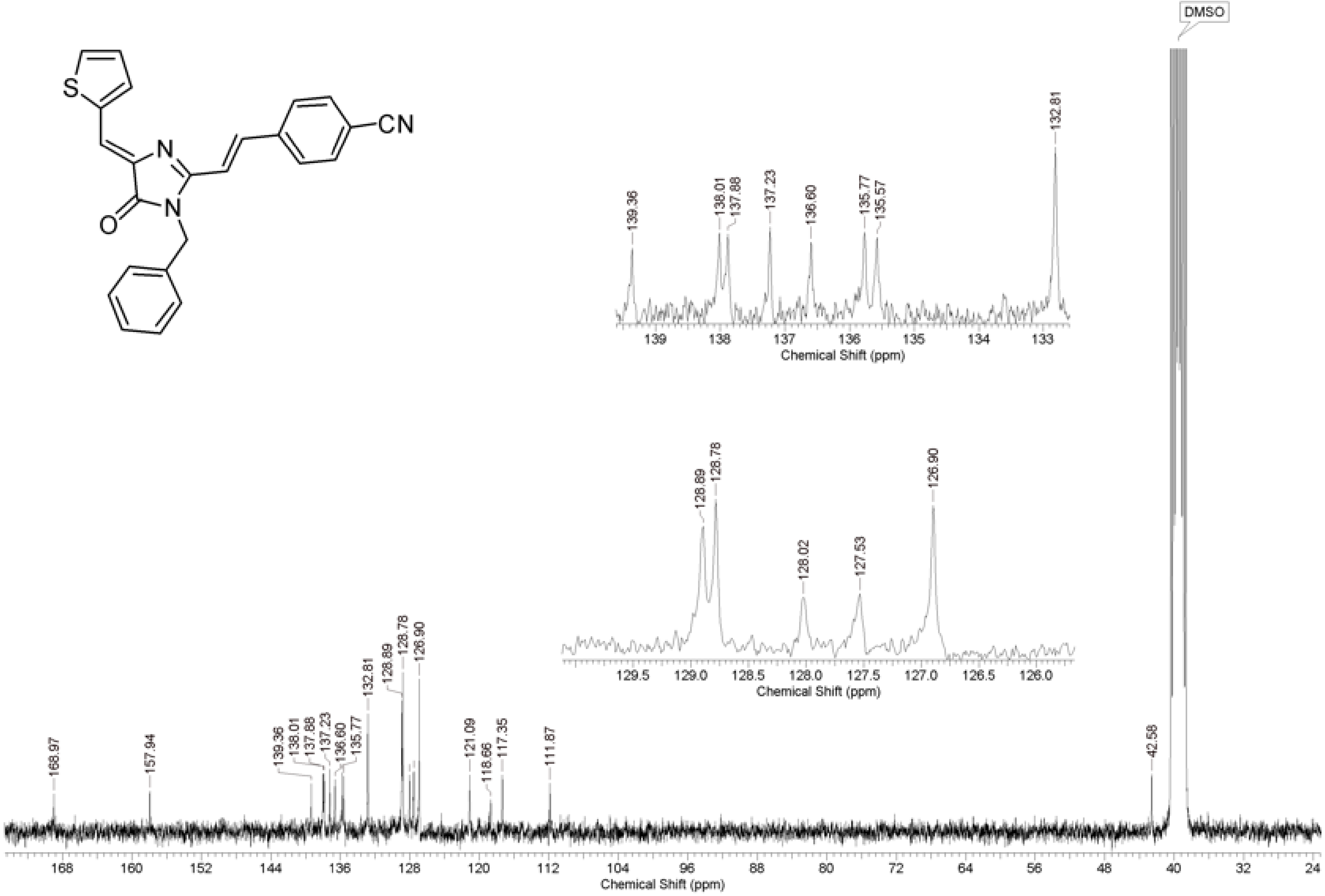
S
N
N
O
CN
DMSO
168.97
157.94
139.36
138.01
137.88
137.23
136.60
135.77
135.57
132.81
128.89
128.78
128.02
127.53
126.90
121.09
118.66
117.35
111.87
42.58
Chemical Shift (ppm)

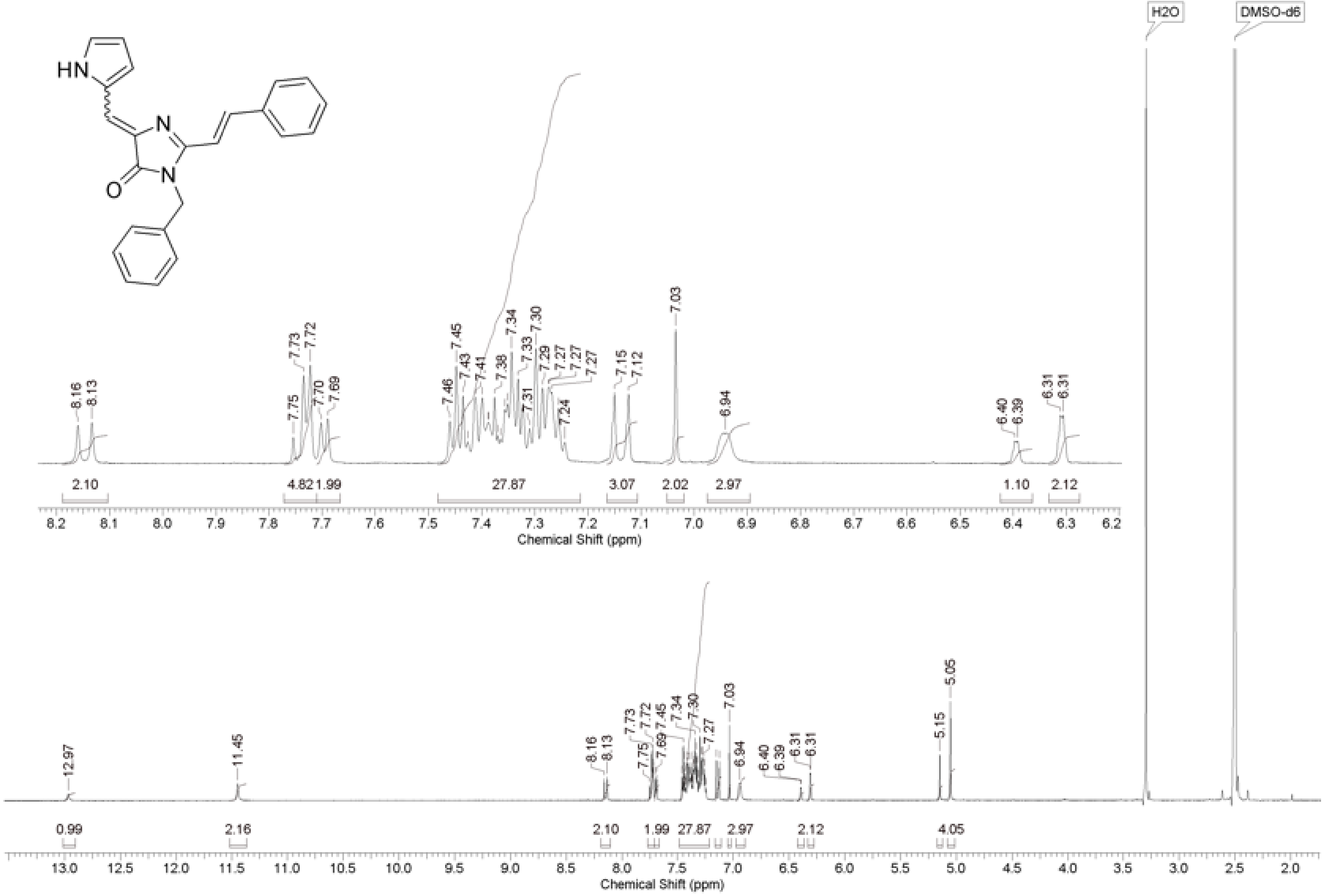
HN
N
N
O
H2O
DMSO-d6
Chemical Shift (ppm)
Chemical Shift (ppm)

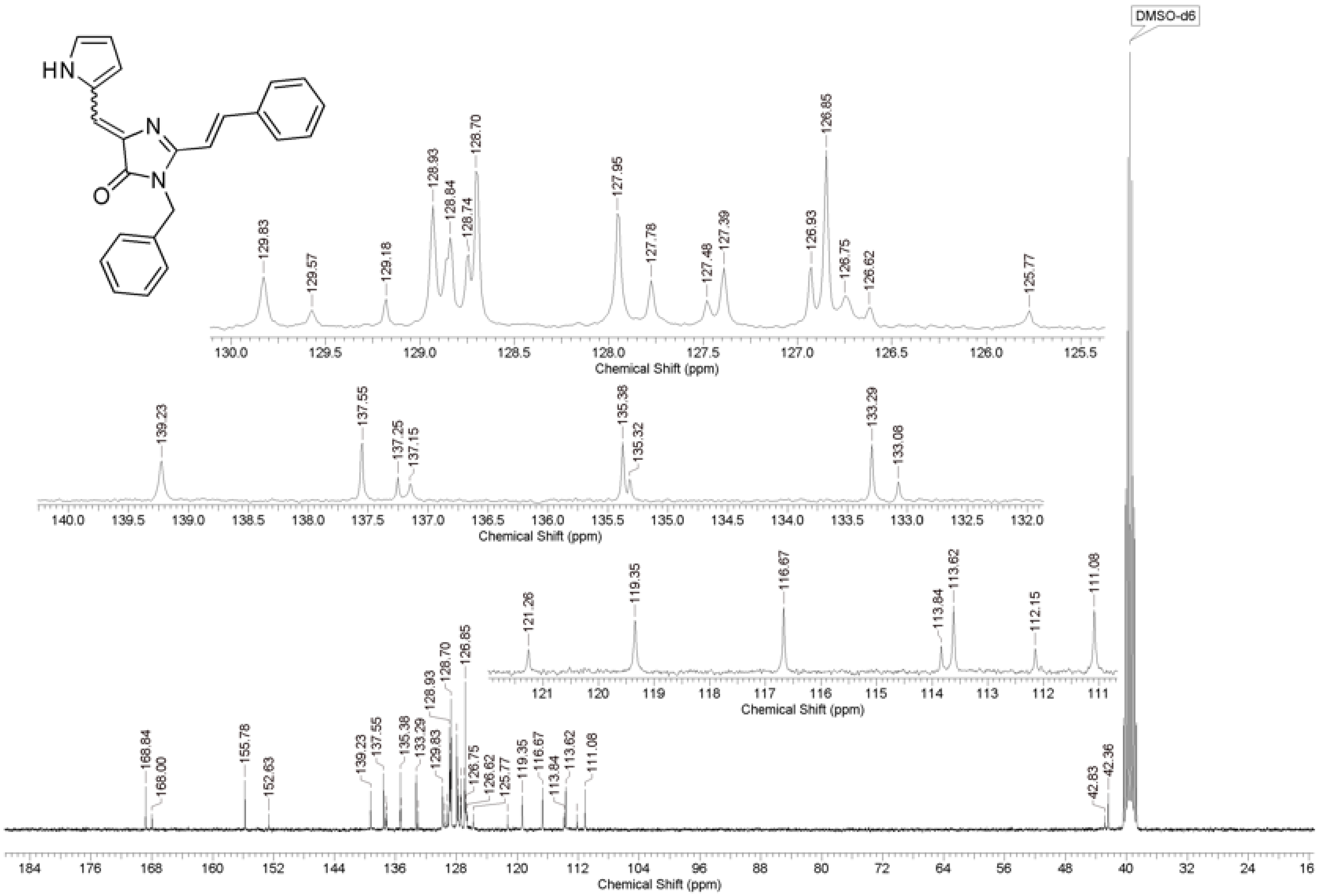
HN
N
O
N
DMSO-d6
129.83
129.57
129.18
128.93
128.84
128.74
128.70
127.95
127.78
127.48
127.39
126.93
126.85
126.75
126.62
125.77
Chemical Shift (ppm)
139.23
137.55
137.25
137.15
135.38
135.32
133.29
133.08
Chemical Shift (ppm)
121.26
119.35
116.67
113.84
113.62
112.15
111.08
Chemical Shift (ppm)
168.84
168.00
155.78
152.63
42.83
42.36
Chemical Shift (ppm)

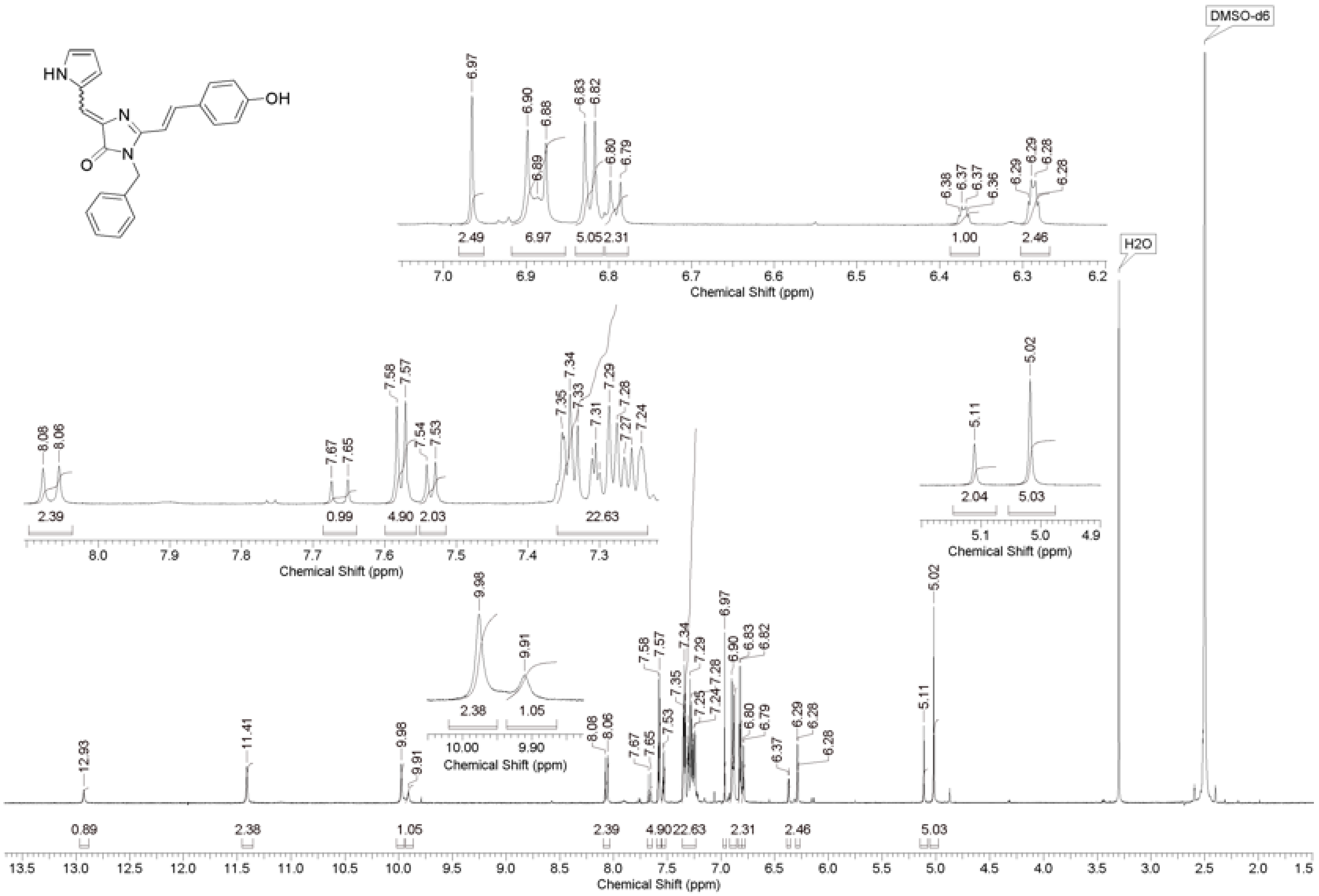
HN
N
OH
O
N
DMSO-d6
H2O
Chemical Shift (ppm)
2.49
6.97
5.05
2.31
1.00
2.46
2.39
0.99
4.90
2.03
22.63
2.04
5.03
2.38
1.05
0.89

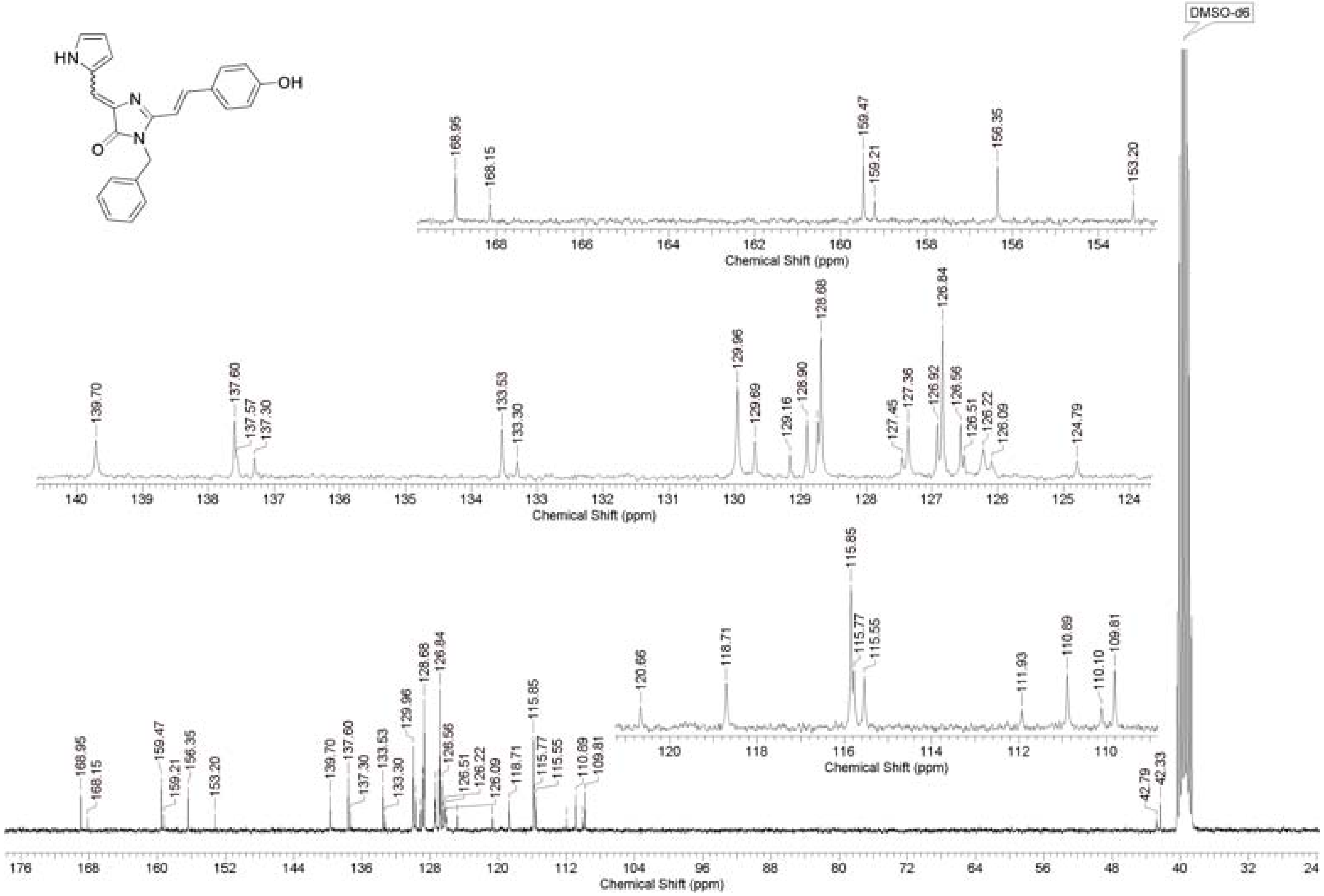
HN
OH
N
O
N
DMSO-d6
168.95
168.15
159.47
159.21
156.35
153.20
Chemical Shift (ppm)
139.70
137.60
137.57
137.30
133.53
133.30
129.96
129.69
129.16
128.90
128.68
127.45
127.36
126.92
126.84
126.56
126.51
126.22
126.09
124.79
Chemical Shift (ppm)
120.66
118.71
115.85
115.77
115.55
111.93
110.89
110.10
109.81
Chemical Shift (ppm)
42.79
42.33
Chemical Shift (ppm)

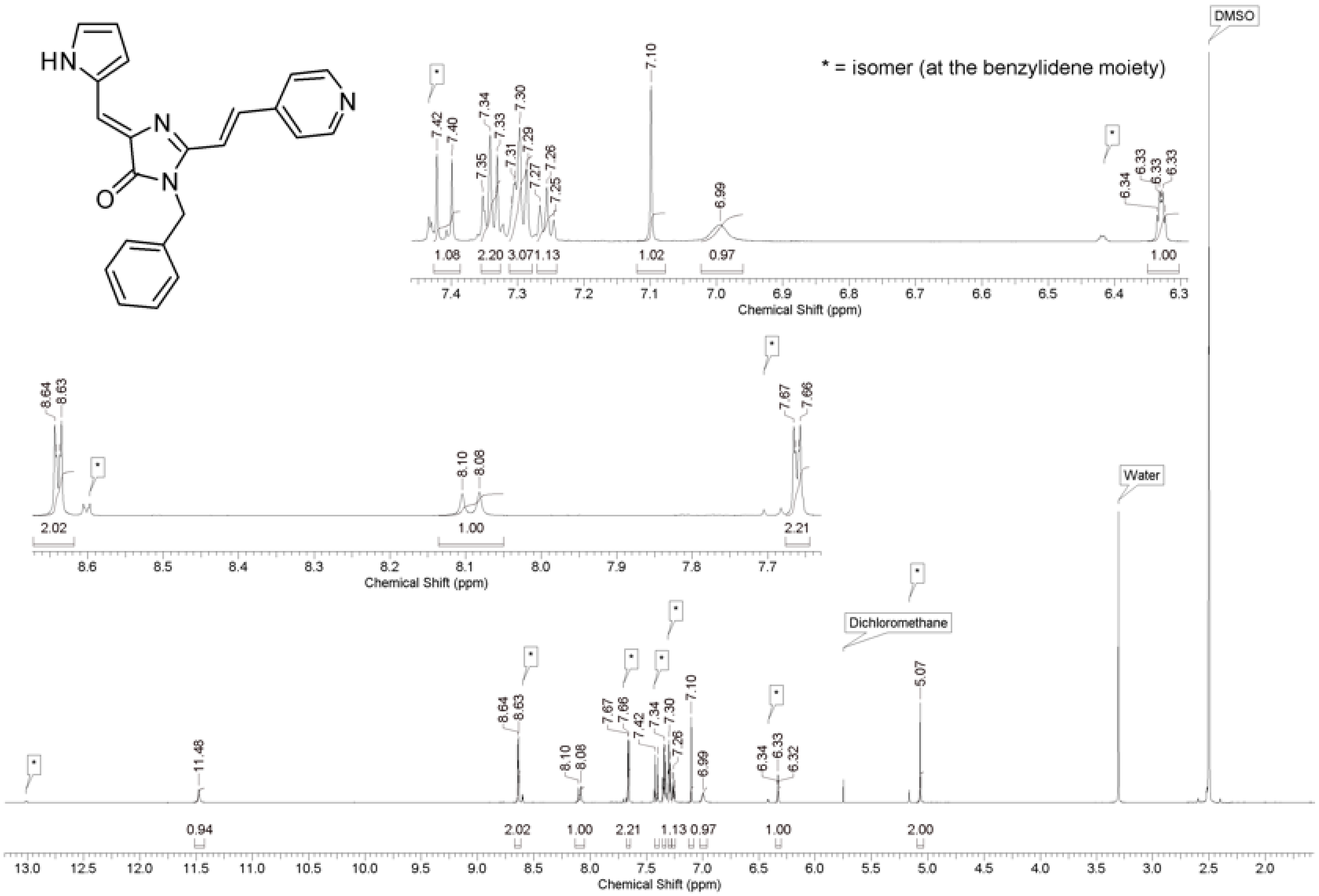
* = isomer (at the benzylidene moiety)
DMSO
Water
Dichloromethane
Chemical Shift (ppm)

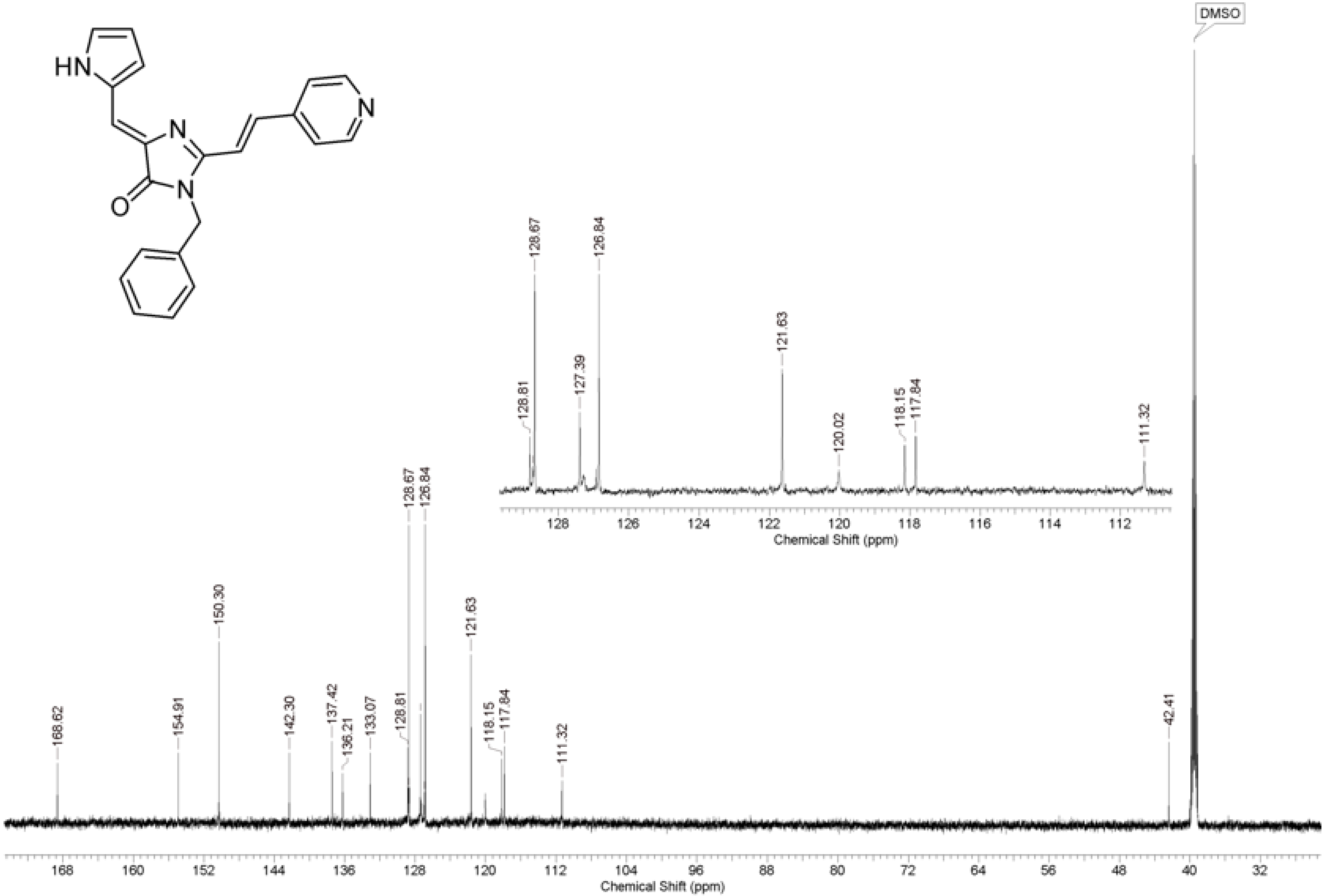
HN
N
N
N
O
DMSO
168.62
154.91
150.30
142.30
137.42
136.21
133.07
128.81
128.67
126.84
127.39
121.63
120.02
118.15
117.84
111.32
42.41
Chemical Shift (ppm)

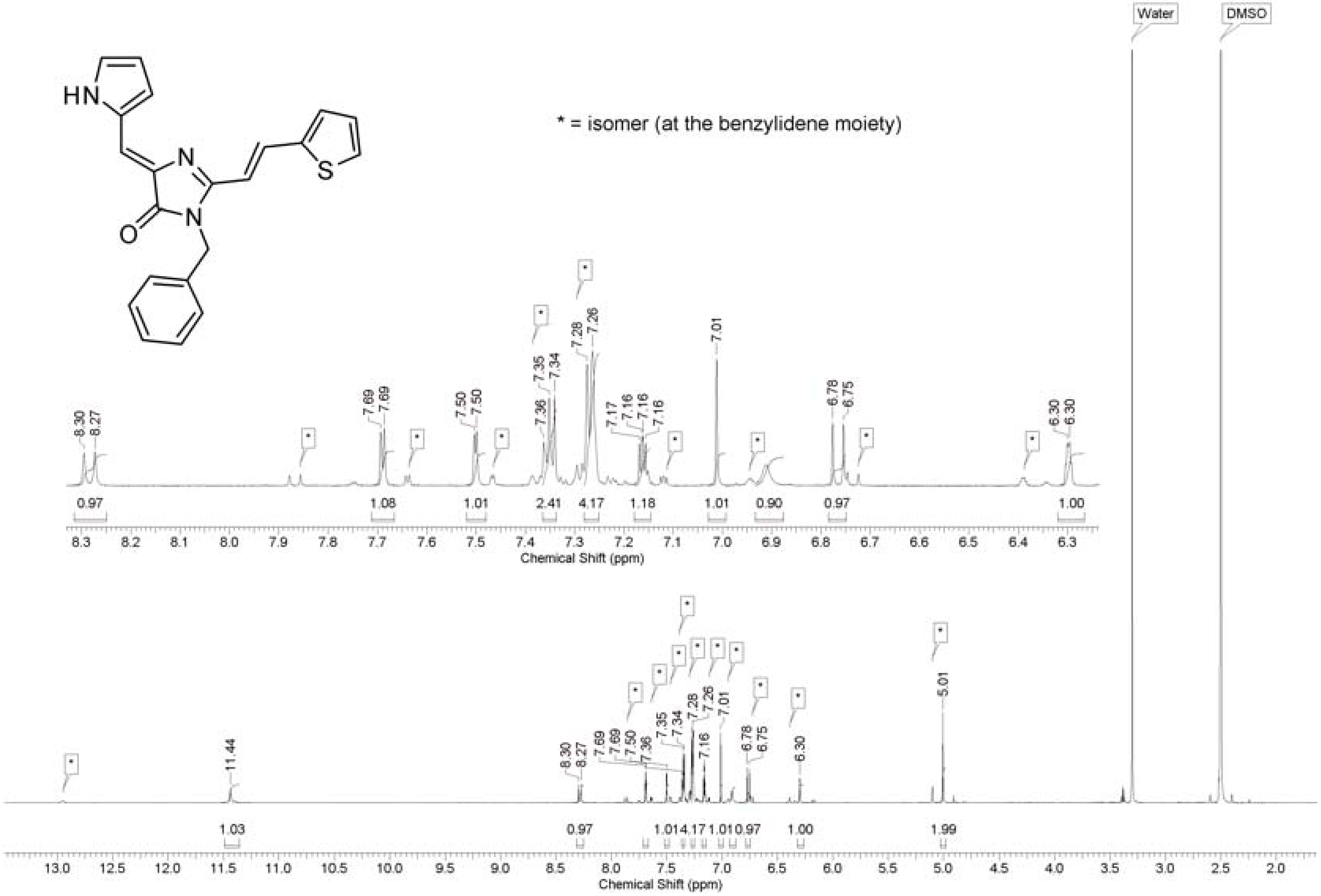

Water
DMSO
Chemical Shift (ppm)
Chemical Shift (ppm)

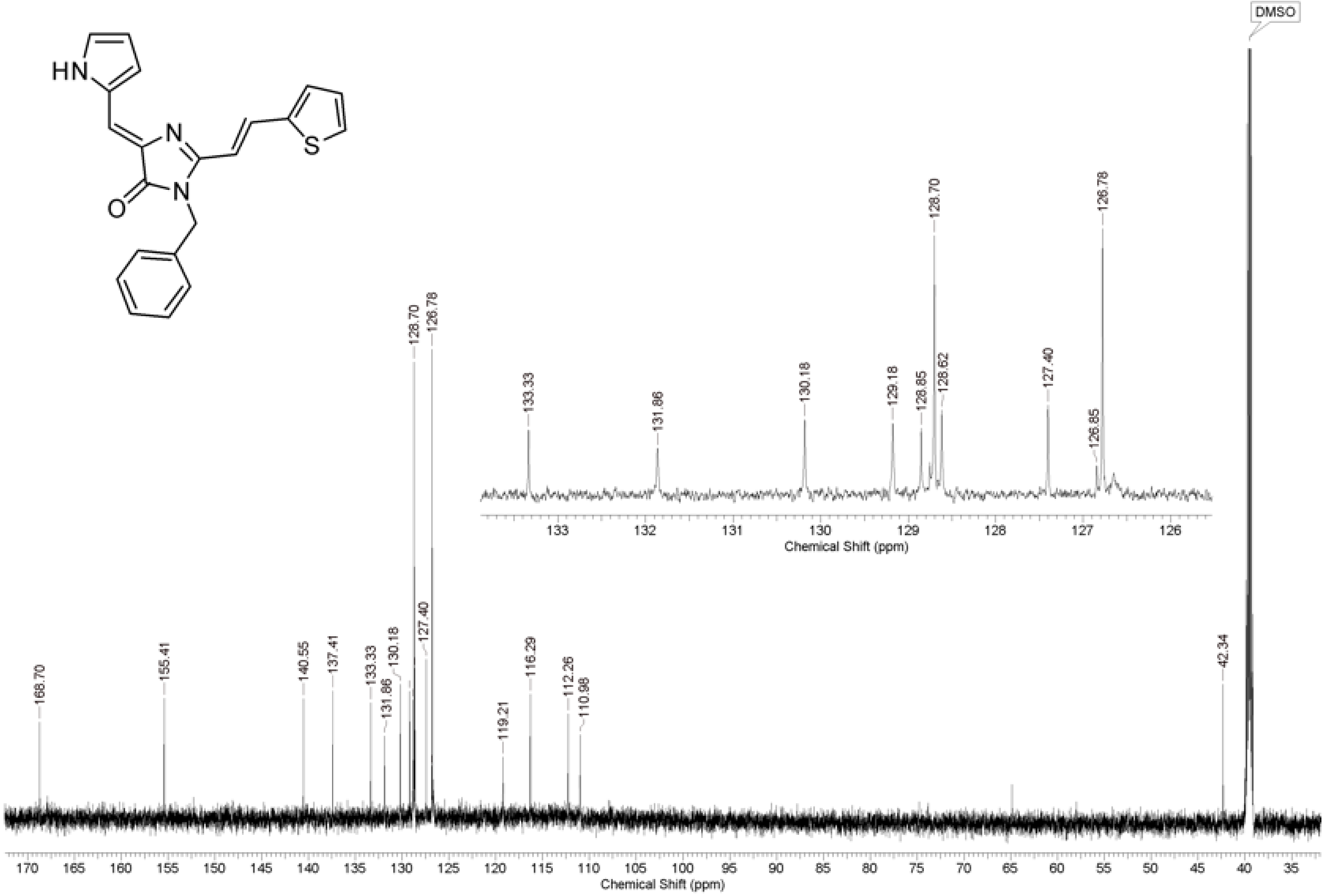
HN
N
S
O
N
DMSO
168.70
155.41
140.55
137.41
133.33
131.86
130.18
127.40
128.70
126.78
119.21
116.29
112.26
110.98
42.34
Chemical Shift (ppm)
129.18
128.85
128.62
126.85
Chemical Shift (ppm)

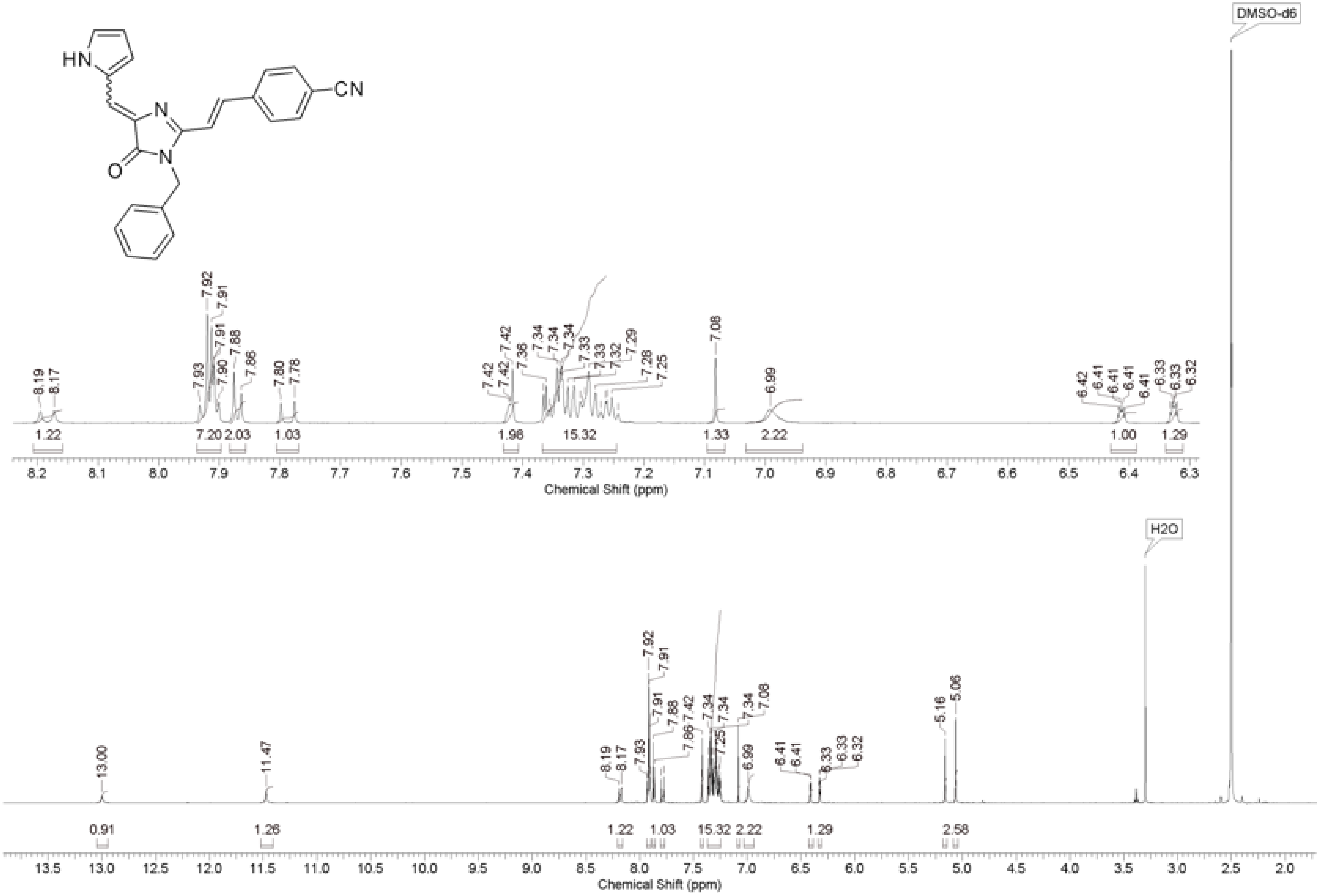
HN
N
N
O
CN
DMSO-d6
H2O
Chemical Shift (ppm)
Chemical Shift (ppm)

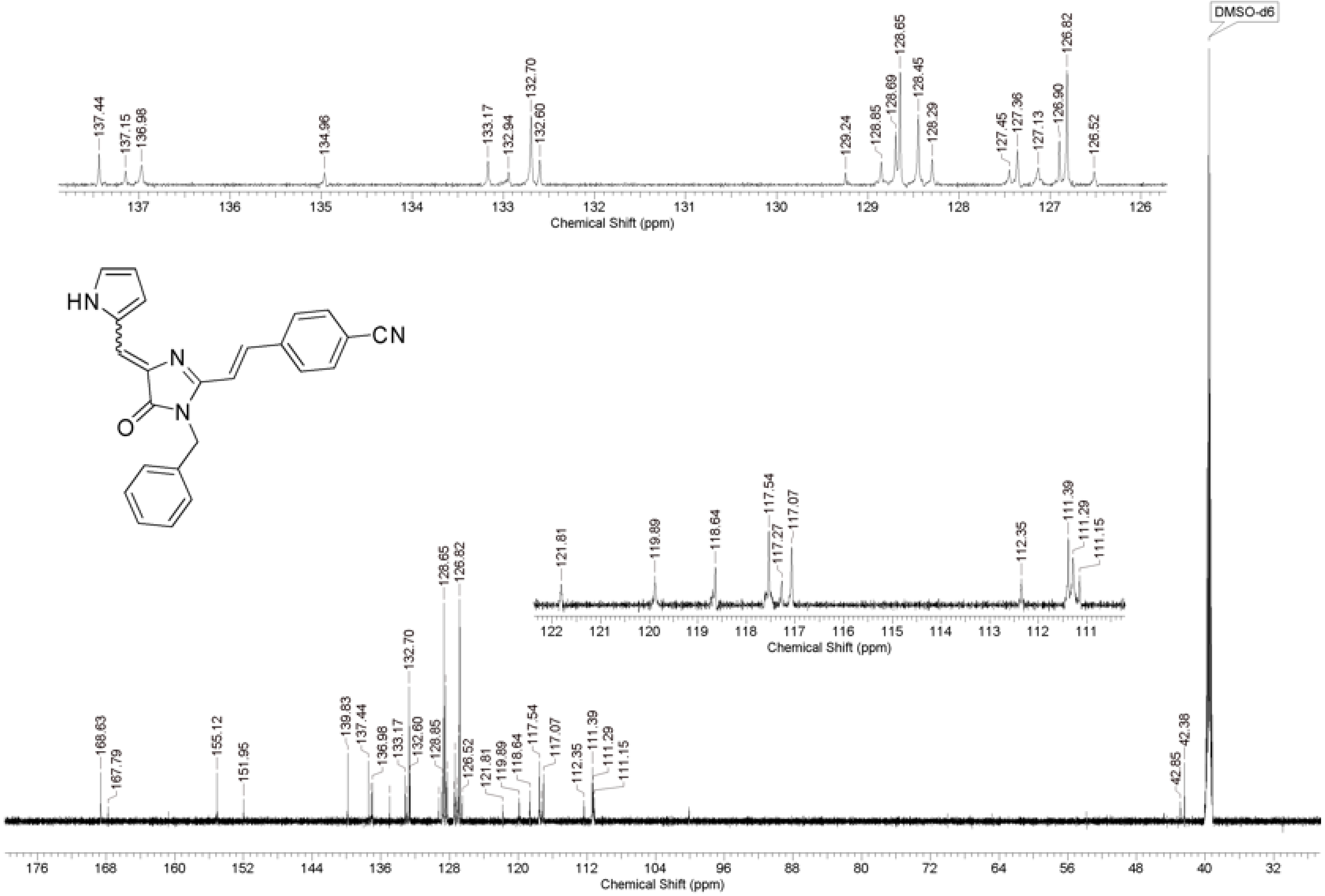
DMSO-d6
137.44
137.15
136.98
134.96
133.17
132.94
132.70
132.60
129.24
128.85
128.69
128.65
128.45
128.29
127.45
127.36
127.13
126.90
126.82
126.52
Chemical Shift (ppm)
HN
N
N
O
CN
121.81
119.89
118.64
117.54
117.27
117.07
112.35
111.39
111.29
111.15
Chemical Shift (ppm)
168.63
167.79
155.12
151.95
139.83
137.44
136.98
133.17
132.70
132.60
128.85
128.65
126.82
126.52
121.81
119.89
118.64
117.54
117.07
112.35
111.39
111.29
111.15
42.85
42.38
Chemical Shift (ppm)

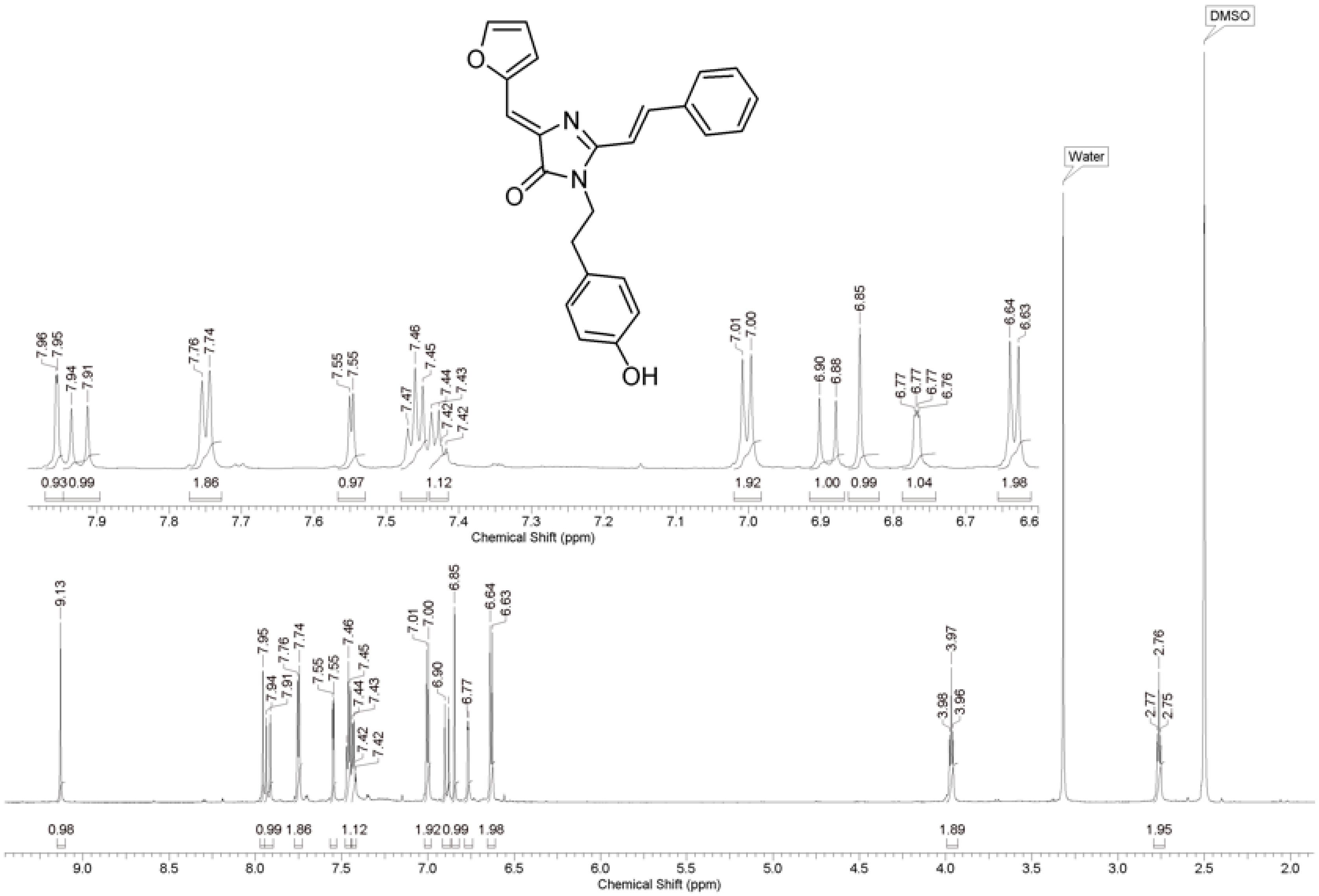

OH
DMSO
Water
Chemical Shift (ppm)
Chemical Shift (ppm)

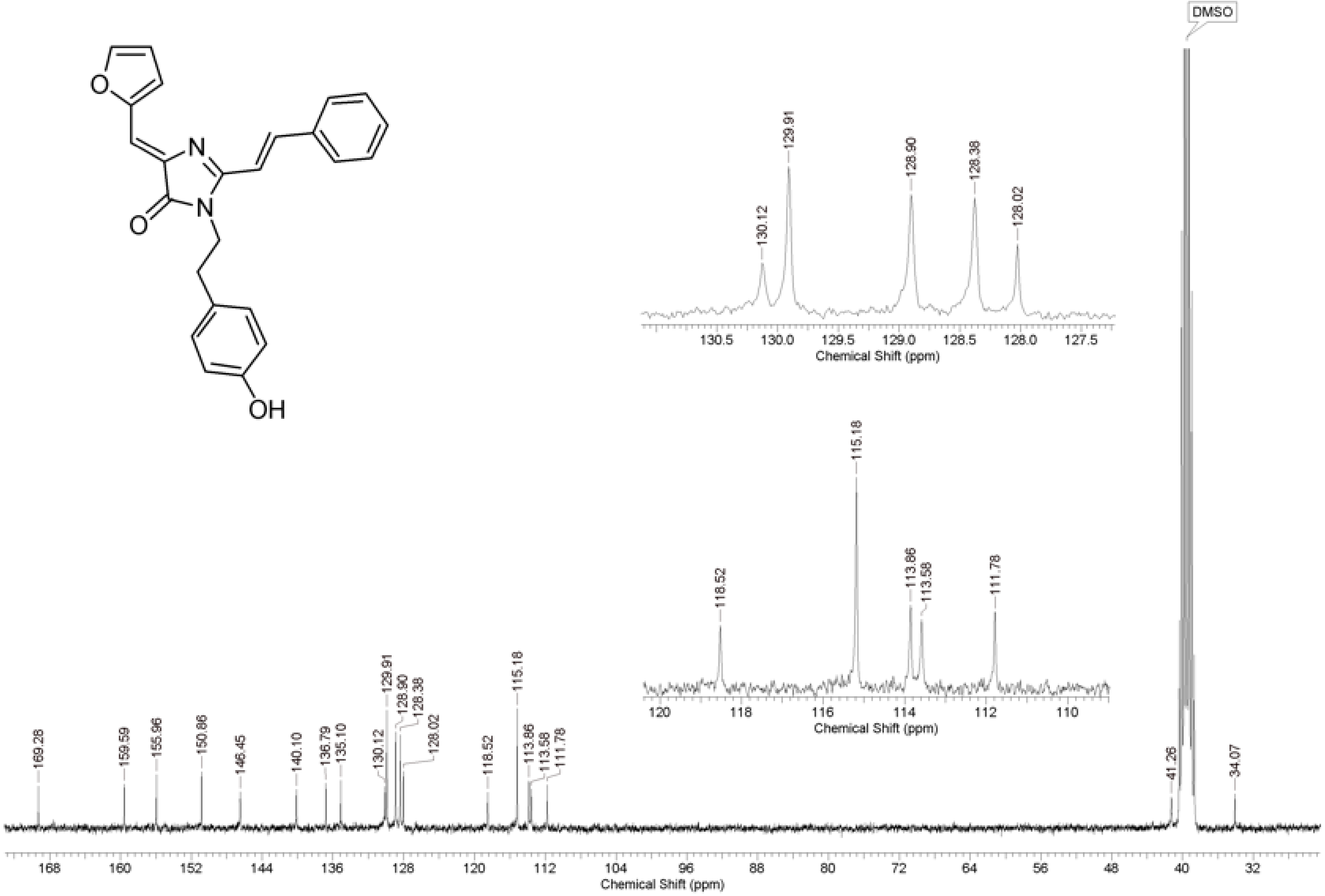

OH
O
N
N
O
DMSO
169.28
159.59
155.96
150.86
146.45
140.10
136.79
135.10
130.12
129.91
128.90
128.38
128.02
118.52
115.18
113.86
113.58
111.78
41.26
34.07
Chemical Shift (ppm)

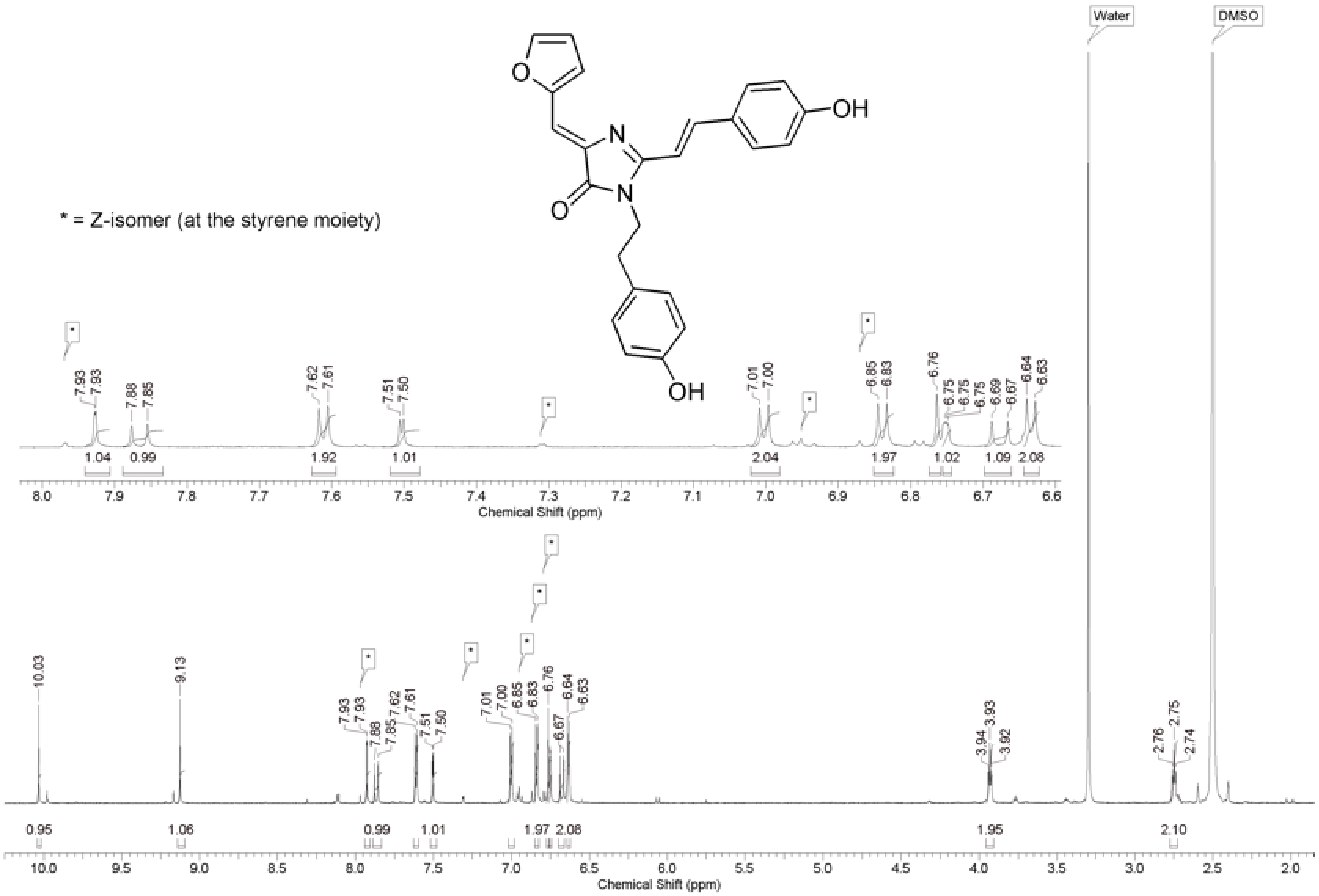

* = Z-isomer (at the styrene moiety)
OH
N
O
N
O
OH
Water
DMSO
Chemical Shift (ppm)
Chemical Shift (ppm)

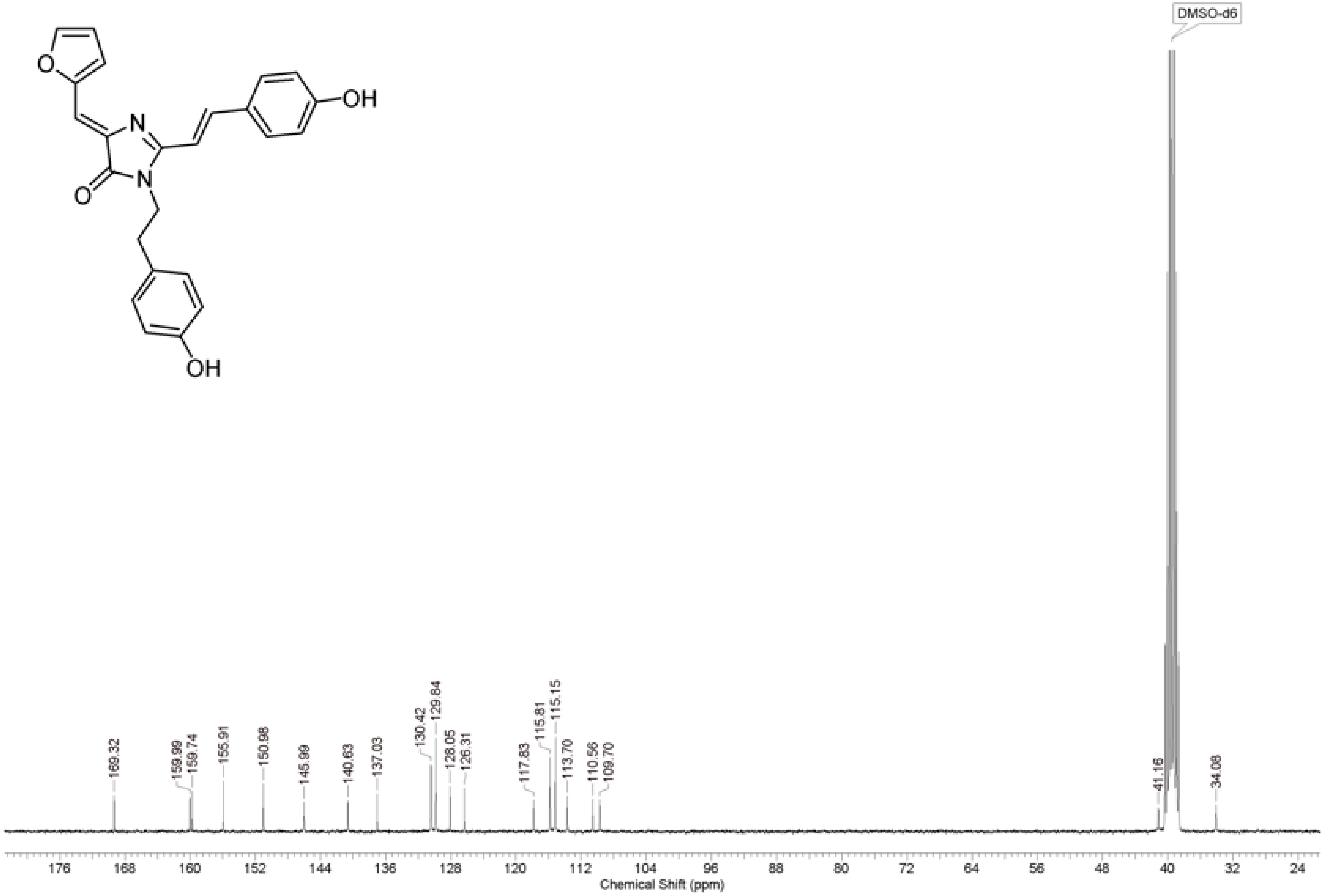
OH
N
N
O
O
OH
DMSO-d6
169.32
159.99
159.74
155.91
150.98
145.99
140.63
137.03
130.42
129.84
128.05
126.31
117.83
115.81
115.15
113.70
110.56
109.70
41.16
34.08
176 168 160 152 144 136 128 120 112 104 96 88 80 72 64 56 48 40 32 24
Chemical Shift (ppm)

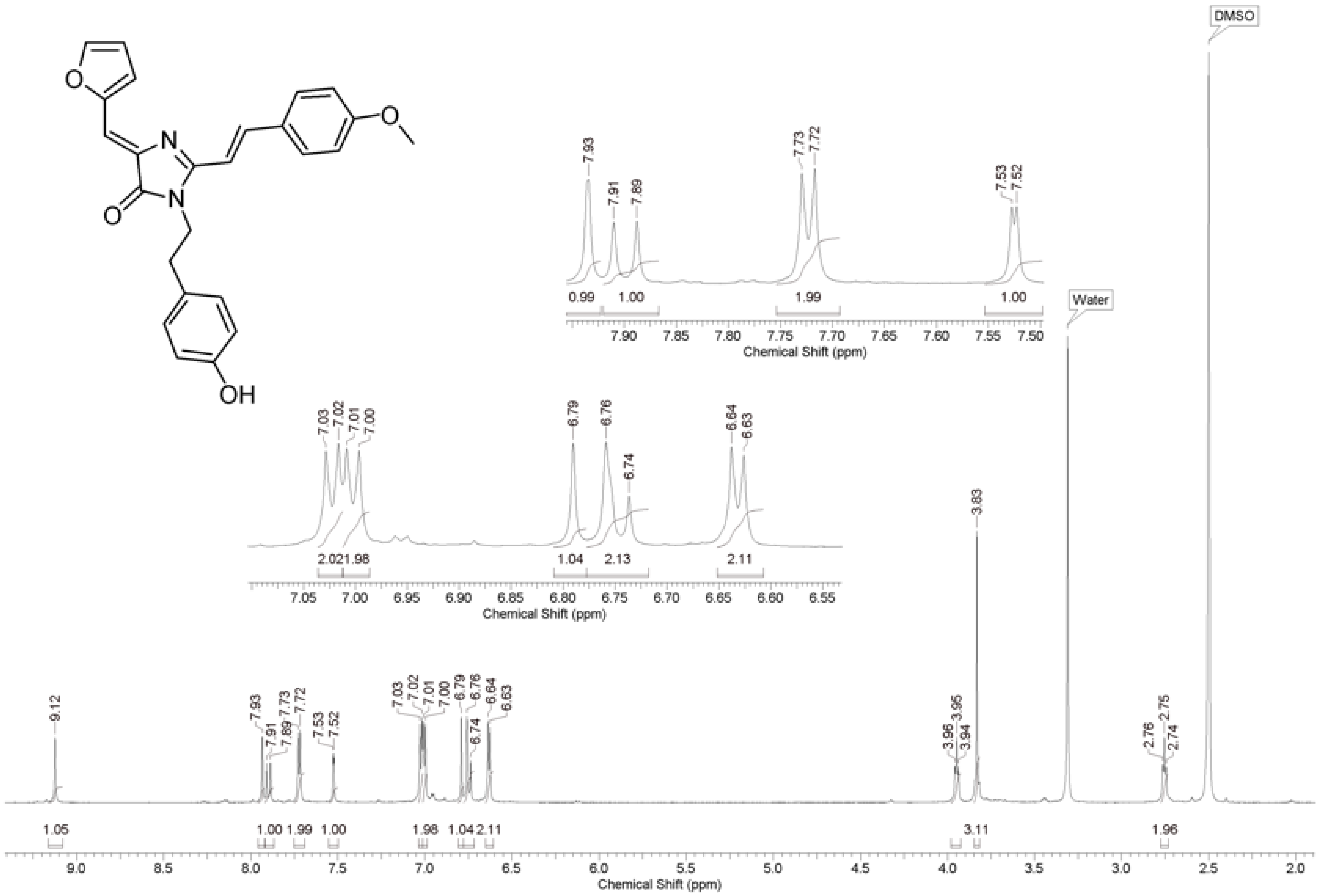

DMSO
Water
OH
N
N
O
O
O
Chemical Shift (ppm)

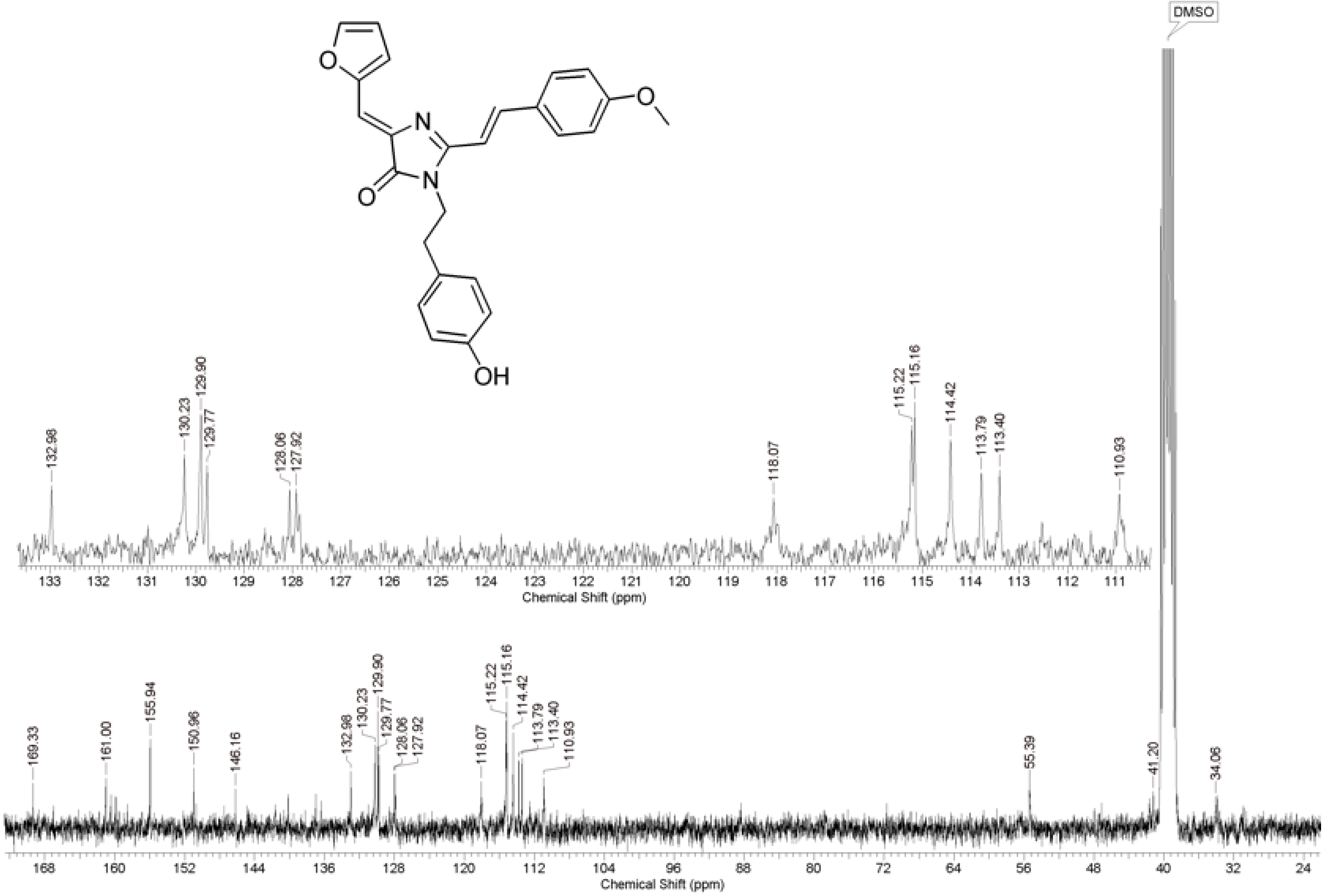

O
N
N
O
O
OH
DMSO
132.98
130.23
129.90
129.77
128.06
127.92
118.07
115.22
115.16
114.42
113.79
113.40
110.93
Chemical Shift (ppm)
169.33
161.00
155.94
150.96
146.16
55.39
41.20
34.06
Chemical Shift (ppm)

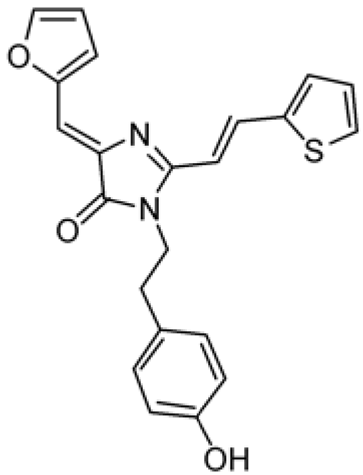

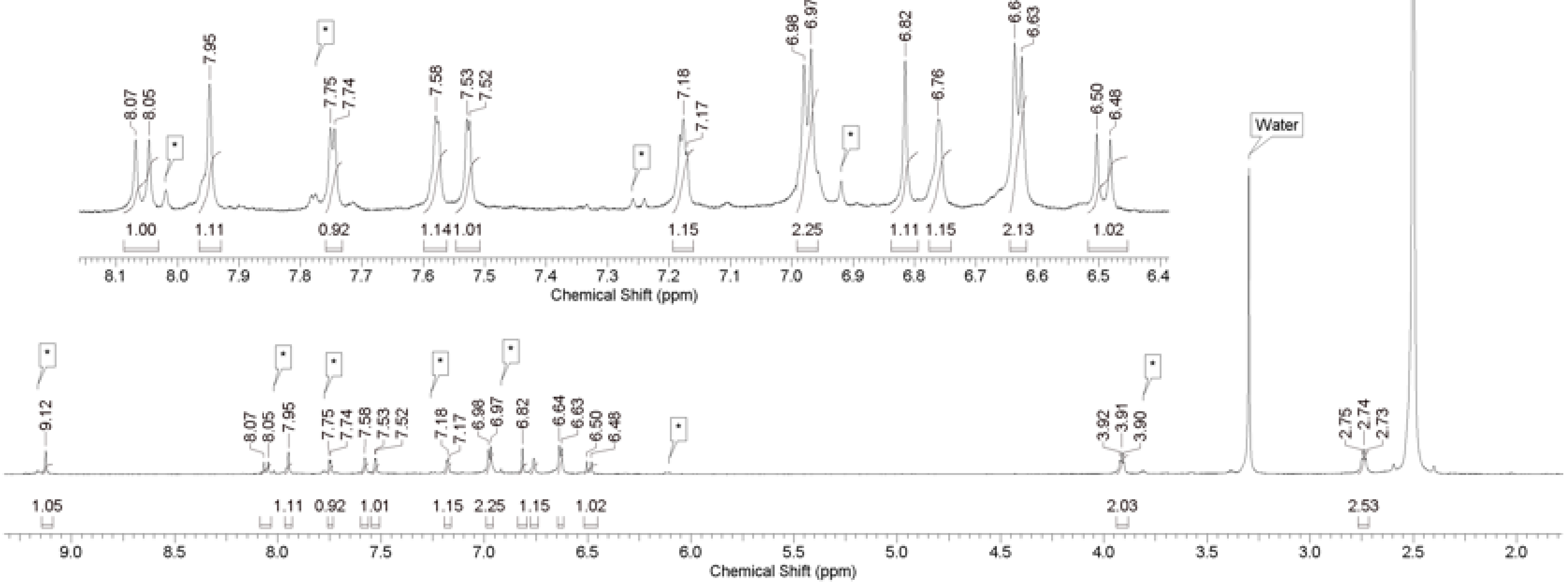
* = Z-isomer (at the styrene moiety)
DMSO
Water
Chemical Shift (ppm)
Chemical Shift (ppm)

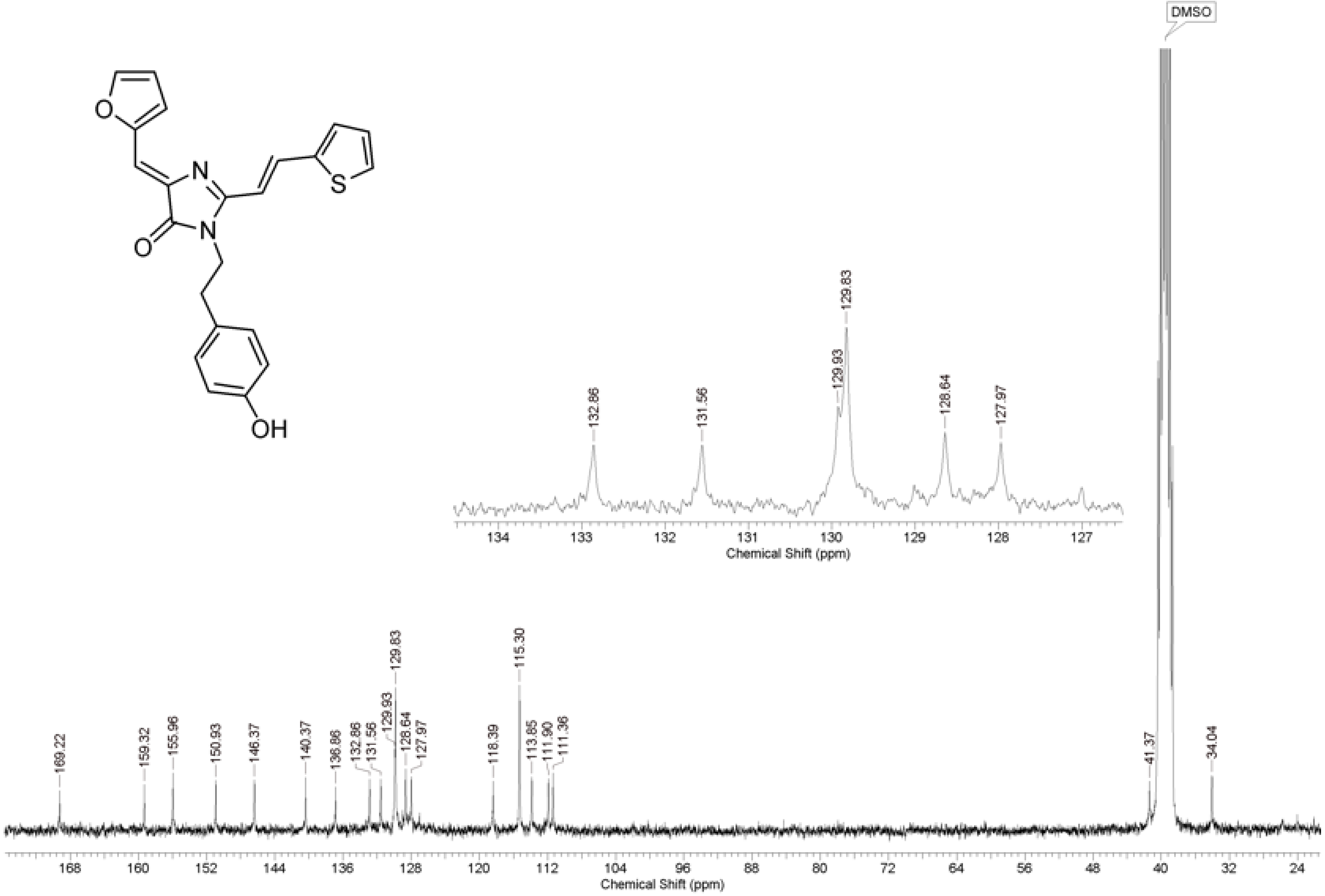
O
N
S
O
N
OH
DMSO
132.86
131.56
129.93
129.83
128.64
127.97
134
133
132
131
130
129
128
127
Chemical Shift (ppm)
169.22
159.32
155.96
150.93
146.37
140.37
136.86
132.86
131.56
129.93
129.83
128.64
127.97
118.39
115.30
113.85
111.90
111.36
41.37
34.04
168
160
152
144
136
128
120
112
104
96
88
80
72
64
56
48
40
32
24
Chemical Shift (ppm)

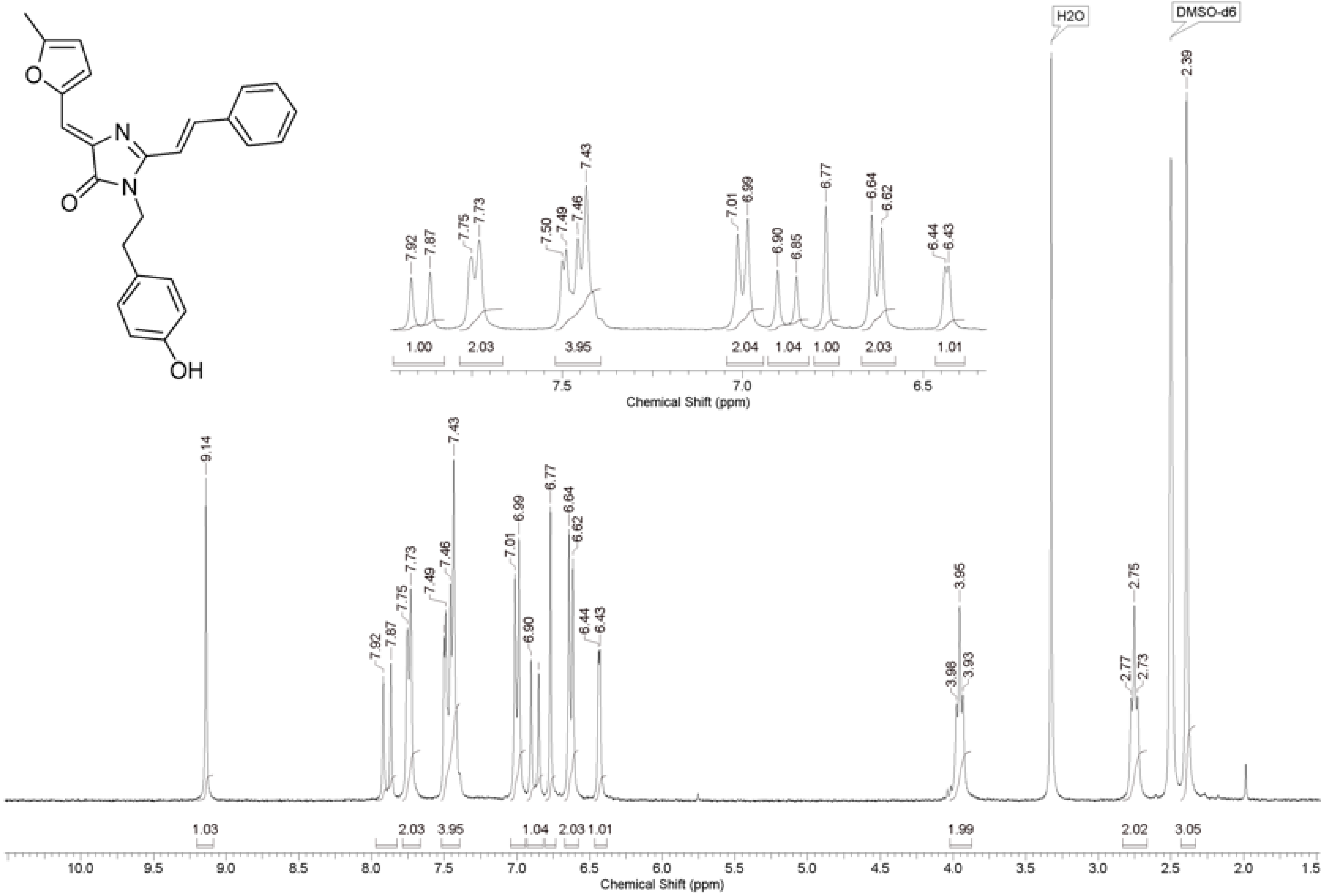

H2O
DMSO-d6
Chemical Shift (ppm)
Chemical Shift (ppm)

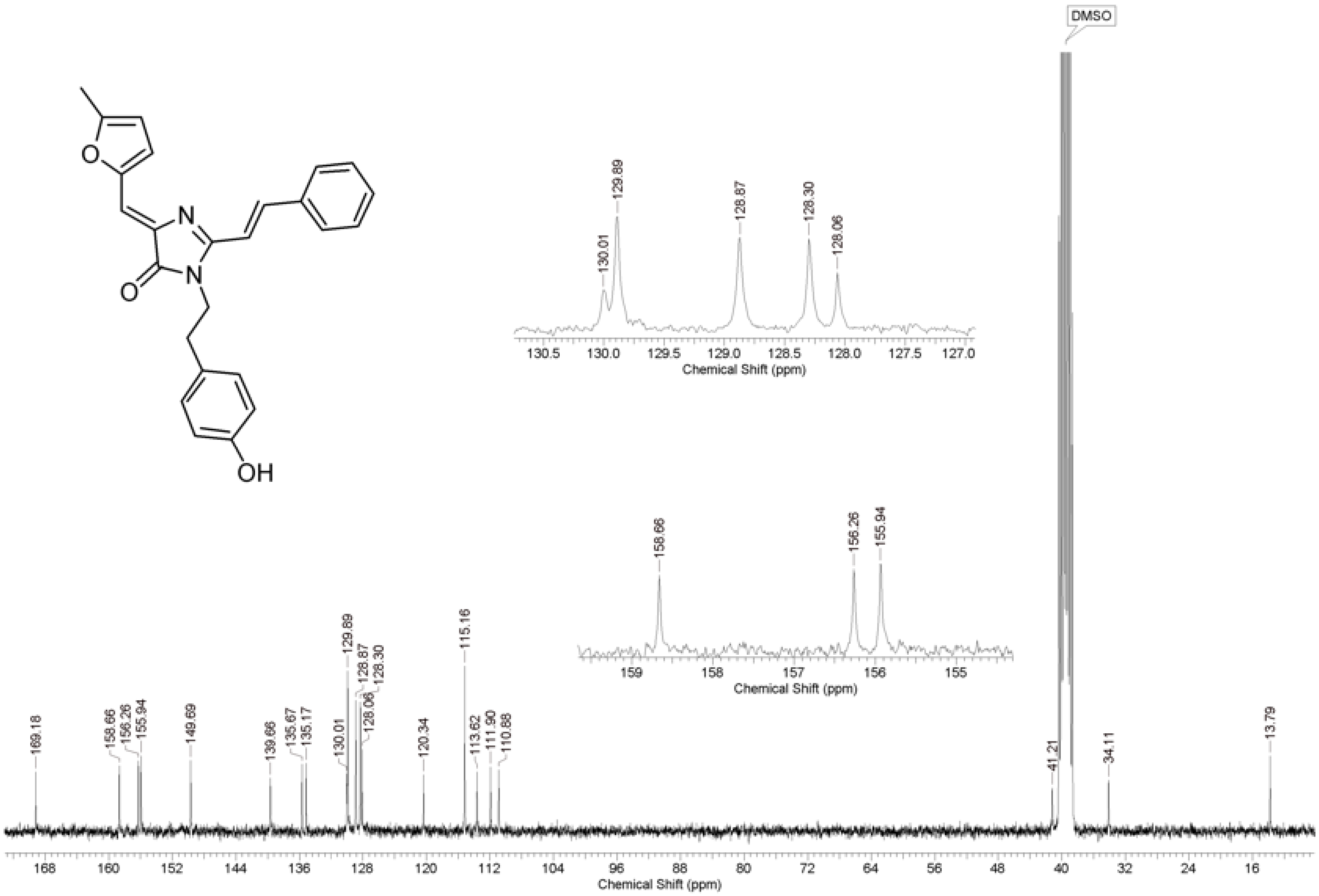
DMSO
169.18
158.66
156.26
155.94
149.69
139.66
135.67
135.17
130.01
129.89
128.87
128.06
128.30
120.34
115.16
113.62
111.90
110.88
41.21
34.11
13.79
Chemical Shift (ppm)
OH
O
N

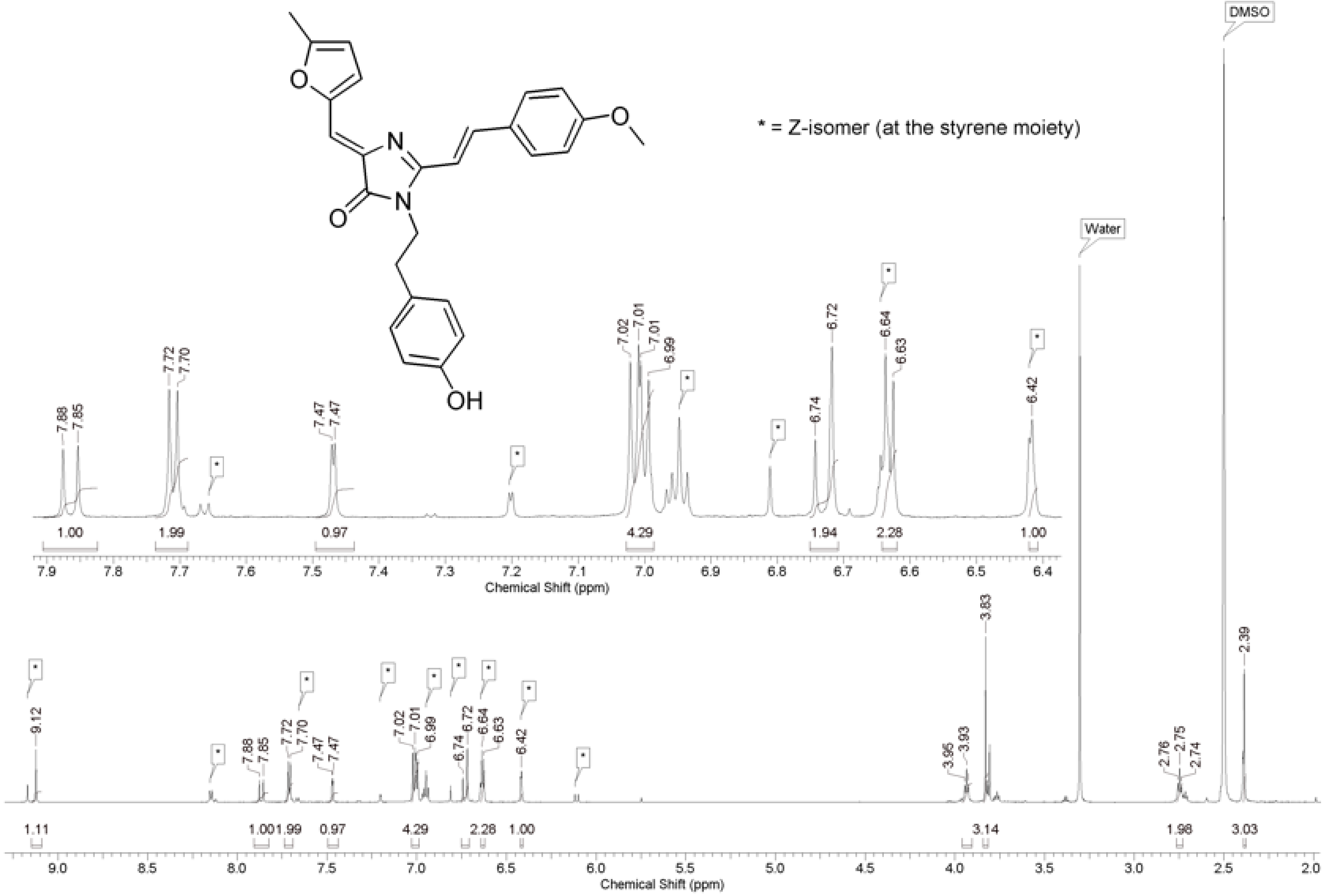
* = Z-isomer (at the styrene moiety)
DMSO
Water
OH
7.88
7.85
7.72
7.70
7.47
7.47
7.02
7.01
7.01
6.99
6.74
6.72
6.64
6.63
6.42
9.12
3.95
3.93
3.83
2.76
2.75
2.74
2.39
1.00
1.99
0.97
4.29
1.94
2.28
1.00
1.11
1.00
1.99
0.97
4.29
2.28
1.00
3.14
1.98
3.03
Chemical Shift (ppm)
Chemical Shift (ppm)

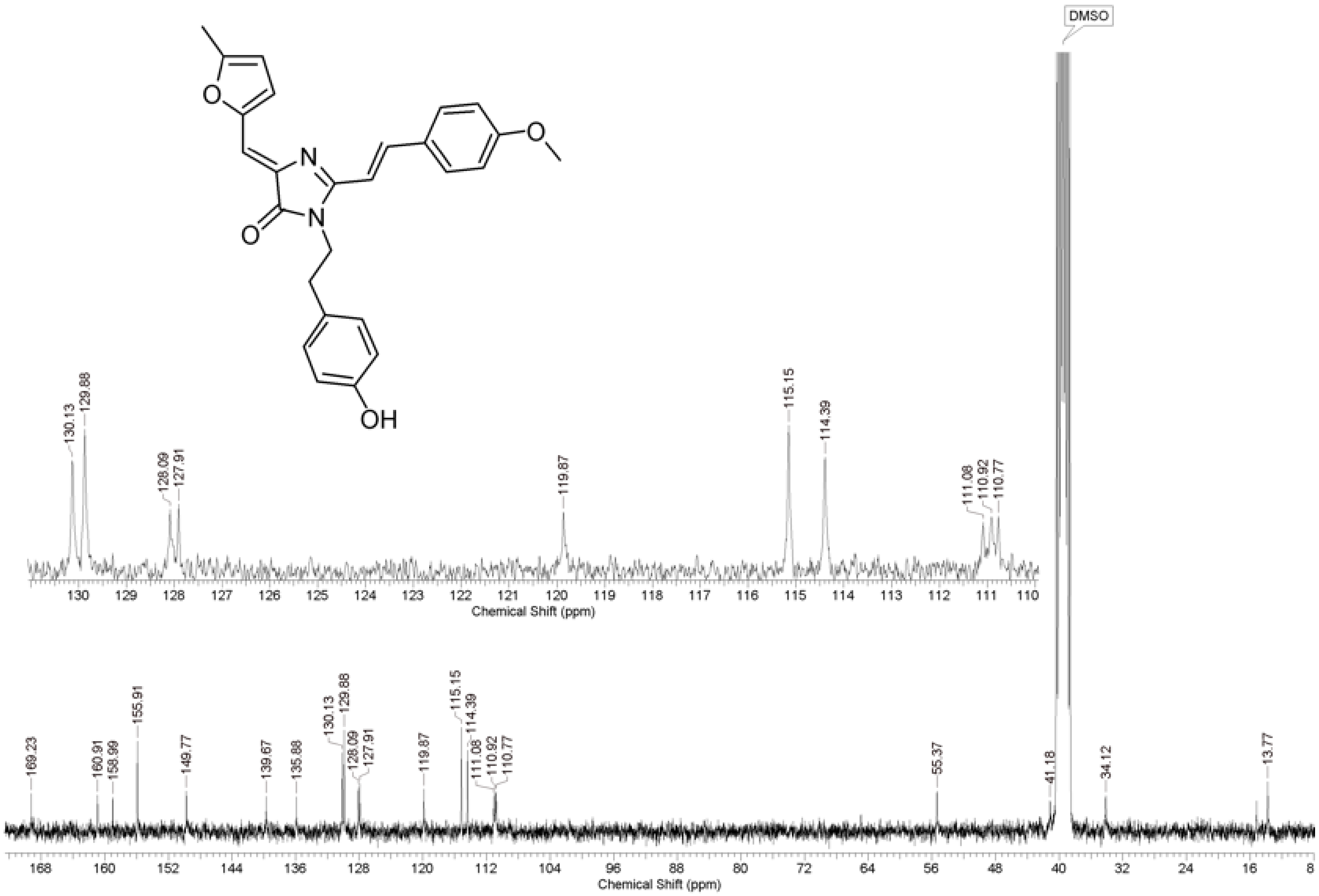
DMSO
130.13
129.88
128.09
127.91
119.87
115.15
114.39
111.08
110.92
110.77
Chemical Shift (ppm)
169.23
160.91
158.99
155.91
149.77
139.67
135.88
130.13
129.88
128.09
127.91
119.87
115.15
114.39
111.08
110.92
110.77
55.37
41.18
34.12
13.77
Chemical Shift (ppm)

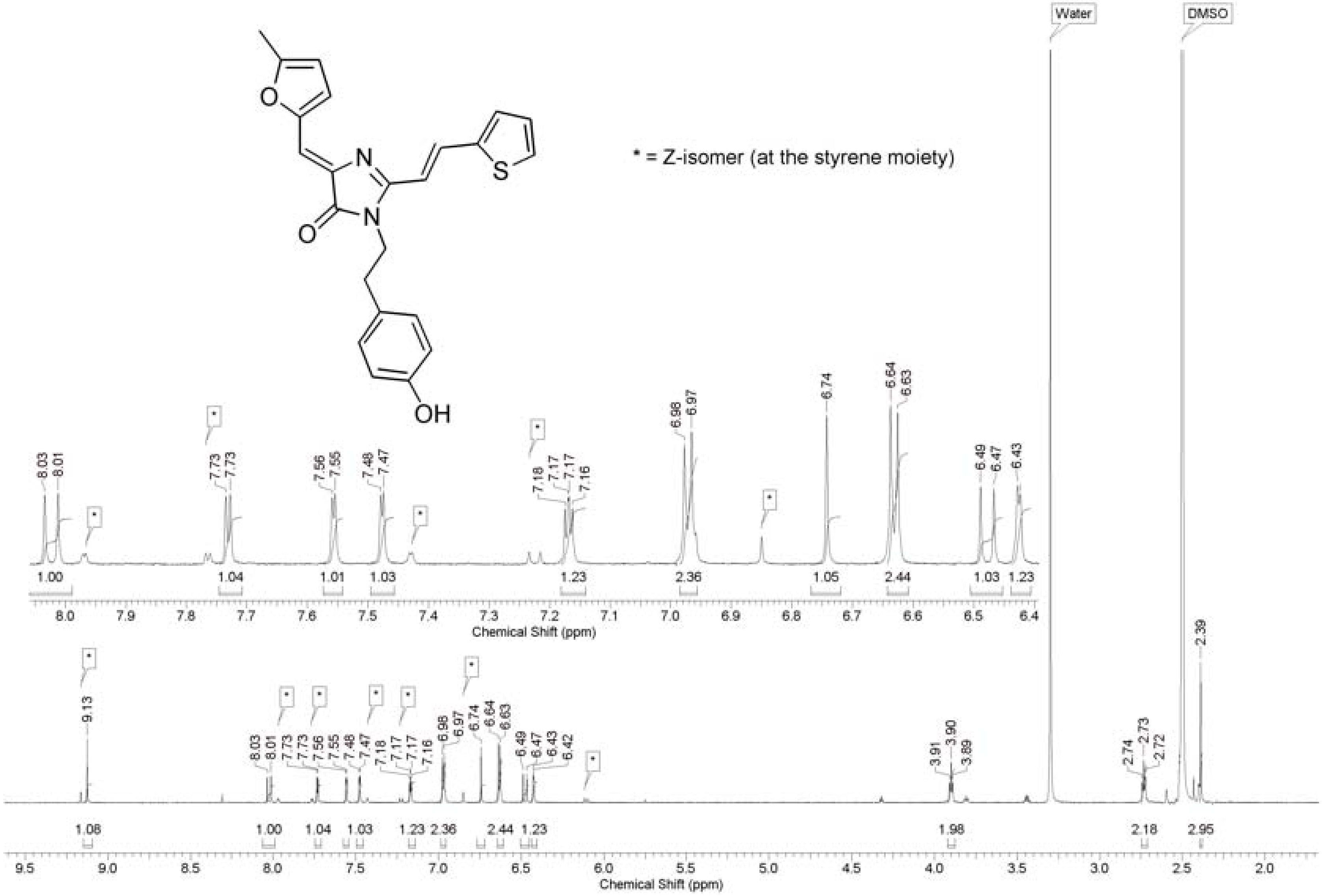
* = Z-isomer (at the styrene moiety)
Water
DMSO
Chemical Shift (ppm)
Chemical Shift (ppm)

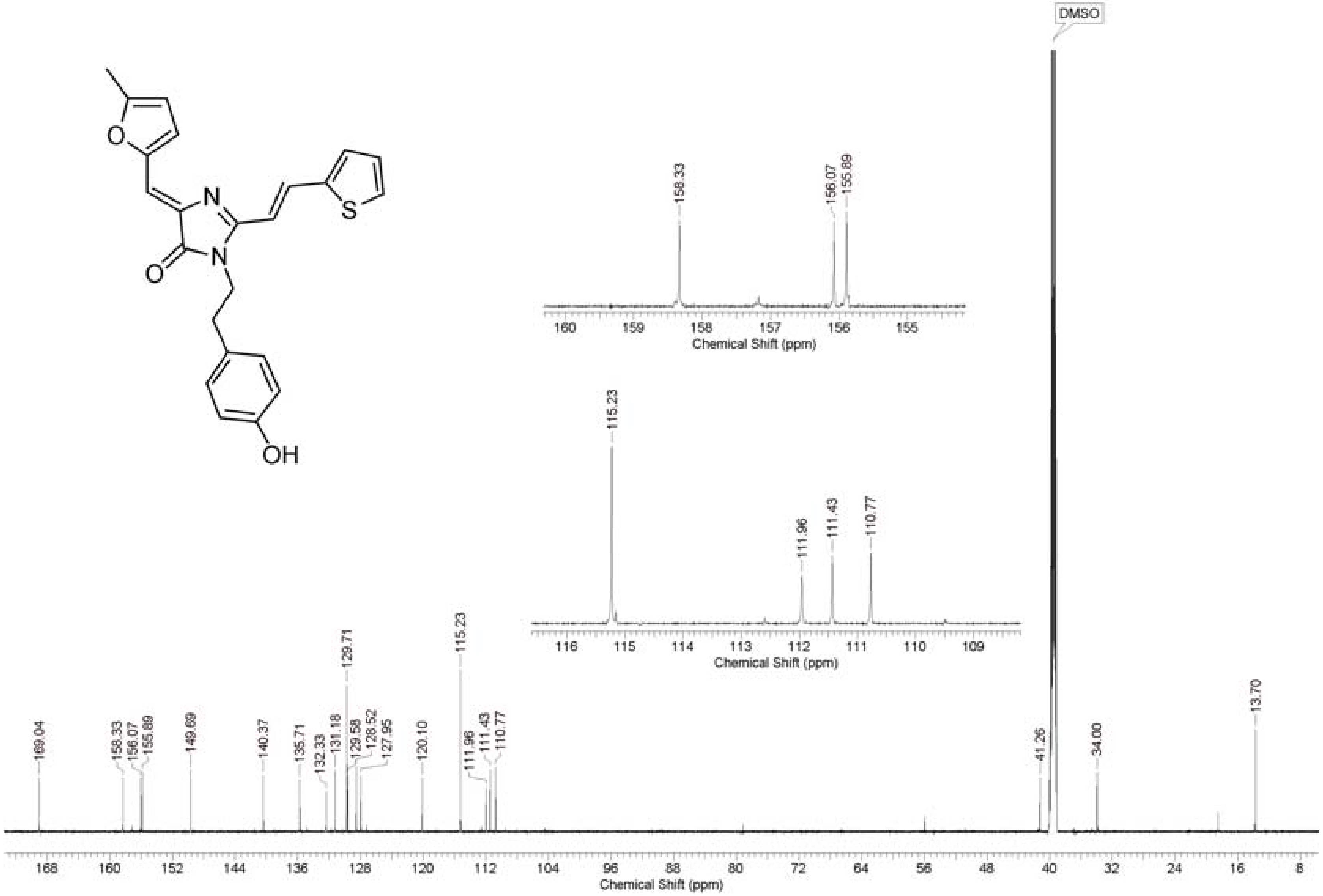
DMSO
158.33
156.07
155.89
160
159
158
157
156
155
Chemical Shift (ppm)
115.23
111.96
111.43
110.77
116
115
114
113
112
111
110
109
Chemical Shift (ppm)
169.04
158.33
156.07
155.89
149.69
140.37
135.71
132.33
131.18
129.71
129.58
128.52
127.95
120.10
115.23
111.96
111.43
110.77
41.26
34.00
13.70
168
160
152
144
136
128
120
112
104
96
88
80
72
64
56
48
40
32
24
16
8
Chemical Shift (ppm)

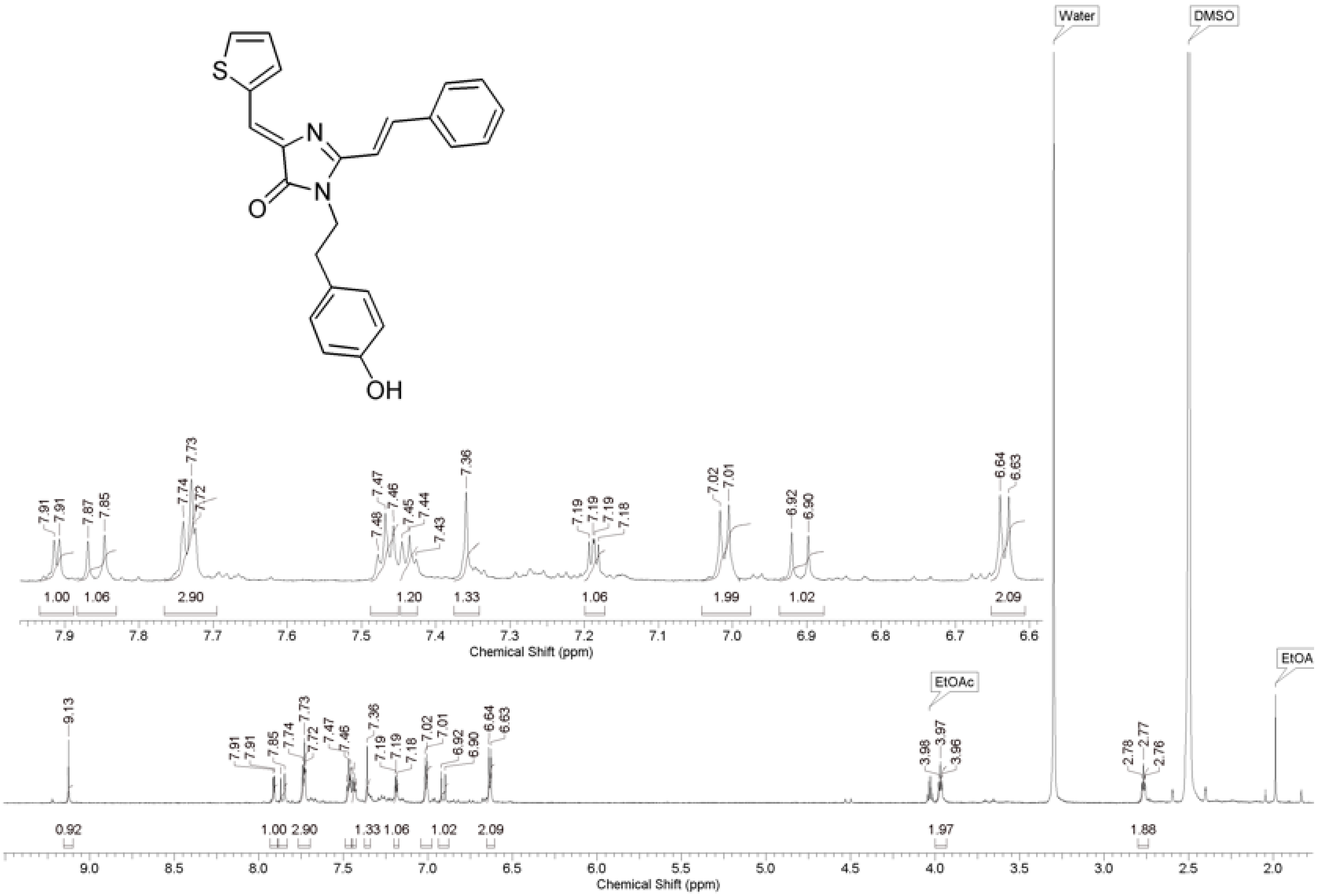
S
N
N
O
OH
Water
DMSO
EtOAc
EtOA
Chemical Shift (ppm)
Chemical Shift (ppm)

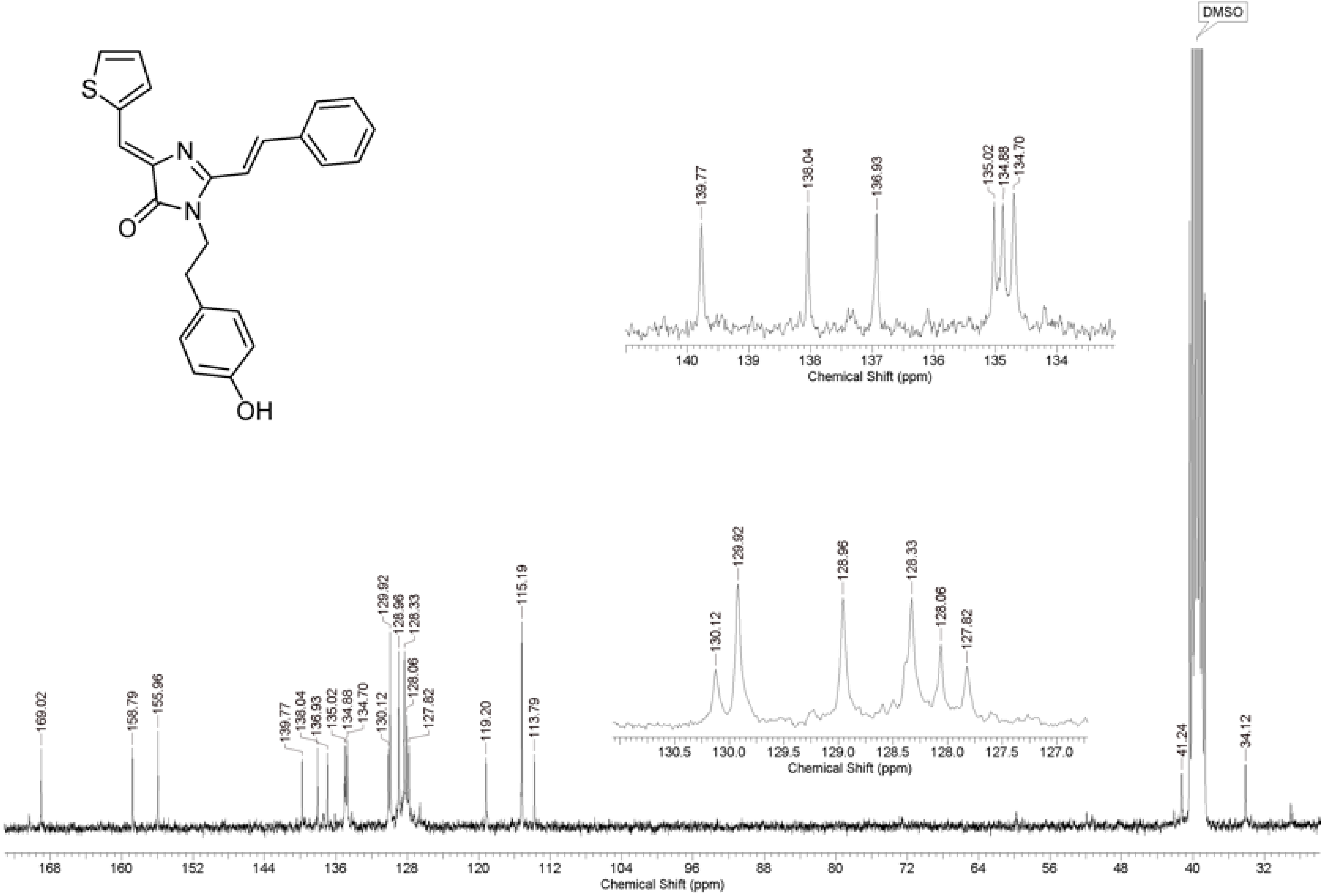
S
N
N
O
OH
DMSO
169.02
158.79
155.96
139.77
138.04
136.93
135.02
134.88
134.70
130.12
129.92
128.96
128.33
128.06
127.82
119.20
115.19
113.79
41.24
34.12
168 160 152 144 136 128 120 112 104 96 88 80 72 64 56 48 40 32
Chemical Shift (ppm)
140 139 138 137 136 135 134
Chemical Shift (ppm)
130.5 130.0 129.5 129.0 128.5 128.0 127.5 127.0
Chemical Shift (ppm)

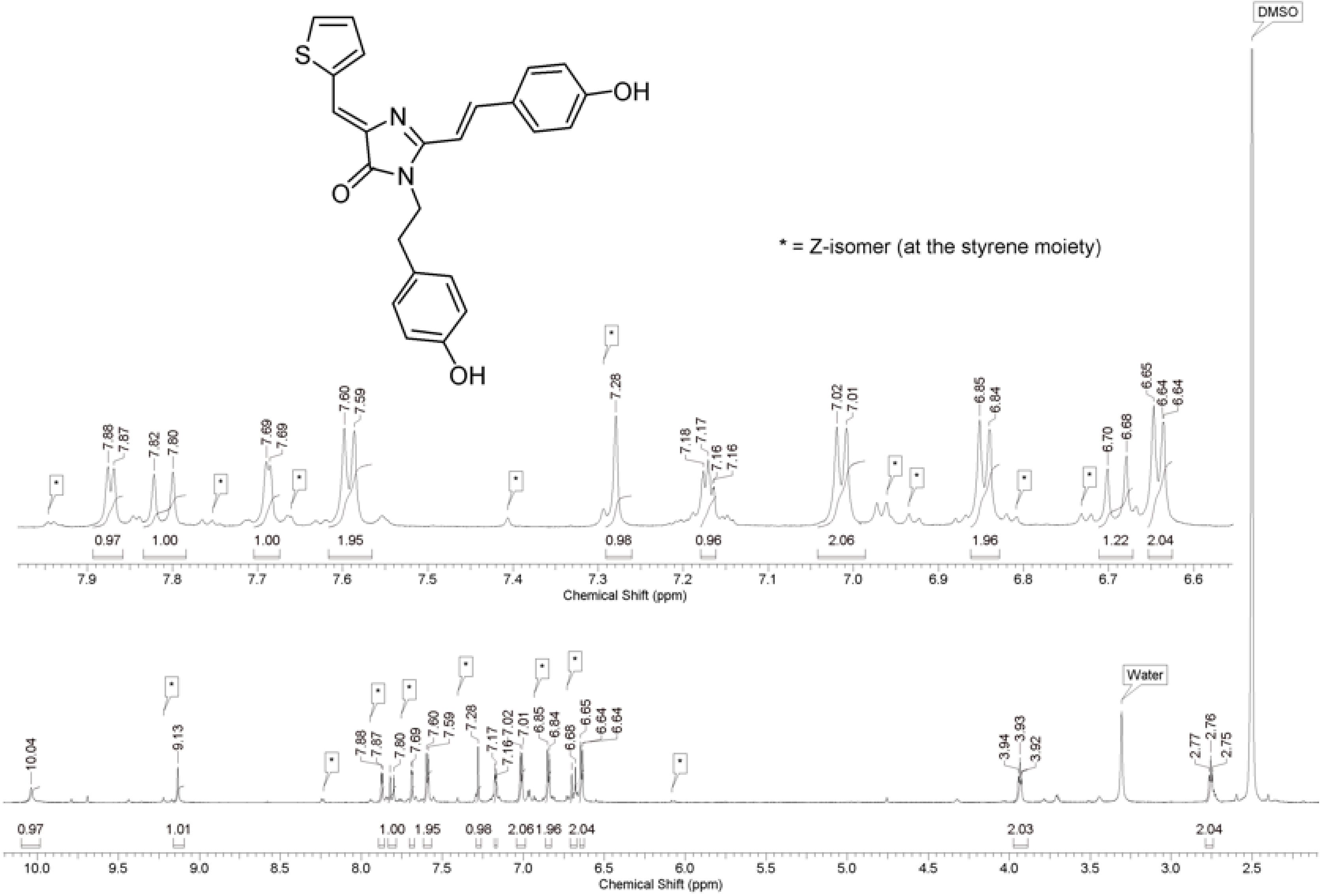
S
N
N
O
OH
OH
* = Z-isomer (at the styrene moiety)
DMSO
Water
Chemical Shift (ppm)
Chemical Shift (ppm)

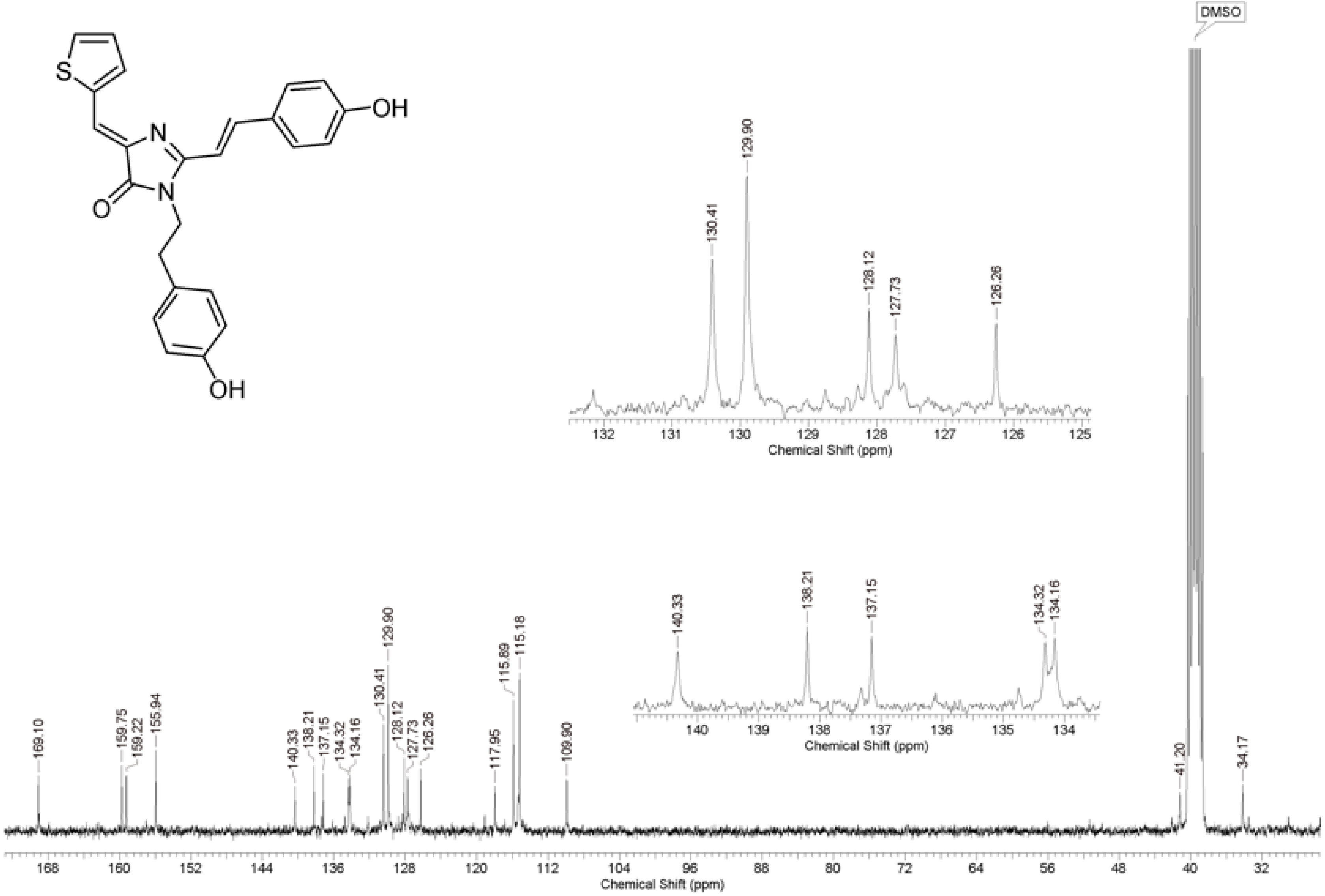
DMSO
169.10
159.75
159.22
155.94
140.33
138.21
137.15
134.32
134.16
130.41
129.90
128.12
127.73
126.26
117.95
115.89
115.18
109.90
41.20
34.17
168
160
152
144
136
128
120
112
104
96
88
80
72
64
56
48
40
32
Chemical Shift (ppm)
130.41
129.90
128.12
127.73
126.26
132
131
130
129
128
127
126
125
Chemical Shift (ppm)
140.33
138.21
137.15
134.32
134.16
140
139
138
137
136
135
134
Chemical Shift (ppm)

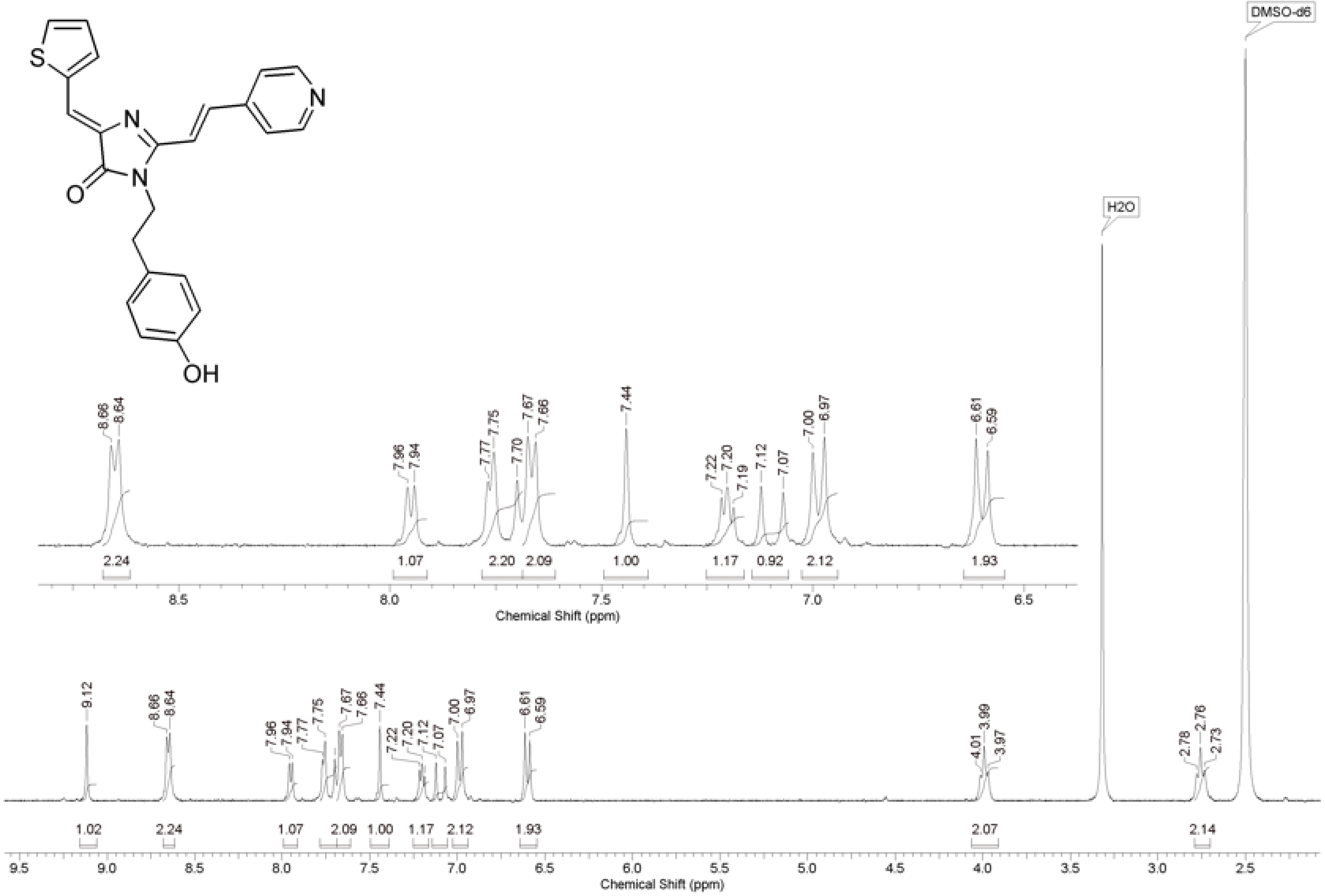
DMSO-d6
H2O
S
N
N
N
O
OH
8.66
8.64
7.96
7.94
7.77
7.75
7.70
7.67
7.66
7.44
7.22
7.20
7.19
7.12
7.07
7.00
6.97
6.61
6.59
2.24
1.07
2.20
2.09
1.00
1.17
0.92
2.12
1.93
Chemical Shift (ppm)
9.12
4.01
3.99
3.97
2.78
2.76
2.73
1.02
2.07
2.14
Chemical Shift (ppm)

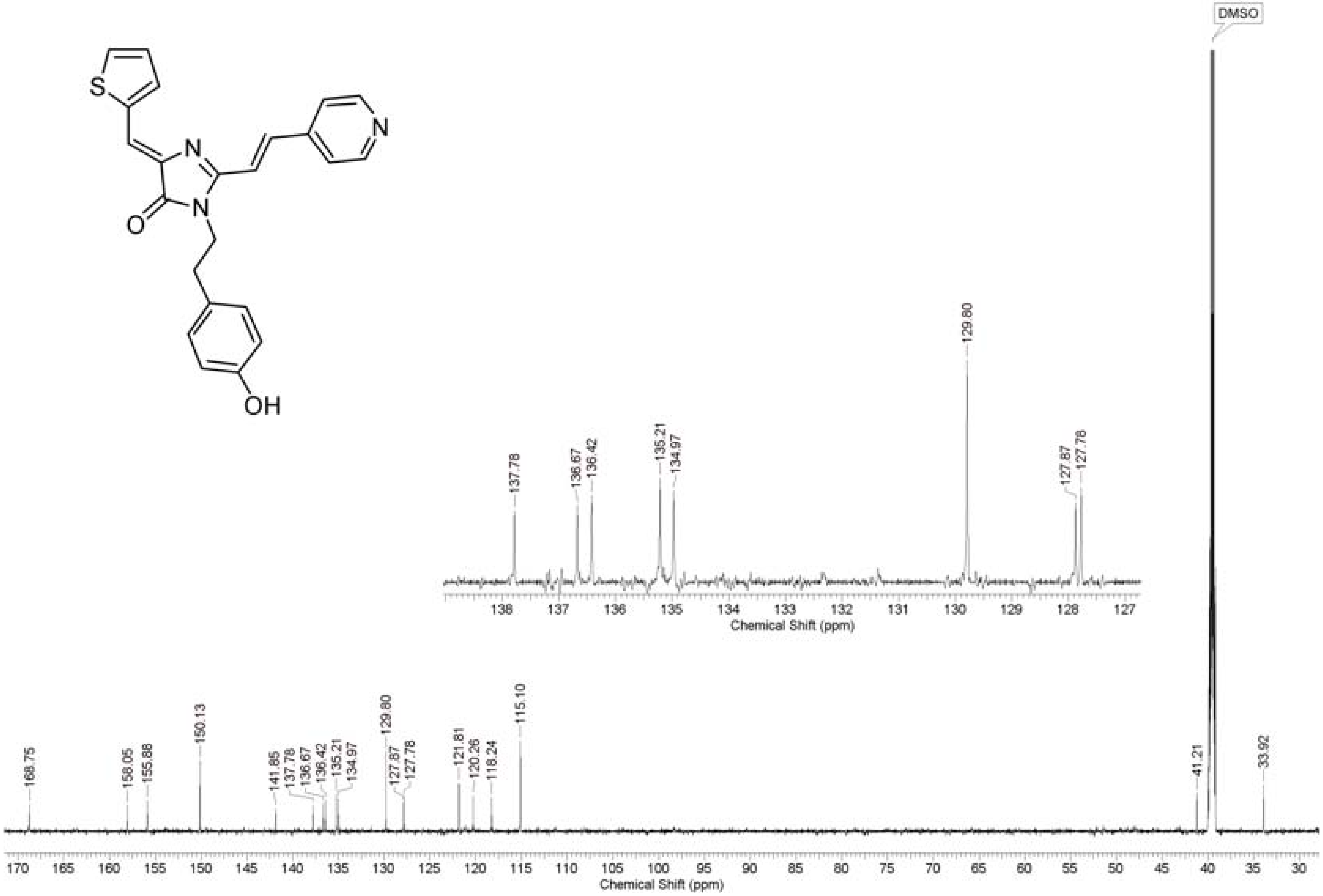
DMSO
129.80
137.78
136.67
136.42
135.21
134.97
127.87
127.78
Chemical Shift (ppm)
168.75
158.05
155.88
150.13
141.85
137.78
136.67
136.42
135.21
134.97
129.80
127.87
127.78
121.81
120.26
118.24
115.10
41.21
33.92
Chemical Shift (ppm)

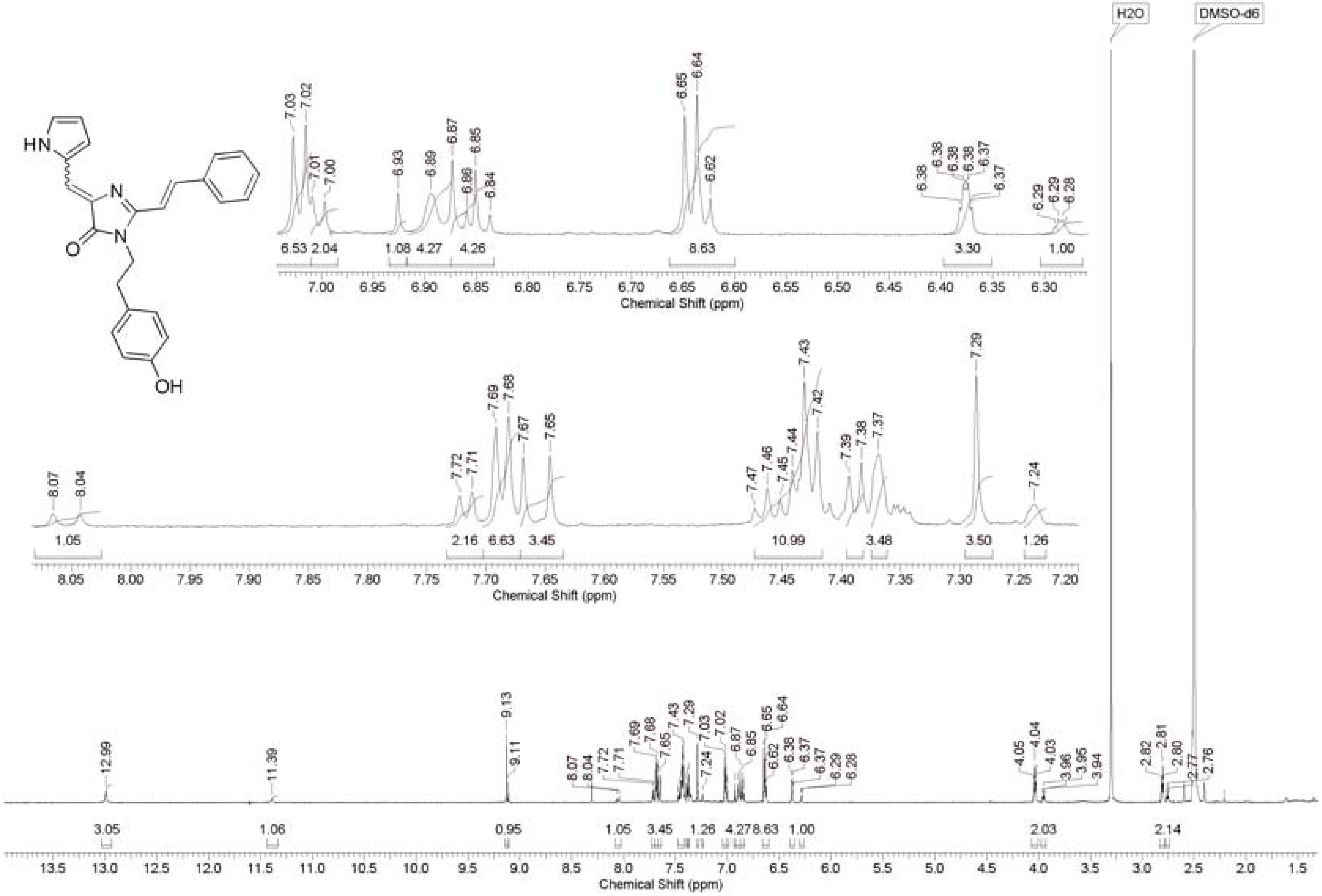
HN
N
N
O
OH
H2O
DMSO-d6
Chemical Shift (ppm)
Chemical Shift (ppm)
Chemical Shift (ppm)

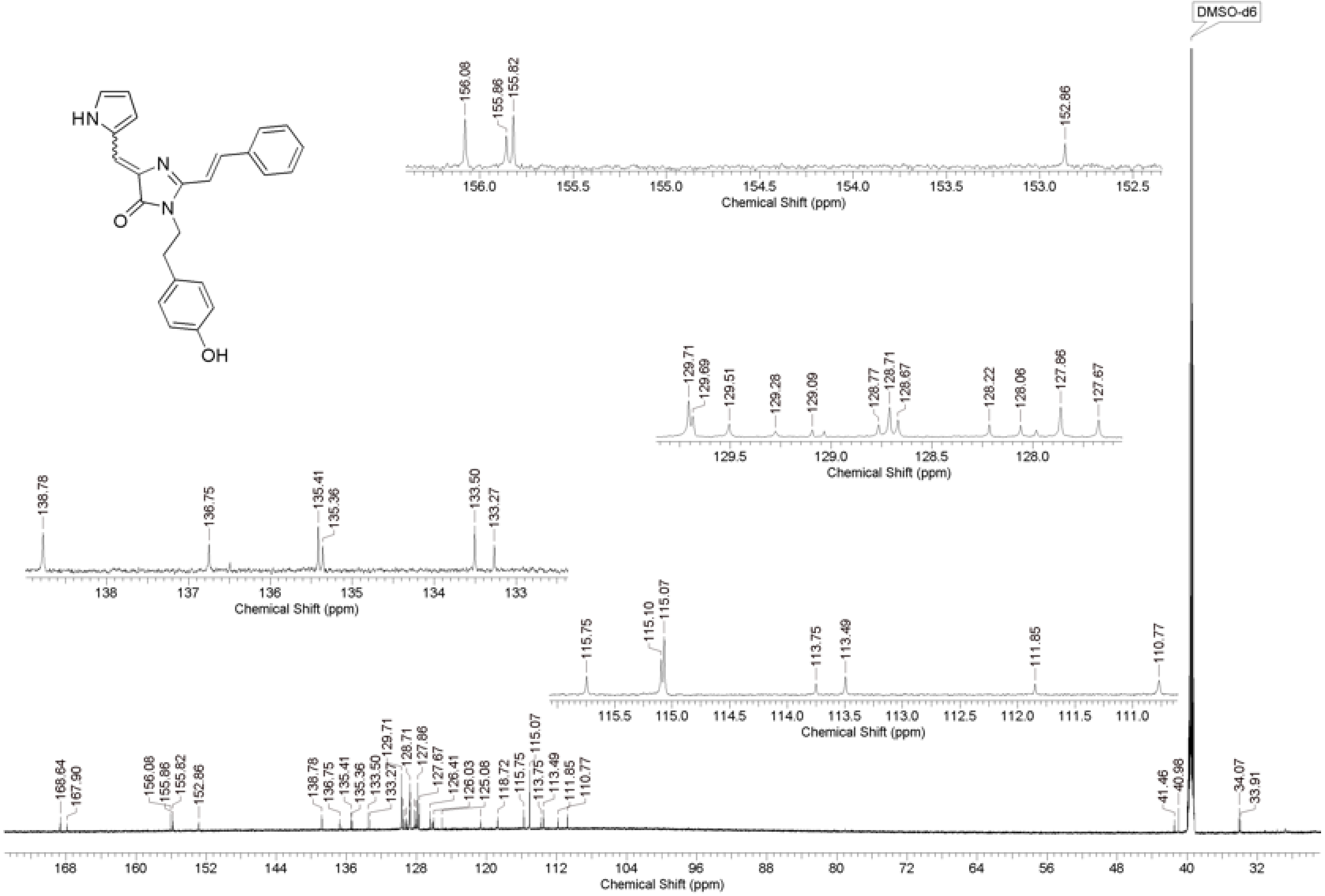
HN
N
N
O
OH
DMSO-d6
156.08
155.86
155.82
152.86
Chemical Shift (ppm)
129.71
129.69
129.51
129.28
129.09
128.77
128.71
128.67
128.22
128.06
127.86
127.67
138.78
136.75
135.41
135.36
133.50
133.27
115.75
115.10
115.07
113.75
113.49
111.85
110.77
168.64
167.90
126.41
126.03
125.08
118.72
41.46
40.98
34.07
33.91

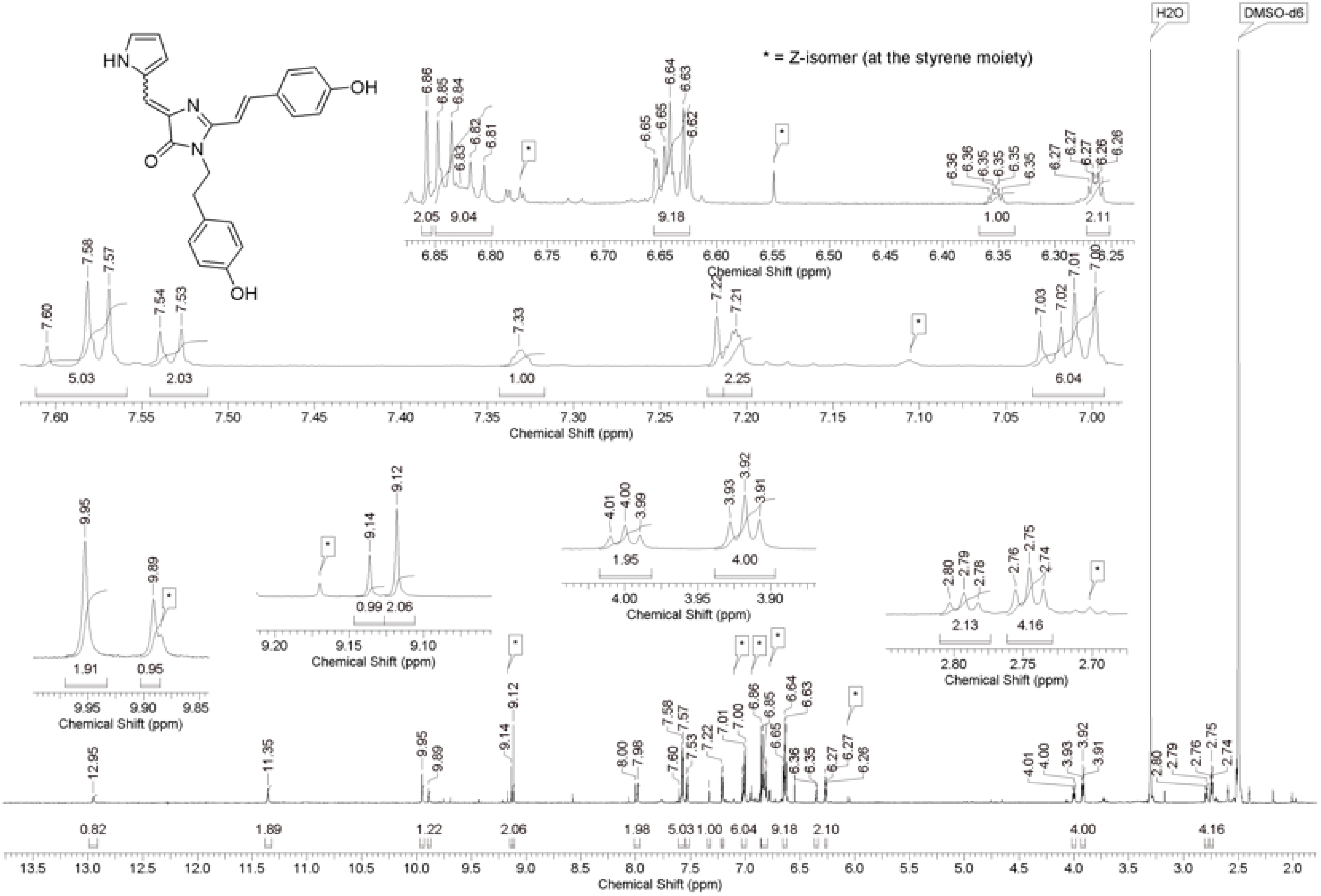
HN
N
N
O
OH
OH
* = Z-isomer (at the styrene moiety)
H2O
DMSO-d6
Chemical Shift (ppm)

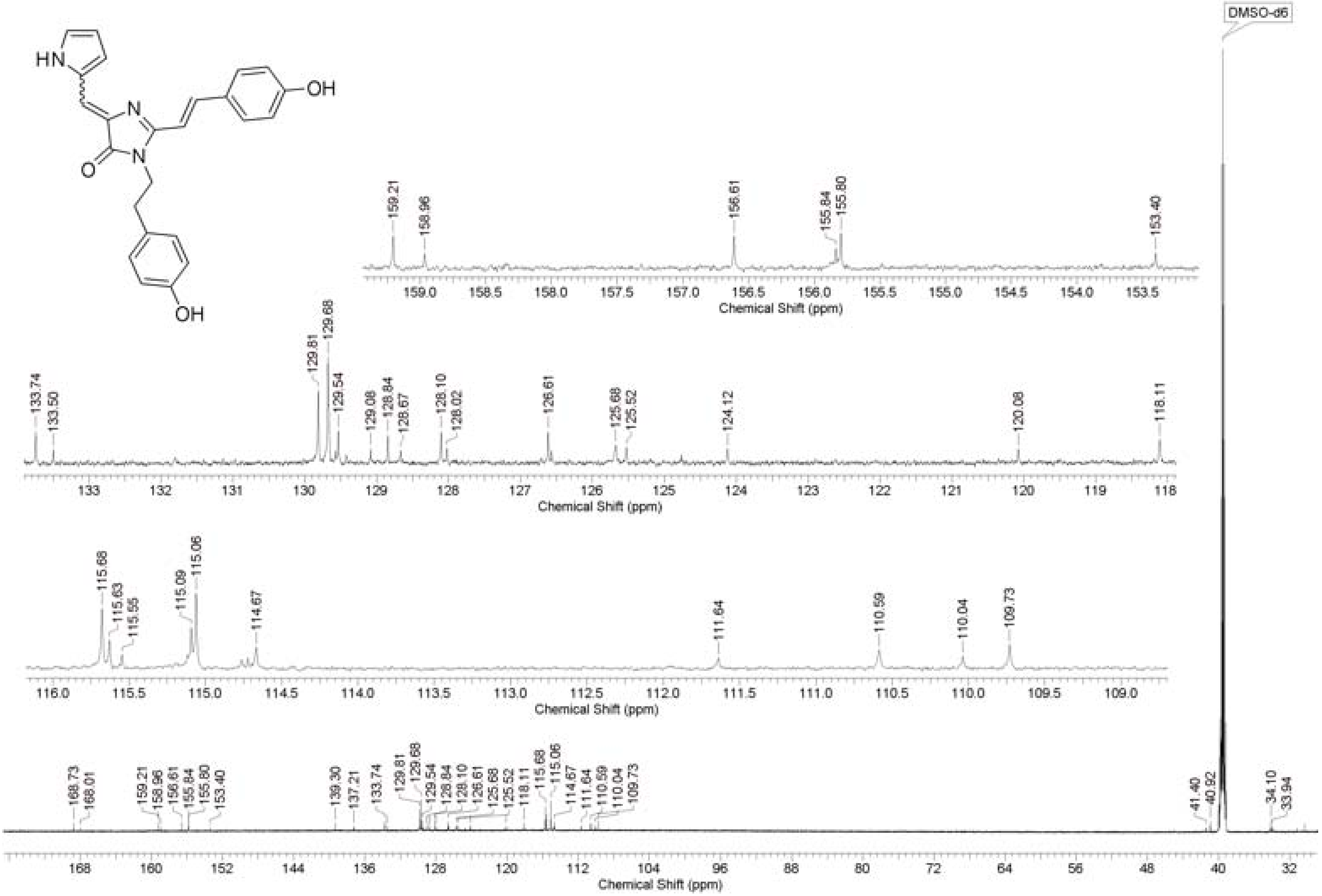

HN
N
OH
O
N
OH
DMSO-d6
159.21
158.96
156.61
155.84
155.80
153.40
Chemical Shift (ppm)
133.74
133.50
129.81
129.68
129.54
129.08
128.84
128.67
128.10
128.02
126.61
125.68
125.52
124.12
120.08
118.11
Chemical Shift (ppm)
115.68
115.63
115.55
115.09
115.06
114.67
111.64
110.59
110.04
109.73
Chemical Shift (ppm)
168.73
168.01
139.30
137.21
41.40
40.92
34.10
33.94
Chemical Shift (ppm)
